# Supplementary material for: Convergent evolution of small molecule pheromones in Pristionchus nematodes
Source: eLife. 2020 Apr 27;9:e55687. doi: 10.7554/eLife.55687 (PMC7224695; doi:10.7554/eLife.55687)
Supplement: Supplementary file 1. — a. NMR spectra of simple ASCR. b. NMR spectra of DASC chemicals. c. NMR spectra of PASC chemicals. d. NMR spectra of UBAS chemicals. e. NMR spectra of UPAS chemicals. [file elife-55687-supp1.pdf]

supplementary file 1: NMR spectra of isolated or enriched NDMMs	Pages
**supplementary file 1a.** NMR spectra of simple ASCR	S2
**supplementary file 1b.** NMR spectra of DASC chemicals	S27
**supplementary file 1c.** NMR spectra of PASC chemicals	S75
**supplementary file 1d.** NMR spectra of UBAS chemicals	S89
**supplementary file 1e.** NMR spectra of UPAS chemicals	S132

supplementary file 1a: NMR spectra of simple ASCR Pages

Figures 1-4. NMR spectra of ascr#9 (800 MHz, CD_3OD) isolated from *P. entomophagus*. S3
Figures 5-9. NMR spectra of ascr#1 (800 MHz, CD_3OD) isolated from *P. triformis*. S7
Figures 10-11. NMR spectra of ascr#7 (800 MHz, CD_3OD) isolated from *P. uniformis*. S12
Figures 12-15. NMR spectra of ascr#10 (800 MHz, CD_3OD) isolated from *P. taiwanensis*. S14
Figures 16-17. NMR spectra of ascr#10 (800 MHz, CD_3OD) isolated from *P. laevicollis*. S18
Figures 18-22. NMR spectra of ascr#10 (800 MHz, CD_3OD) isolated from *P. maxplancki*. S20
Figures 23-24. NMR spectra of ascr#10 (800 MHz, CD_3OD) isolated from *P. fukushimae*. S25

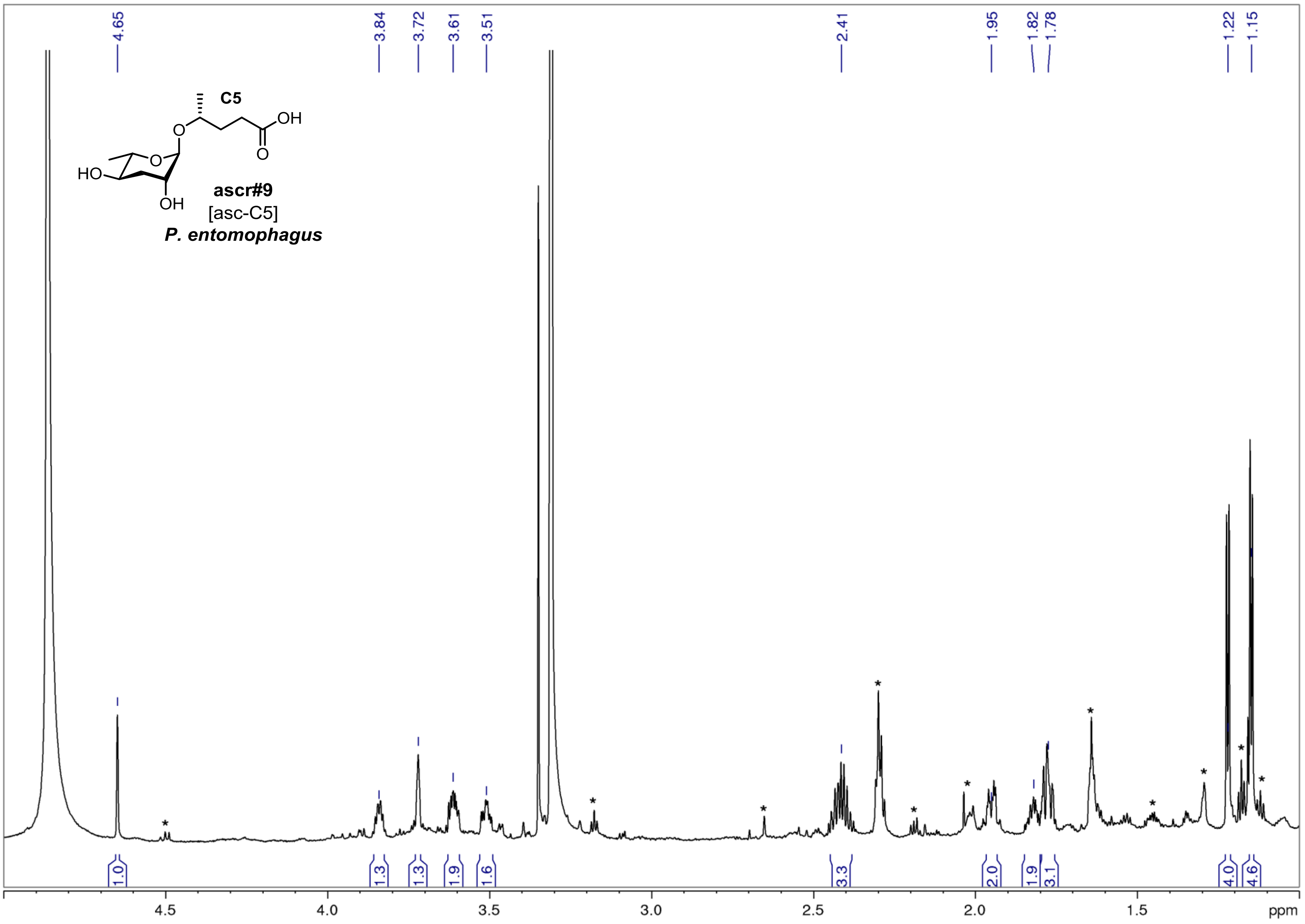

Figure 1. ^{1}H NMR spectrum of ascr#9 [asc-C5] (800 MHz, CD_3OD) isolated from the *exo*-metabolome of *P. entomophagus*.

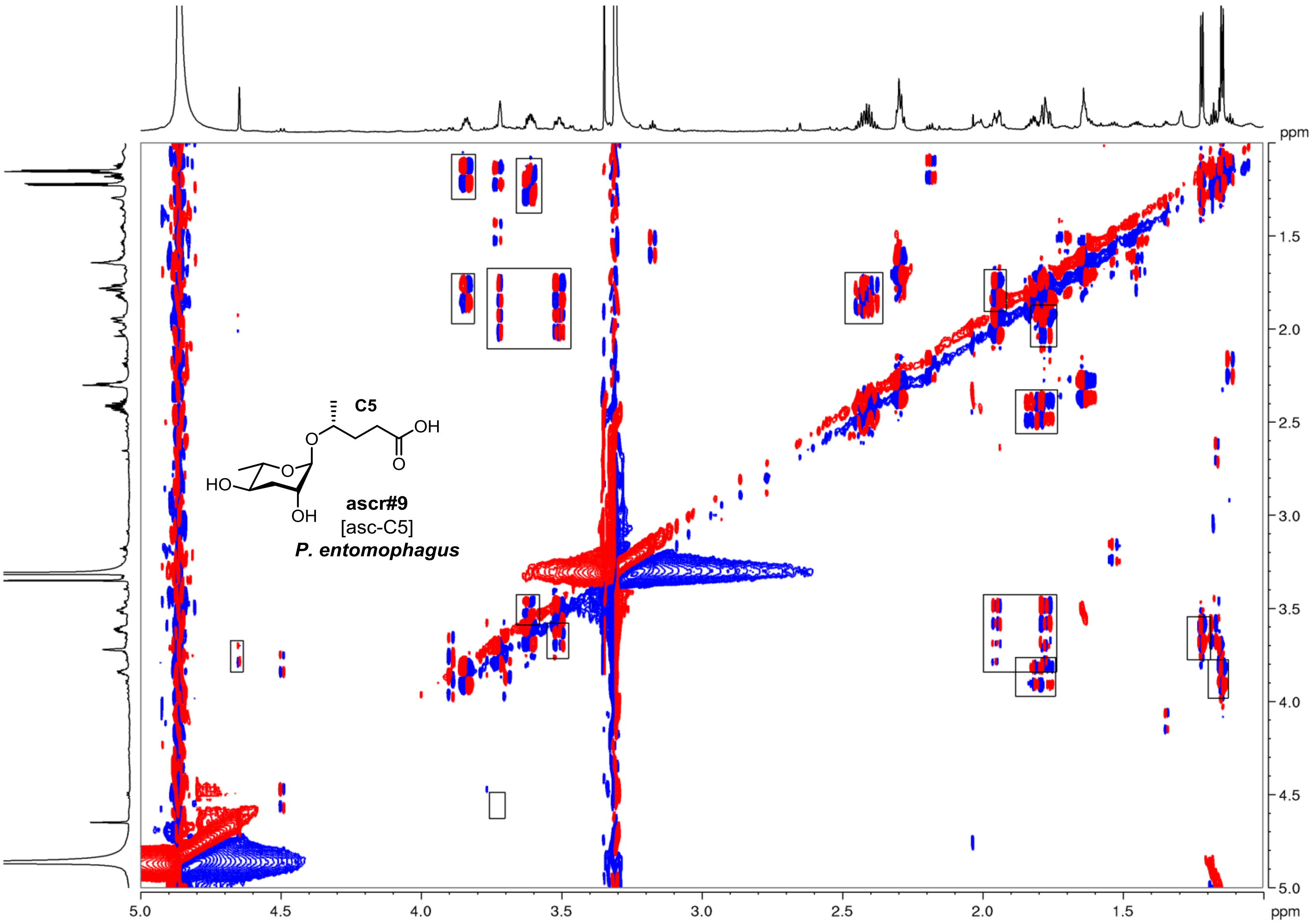

Figure 2. *dqf*-COSY spectrum of ascr#9 [asc-C5] (800 MHz, CD_3OD) isolated from the *exo*-metabolome of *P. entomophagus*. Boxed cross peaks are derived from ascr#9.

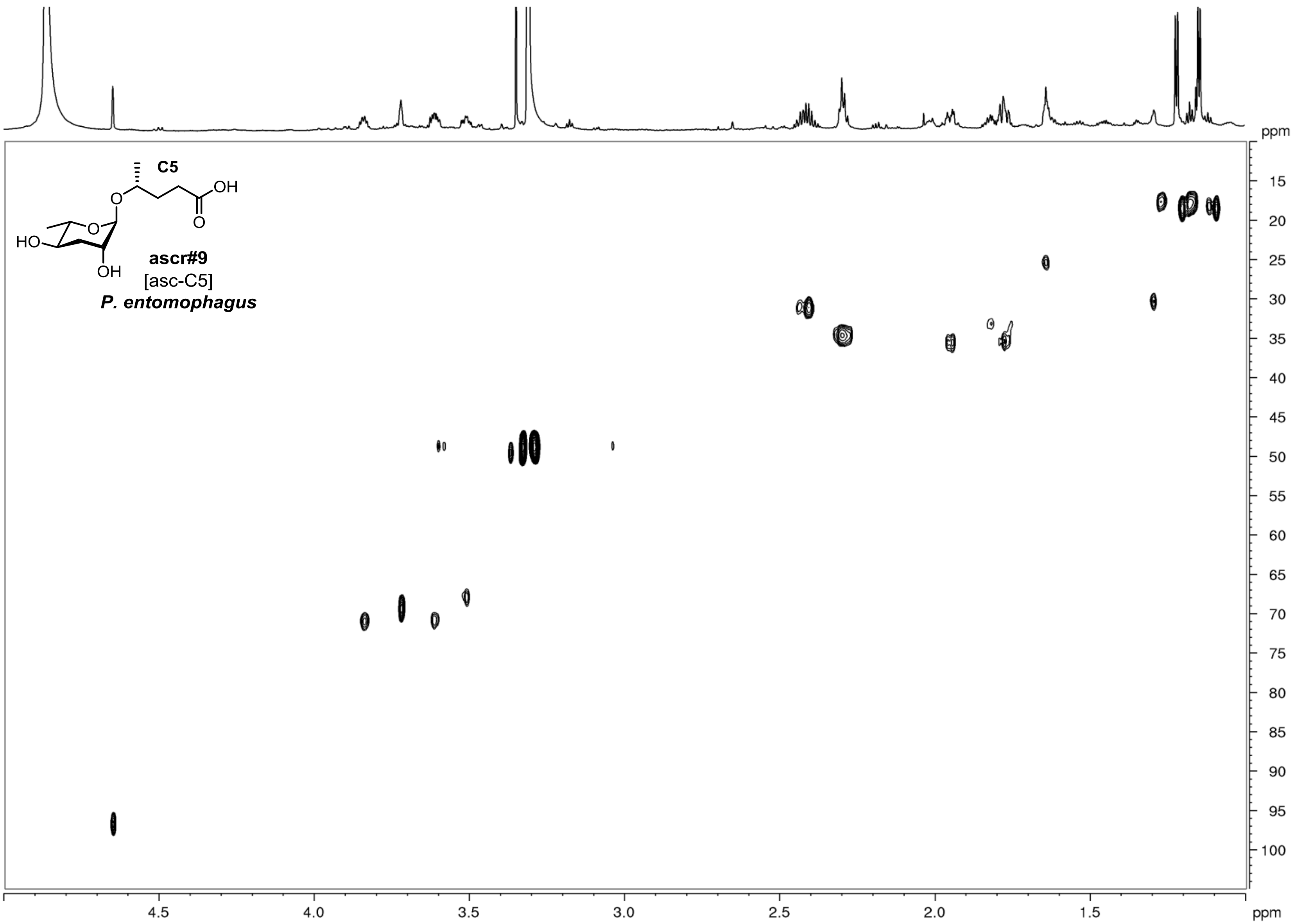

Figure 3. HSQC spectrum of ascr#9 [asc-C5] (800 MHz, CD_3OD) isolated from the *exo*-metabolome of *P. entomophagus*.

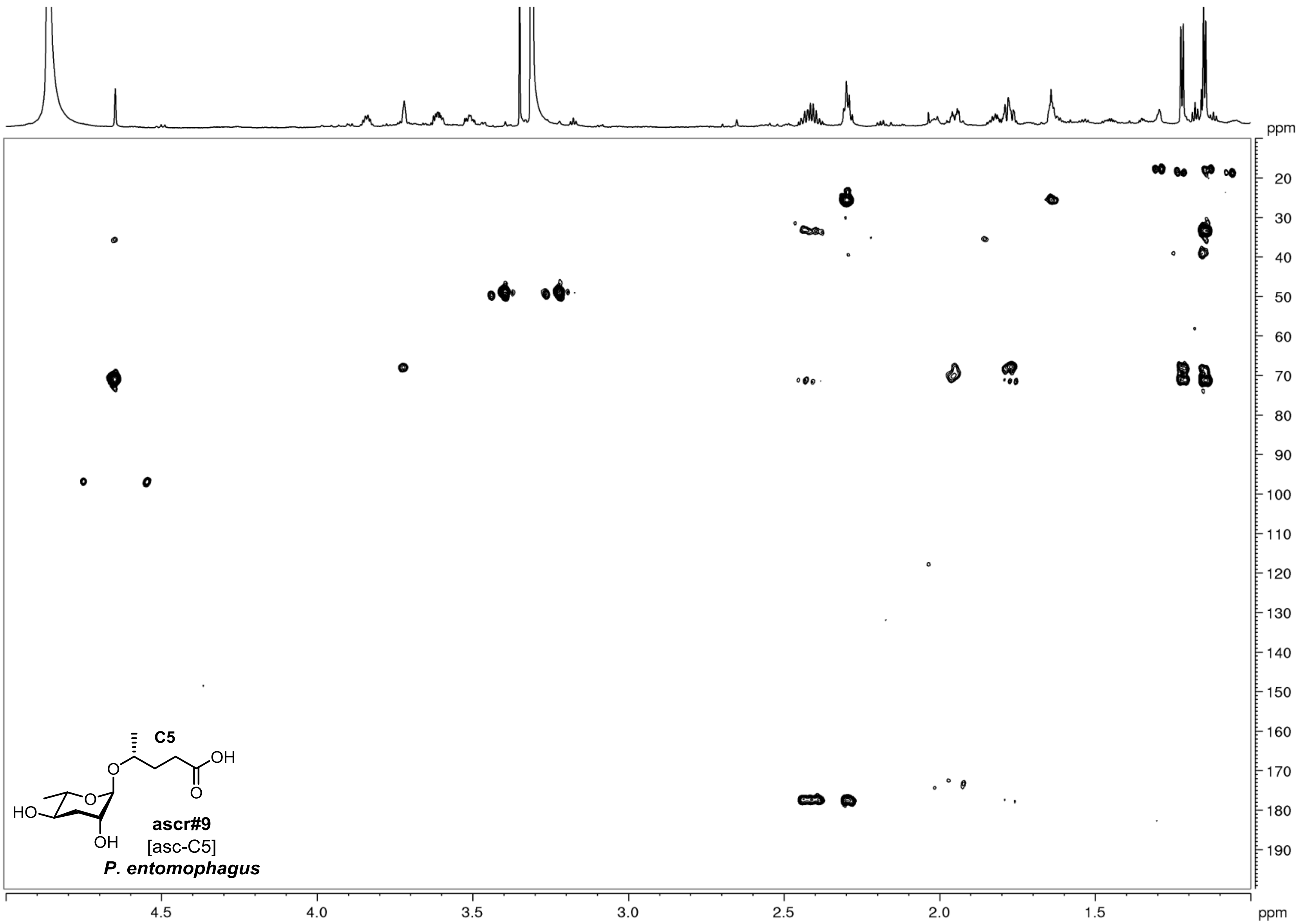

Figure 4. HMBC spectrum of ascr#9 [asc-C5] (800 MHz, CD_3OD) isolated from the *exo*-metabolome of *P. entomophagus*.

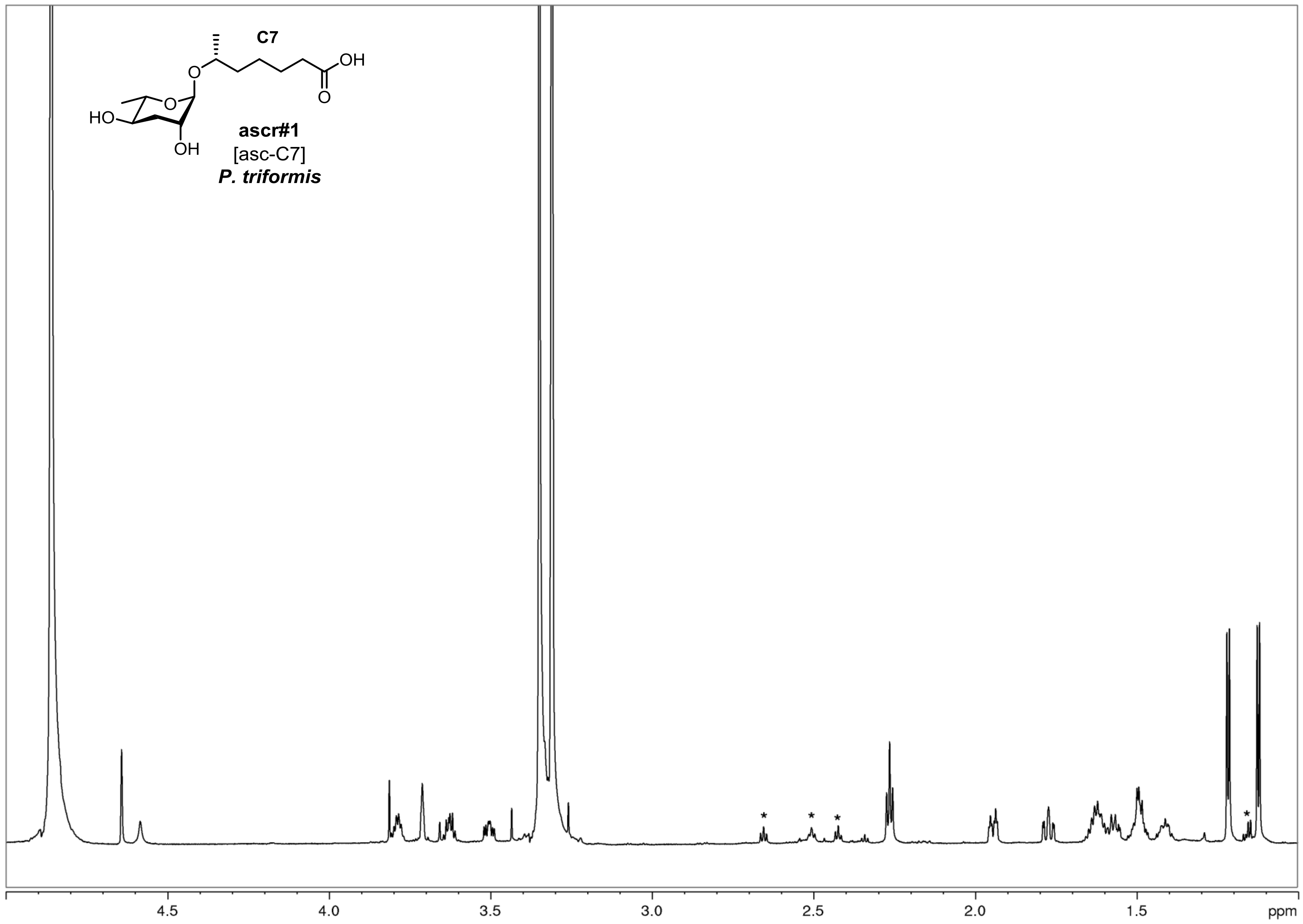

Figure 5. ^{1}H NMR spectrum of ascr#1 [asc-C7] (800 MHz, CD_3OD) isolated from the *exo*-metabolome of *P. triformis*. Asterisks marked peaks are derived from impurities.

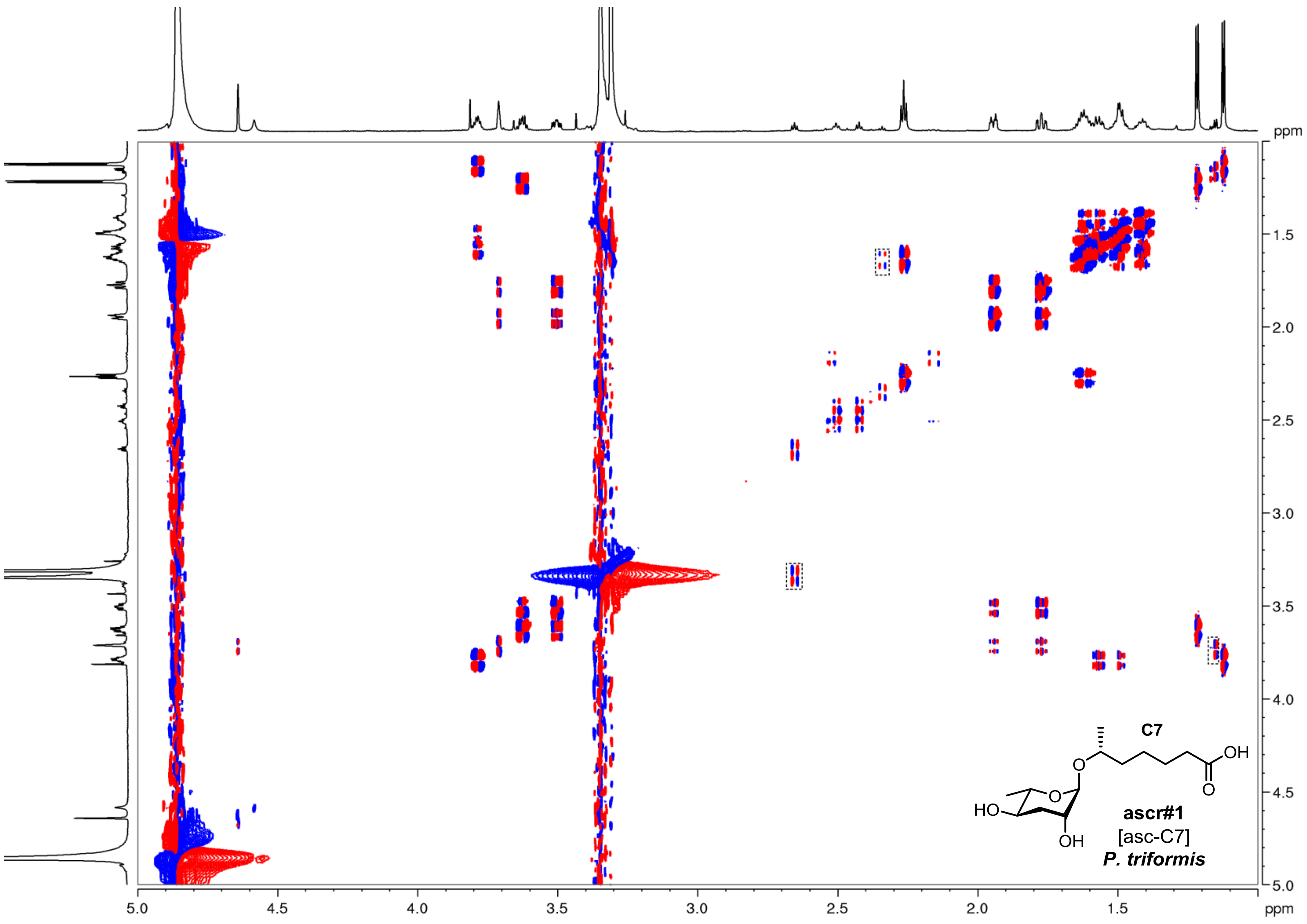

Figure 6. *dqf*-COSY spectrum of ascr#1 [asc-C7] (800 MHz, CD_3OD) isolated from the *exo*-metabolome of *P. triformis*. Dashed line boxed signals are derived from impurities.

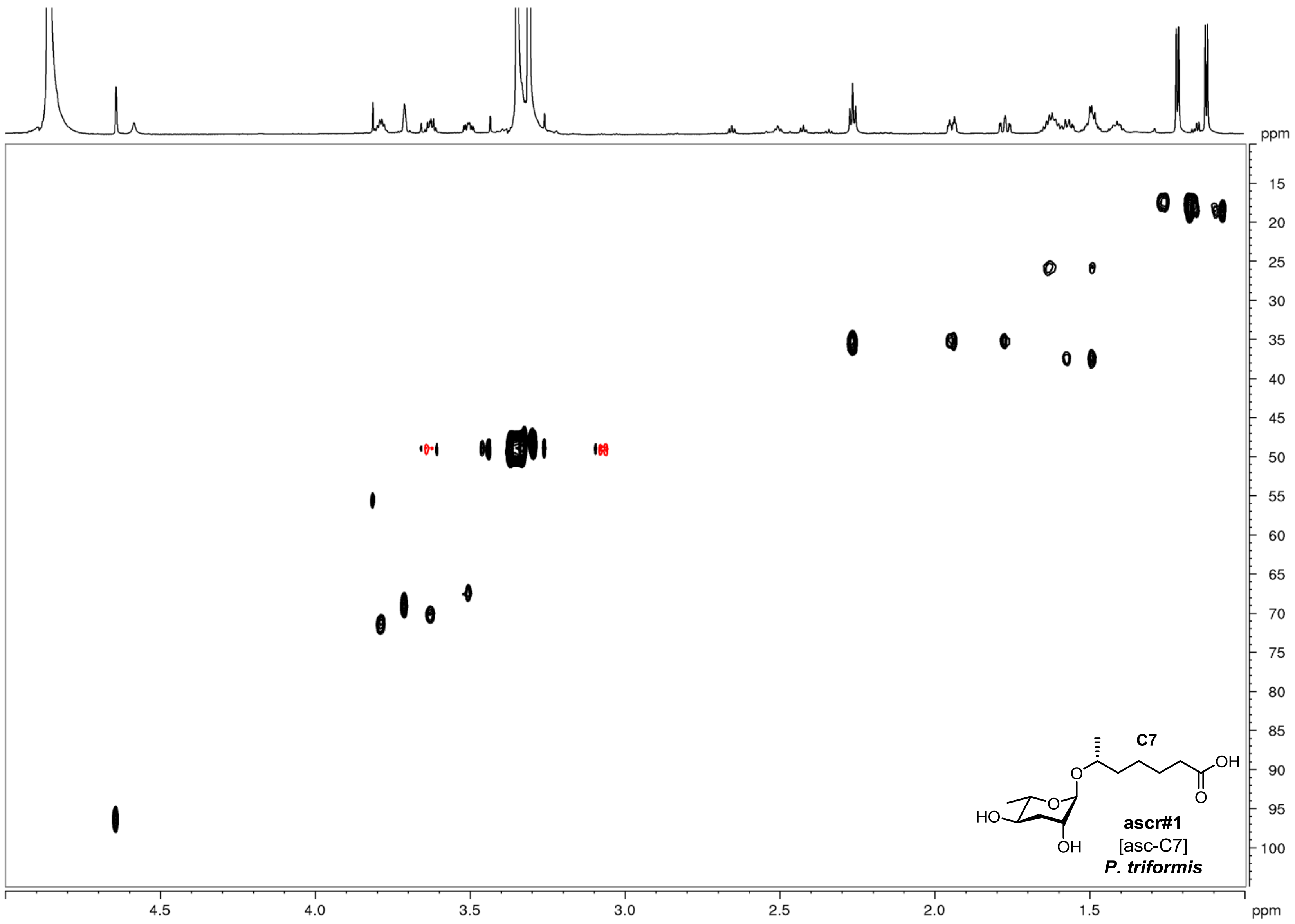

Figure 7. HSQC spectrum of ascr#1 [asc-C7] (800 MHz, CD_3OD) isolated from the *exo*-metabolome of *P. triformis*.

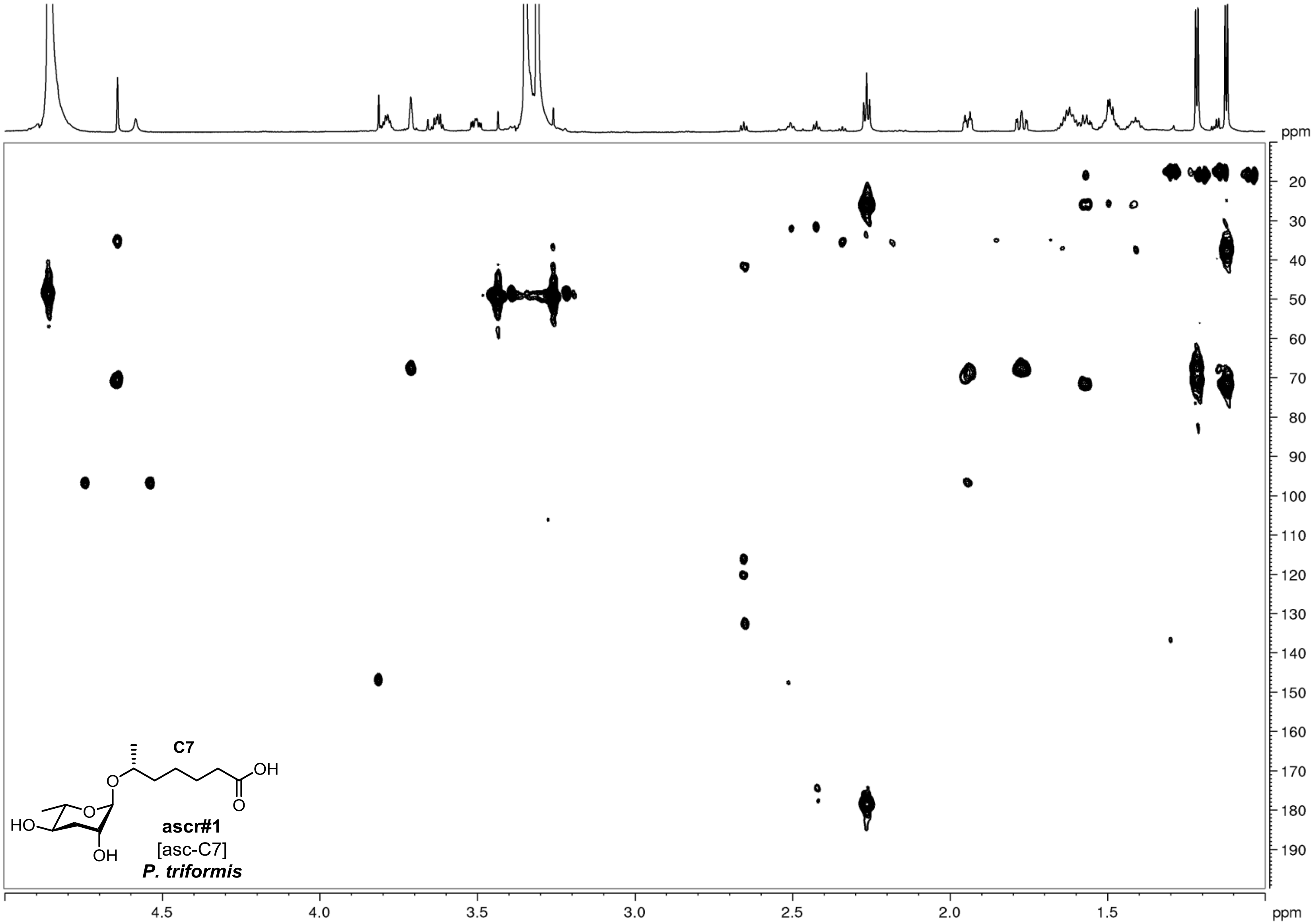

Figure 8. HMBC spectrum of ascr#1 [asc-C7] (800 MHz, CD_3OD) isolated from the *exo*-metabolome of *P. triformis*.

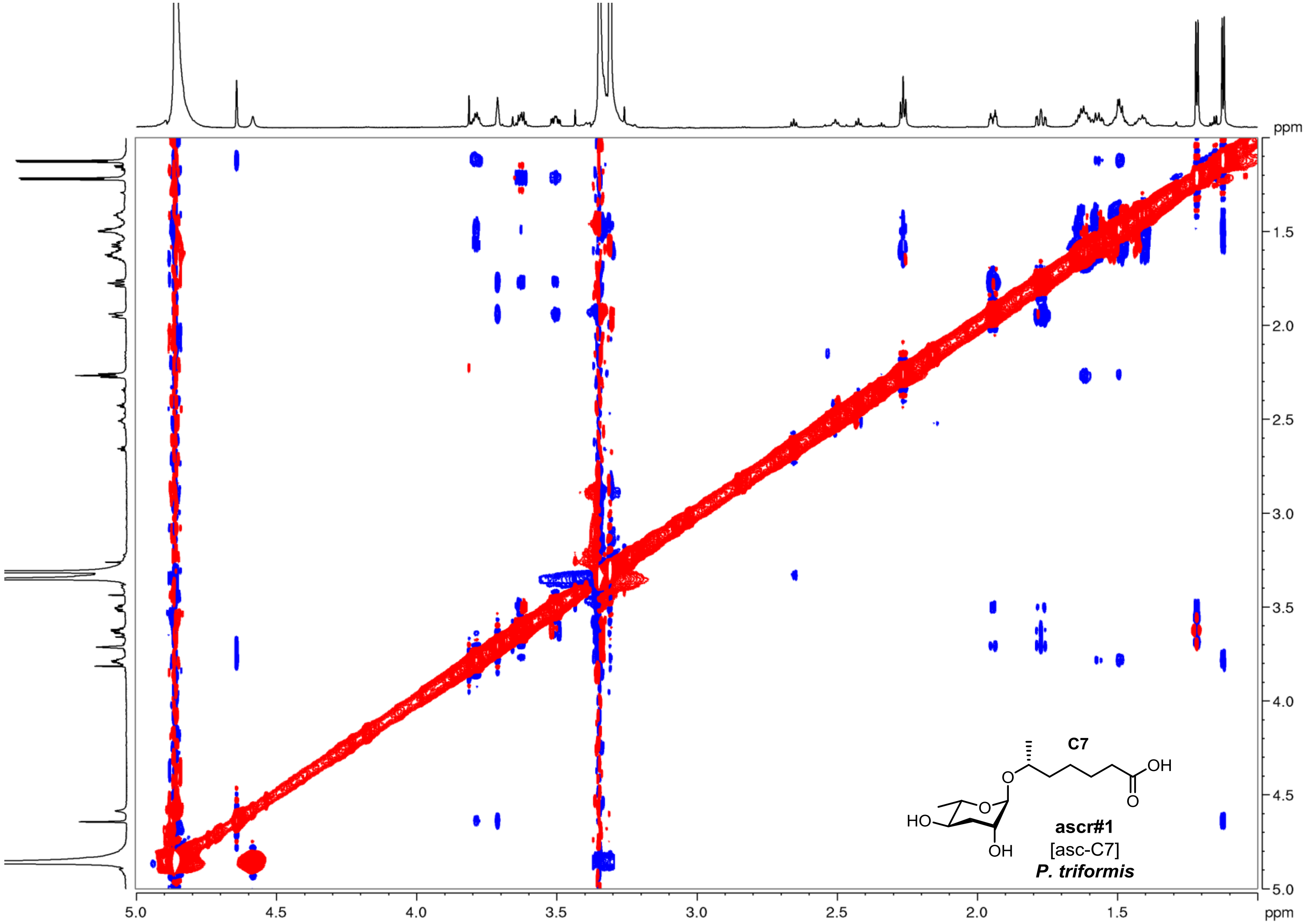

Figure 9. NOESY spectrum of ascr#1 [asc-C7] (800 MHz, CD_3OD) isolated from the *exo*-metabolome of *P. triformis*.

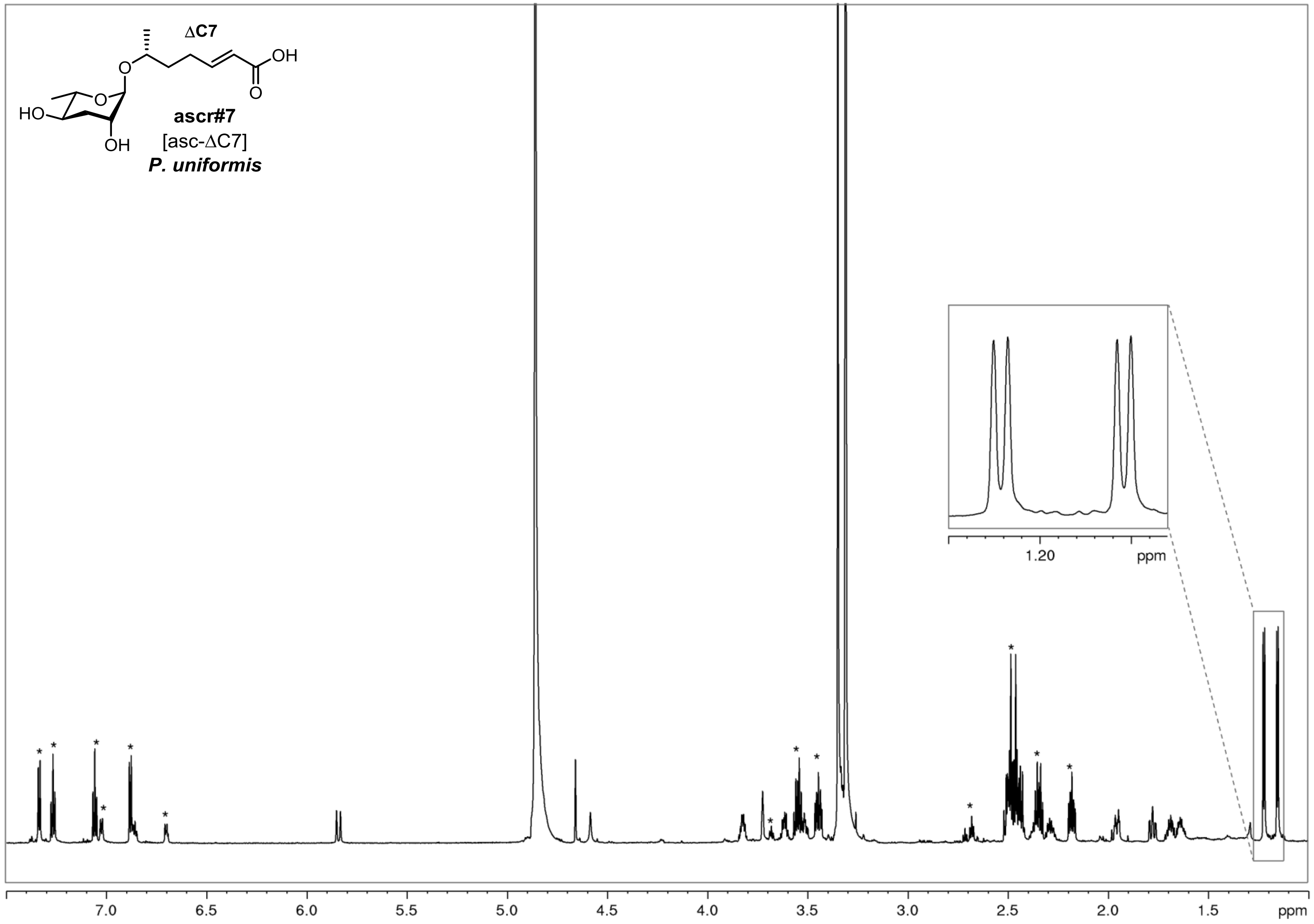

Figure 10. ^{1}H NMR spectrum of an HPLC fraction containing ascr#7 [asc-ΔC7] (800 MHz, CD_3OD) isolated from the *exo*-metabolome of *P. uniformis*. Asteriks marked peaks are derived from impurities.

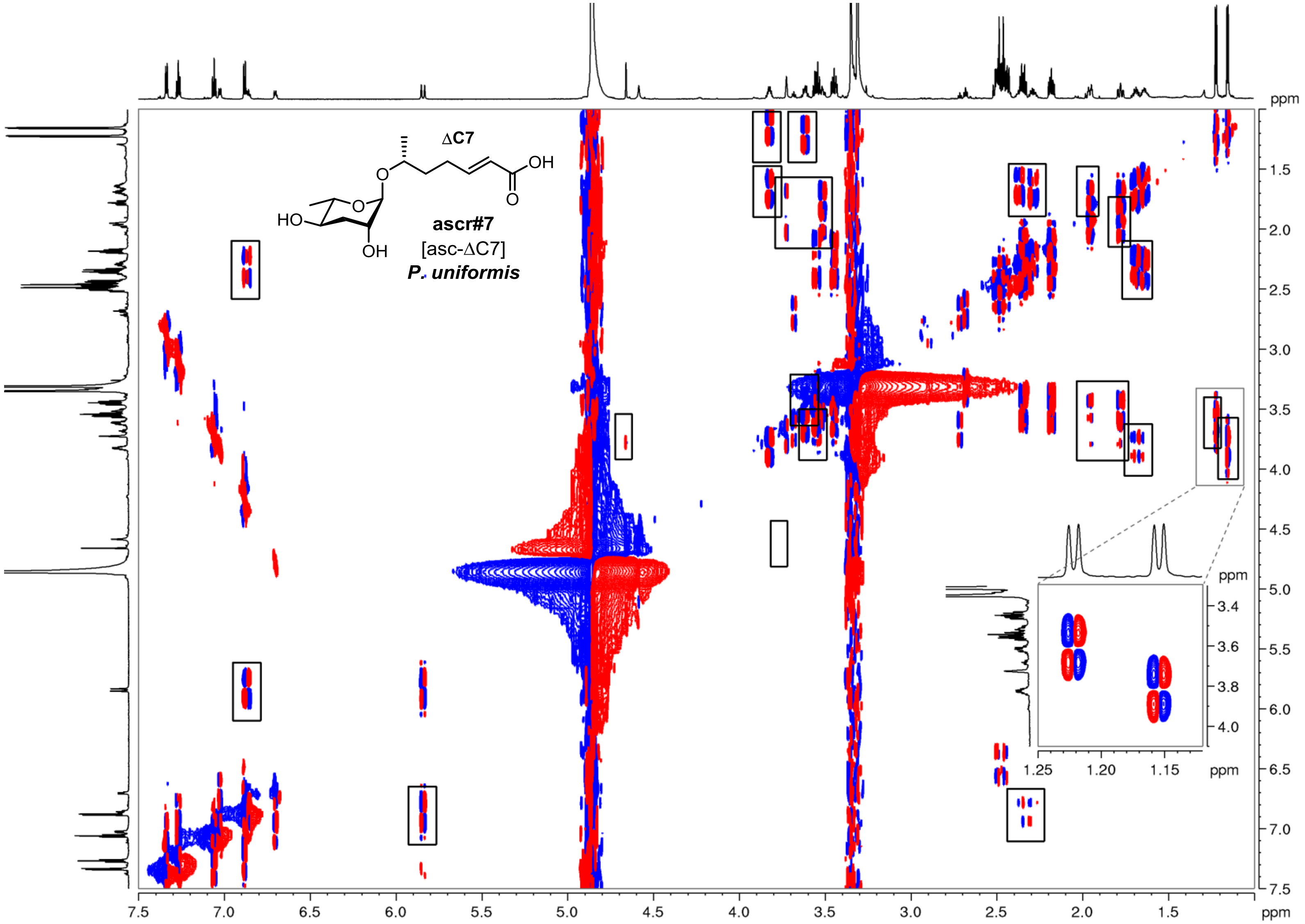

Figure 11. *dqf*-COSY spectrum of an HPLC fraction containing ascr#7 [asc-ΔC7] (800 MHz, CD_3OD) isolated from the *exo*-metabolome of *P. uniformis*. Black boxed signals are derived from ascr#7 [asc-ΔC7].

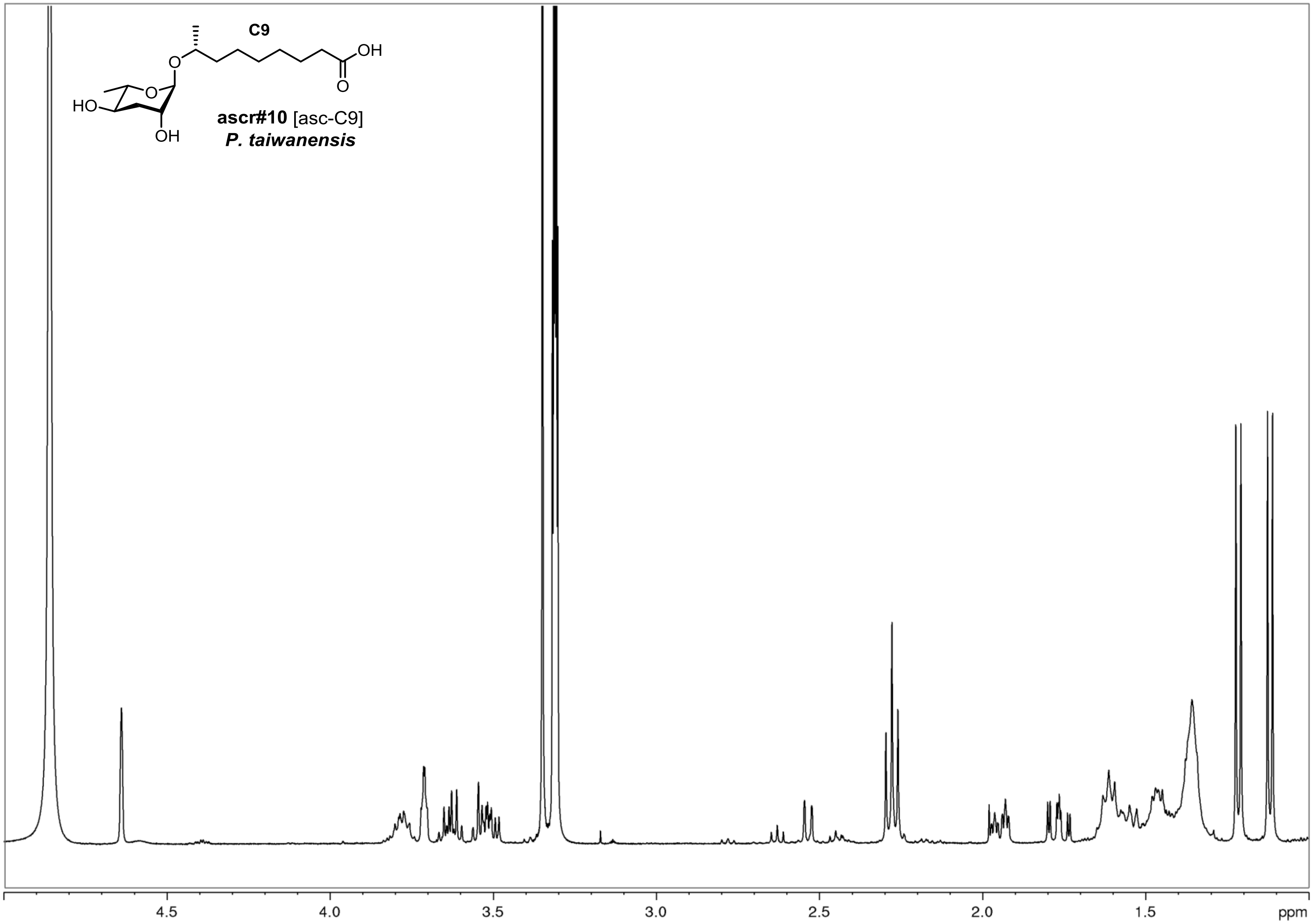

Figure 12. ^{1}H NMR spectrum of ascr#10 [asc-C9] (800 MHz, CD_3OD) isolated from the *exo*-metabolome of *P. taiwanensis*.

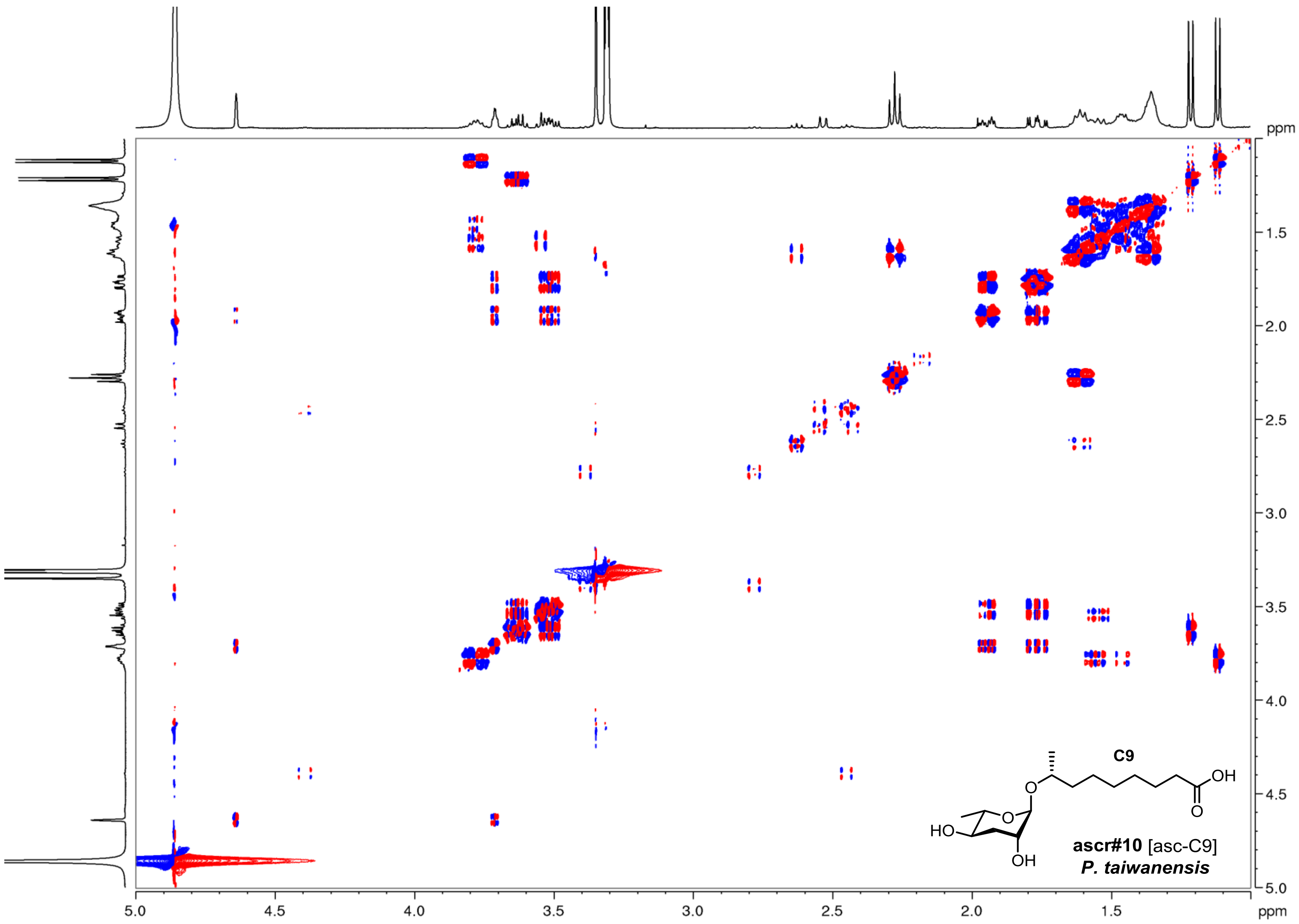

Figure 13. *dqf*-COSY spectrum of ascr#10 [asc-C9] (800 MHz, CD_3OD) isolated from the *exo*-metabolome of *P. taiwanensis*.

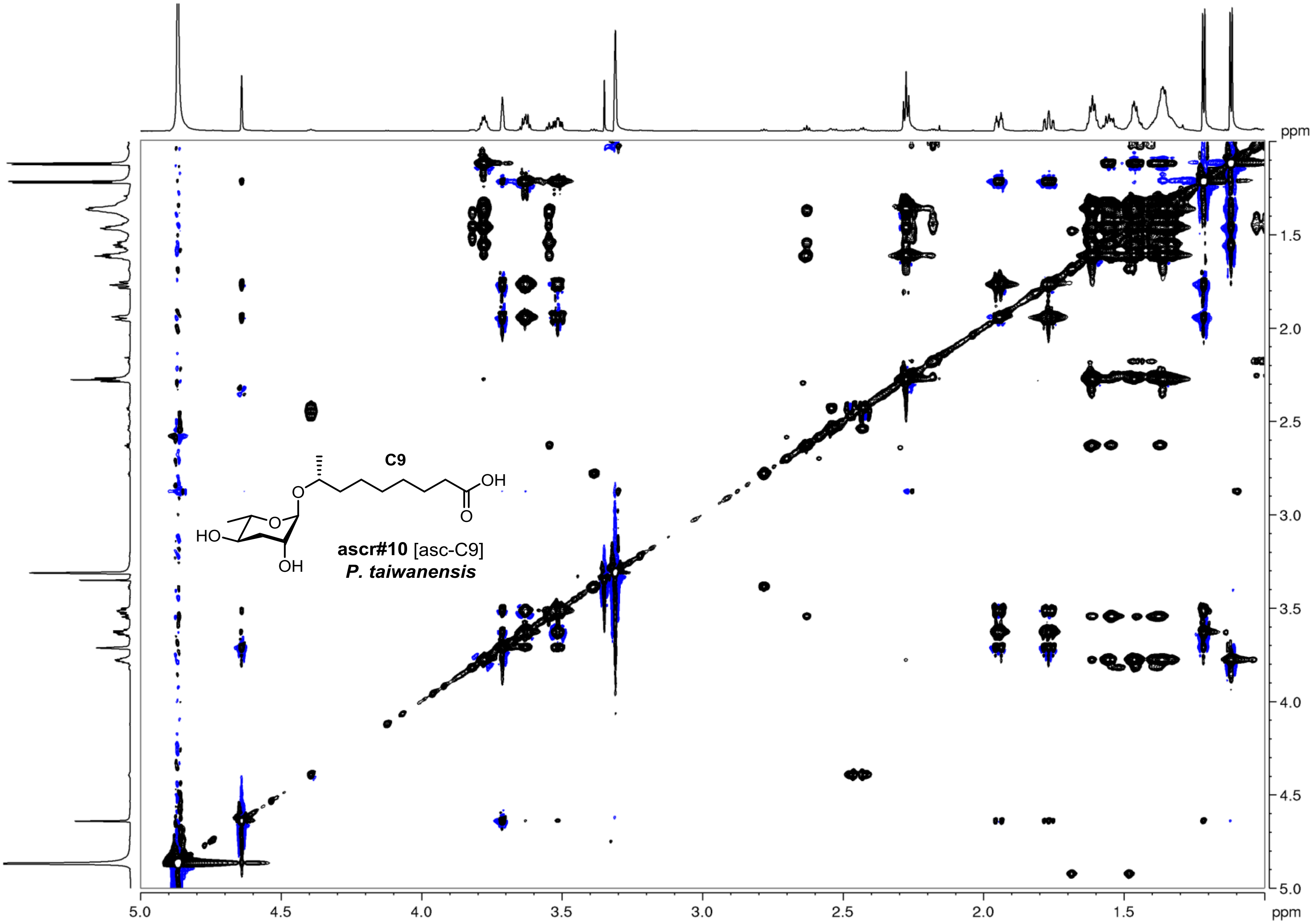

Figure 14. TOCSY spectrum of ascr#10 [asc-C9] (800 MHz, CD_3OD) isolated from the *exo*-metabolome of *P. taiwanensis*.

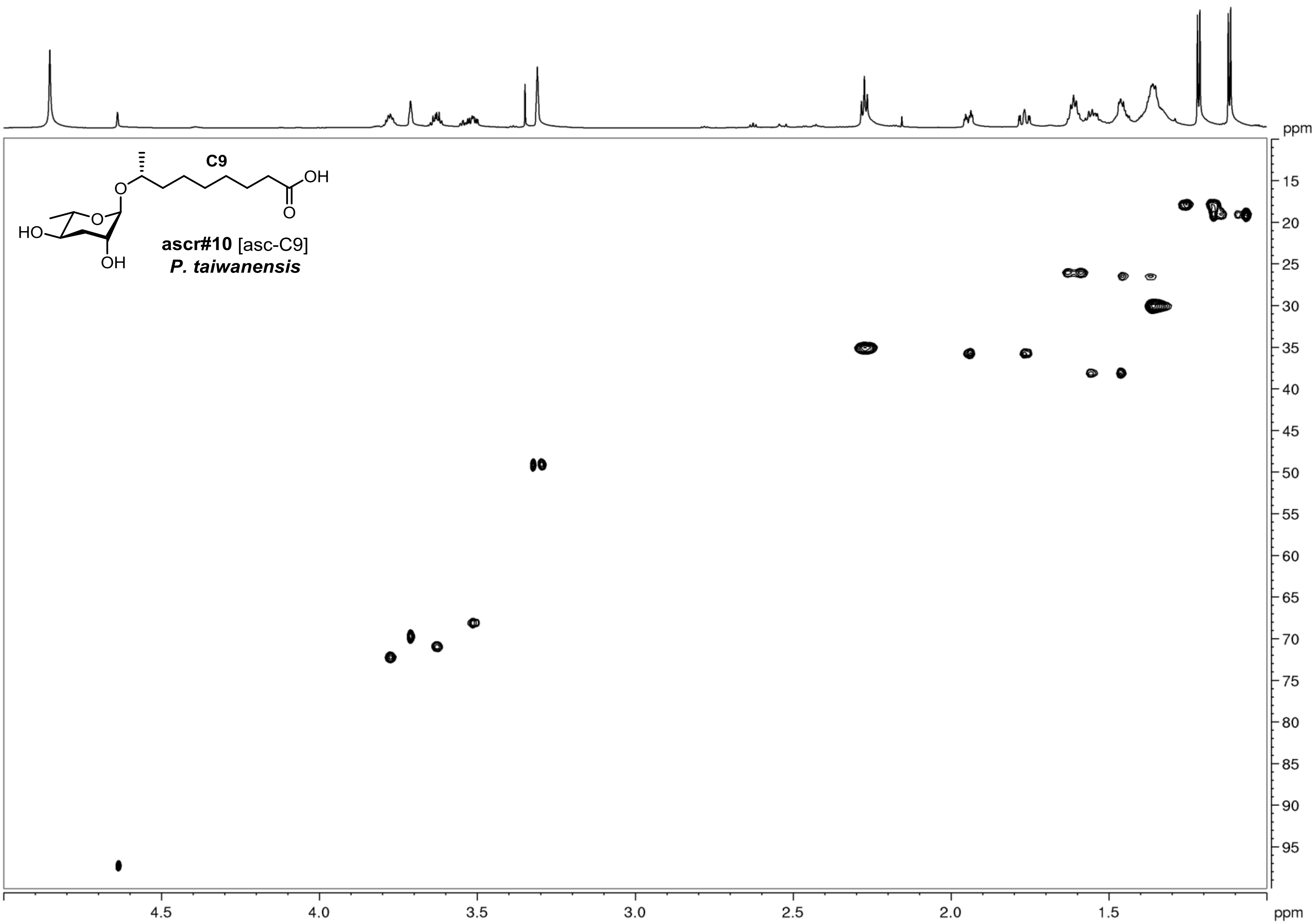

Figure 15. HSQC spectrum of ascr#10 [asc-C9] (800 MHz, CD_3OD) isolated from the *exo*-metabolome of *P. taiwanensis*.

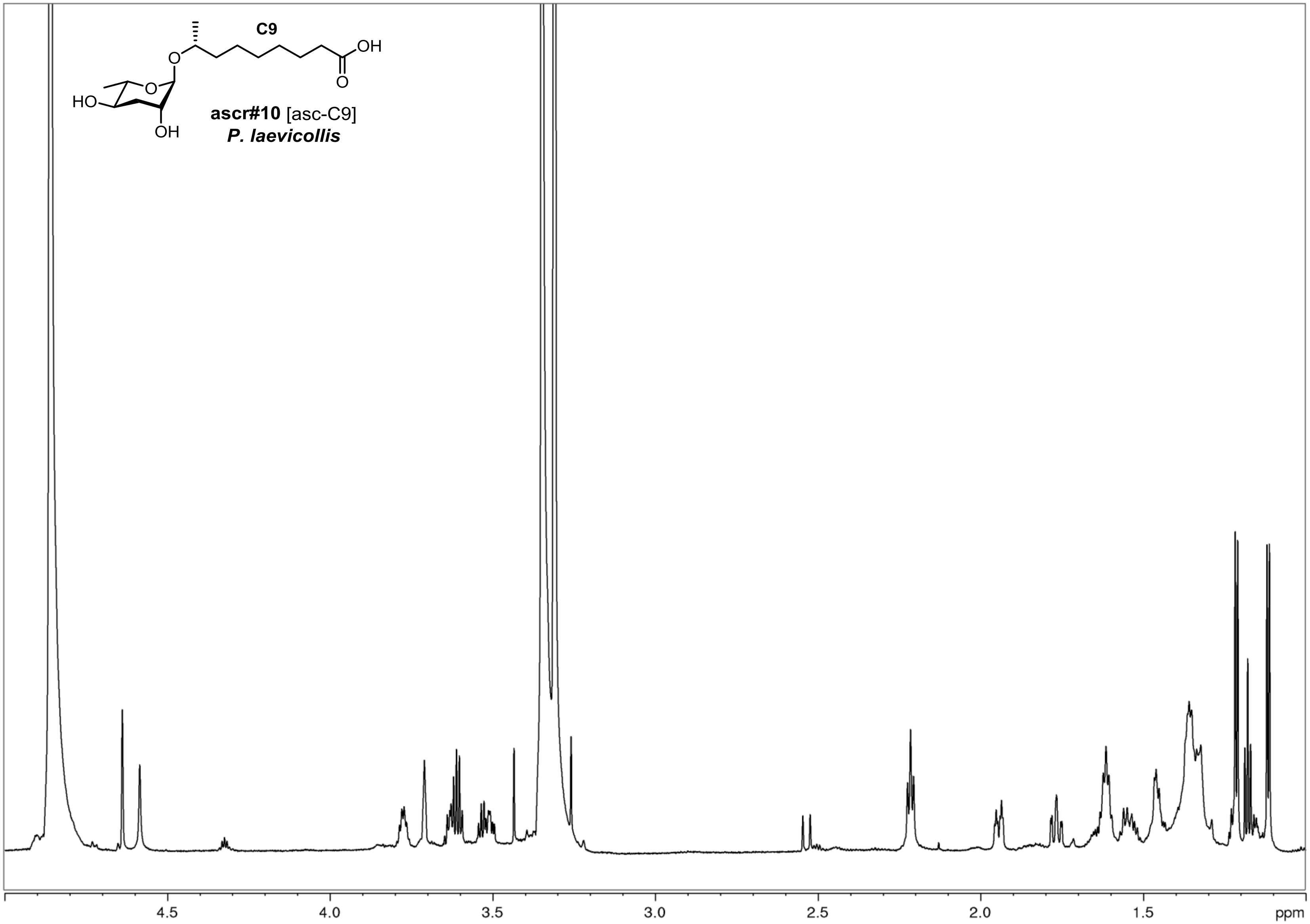

Figure 16. ^{1}H NMR spectrum of ascr#10 [asc-C9] (800 MHz, CD_3OD) isolated from the *exo*-metabolome of *P. laevicollis*.

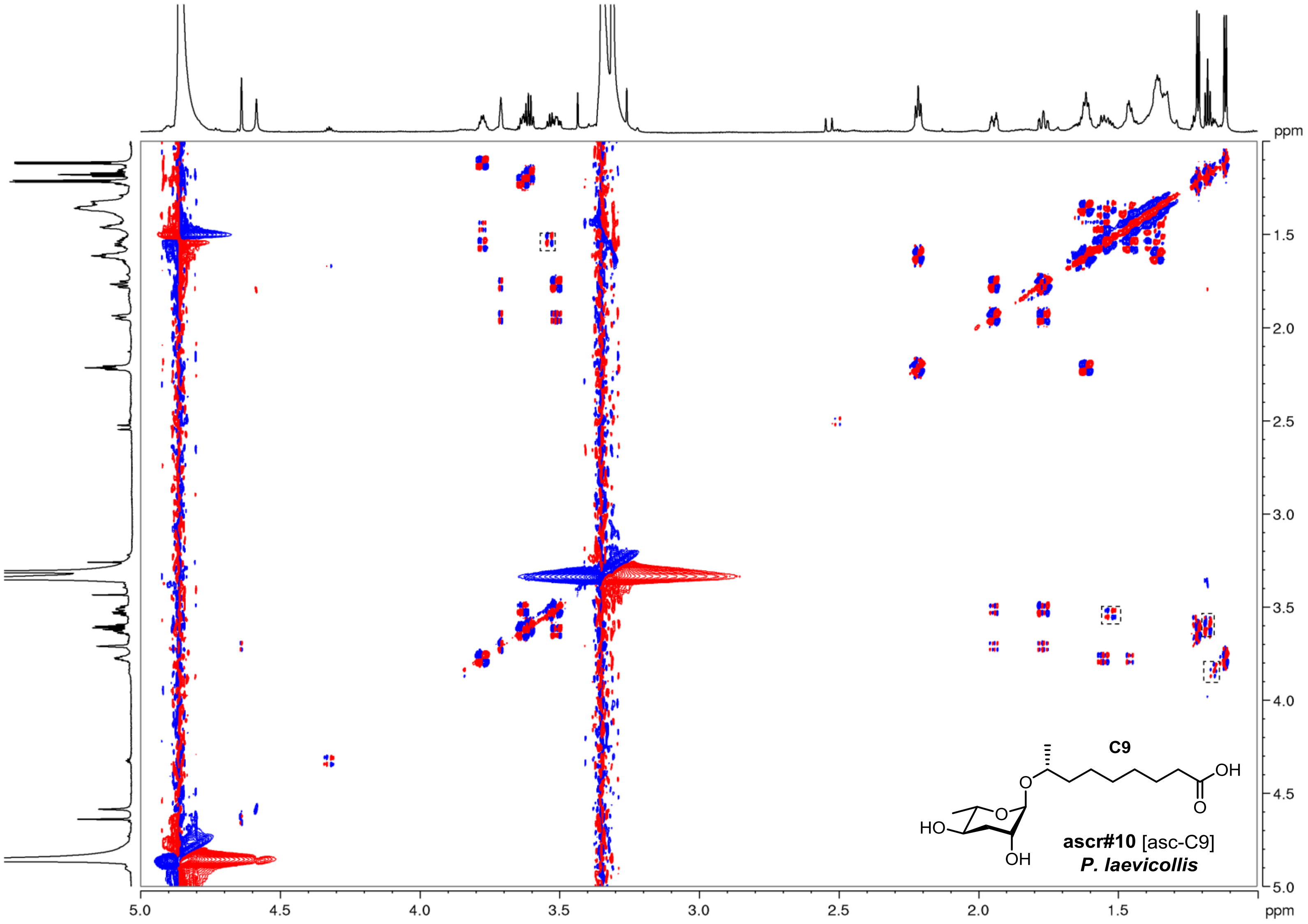

Figure 17. *dqf*-COSY spectrum of ascr#10 [asc-C9] (800 MHz, CD_3OD) isolated from the *exo*-metabolome of *P. laevicollis*. Dashed line boxed signals are derived from impurities.

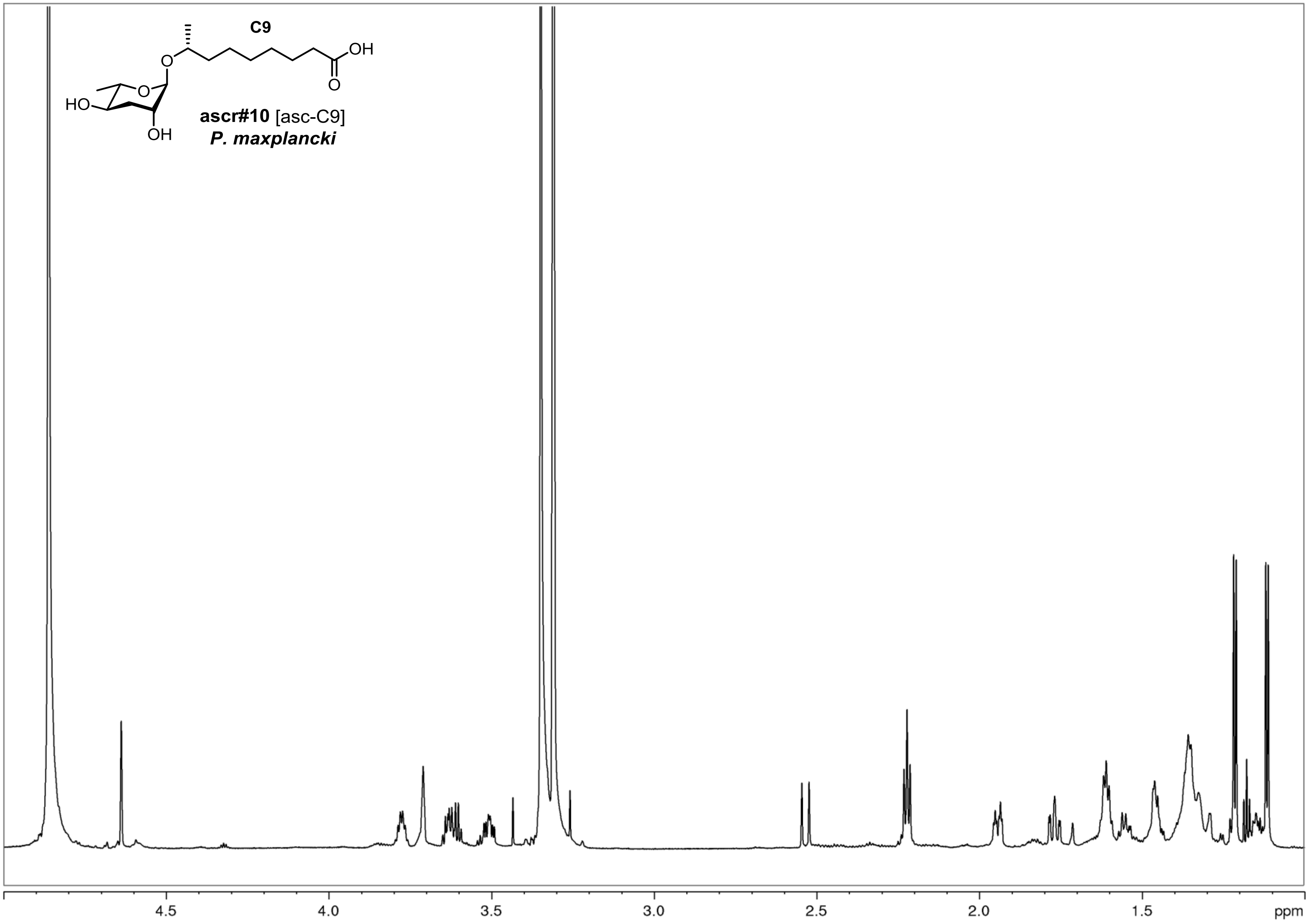

Figure 18. ^{1}H NMR spectrum of ascr#10 [asc-C9] (800 MHz, CD_3OD) isolated from the *exo*-metabolome of *P. maxplancki*.

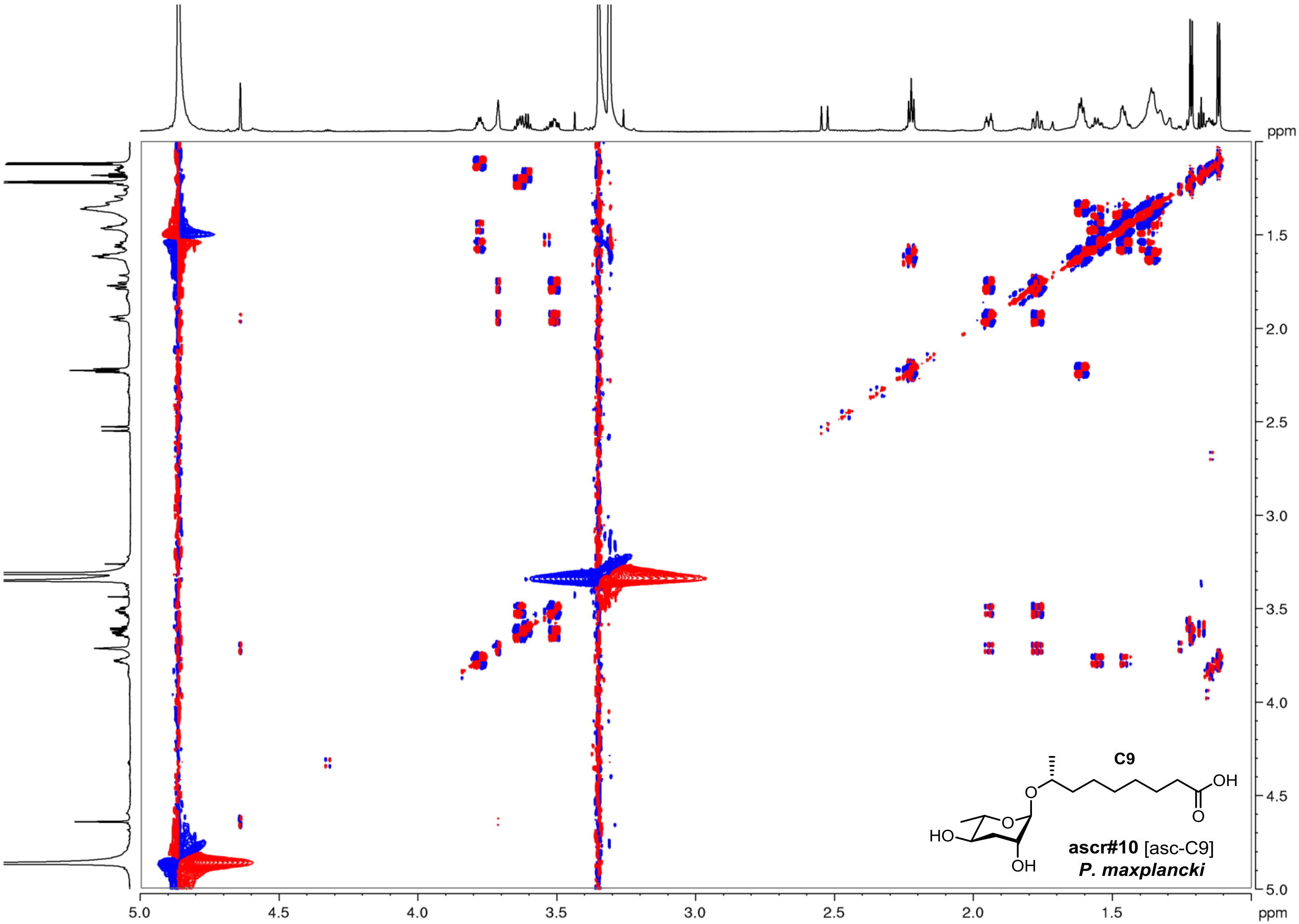

Figure 19. *dqf*-COSY spectrum of ascr#10 [asc-C9] (800 MHz, CD_3OD) isolated from the *exo*-metabolome of *P. maxplancki*.

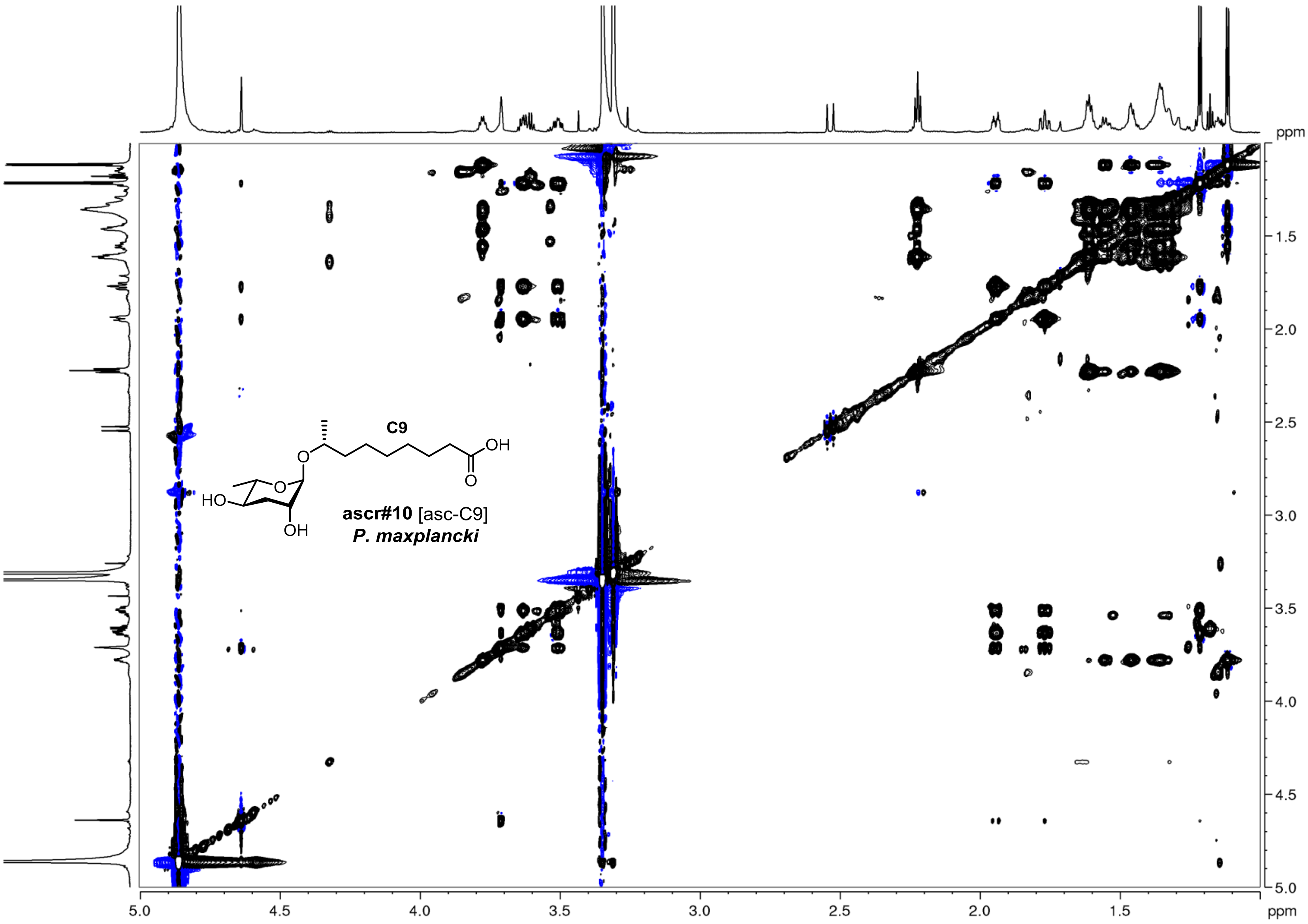

Figure 20. TCOSY spectrum of ascr#10 [asc-C9] (800 MHz, CD_3OD) isolated from the *exo*-metabolome of *P. maxplancki*.

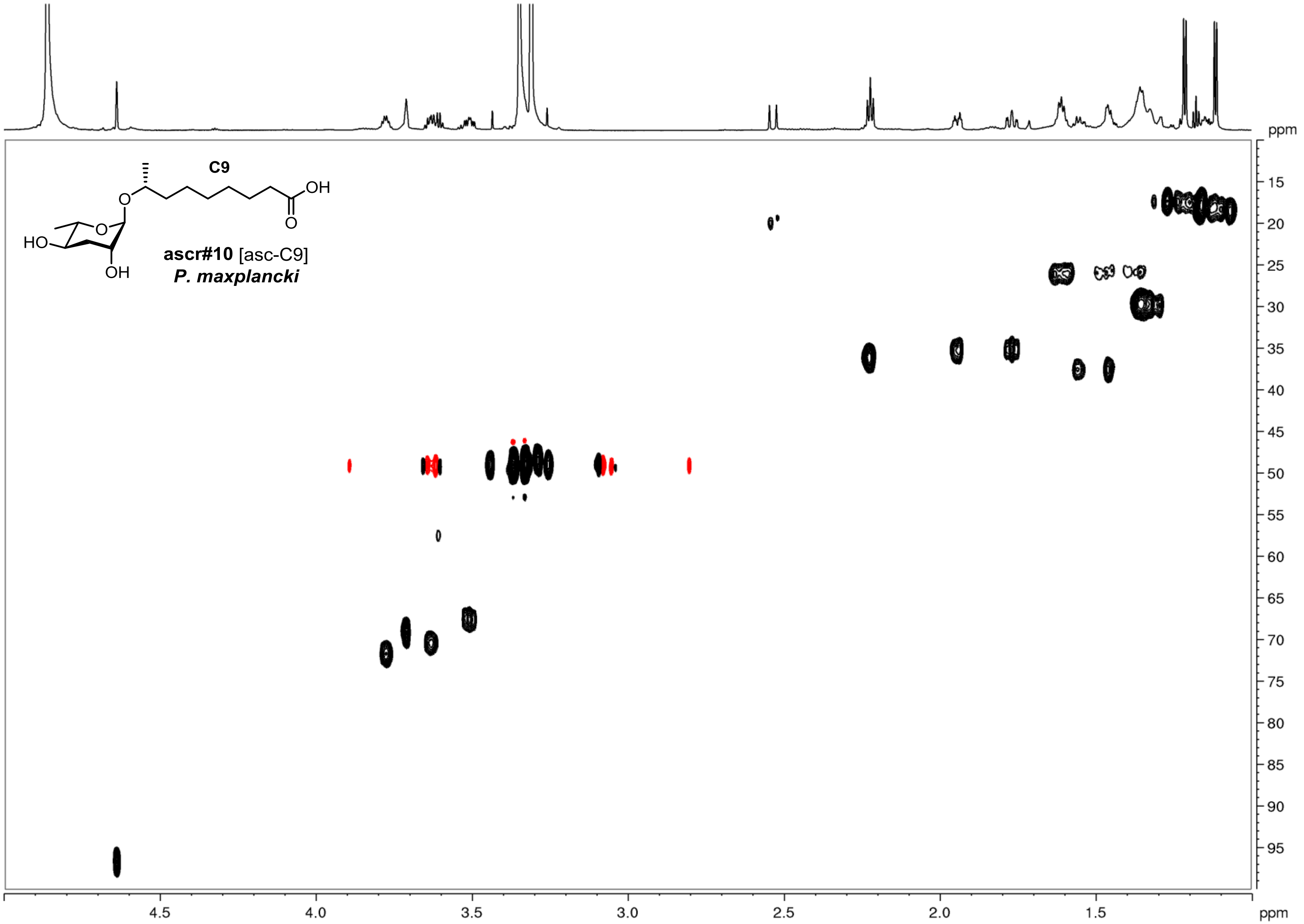

Figure 21. HSQC spectrum of ascr#10 [asc-C9] (800 MHz, CD_3OD) isolated from the *exo*-metabolome of *P. maxplancki*.

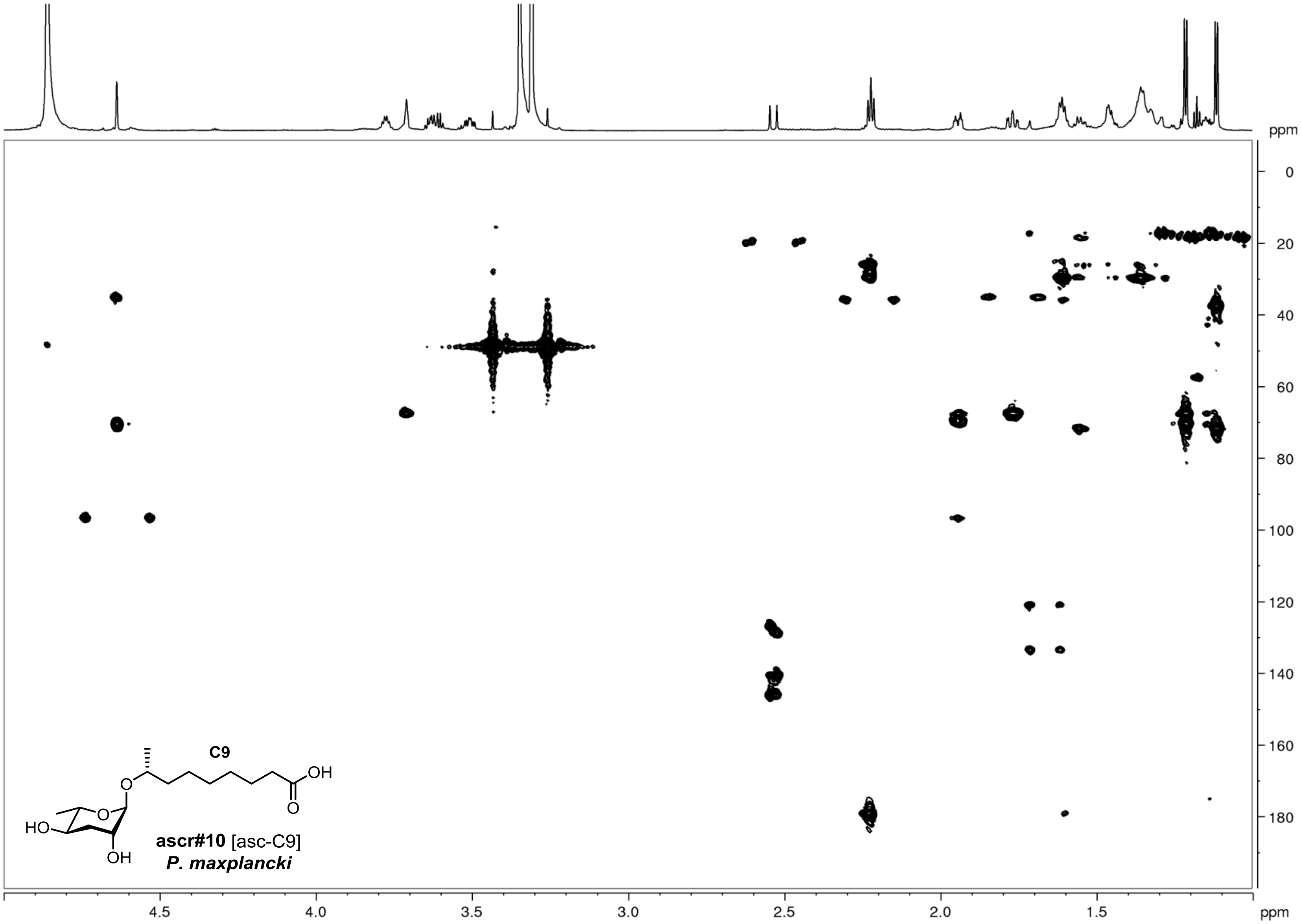

Figure 22. HMBC spectrum of ascr#10 [asc-C9] (800 MHz, CD_3OD) isolated from the *exo*-metabolome of *P. maxplancki*.

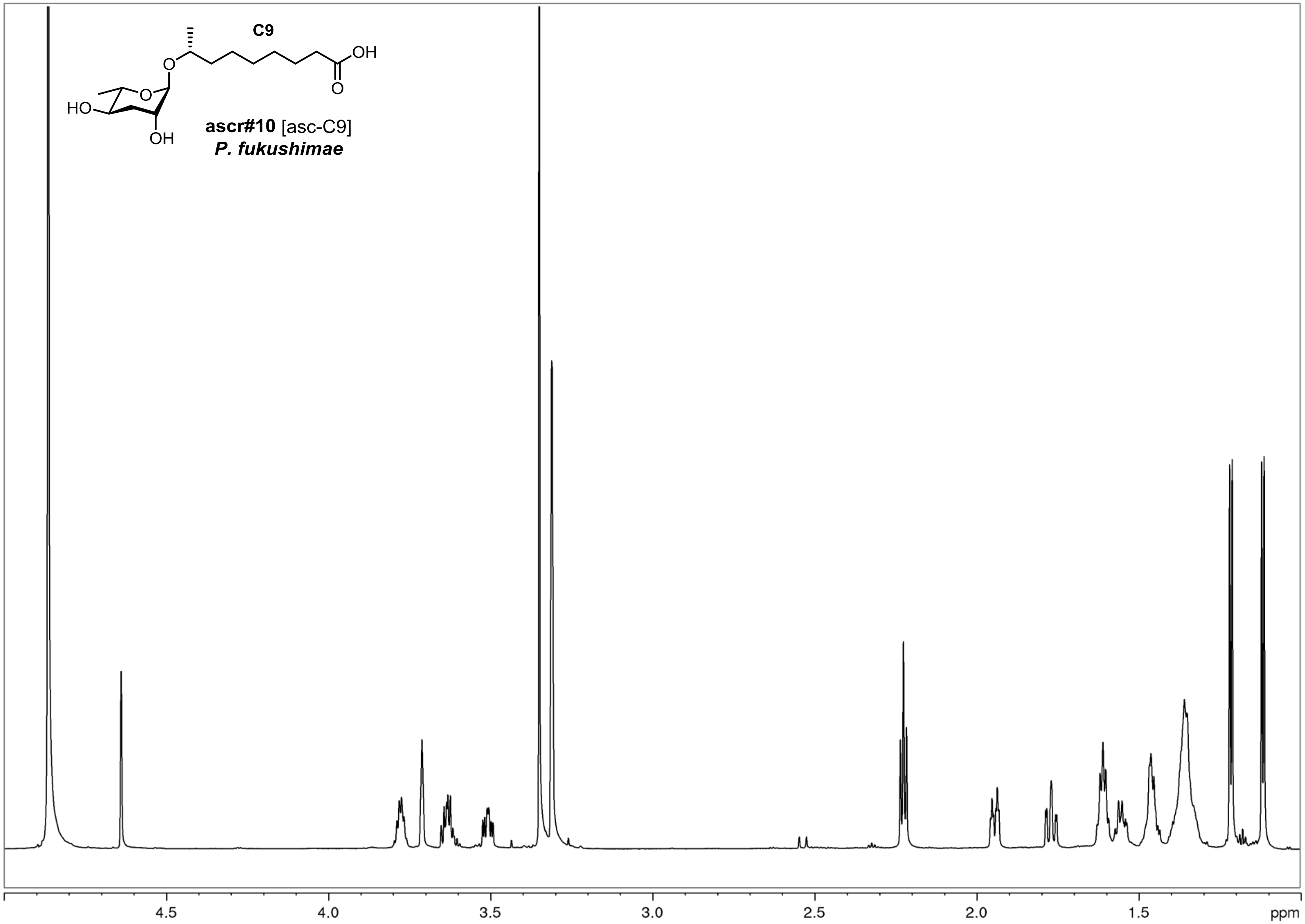

Figure 23. ^{1}H NMR spectrum of ascr#10 [asc-C9] (800 MHz, CD_3OD) isolated from the *exo*-metabolome of *P. fukushimae*.

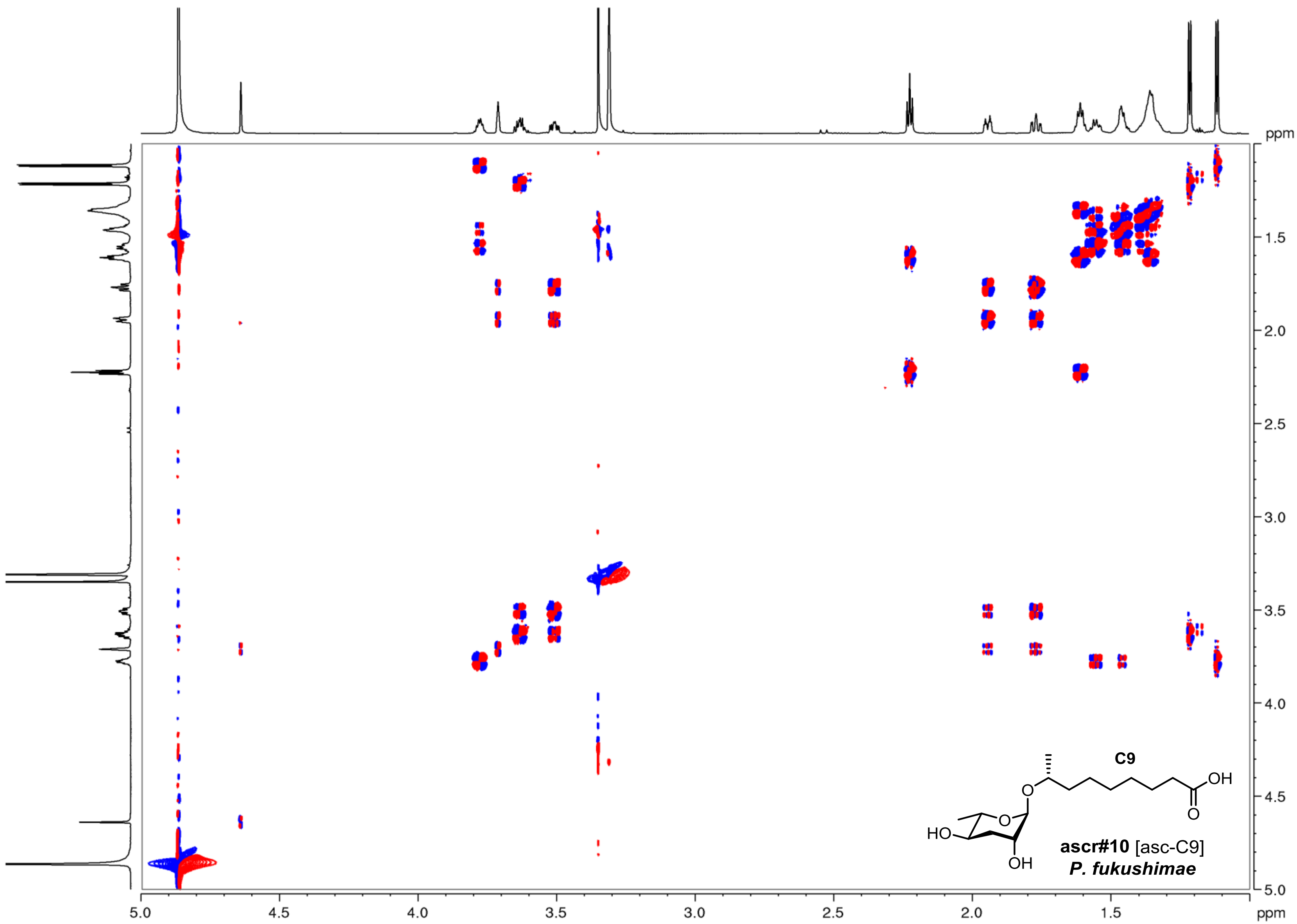

Figure 24. *dqf*-COSY spectrum of ascr#10 [asc-C9] (800 MHz, CD_3OD) isolated from the *exo*-metabolome of *P. fukushimae*.

supplementary file 1b: NMR spectra of DASC chemicals Pages

Figure 1. NMR spectra of HPLC enriched fraction containing dasc#3 (**1**) from *P. fukushimae*. S28

Figure 2-7. NMR spectra of dasc#4 (**2**) (800 MHz, CD_3OD) isolated from *P. taiwanensis*. S29

Figure 8-13. NMR spectra of dasc#4 (**2**) (800 MHz) isolated from *P. entomophagus*. S35

Figure 14-15. NMR spectra of dasc#4 (**2**) (800 MHz) isolated from *P. pacificus*. S41

Figure 16-20. NMR spectra of dasc#5 (**3**) (800 MHz, CD_3OD) isolated from *P. mayeri*. S43

Figure 21-26. NMR spectra of dasc#5 (**3**) (800 MHz, CD_3OD) isolated from *D. magnus*. S48

Figure 27. NMR spectra of dasc#6 (**4**) (800 MHz, CD_3OD) isolated from *P. entomophagus*. S54

Figure 28-31. NMR spectra of dasc#10 (**5**) (800 MHz, CD_3OD) isolated from *P. mayeri*. S55

Figure 32-39. NMR spectra of dasc#14 (**6**) (800 MHz) isolated from *P. entomophagus*. S59

Figure 40-46. NMR spectra of dasc#17 (**8**) (800 MHz) isolated from *P. uniformis*. S67

Figure 47. NMR spectra of an HPLC enriched mixture of dasc#16 (**7**) and dasc#17 (**8**). S74

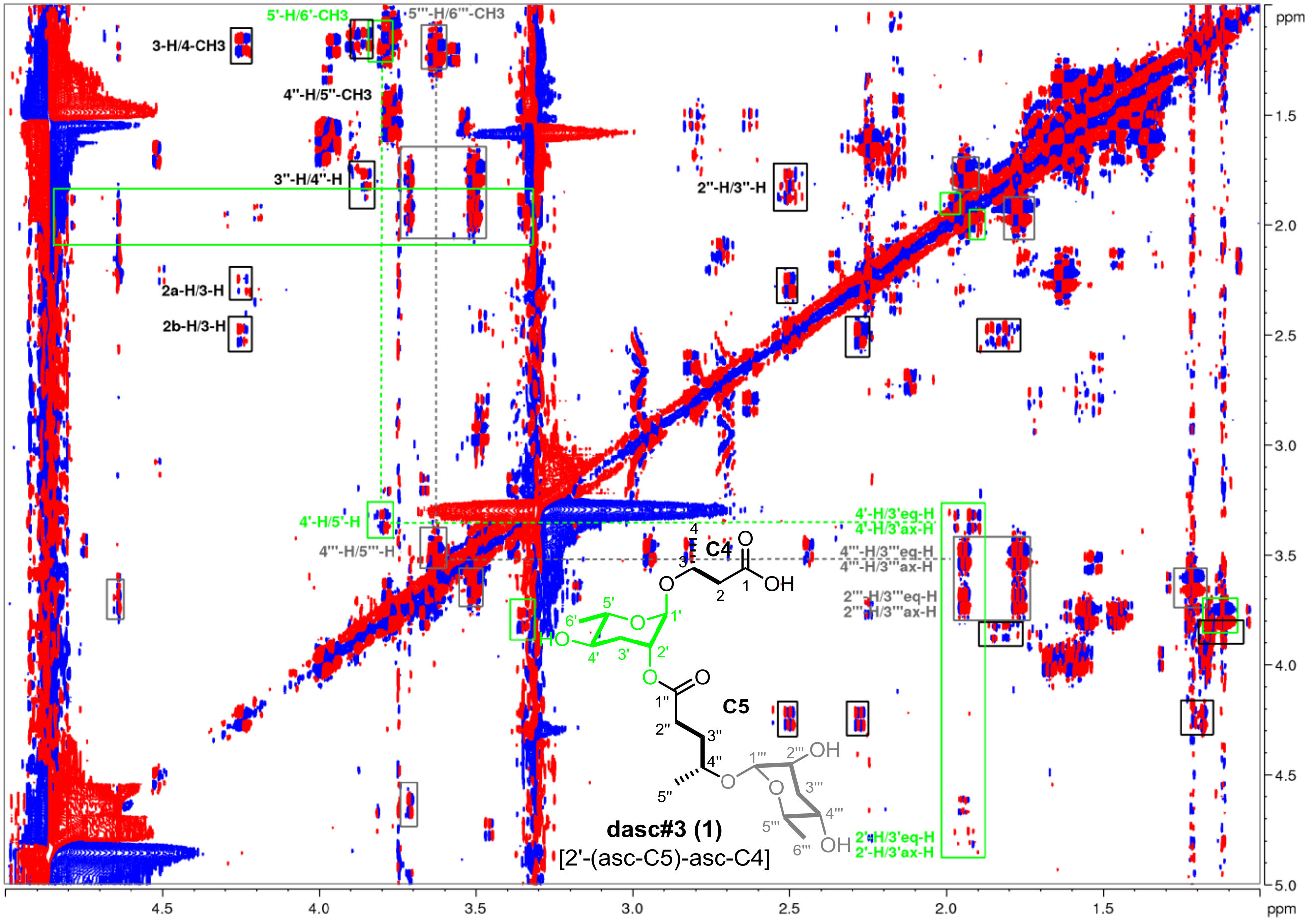

Figure 1. *dqf*-COSY spectrum of an enriched HPLC fraction containing dasc#3 [2'-(asc-C5)-asc-C4, **1**] (800 MHz, CD_3OD) isolated from the *exo*-metabolome of *P. fukushimae*. Characteristic ^1H, ^1H-COSY correlations of ascarylose (green boxed signals) from the second ascaroside ascr#11 [asc-C4] in dasc#3 [2'-(asc-C5)-asc-C4, **1**] showed that C-2' was substituted, given that the chemical shift of H-2' was shifted to the low field at 4.77 ppm, comparing to the chemical shift of H-2''' (3.71 ppm) at unsubstituted C-2''' (grey boxed signals). Two C4 and C5 fatty acid side chains from ascr#11 [asc-C4] and ascr#9 [asc-C5] were also observed (black boxed signals) in the spectrum. *dqf*-COSY spectroscopic data together with LC-MS/MS data (***supplementary file 2a – Figure 1***) suggests that dasc#3 [2'-(asc-C5)-asc-C4, **1**] is a dimeric ascaroside with ascr#9 [asc-C5] linked at the 2'-position of ascr#11 [asc-C4].

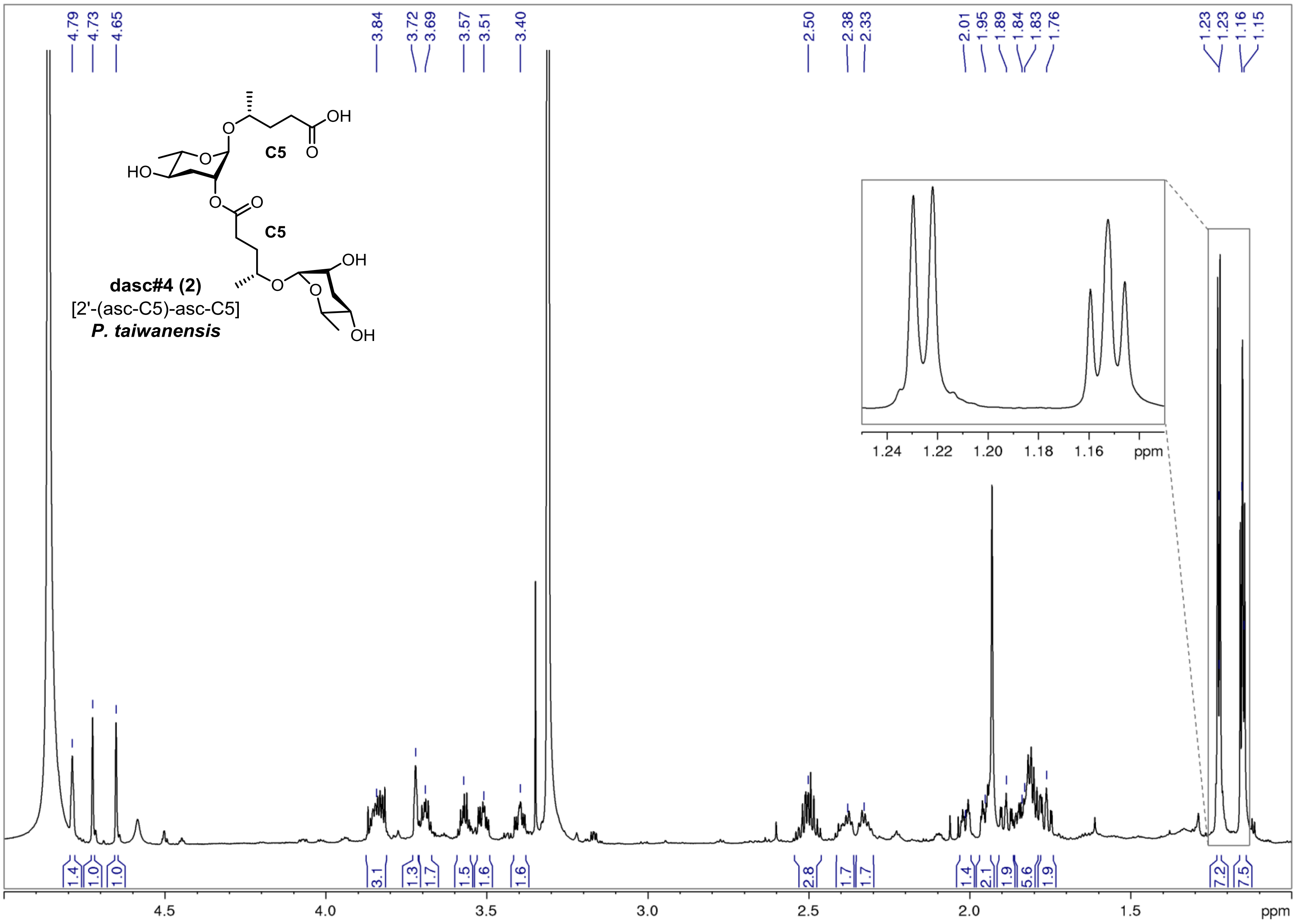

Figure 2. ^{1}H NMR spectrum of dasc#4 [2'-(asc-C5)-asc-C5, **2**] (800 MHz, CD_3OD) isolated from the *exo*-metabolome of *P. taiwanensis*.

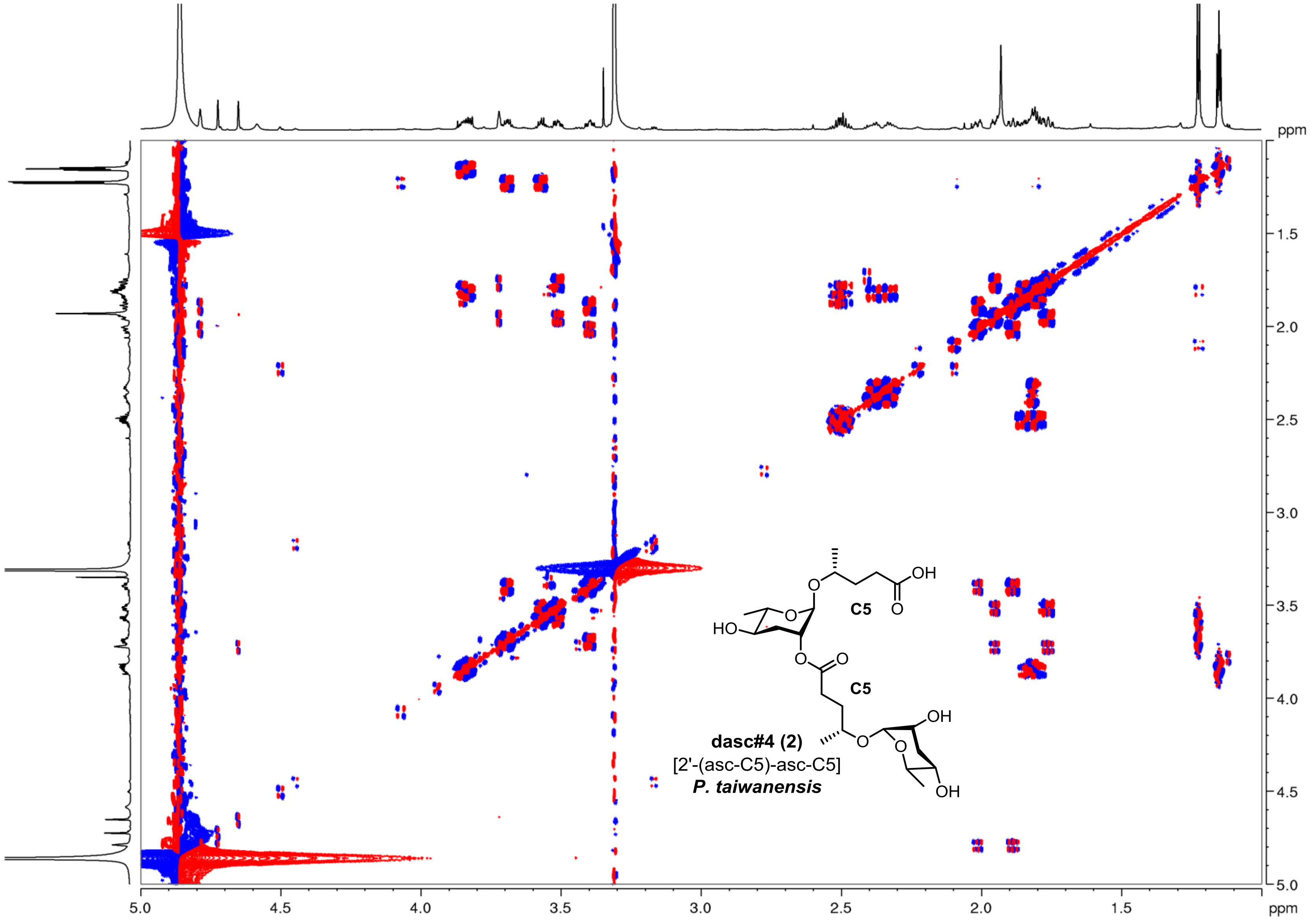

Figure 3. *dqf*-COSY spectrum of dasc#4 [2'-(asc-C5)-asc-C5, **2**] (800 MHz, CD_3OD) isolated from the *exo*-metabolome of *P. taiwanensis*.

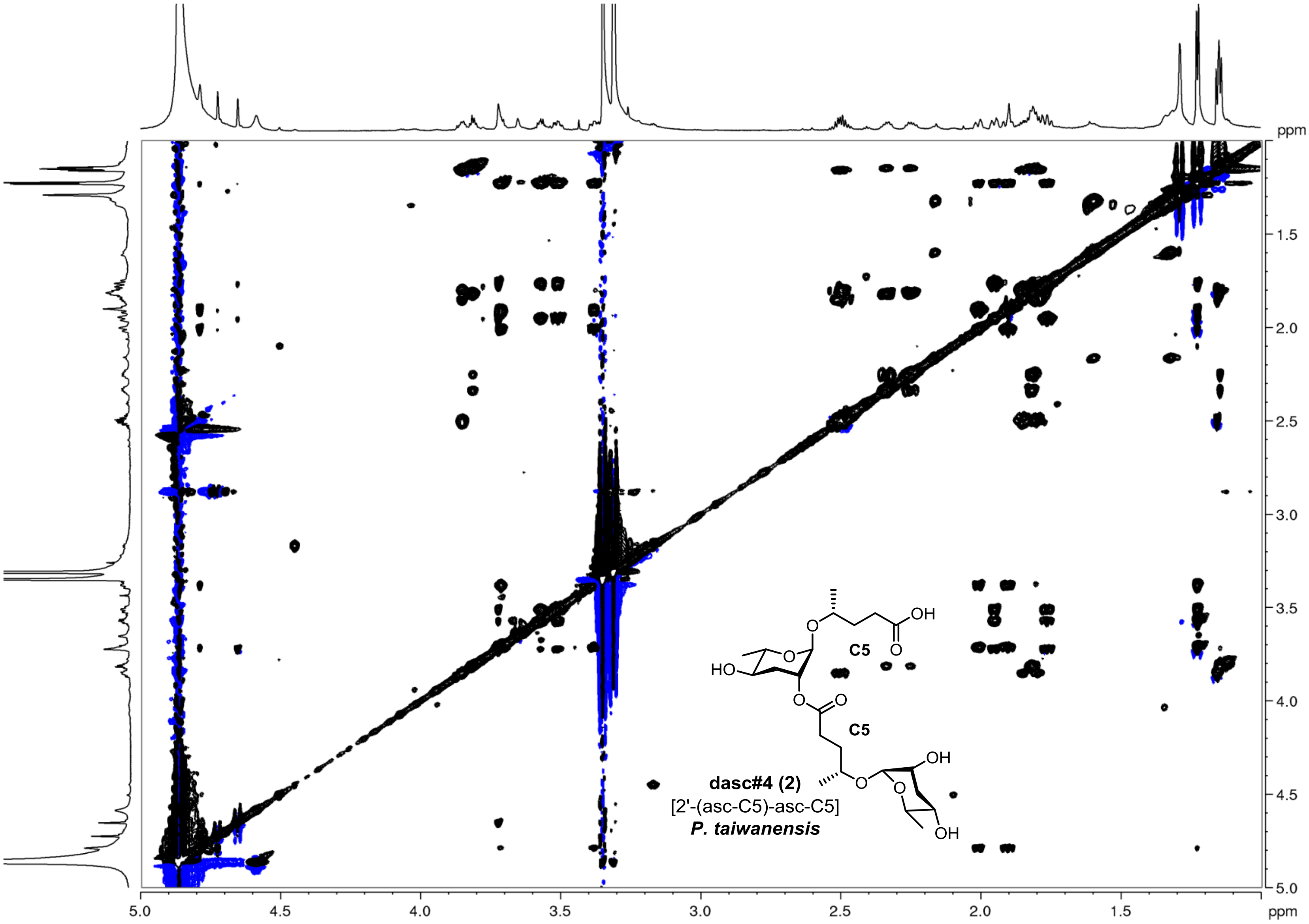

Figure 4. TOCSY spectrum of dasc#4 [2'-(asc-C5)-asc-C5, **2**] (800 MHz, CD_3OD) isolated from the *exo*-metabolome of *P. taiwanensis*.

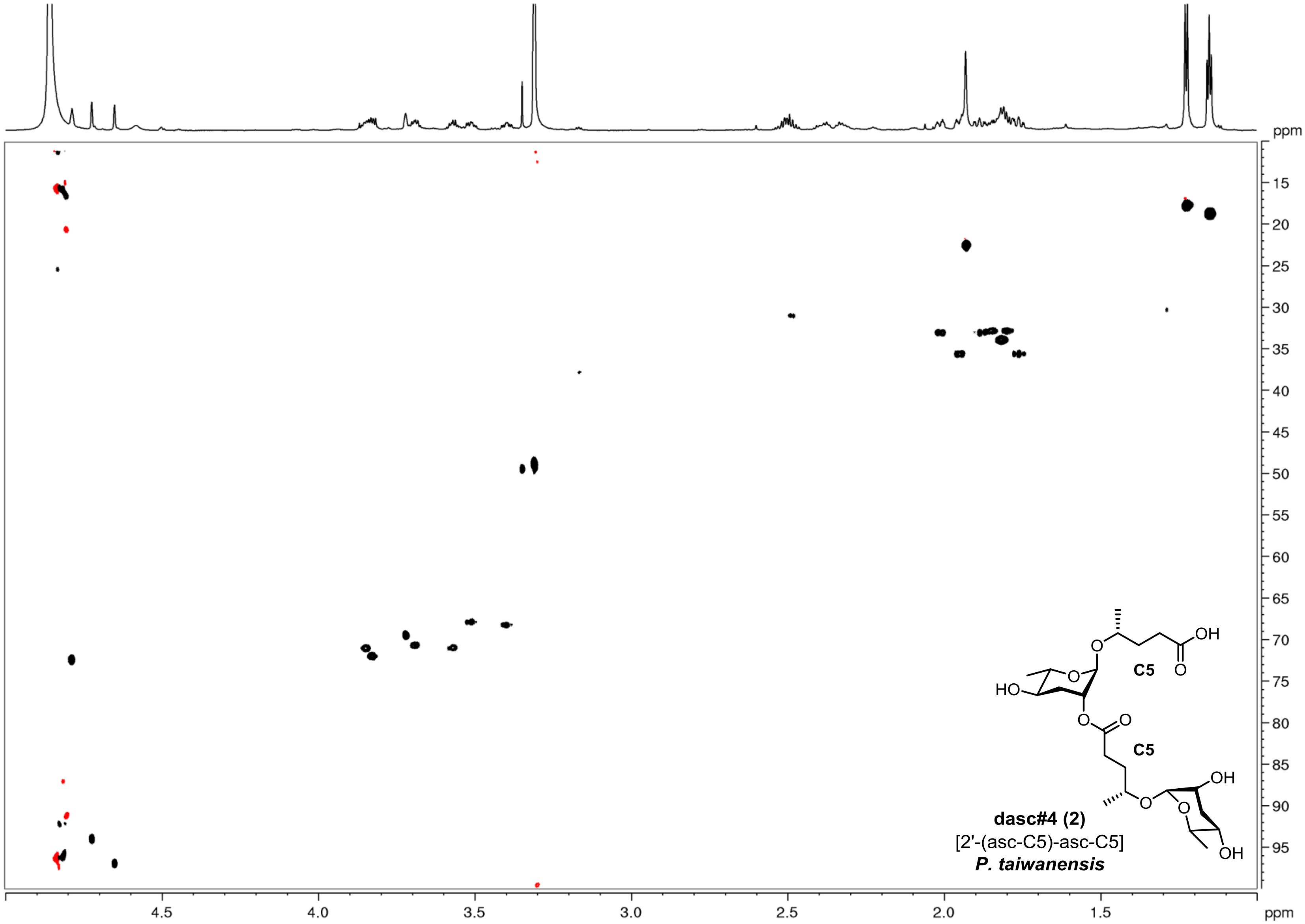

Figure 5. HSQC spectrum of dasc#4 [2'-(asc-C5)-asc-C5, **2**] (800 MHz, CD_3OD) isolated from the *exo*-metabolome of *P. taiwanensis*.

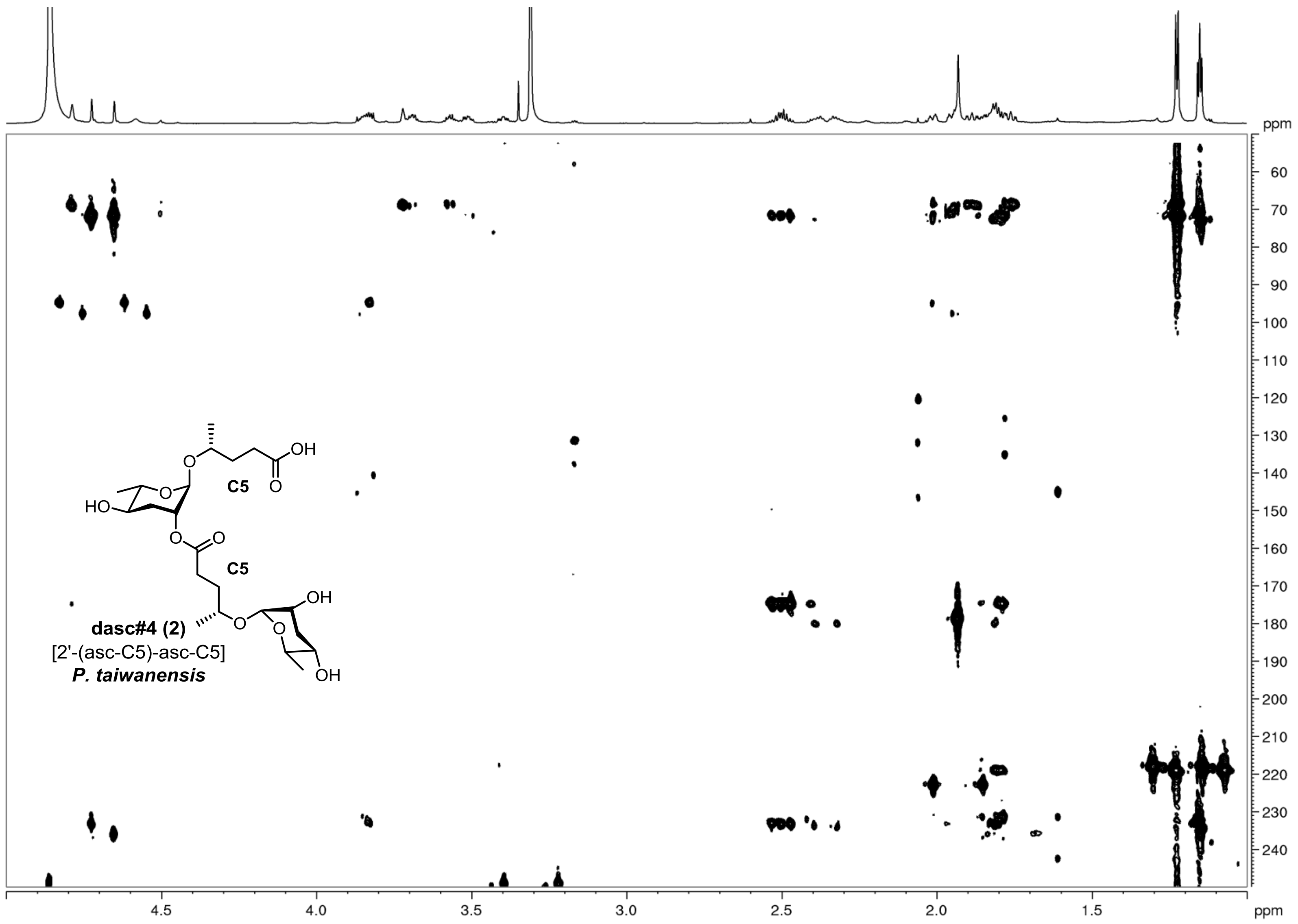

Figure 6. HMBC spectrum of dasc#4 [2'-(asc-C5)-asc-C5, **2**] (800 MHz, CD_3OD) isolated from the *exo*-metabolome of *P. taiwanensis* (SW = 199.5598).

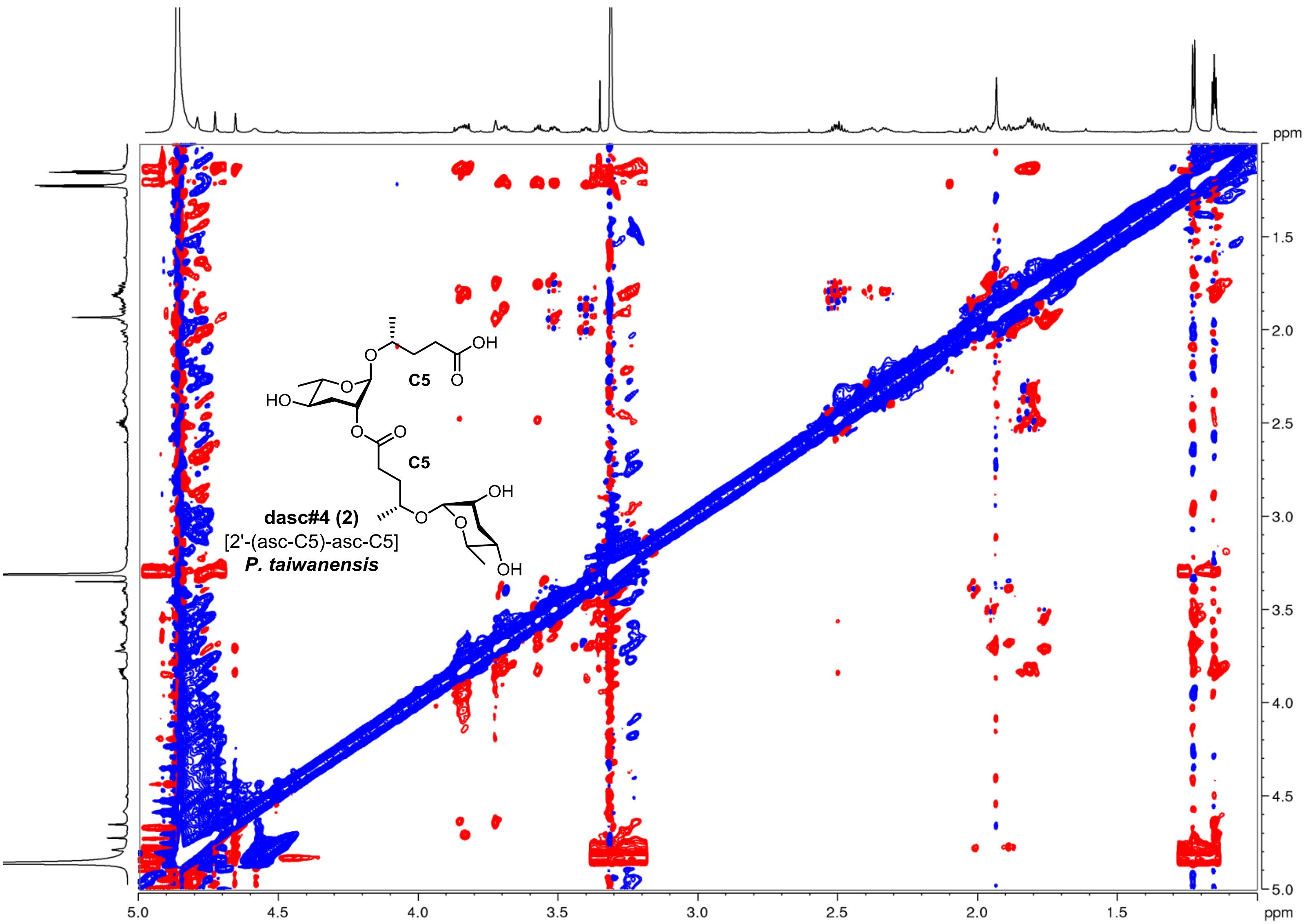

Figure 7. NOESY spectrum of dasc#4 [2'-(asc-C5)-asc-C5, **2**] (800 MHz, CD_3OD) isolated from the *exo*-metabolome of *P. taiwanensis*.

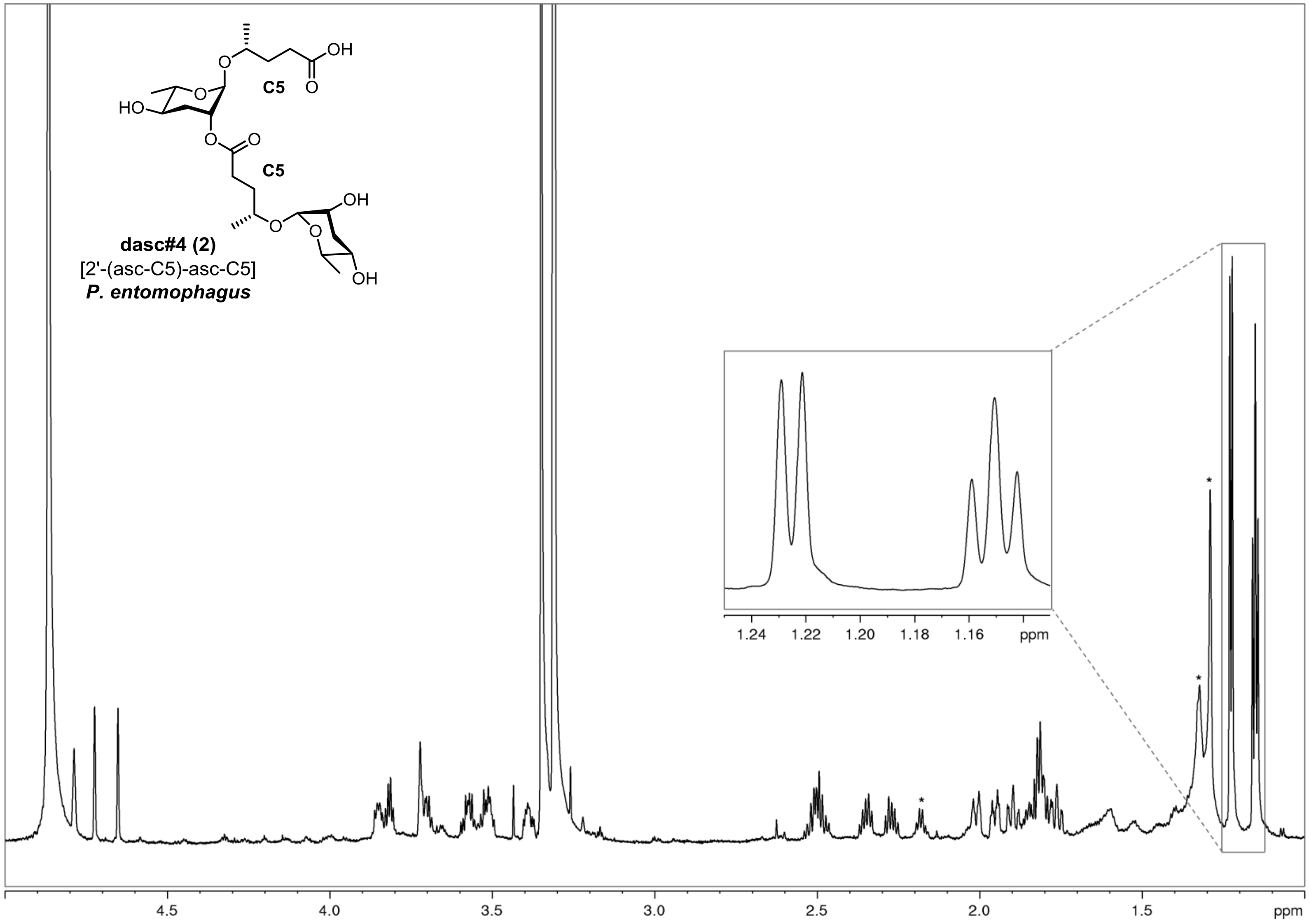

Figure 8. ^{1}H NMR spectrum of dasc#4 [2'-(asc-C5)-asc-C5, **2**] (800 MHz, CD_3OD) isolated from the *exo*-metabolome of *P. entomophagus*. Asterisks marked peaks are derived from impurities.

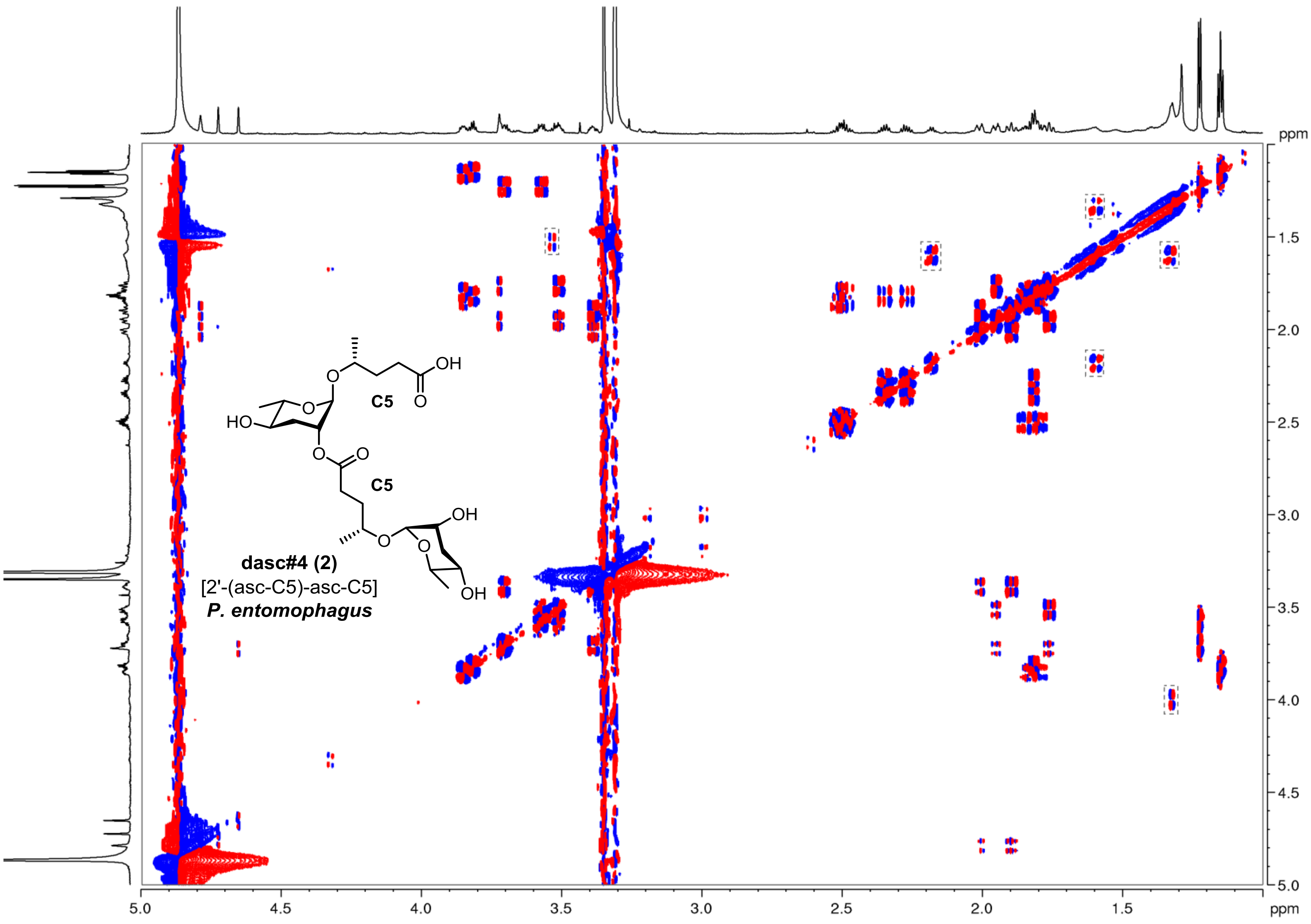

Figure 9. *dqf*-COSY spectrum of dasc#4 [2'-(asc-C5)-asc-C5, **2**] (800 MHz, CD_3OD) isolated from the *exo*-metabolome of *P. entomophagus*. Dashed line boxed signals are derived from impurities.

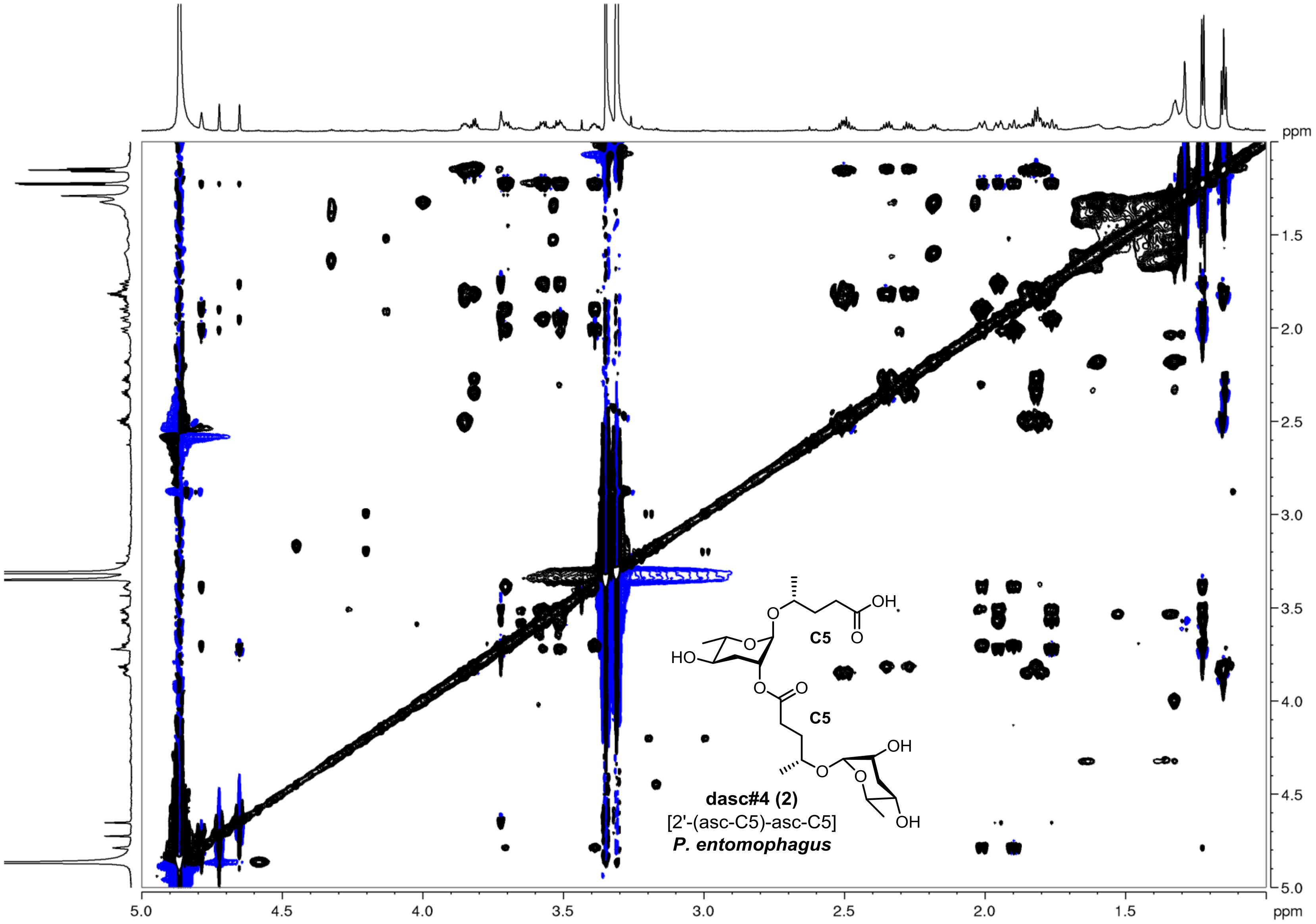

Figure 10. TOCSY spectrum of dasc#4 [2'-(asc-C5)-asc-C5, **2**] (800 MHz, CD_3OD) isolated from the *exo*-metabolome of *P. entomophagus*.

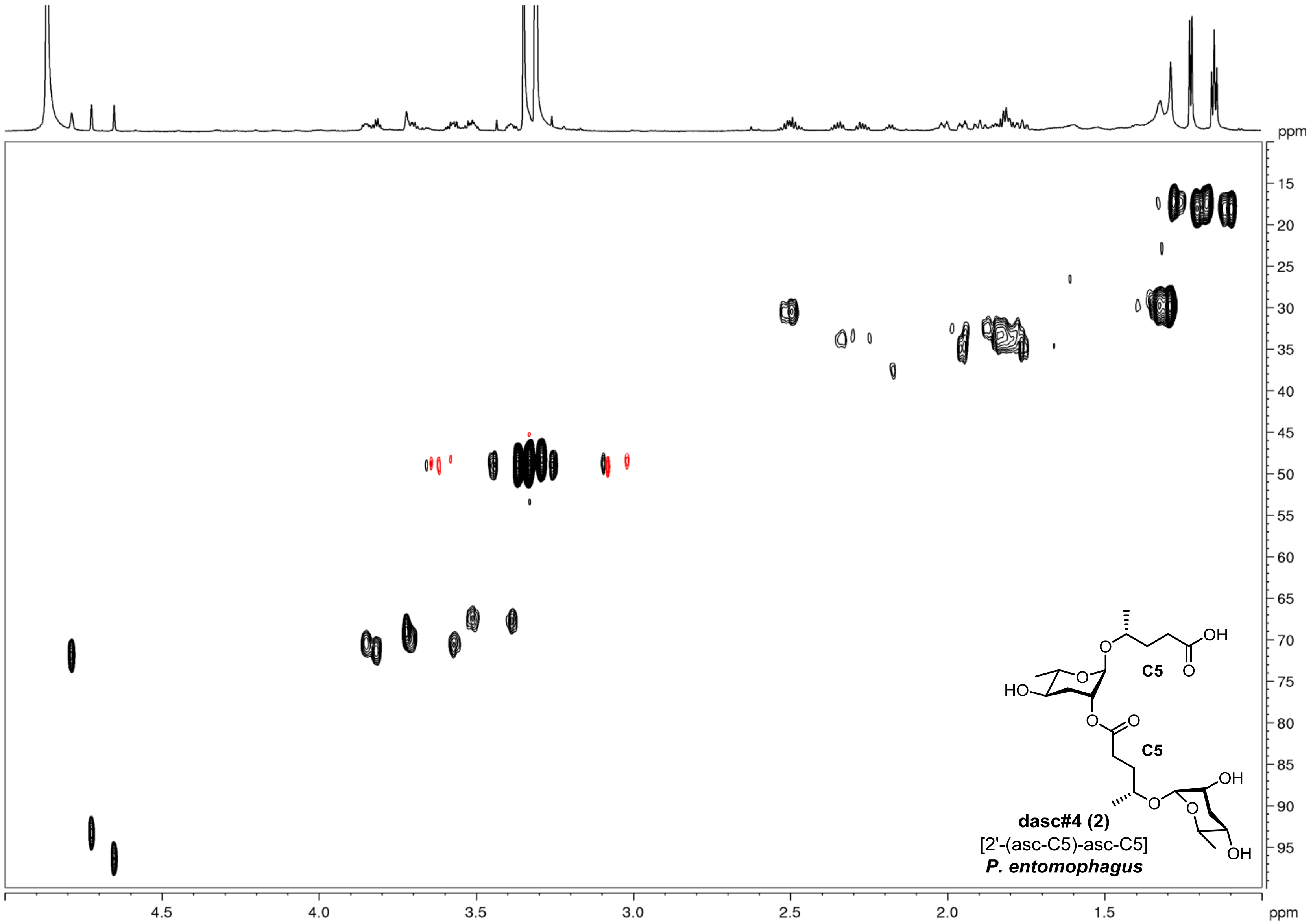

Figure 11. HSQC spectrum of dasc#4 [2'-(asc-C5)-asc-C5, **2**] (800 MHz, CD_3OD) isolated from the *exo*-metabolome of *P. entomophagus*.

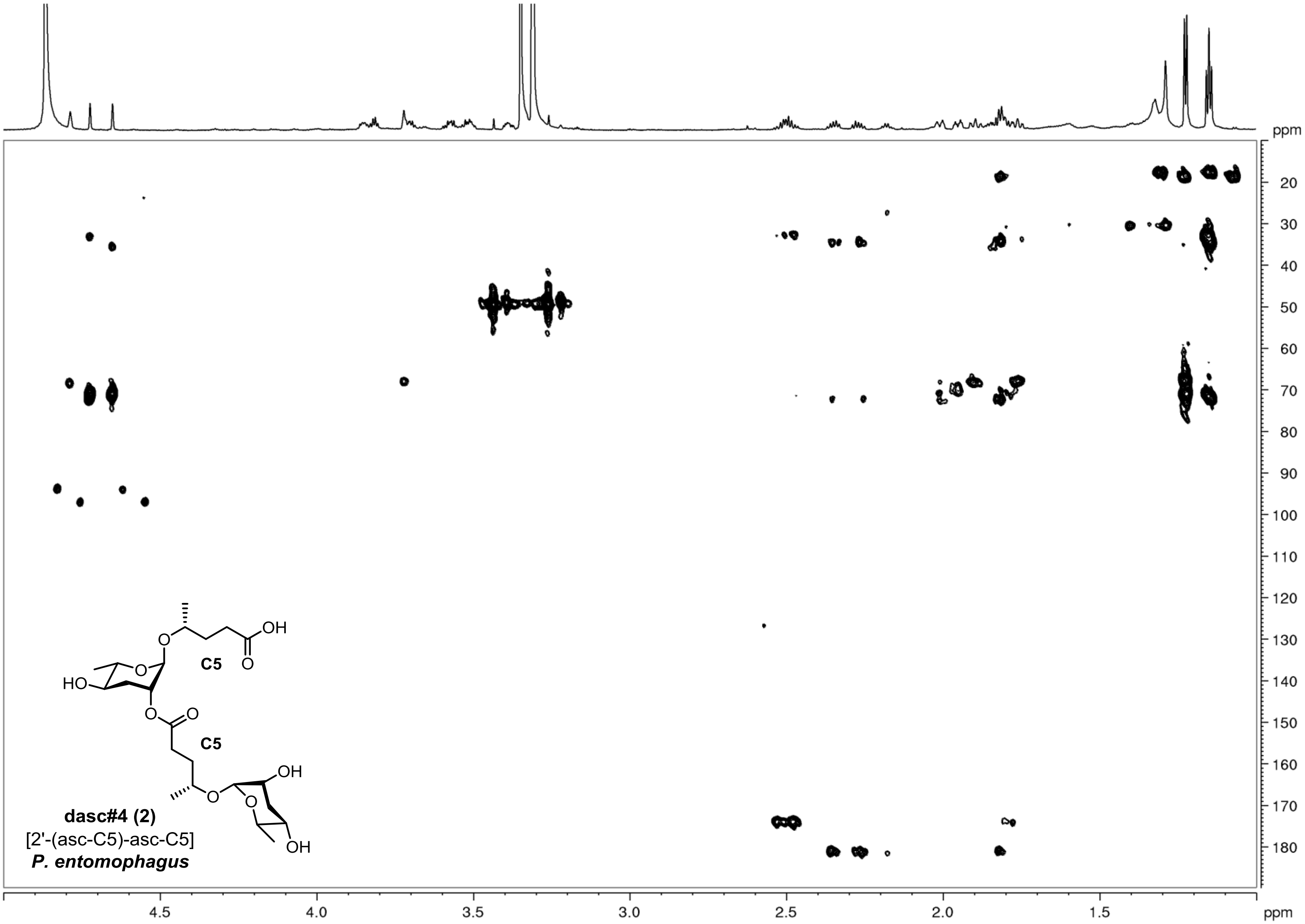

Figure 12. HMBC spectrum of dasc#4 [2'-(asc-C5)-asc-C5, **2**] (800 MHz, CD_3OD) isolated from the *exo*-metabolome of *P*. *entomophagus*.

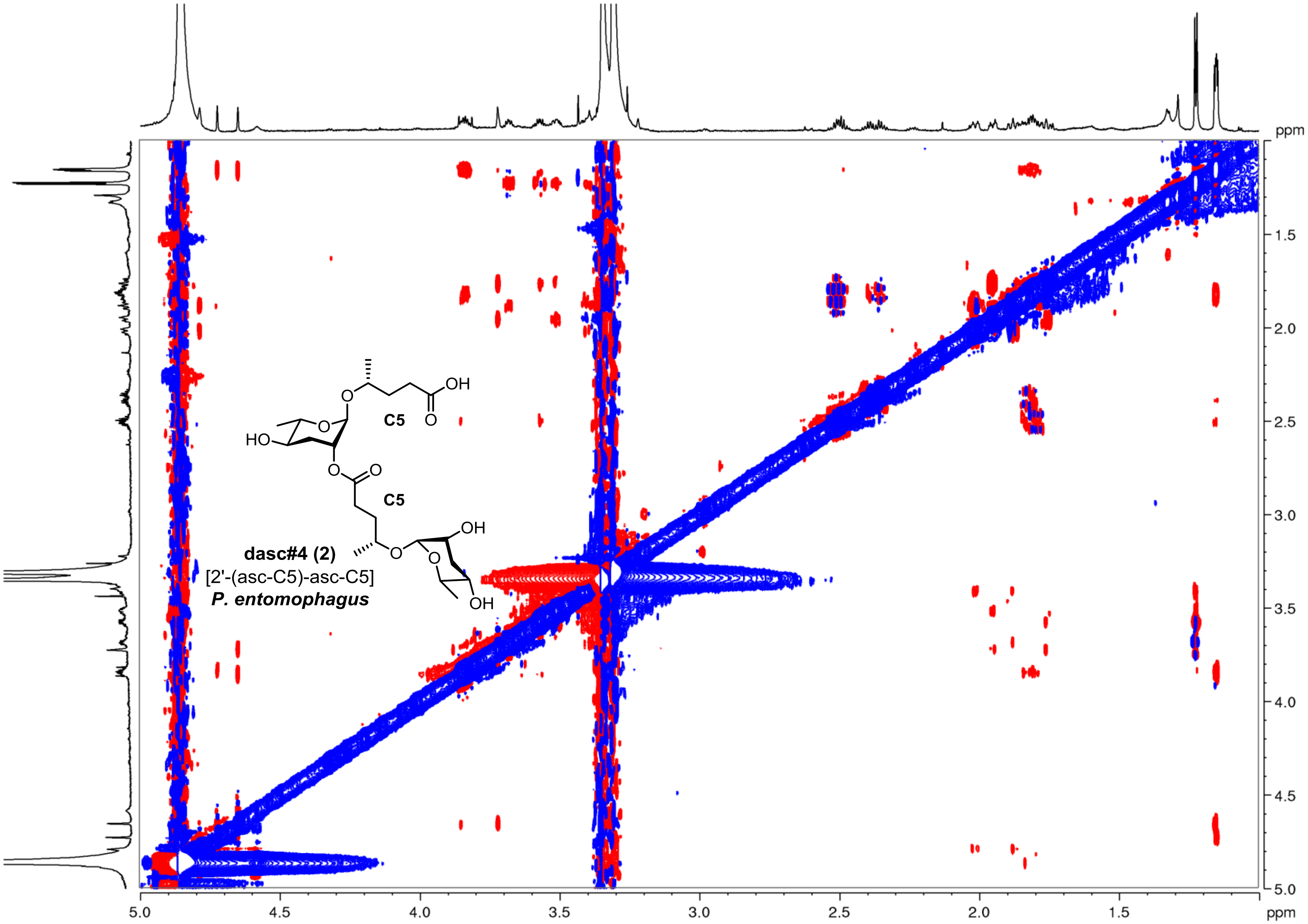

Figure 13. NOESY spectrum of dasc#4 [2'-(asc-C5)-asc-C5, **2**] (800 MHz, CD_3OD) isolated from the *exo*-metabolome of *P. entomophagus*.

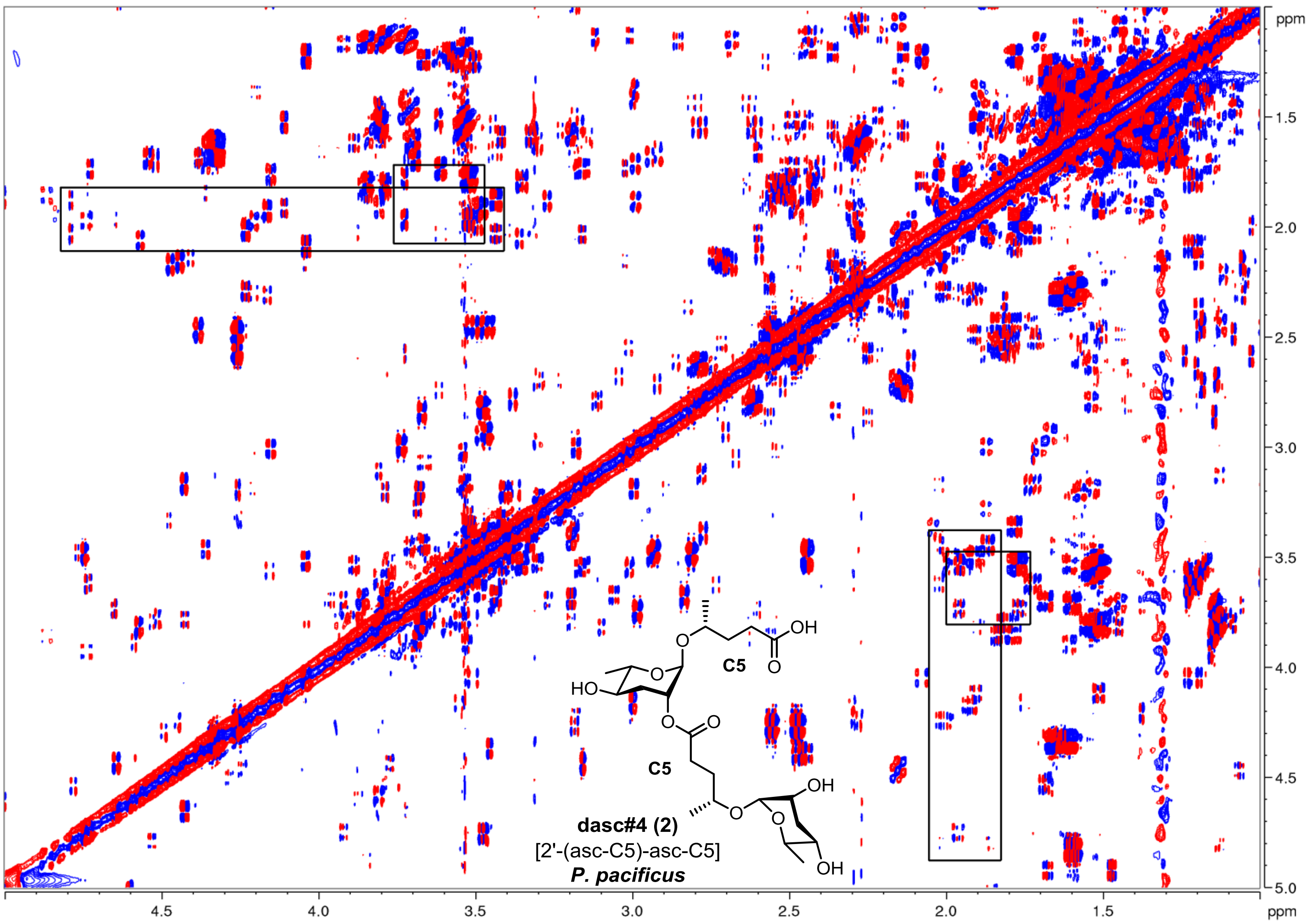

Figure 14. *dqf*-COSY spectrum of an isolated SPE sample containing dasc#4 [2'-(asc-C5)-asc-C5, **2**] (in CD_3OD) derived from the inital fractionation of a 2 l liquid culture of an inbred strain, which was generated by crossing the *P. pacificus* strains RSA016 and RSC011.

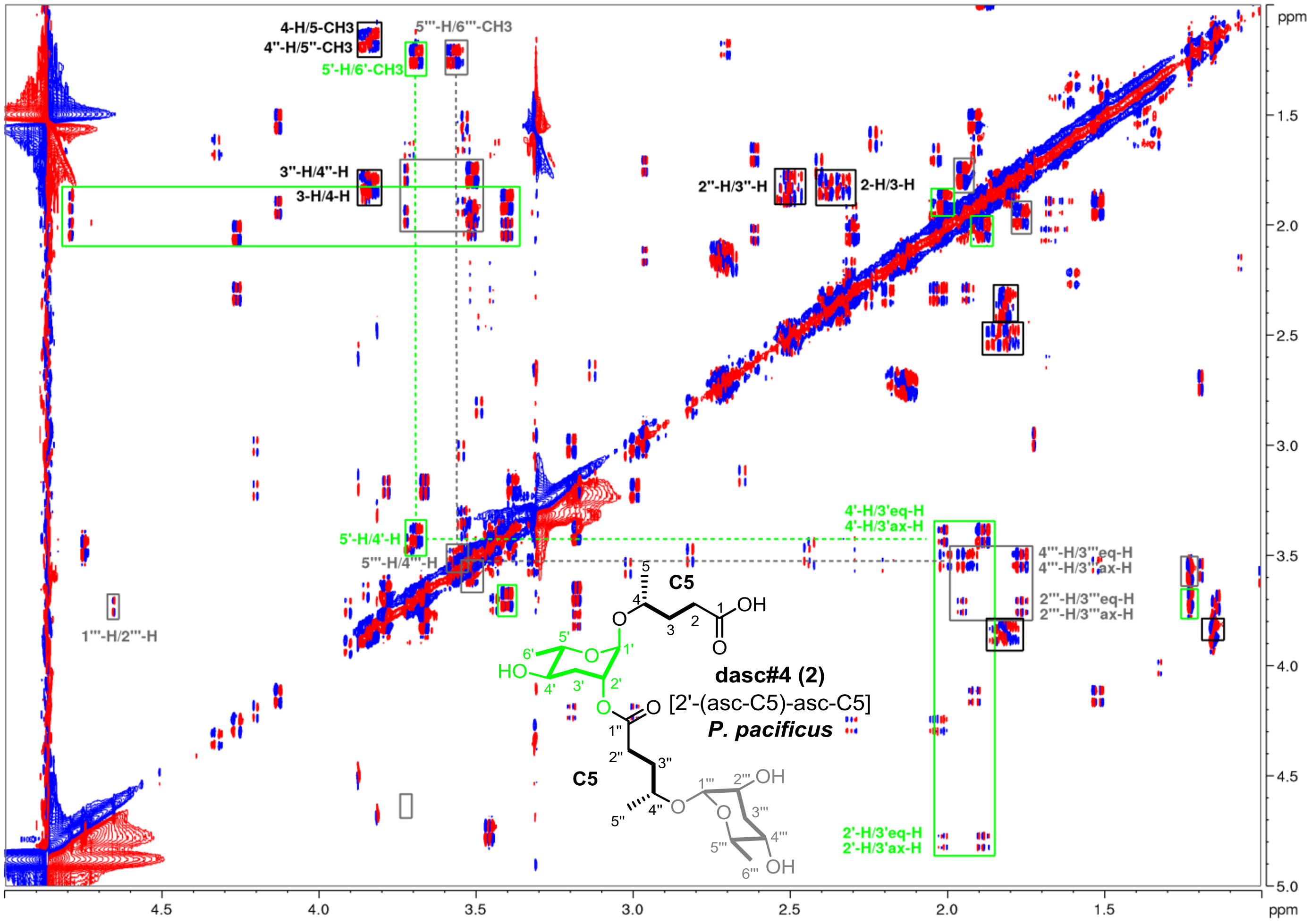

Figure 15. *dqf*-COSY spectrum of an HPLC enriched sample containing dasc#4 [2'-(asc-C5)-asc-C5, **2**] (in CD_3OD) isolated from the *exo*-metabolome of an inbred strain derived from crossing the *P. pacificus* strains RSA016 and RSC011. Key ^{1}H, ^{1}H-COSY correlation signals (black boxed signals) of two (ω-1) style C5 fatty acid side chains and two characteristic ascarylose sugars are observed in the *dqf*-COSY spectrum. One ascarylose sugar is modified at the 2'-position (green boxed signals) due to the chemical shift of H-2' at 4.79 ppm and another one is free of any modification (grey boxed signals), which are completely identical to the *dqf*-COSY data of dasc#4 [2'-(asc-C5)-asc-C5, **2**] isolated from *P. taiwanensis* (***supplementary file 1b – Figure 3***) and *P. entomophagus* (***supplementary file 1b – Figure 9***).

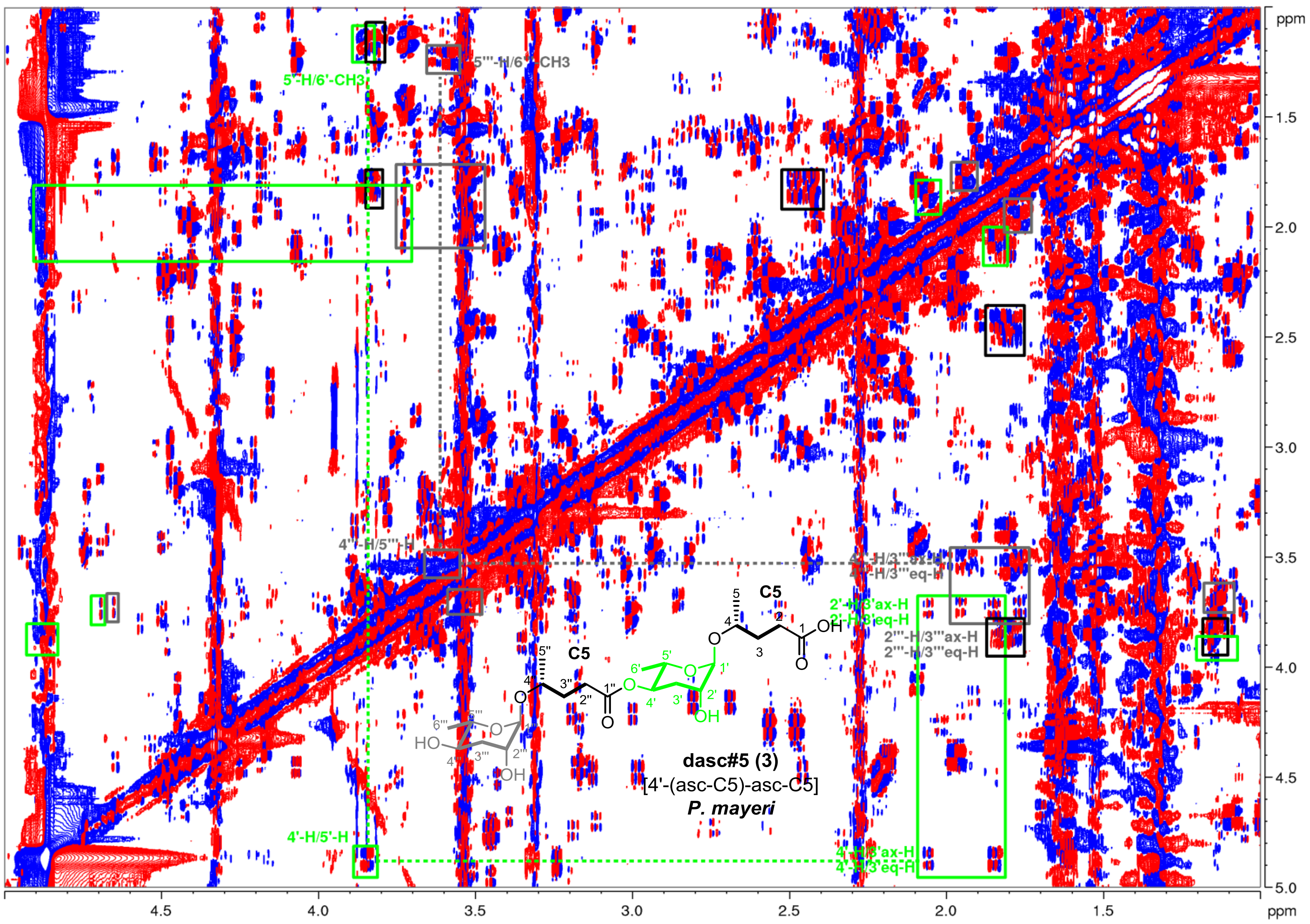

Figure 16. *dqf*-COSY spectrum of an SPE enriched sample containing dasc#5 [4'-(asc-C5)-asc-C5, **3**] (800 MHz, CD_3OD) isolated from *P. mayeri exo*-metabolome.

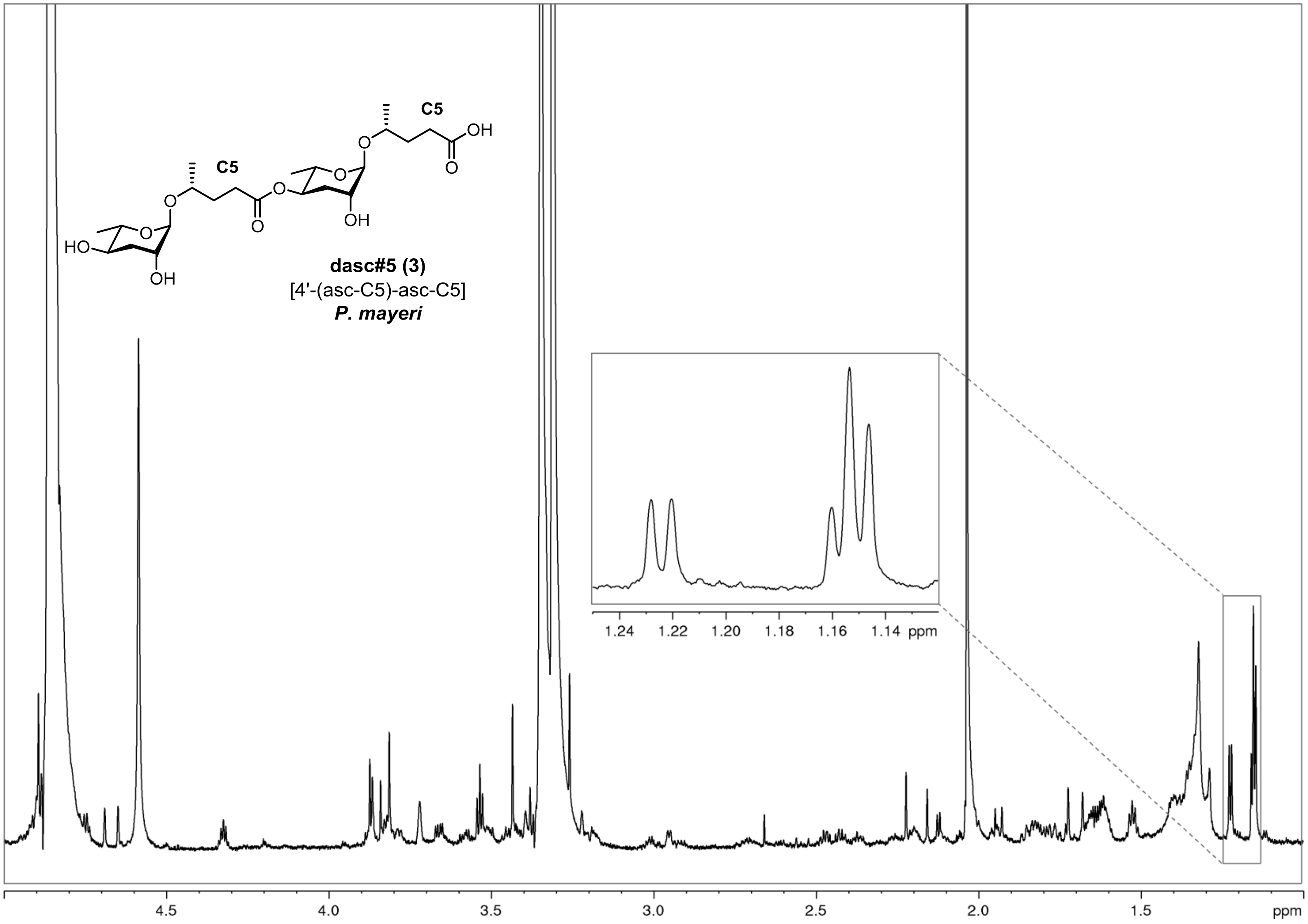

Figure 17. ^{1}H NMR spectrum of an HPLC enriched sample containing dasc#5 [4'-(asc-C5)-asc-C5, **3**] (800 MHz, CD_3OD) isolated from *P. mayeri exo*-metabolome.

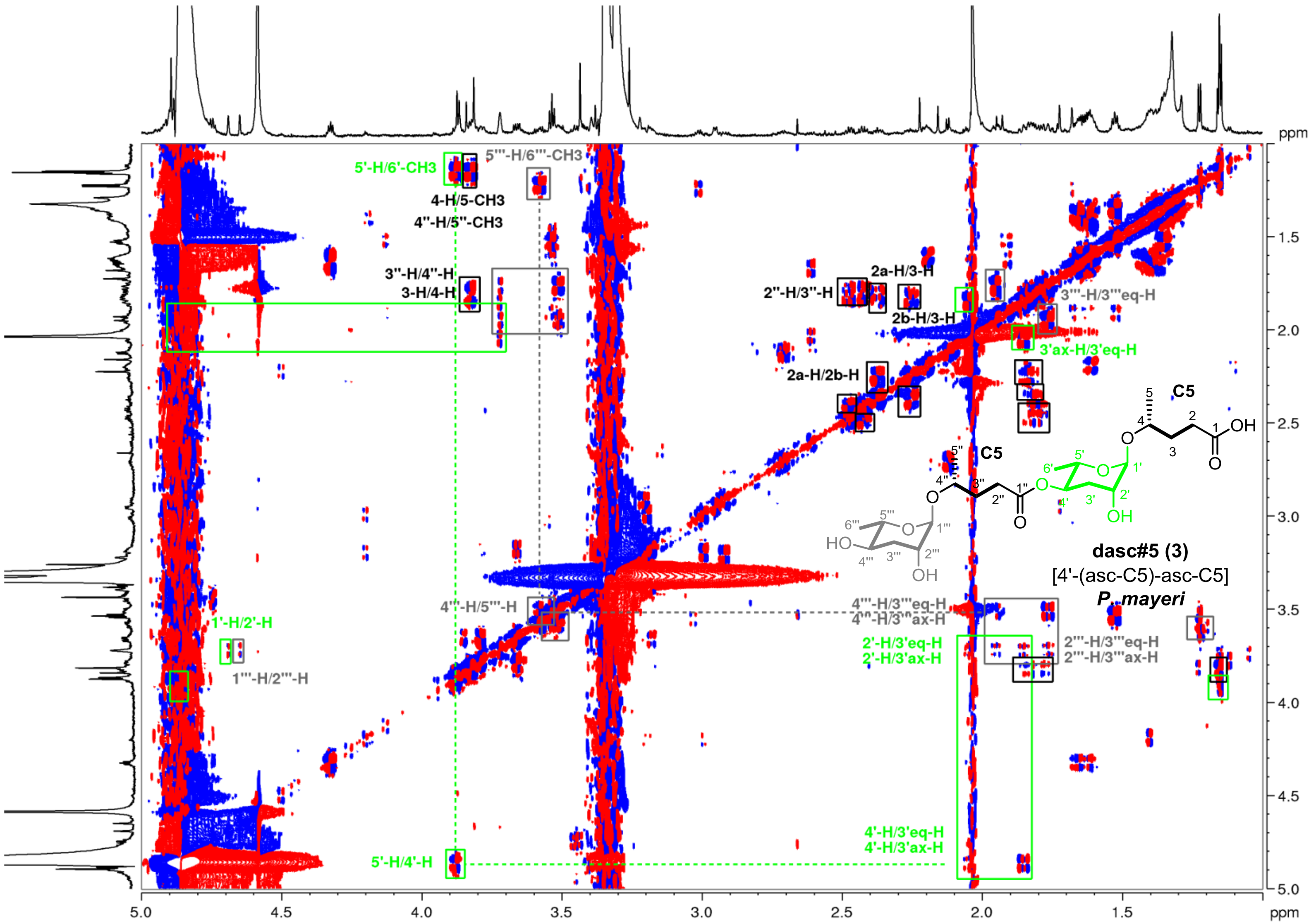

Figure 18. *dqf*-COSY spectrum of an HPLC enriched sample containing dasc#5 [4'-(asc-C5)-asc-C5, **3**] (800 MHz, CD_3OD) isolated from the *exo*-metabolome of *P. mayeri*.

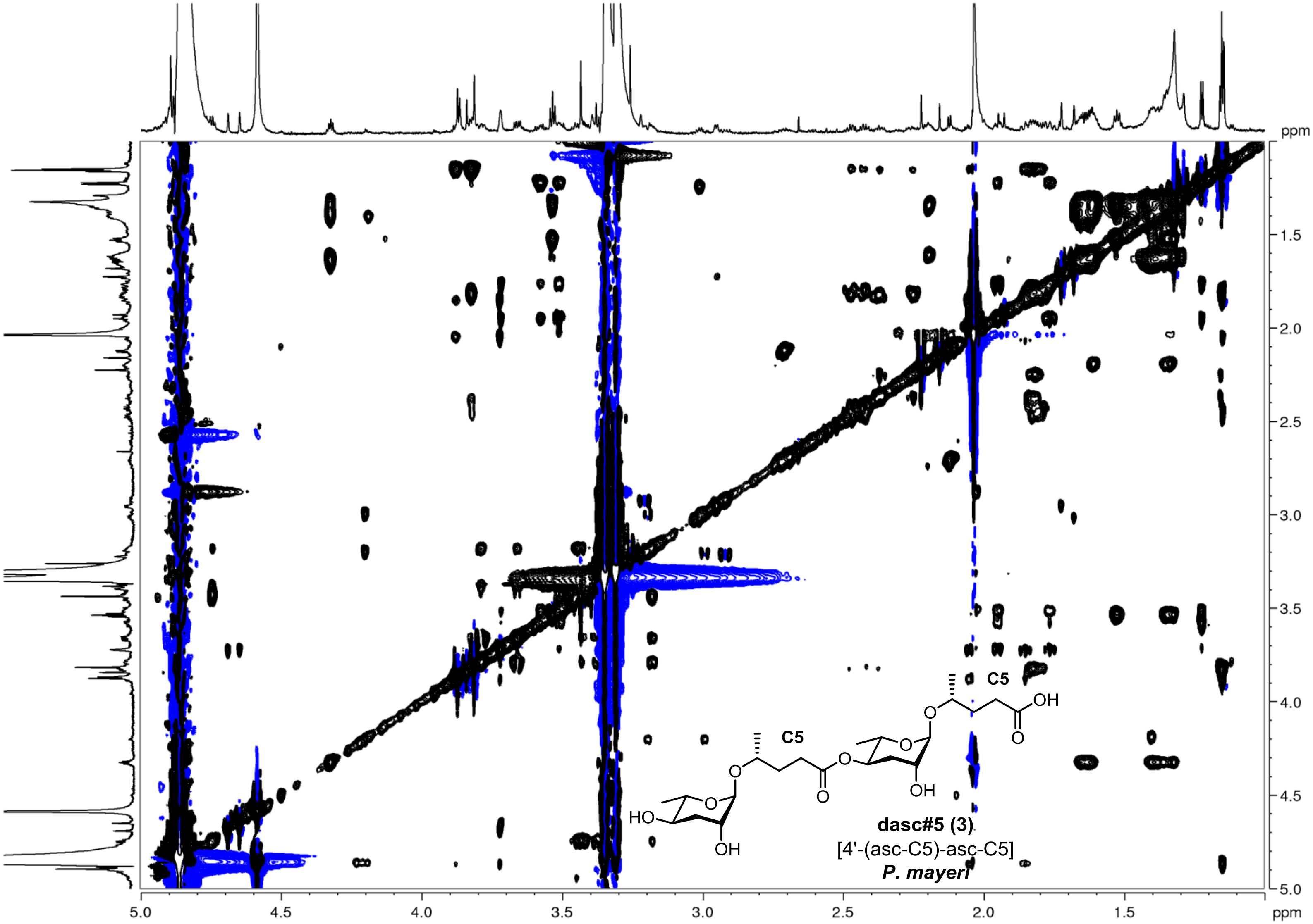

Figure 19. TOCSY spectrum of an HPLC enriched sample containing dasc#5 [4'-(asc-C5)-asc-C5, **3**] (800 MHz, CD_3OD) isolated from the *exo*-metabolome of *P. mayeri*.

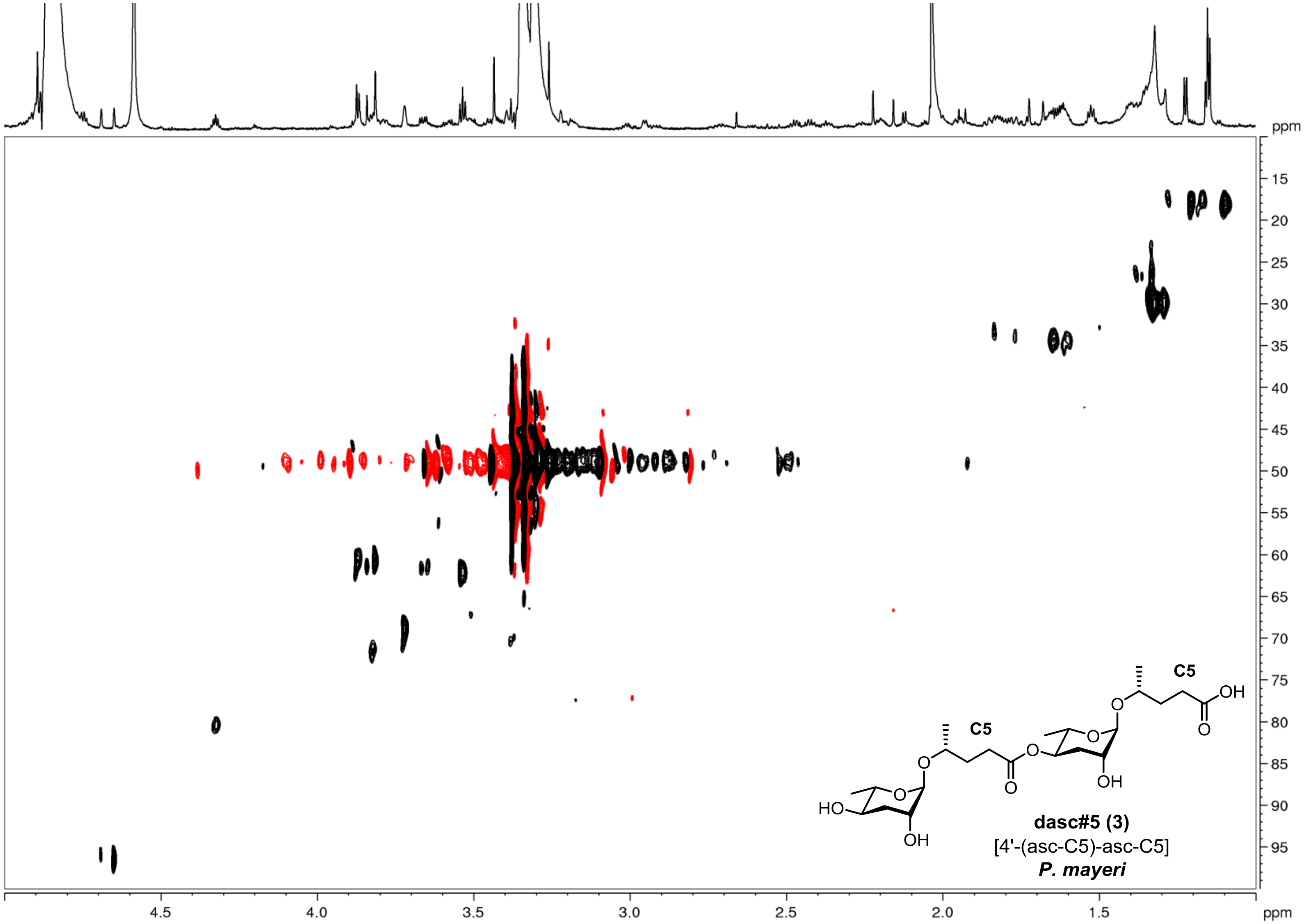

Figure 20. HSQC spectrum of an HPLC enriched sample containing dasc#5 [4'-(asc-C5)-asc-C5, **3**] (800 MHz, CD_3OD) isolated from the *exo*-metabolome of *P. mayeri*.

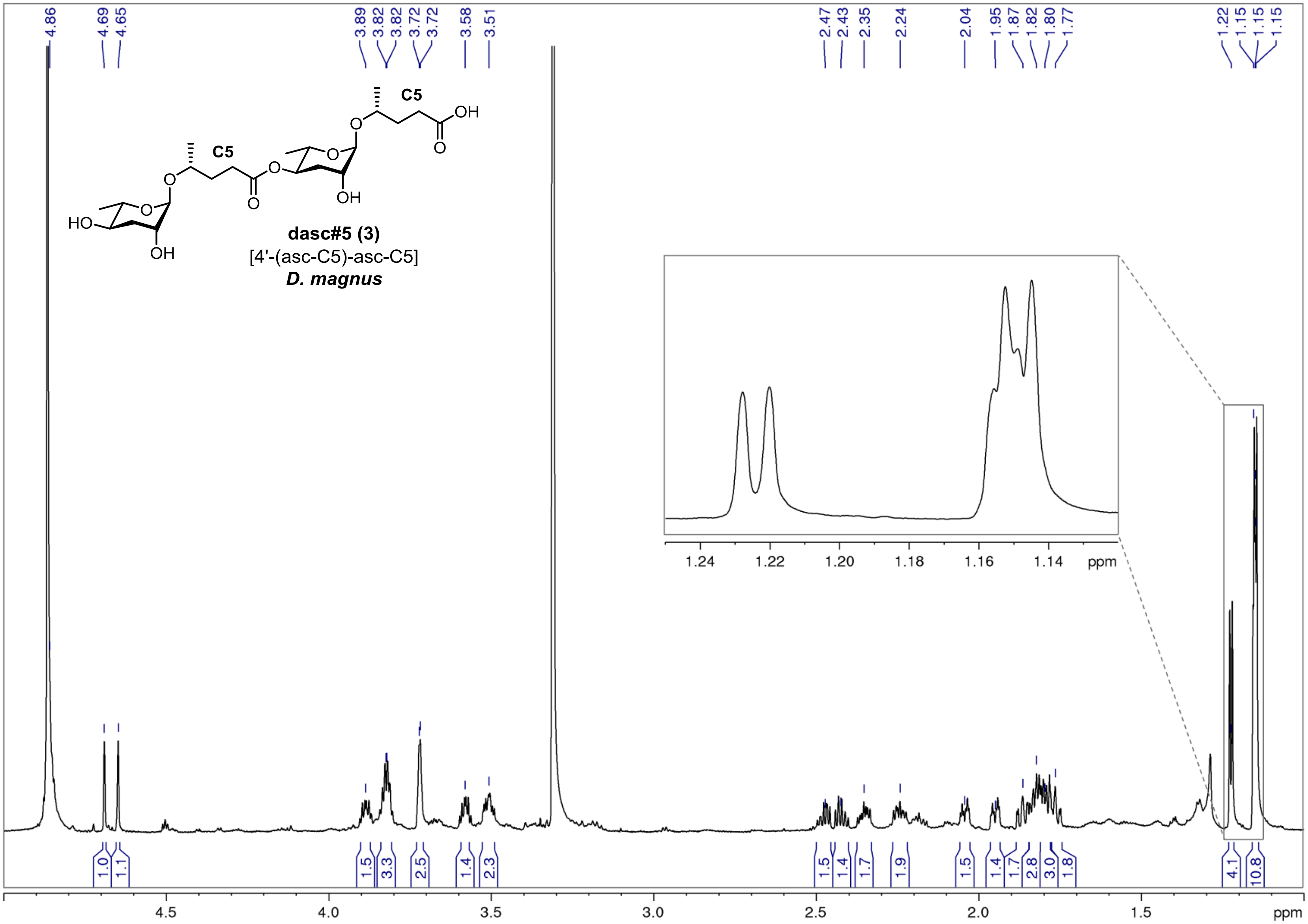

Figure 21. ^{1}H NMR spectrum of dasc#5 [4'-(asc-C5)-asc-C5, **3**] (800 MHz, CD_3OD) isolated from the *exo*-metabolome of *D. magnus*.

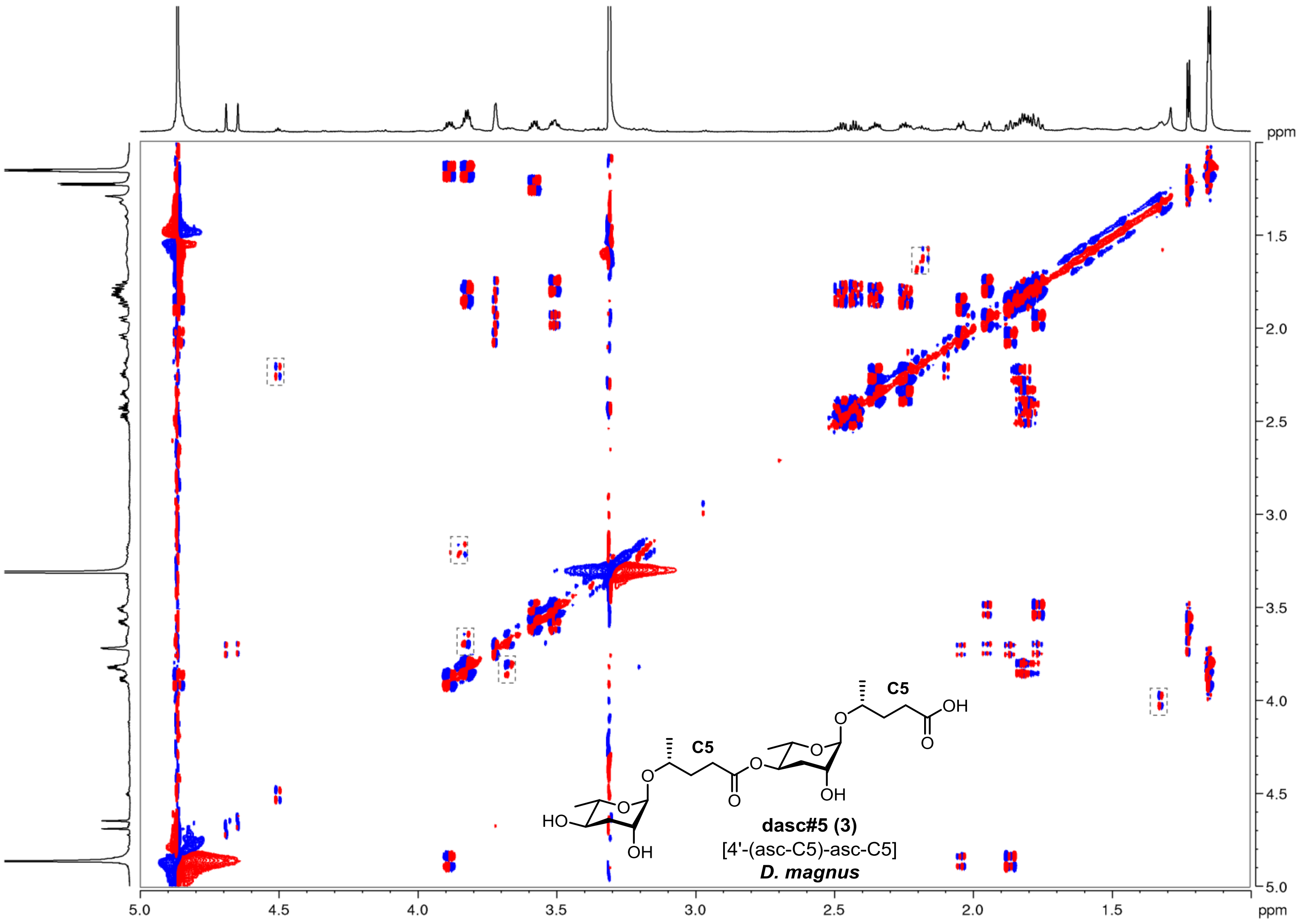

Figure 22. *dqf*-COSY spectrum of dasc#5 [4'-(asc-C5)-asc-C5, **3**] (800 MHz, CD_3OD) isolated from the *exo*-metabolome of *D. magnus*. Dashed line boxed signals are derived from imputities.

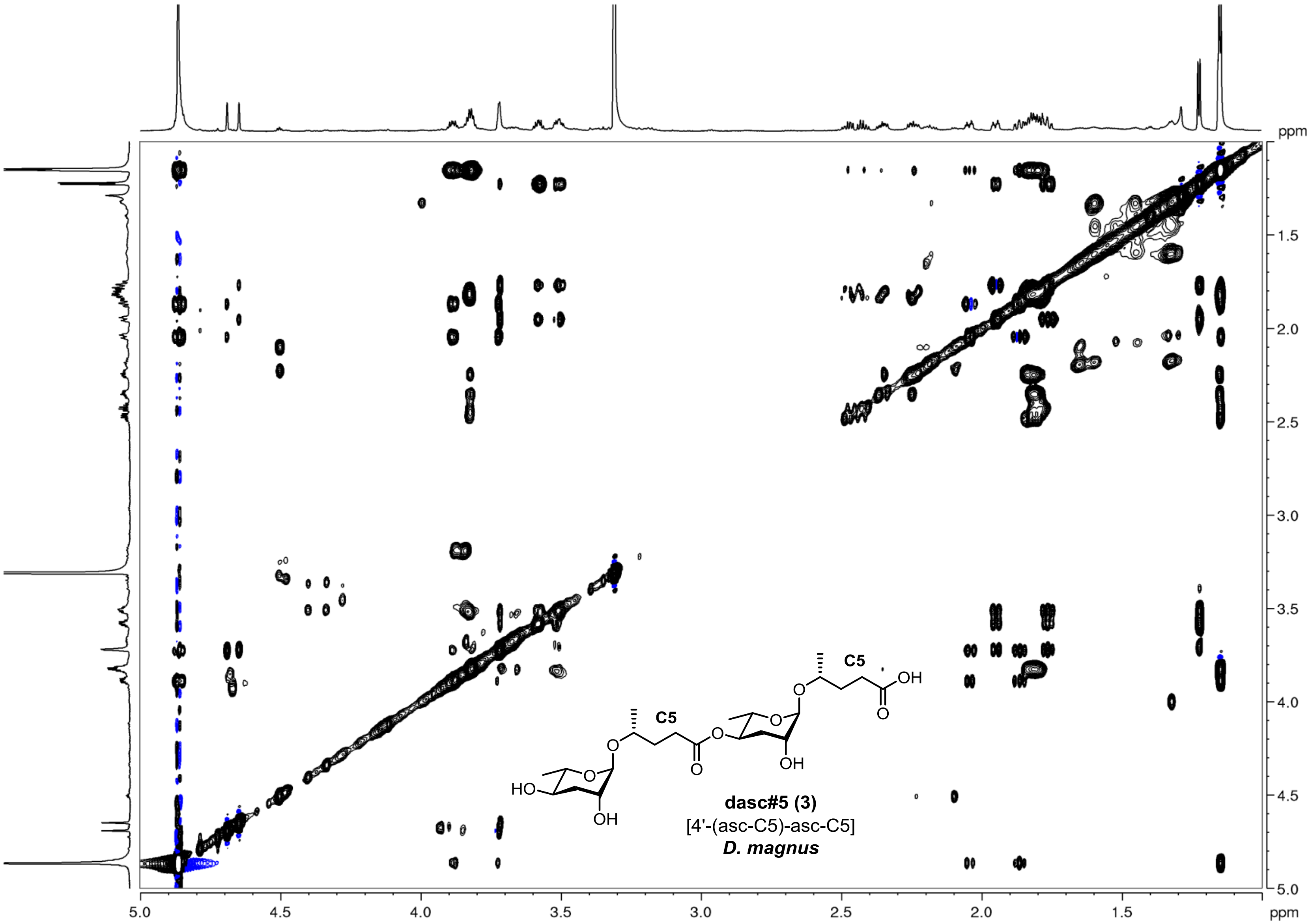

Figure 23. TOCSY spectrum of dasc#5 [4'-(asc-C5)-asc-C5, **3**] (800 MHz, CD_3OD) isolated from the *exo*-metabolome of *D. magnus*.

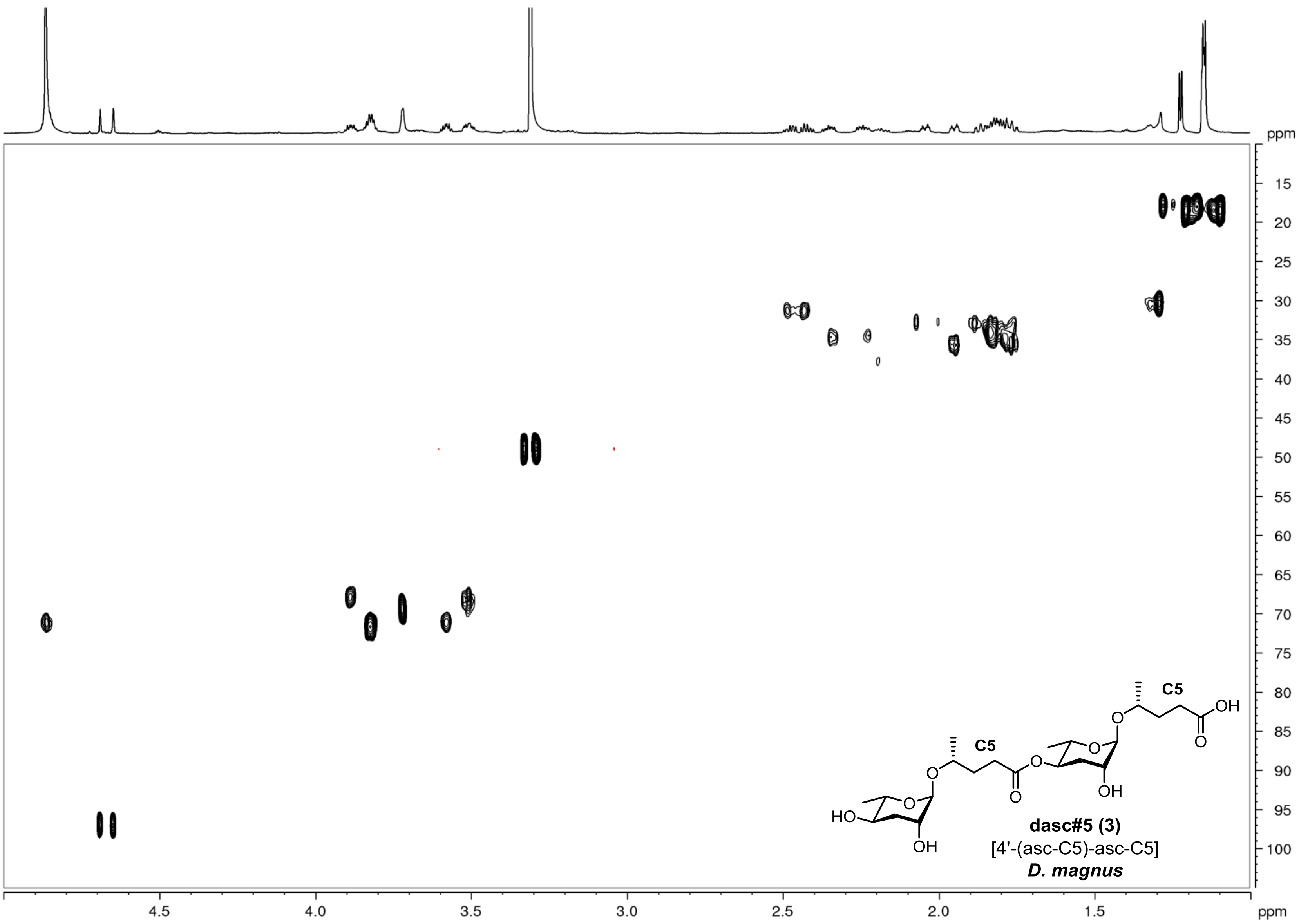

Figure 24. HSQC spectrum of dasc#5 [4'-(asc-C5)-asc-C5, **3**] (800 MHz, CD_3OD) isolated from the *exo*-metabolome of *D. magnus*.

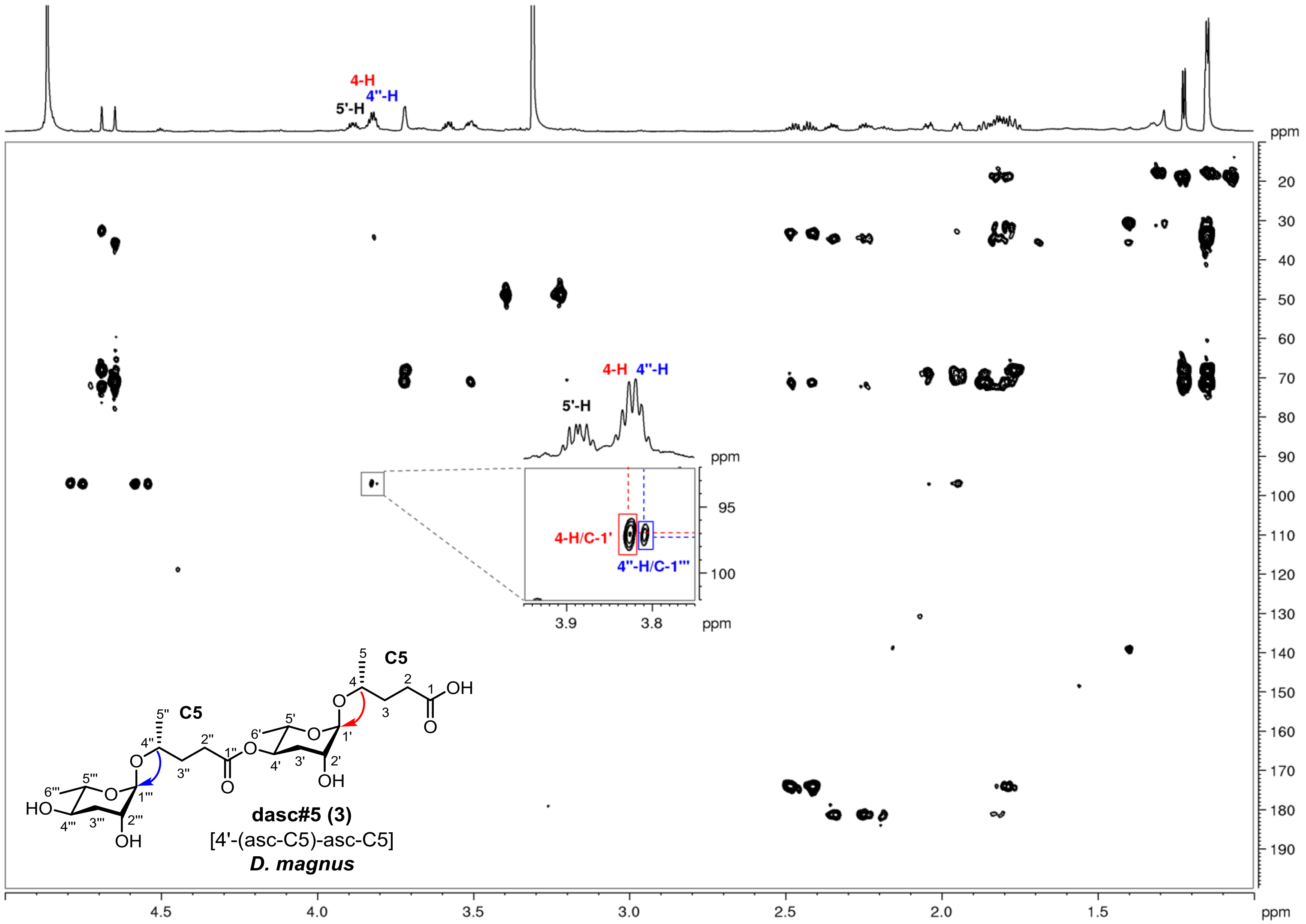

Figure 25. HMBC spectrum of dasc#5 [4'-(asc-C5)-asc-C5, **3**] (800 MHz, CD_3OD) isolated from *D. magnus*. Weak correlation signals from H-4 to C-1' (red colour marked signals) and from H-4" to C-1''' (blue colour marked signals) are amplified and highlighted in the middle of HMBC spectrum.

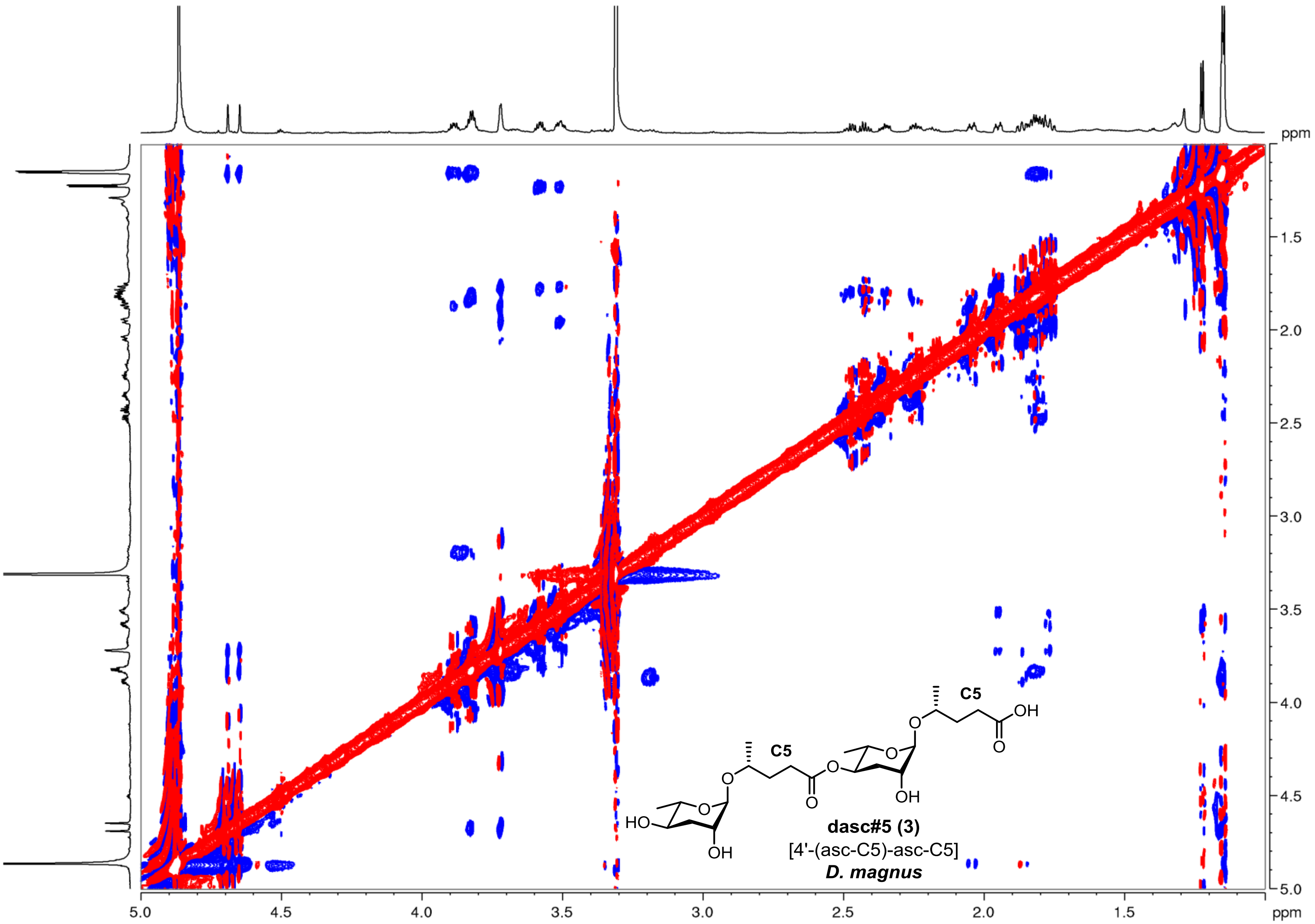

Figure 26. NOESY spectrum of dasc#5 [4'-(asc-C5)-asc-C5, **3**] (800 MHz, CD_3OD) isolated from the *exo*-metabolome of *D. magnus*.

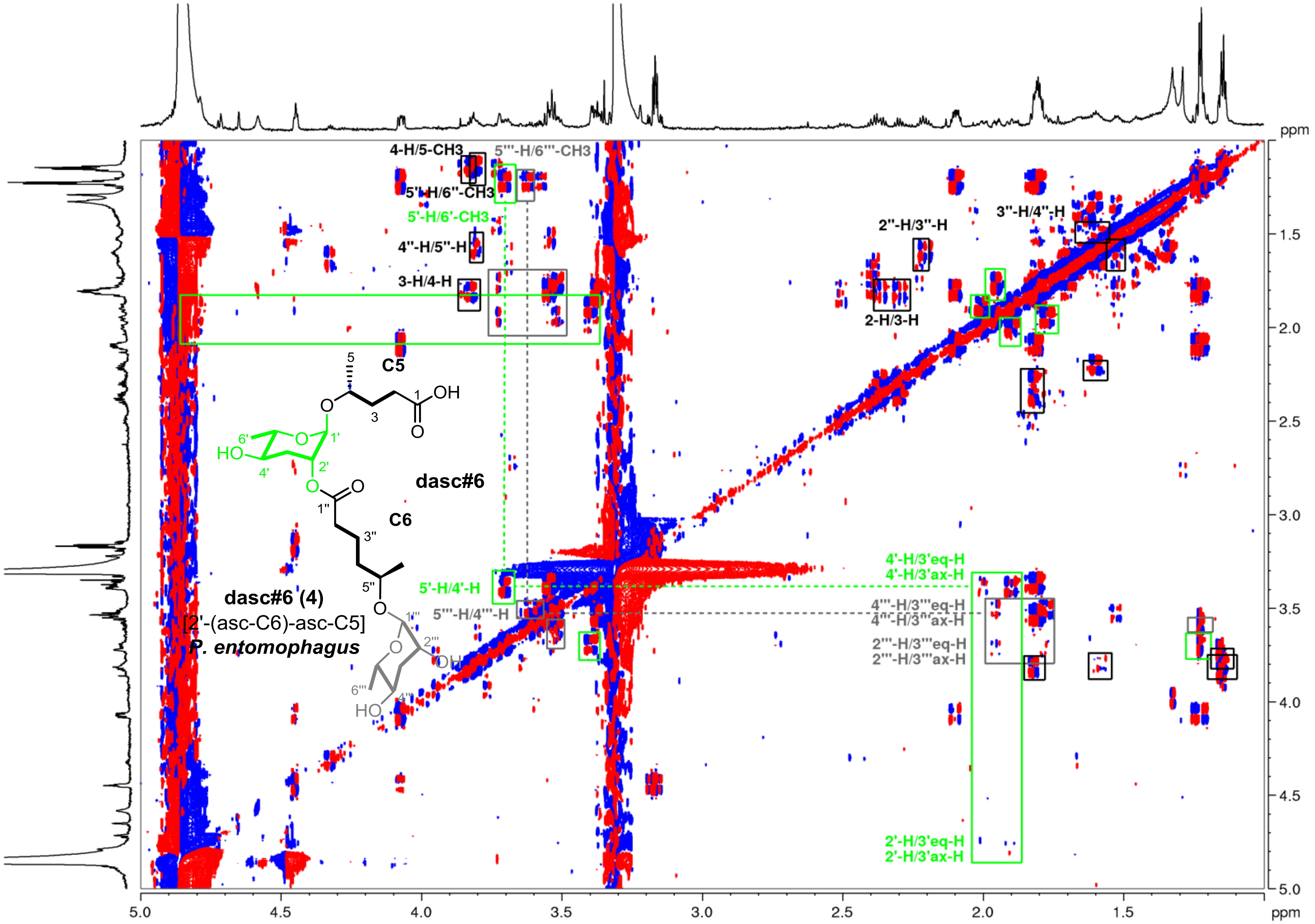

Figure 27. *dqf*-COSY spectrum of an enriched HPLC fraction containing dasc#6 [2'-(asc-C6)-asc-C5, **4**] (in CD_3OD) isolated from the *P. entomophagus exo*-metabolome. Green and grey colour boxed signals are derived from the 2'-substituted ascarylose sugar and free ascarylose sugar, respectively. Black colour boxed signals are derived from the C5 and C6 fatty acid side chains.

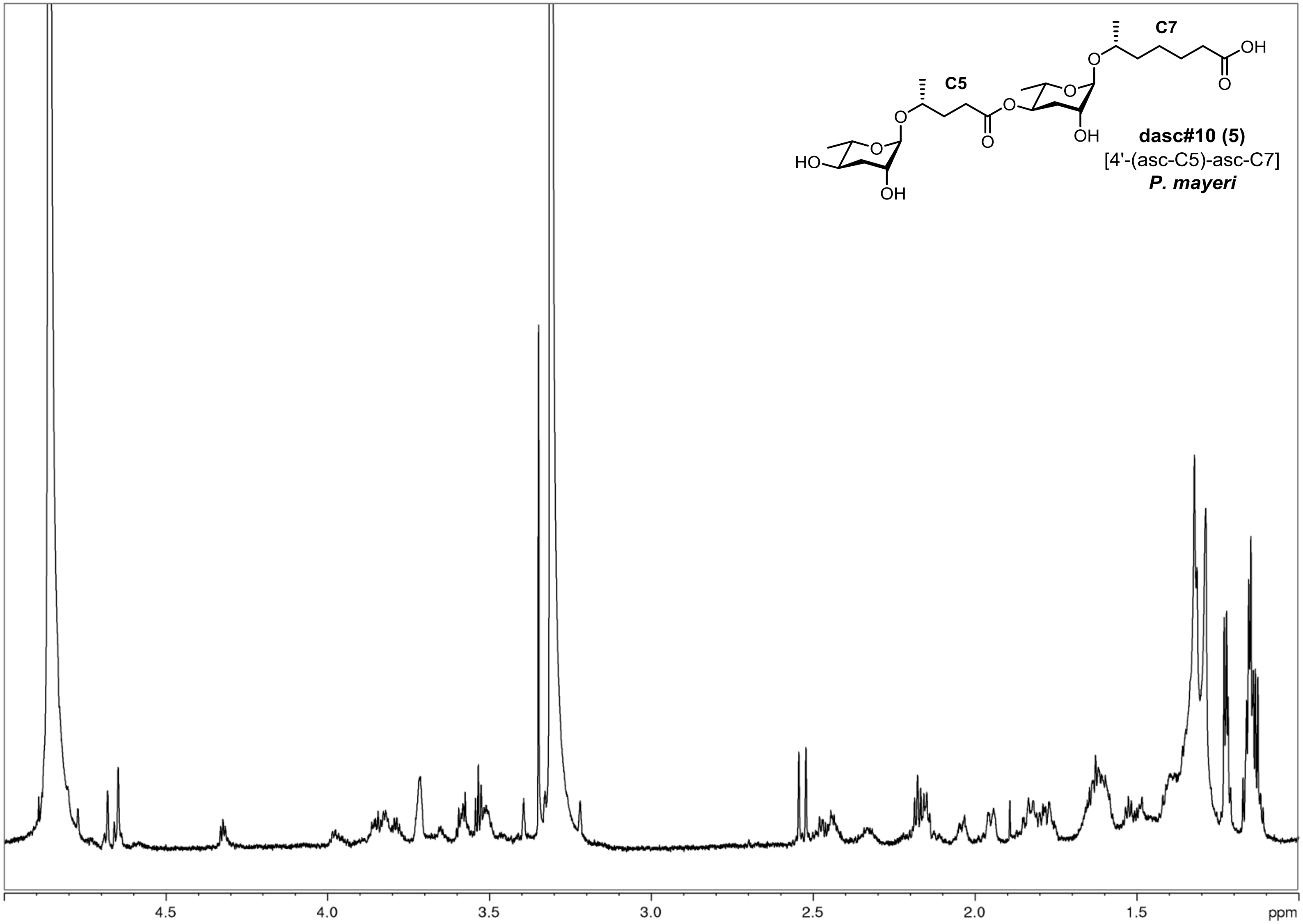

Figure 28. ^{1}H NMR spectrum of an HPLC enriched sample containing dasc#10 [4'-(asc-C5)-asc-C7, **5**] (800 MHz, CD_3OD) isolated from the *exo*-metabolome of *P. mayeri*.

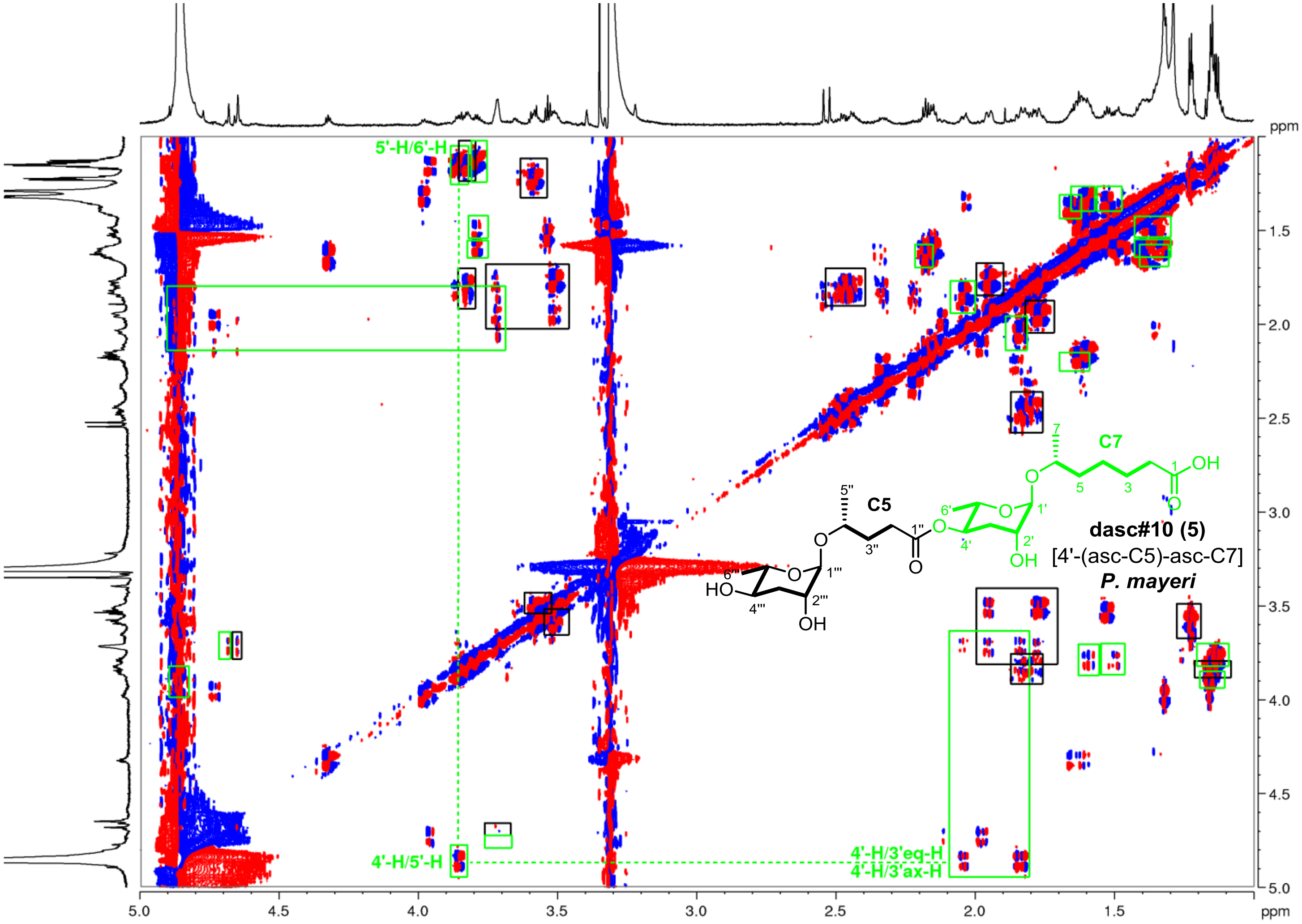

Figure 29. *dqf*-COSY spectrum of an HPLC enriched sample containing dasc#10 [4'-(asc-C5)-asc-C7, **5**] (800 MHz, CD_3OD) isolated from the *exo*-metabolome of *P. mayeri*. ^1H, ^1H-COSY correlation signals of the ascarylose sugar (green boxed signals) from ascr#1 [asc-C7] show that C-4' was substituted by ascr#9 [asc-C5] because the chemical shift of H-4' was shifted to the low field at 4.86 ppm. This suggests that dasc#10 [4'-(asc-C5)-asc-C7, **5**] is a dimeric ascaroside with ascr#9 [asc-C5] linked to the 4'-position of ascr#1 [asc-C7].

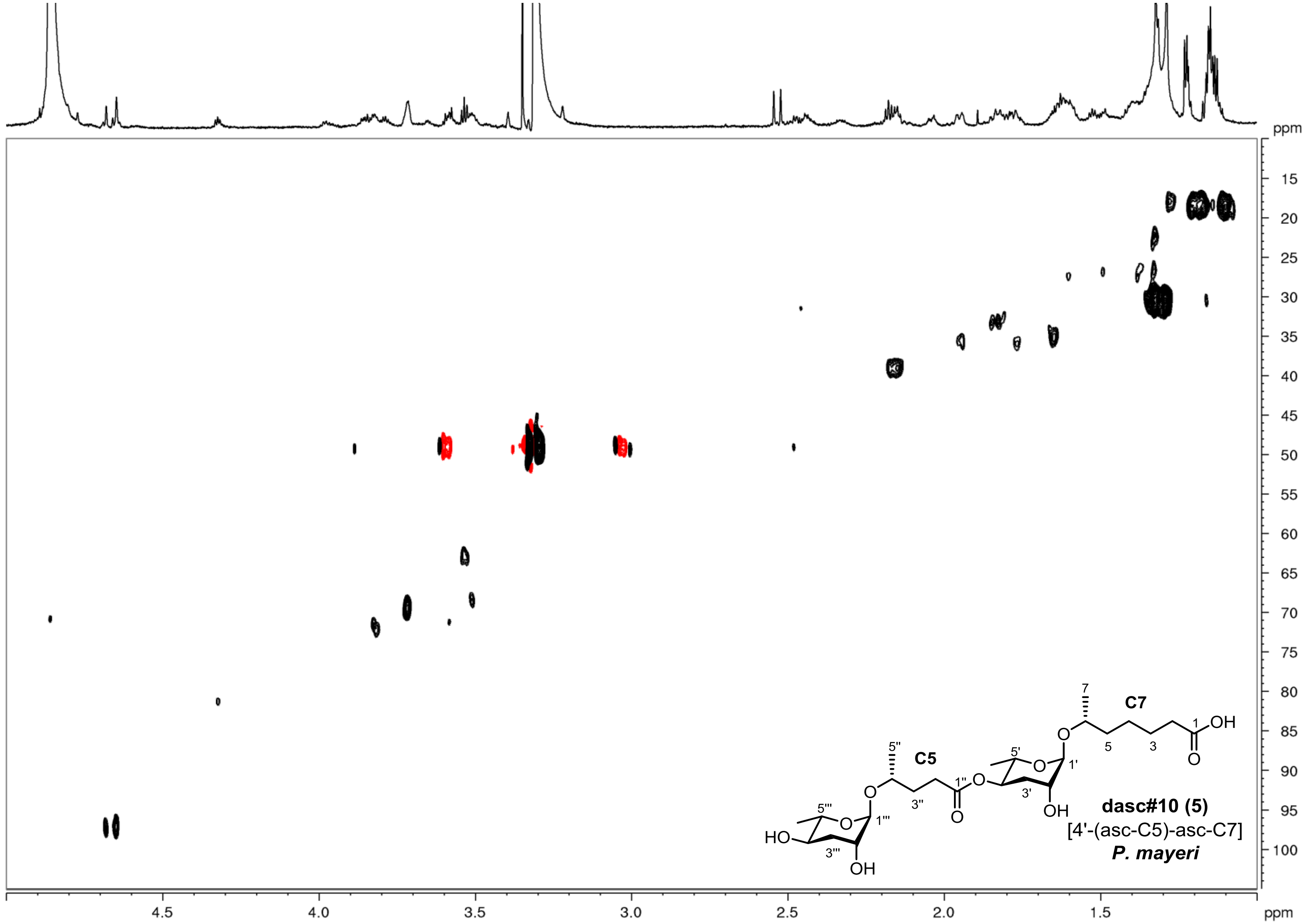

Figure 30. HSQC spectrum of an HPLC enriched sample containing dasc#10 [4'-(asc-C5)-asc-C7, **5**] (800 MHz, CD_3OD) isolated from the *exo*-metabolome of *P. mayeri*. Patial HSQC correlation signals of dasc#10 [4'-(asc-C5)-asc-C7, **5**] are observed and highlighted in the spectrum.

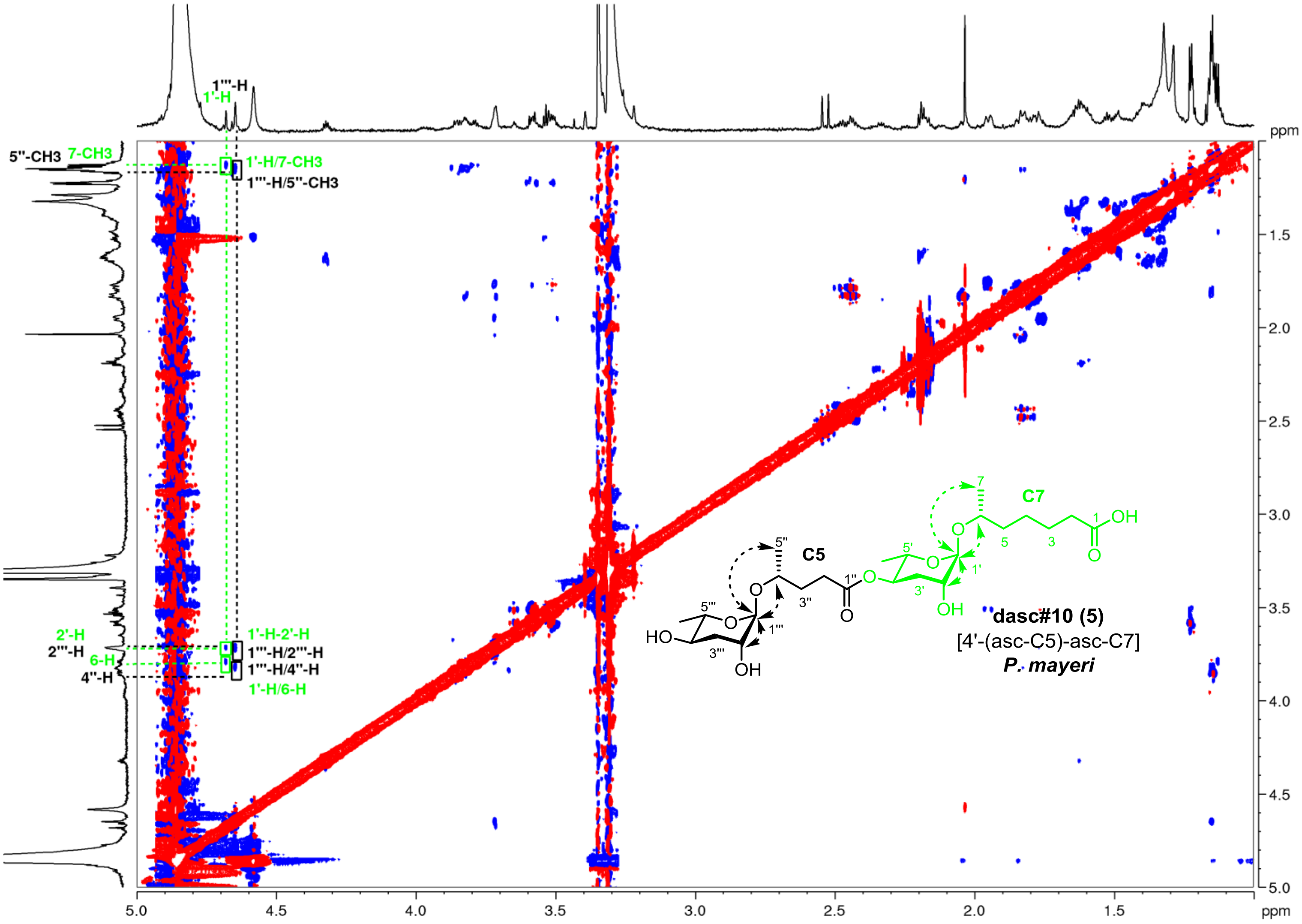

Figure 31. NOESY spectrum of an HPLC enriched sample containing dasc#10 [4'-(asc-C5)-asc-C7, **5**] (800 MHz, CD_3OD) isolated from the *exo*-metabolome of *P. mayeri*. Key NOE correlations of H-1'/H-6 and H-1'/7-CH_3 (green boxed signals) indicate that the 4'-substituted ascarylose sugar is linked to the C7 fatty acid side chain. Key NOE correlations of H-1'''/H-4'' and H-1'''/5''-CH_3 (black boxed signals) indicate that the free ascarylose in dasc#10 (**5**) is linked to the C5 fatty acid side chain.

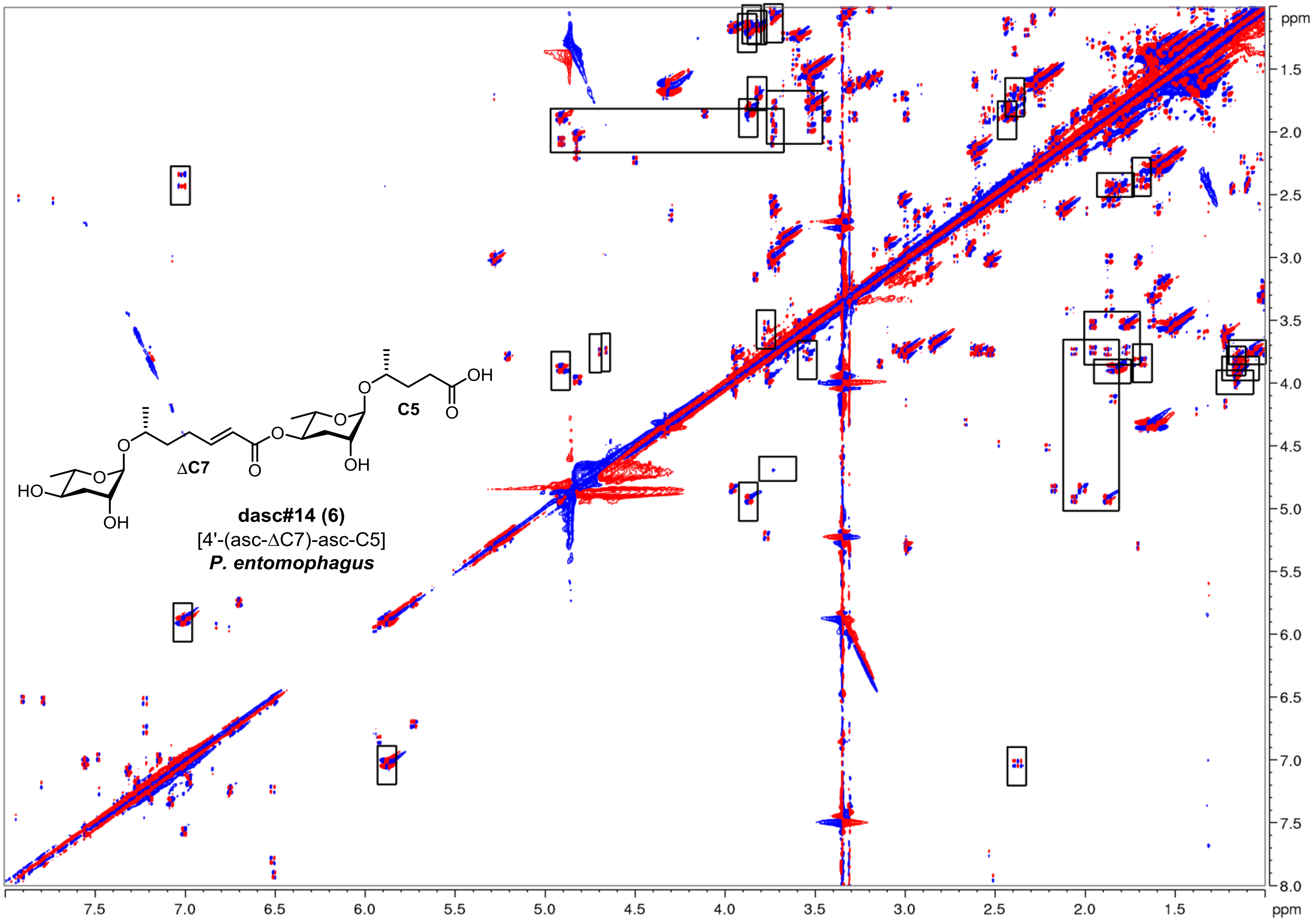

Figure 32. *dqf*-COSY spectrum of an SPE enriched sample containing dasc#14 [4'-(asc-ΔC7)-asc-C5, **6**] (800 MHz, CD_3OD) isolated from the *exo*-metabolome of *P. entomophagus*.

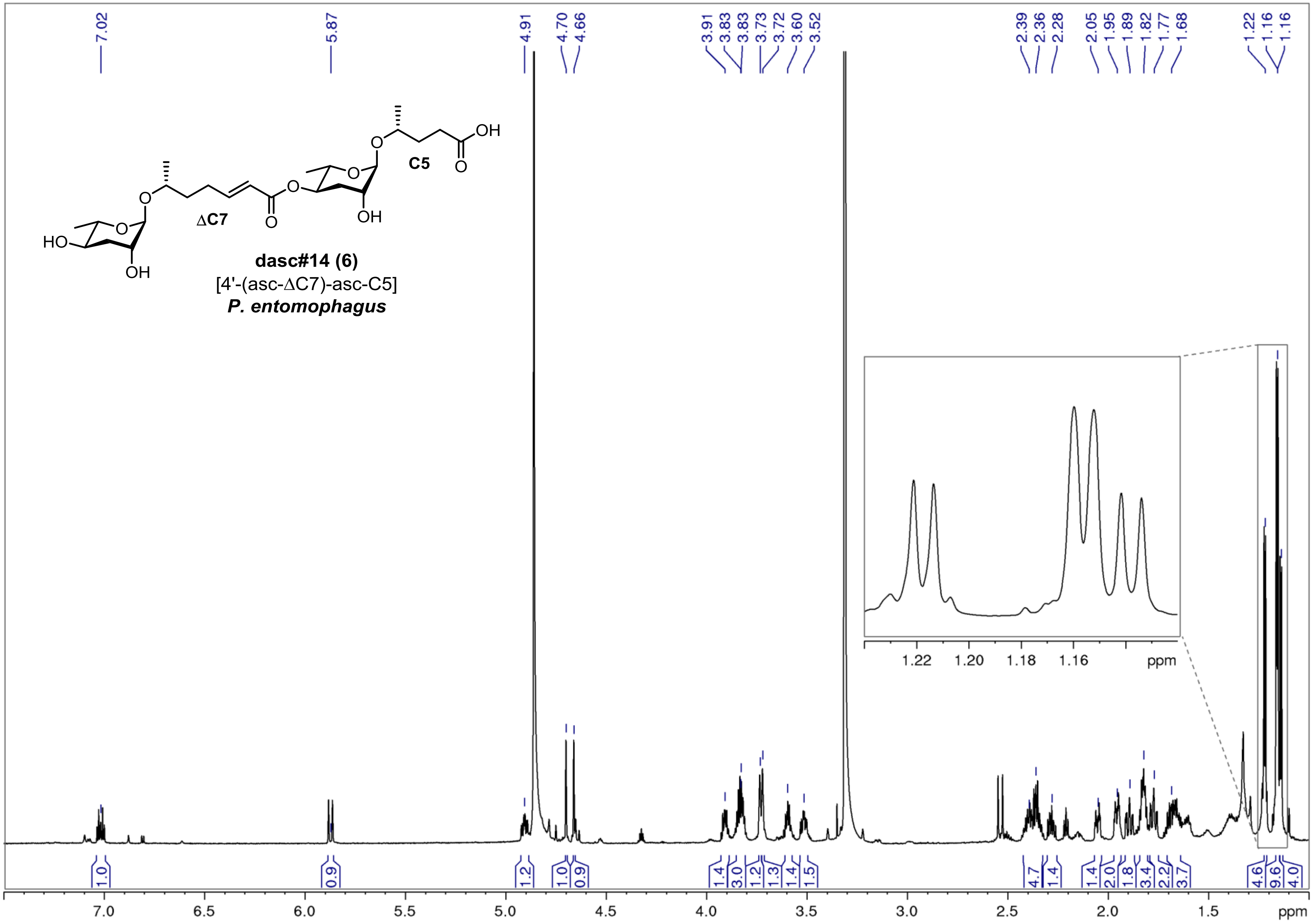

Figure 33. ^{1}H NMR spectrum of dasc#14 [4'-(asc-ΔC7)-asc-C5, **6**] (800 MHz, CD_3OD) isolated from the *exo*-metabolome of *P. entomophagus*.

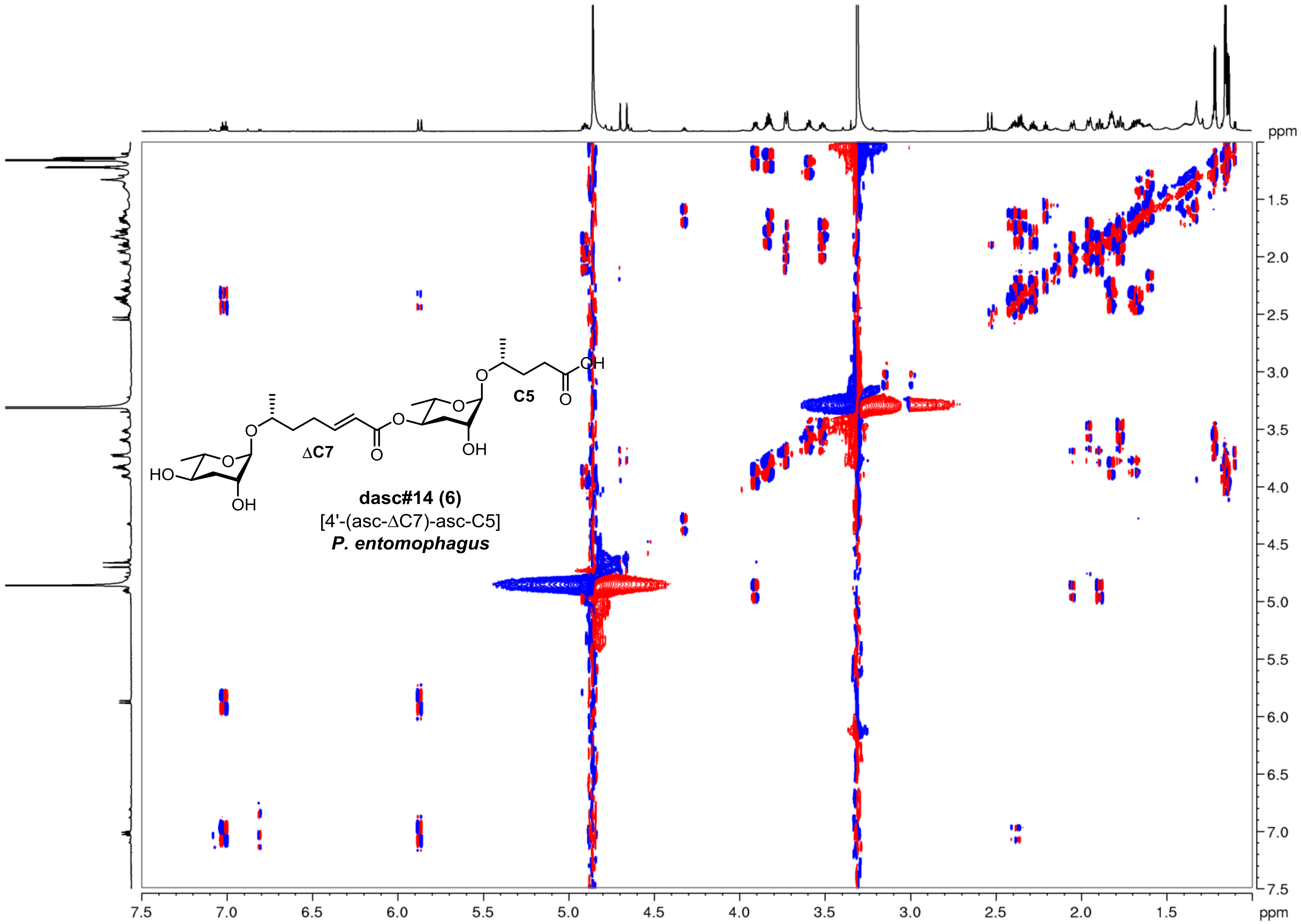

Figure 34. *dqf*-COSY spectrum of dasc#14 [4'-(asc-ΔC7)-asc-C5, **6**] (800 MHz, CD_3OD) isolated from the *exo*-metabolome of *P. entomophagus*.

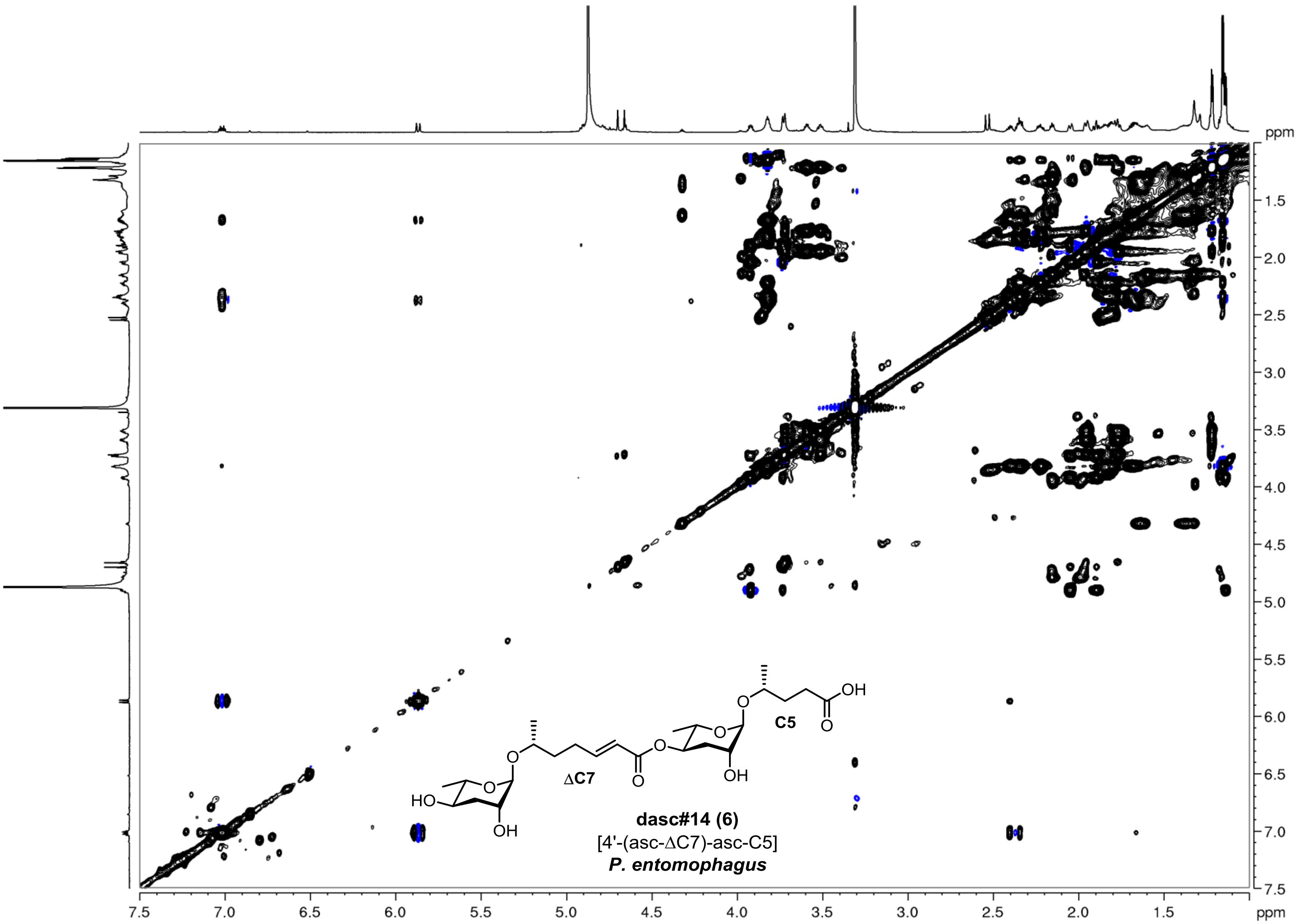

Figure 35. TOCSY spectrum of dasc#14 [4'-(asc-ΔC7)-asc-C5, **6**] (800 MHz, CD_3OD) isolated from the *exo*-metabolome of *P. entomophagus*.

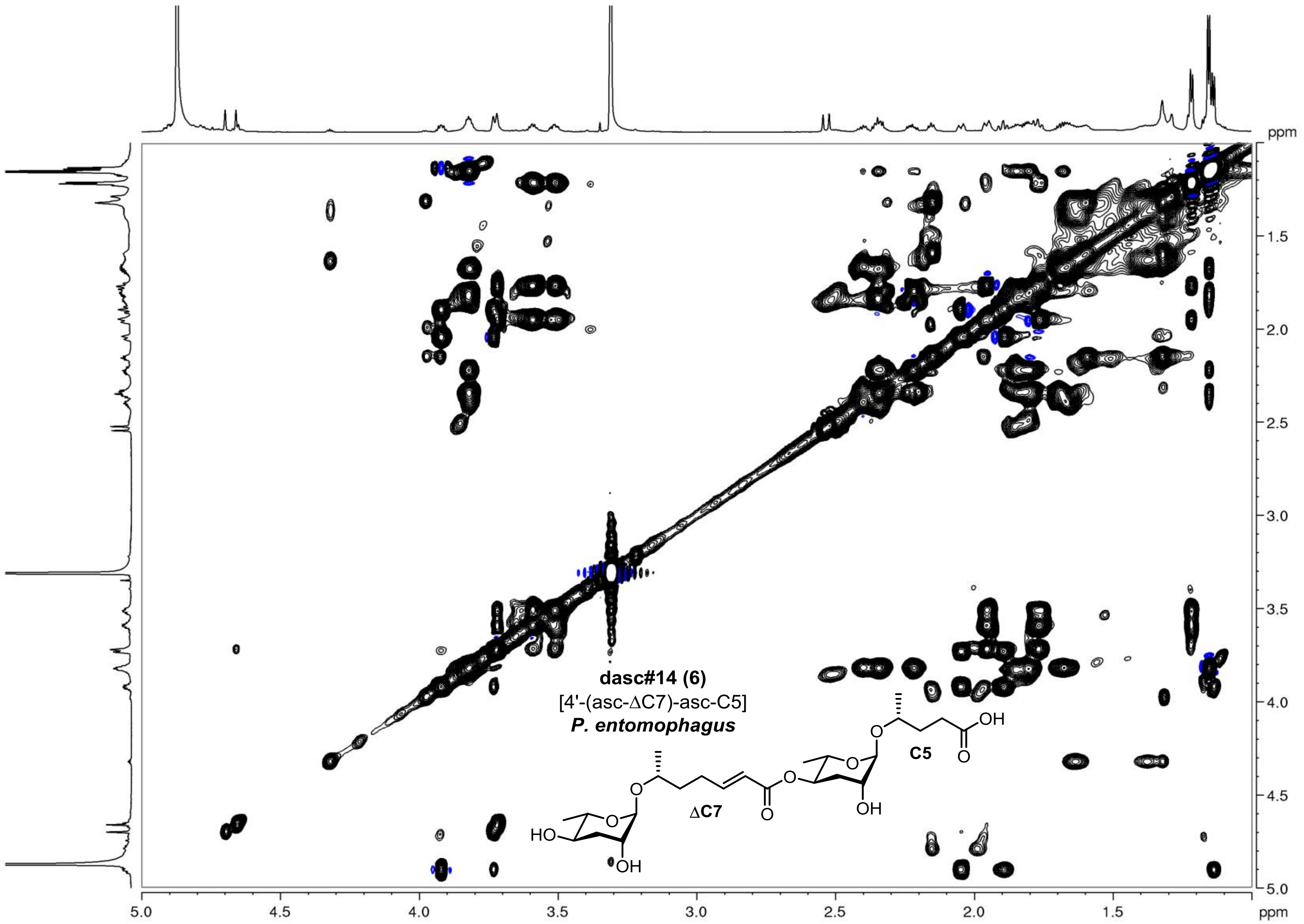

Figure 36. Selected TOCSY spectrum (amplified region from 1.0 ppm to 5.0 ppm) of dasc#14 [4'-(asc-ΔC7)-asc-C5, **6**] (800 MHz, CD_3OD) isolated from *P. entomophagus*.

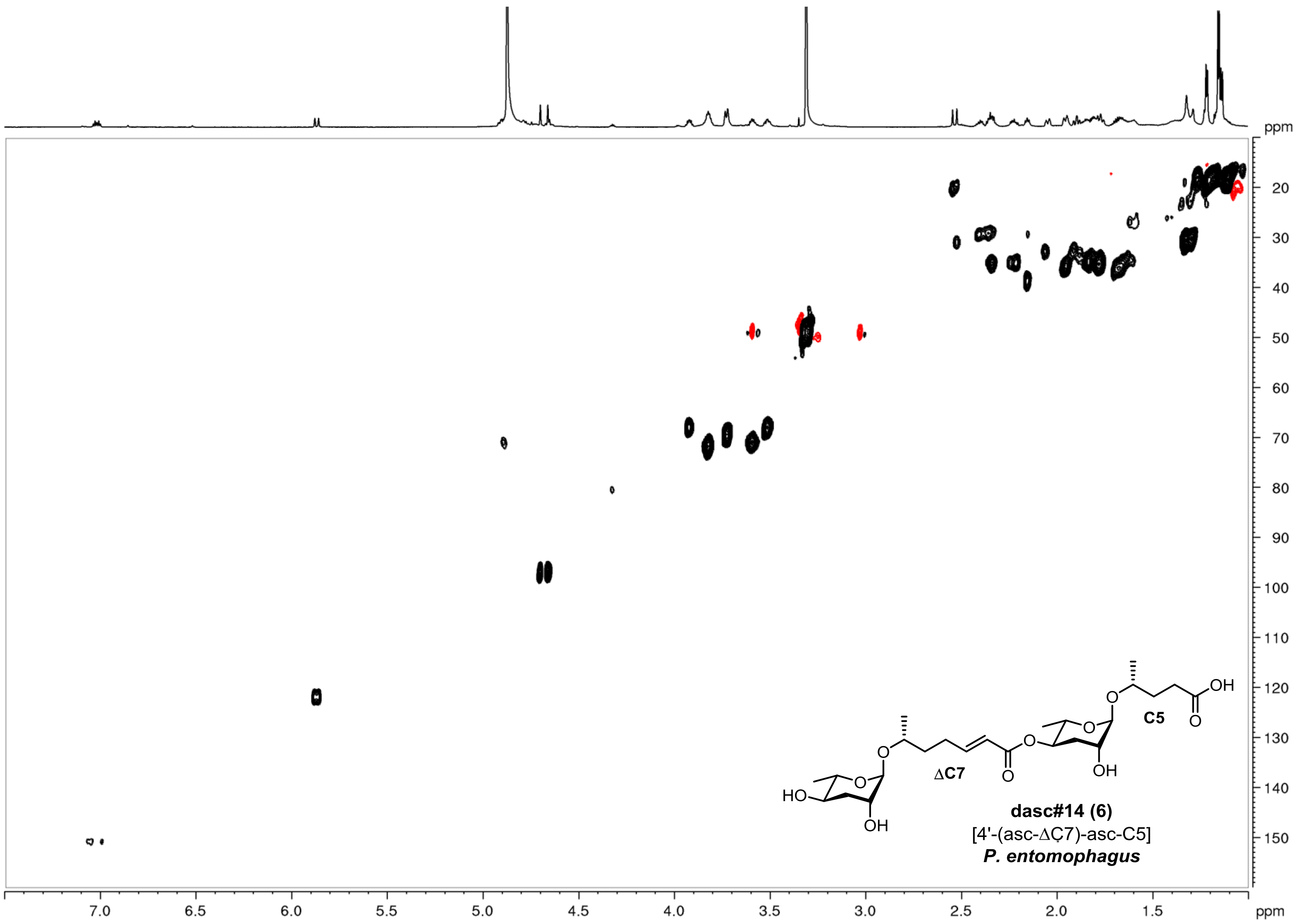

Figure 37. HSQC spectrum of dasc#14 [4'-(asc-ΔC7)-asc-C5, **6**] (800 MHz, CD_3OD) isolated from the *exo*-metabolome of *P. entomophagus*.

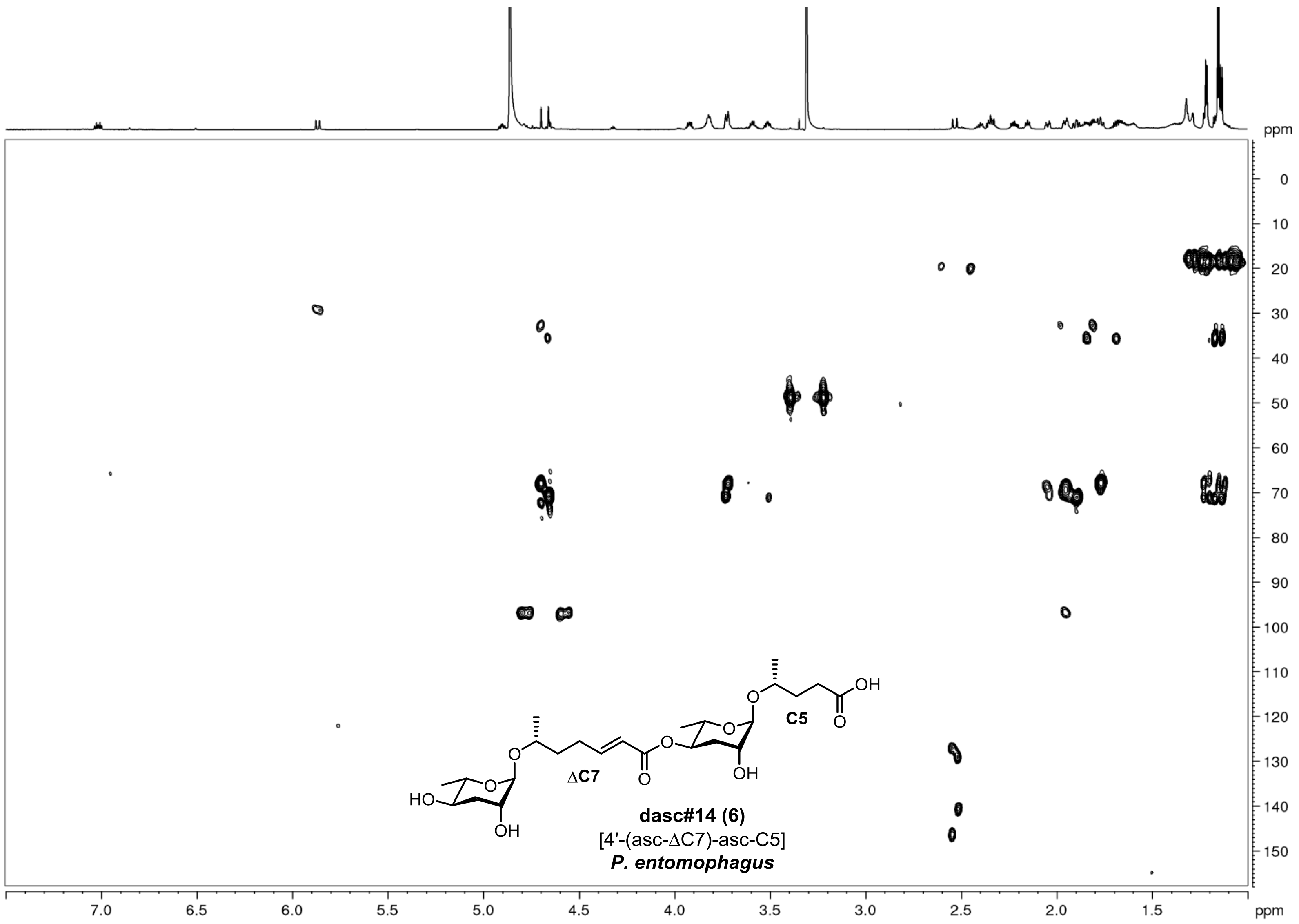

Figure 38. HMBC spectrum of dasc#14 [4'-(asc-ΔC7)-asc-C5, **6**] (800 MHz, CD_3OD) isolated from the *exo*-metabolome of *P. entomophagus*. Only partial HMBC correlation signals are observed due to the low concentration of dasc#14 [4'-(asc-ΔC7)-asc-C5, **6**].

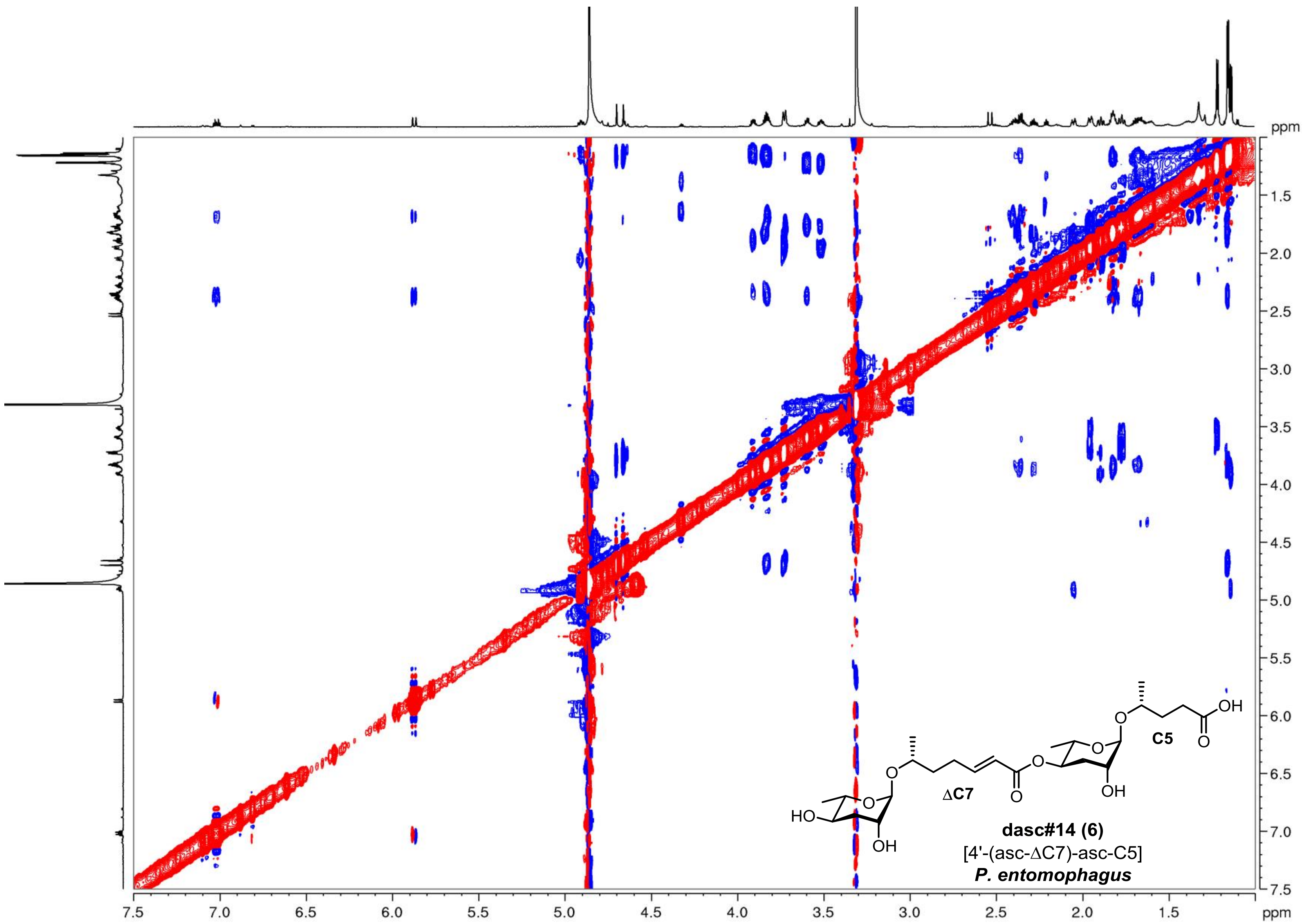

Figure 39. NOESY spectrum of dasc#14 [4'-(asc-ΔC7)-asc-C5, **6**] (800 MHz, CD_3OD) isolated from the *exo*-metabolome of *P. entomophagus*.

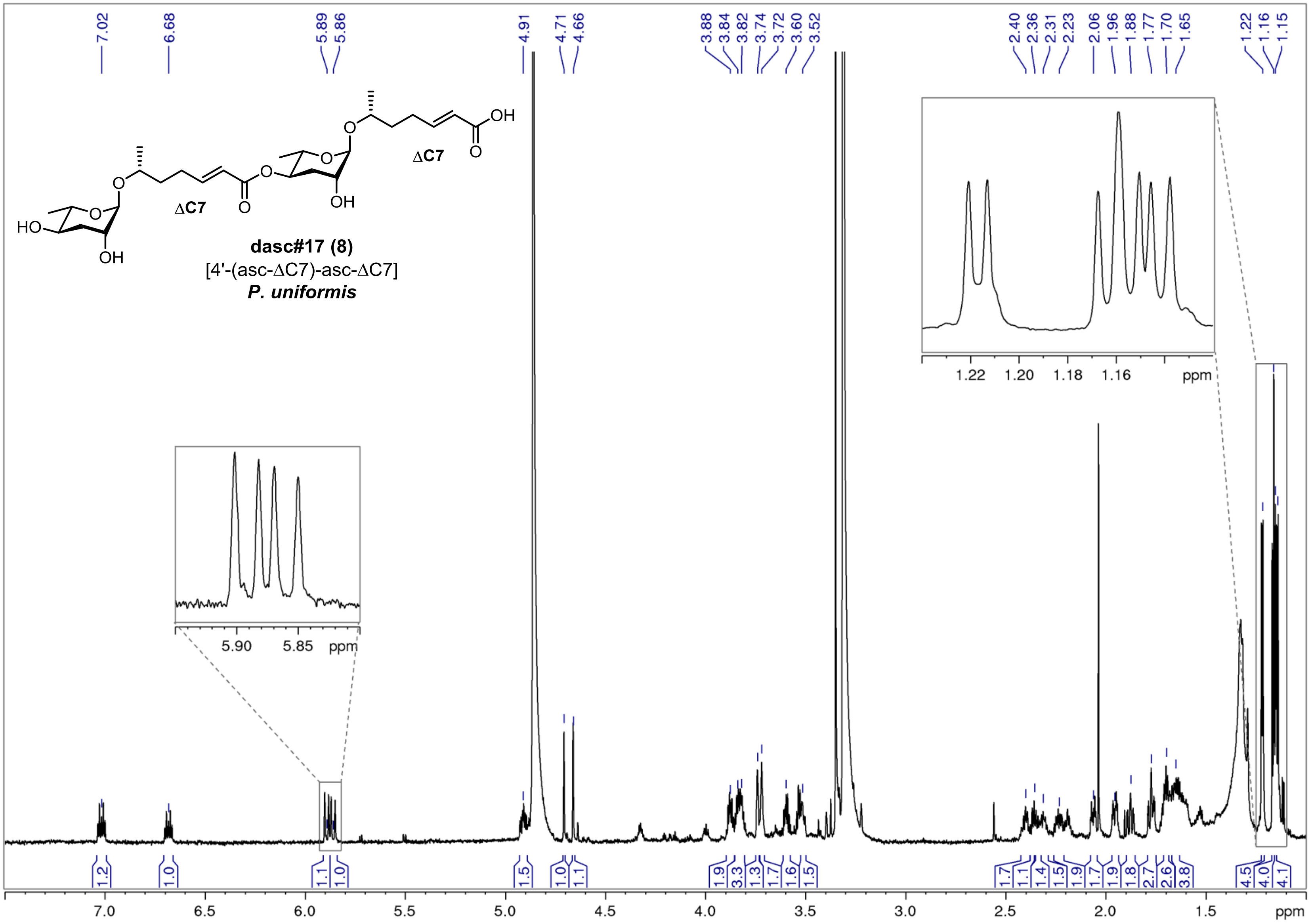

Figure 40. ^{1}H NMR spectrum of dasc#17 [4'-(asc-ΔC7)-asc-ΔC7, **8**] (800 MHz, CD_3OD) isolated from the *exo*-metabolome of *P. uniformis*.

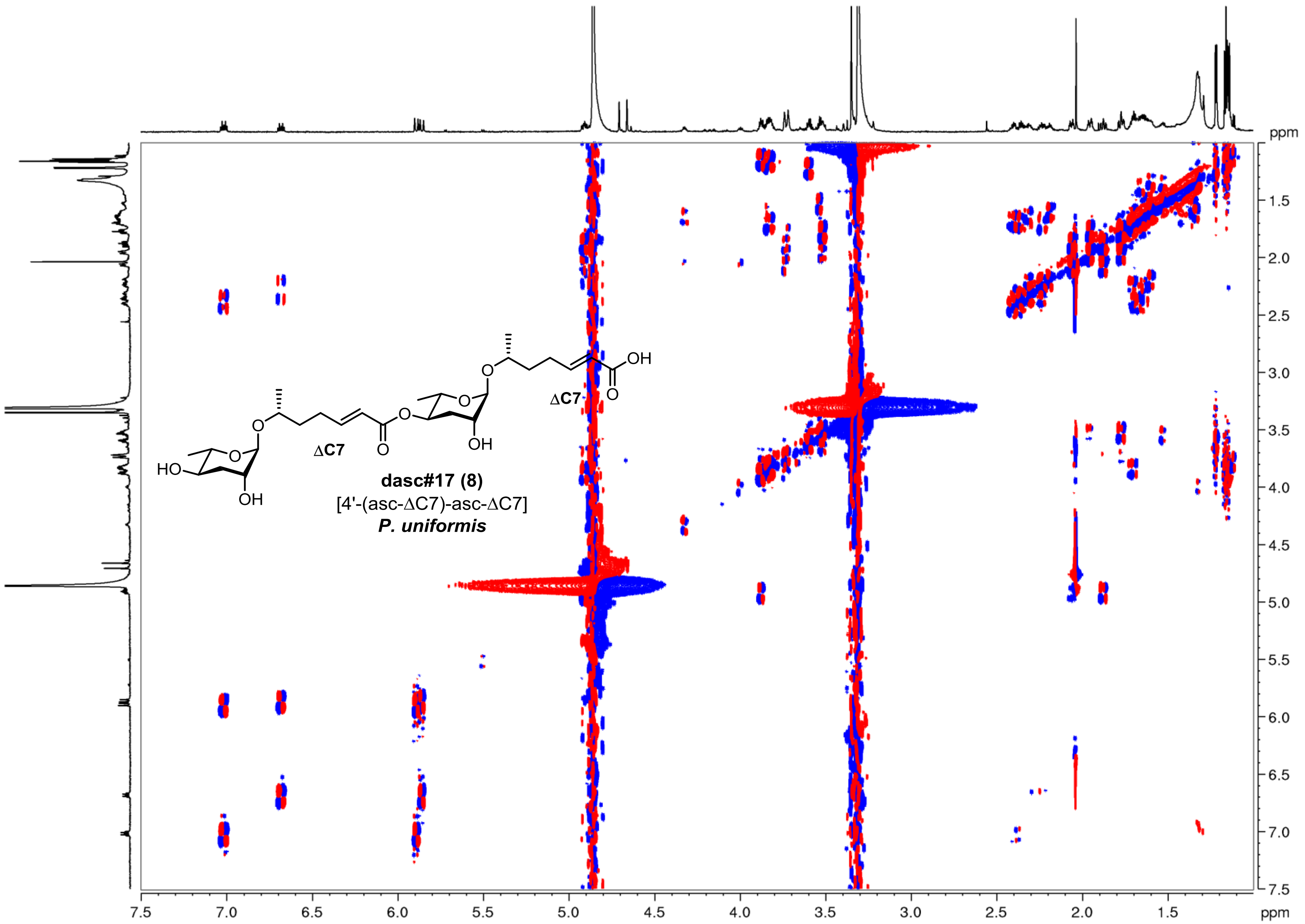

Figure 41. *dqf*-COSY spectrum of dasc#17 [4'-(asc-ΔC7)-asc-ΔC7, **8**] (800 MHz, CD_3OD) isolated from the *exo*-metabolome of *P. uniformis*.

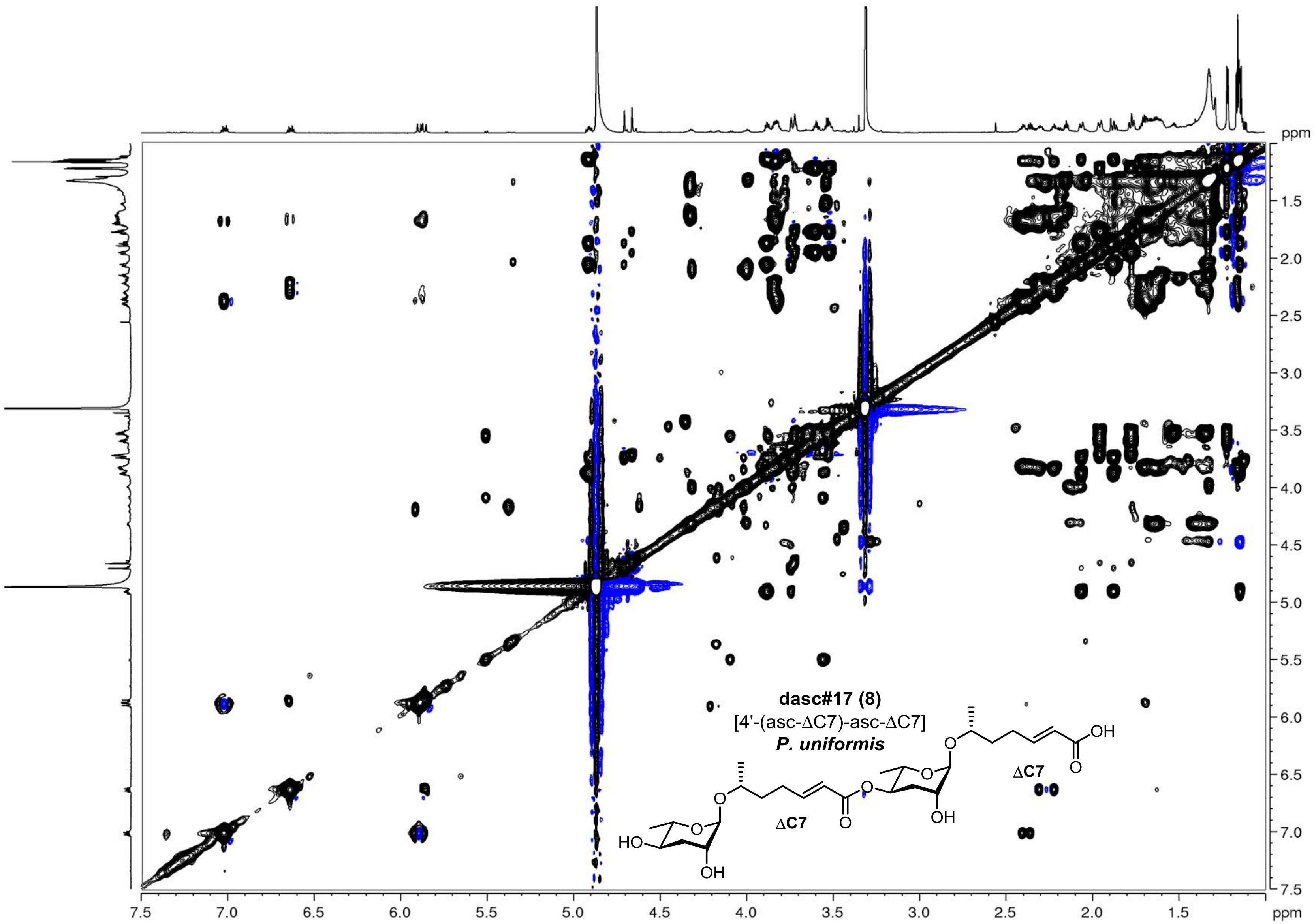

Figure 42. TOCSY spectrum of dasc#17 [4'-(asc-ΔC7)-asc-ΔC7, **8**] (800 MHz, CD_3OD) isolated from the *exo*-metabolome of *P. uniformis*.

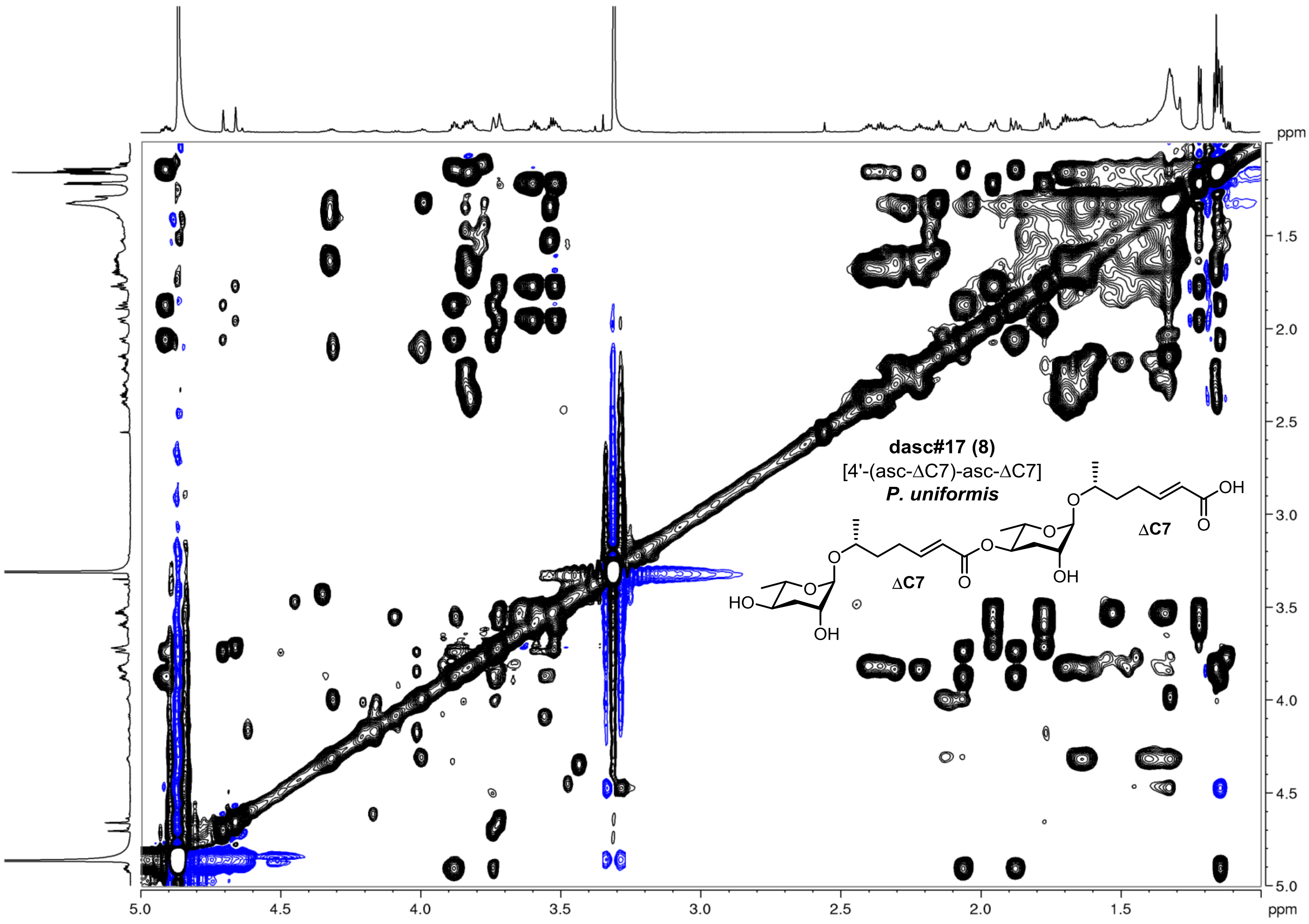

Figure 43. Selected partial TOCSY spectrum of dasc#17 [4'-(asc-ΔC7)-asc-ΔC7, **8**] (800 MHz, CD_3OD) isolated from the *exo*-metabolome of *P. uniformis*.

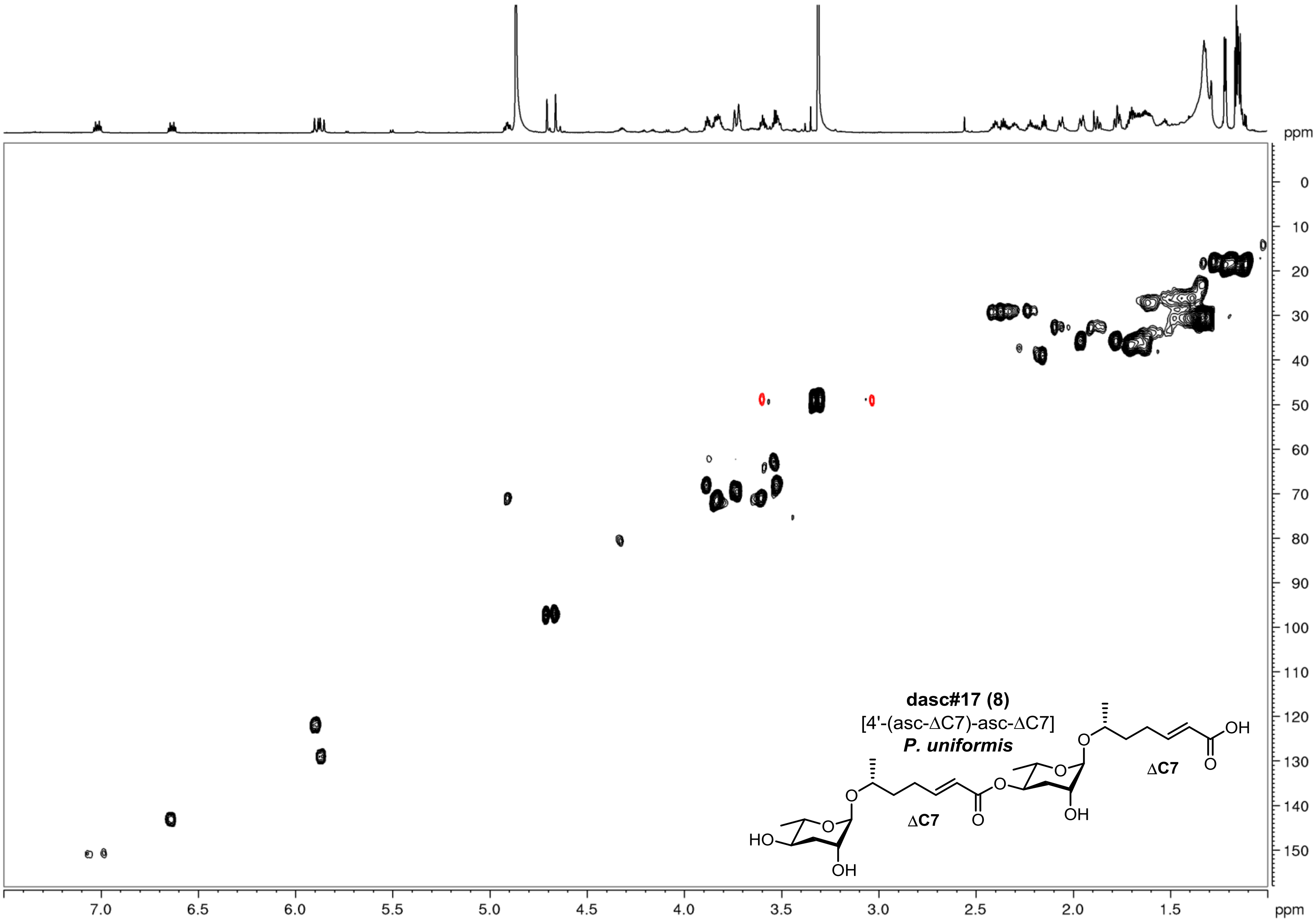

Figure 44. HSQC spectrum of dasc#17 [4'-(asc-ΔC7)-asc-ΔC7, **8**] (800 MHz, CD_3OD) isolated from the *exo*-metabolome of *P. uniformis*.

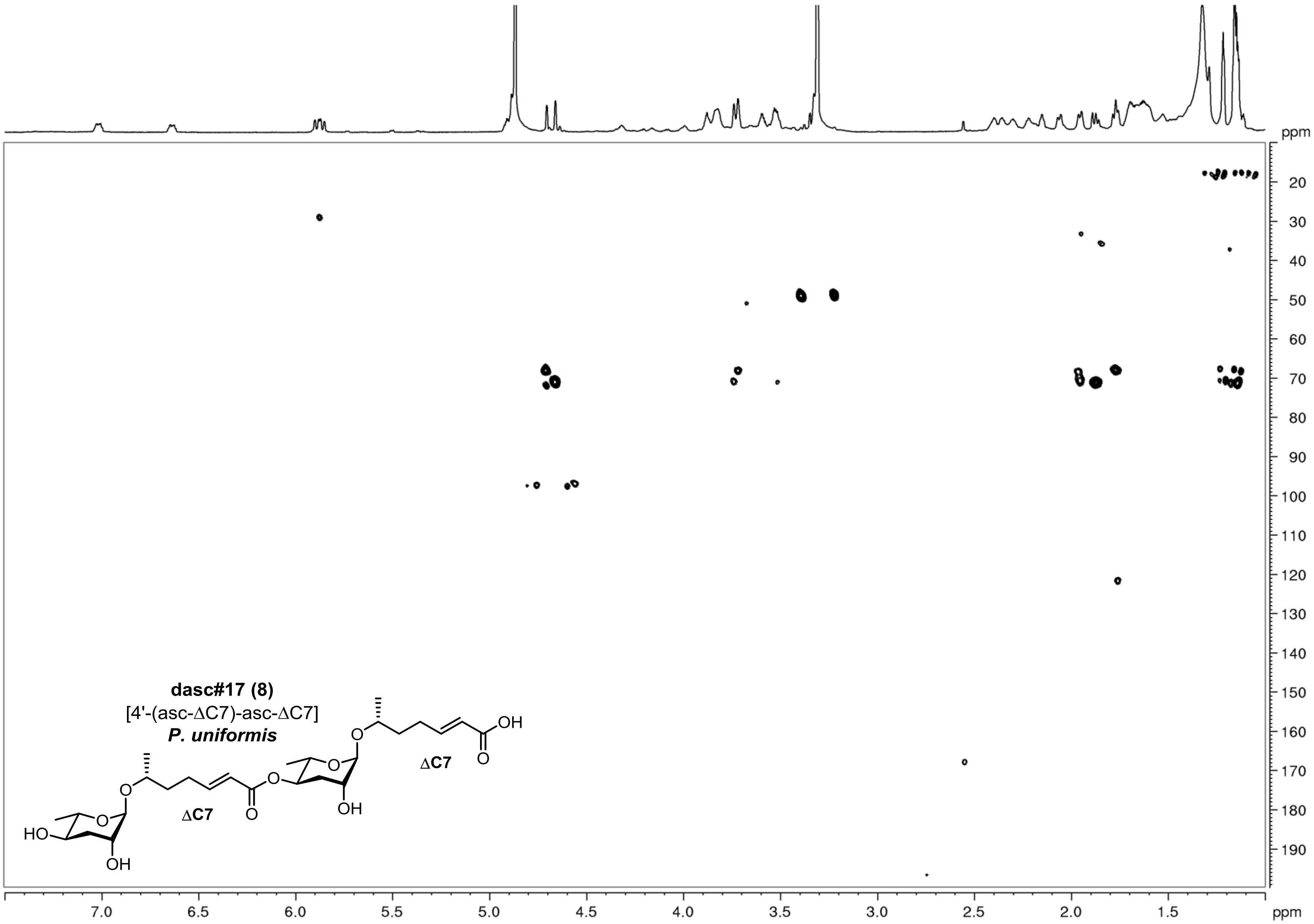

Figure 45. HMBC spectrum of dasc#17 [4'-(asc-ΔC7)-asc-ΔC7, **8**] (800 MHz, CD_3OD) isolated from the *exo*-metabolome of *P. uniformis*.

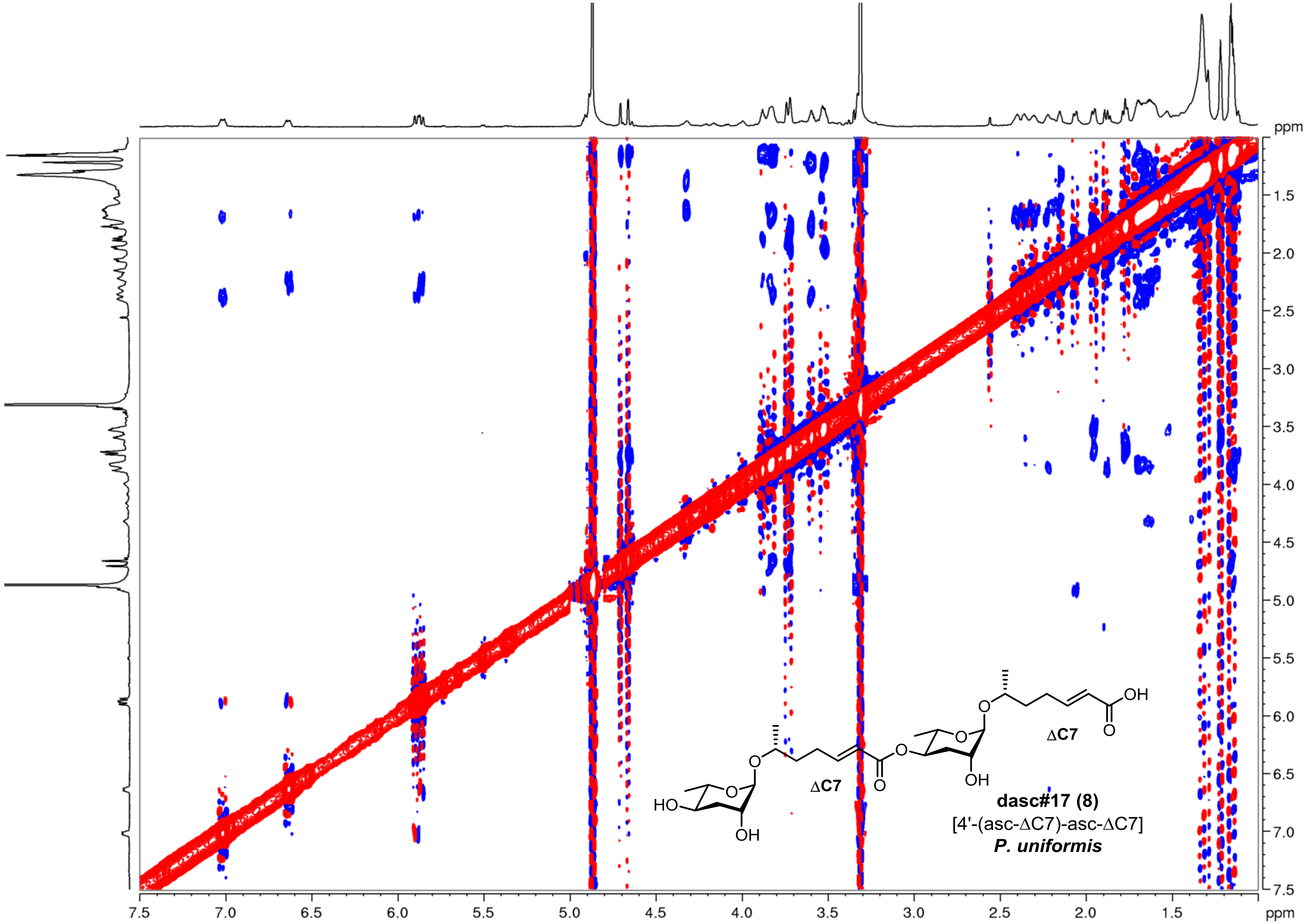

Figure 46. NOESY spectrum of dasc#17 [4'-(asc-ΔC7)-asc-ΔC7, **8**] (800 MHz, CD_3OD) isolated from the *exo*-metabolome of *P. uniformis*.

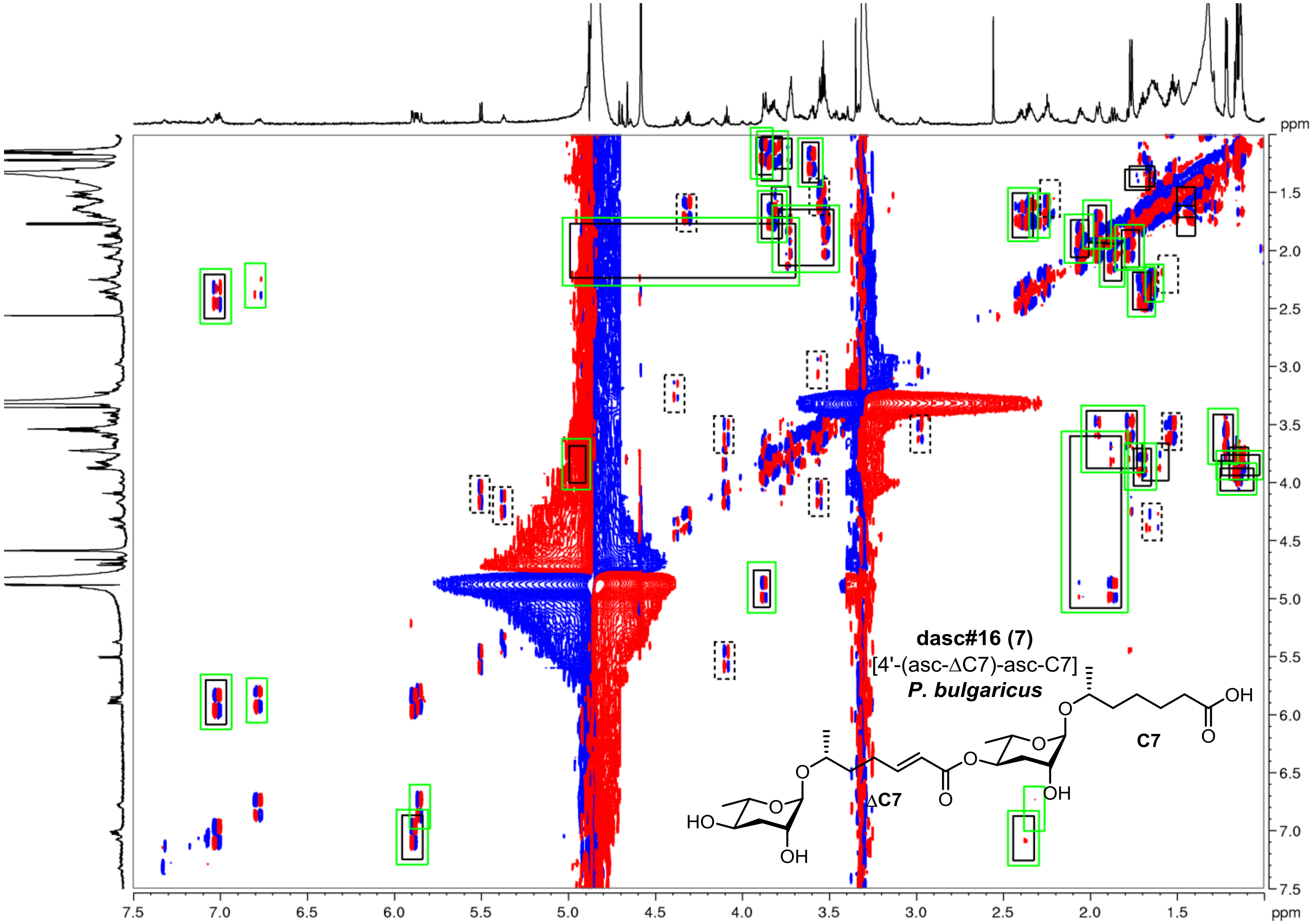

Figure 47. *dqf*-COSY spectrum (800 MHz, CD_3OD) of an HPLC enriched sample containing a mixture of dasc#16 [4'-(asc-ΔC7)-asc-C7, **7**] and dasc#17 [4'-(asc-ΔC7)-asc-ΔC7, **8**] (ratio of ca. 1:1) isolated from the *P. bulgaricus exo*-metabolome. Both hydrogens of dasc#16 (**7**) and dasc#17 (**8**) at the 4'-position are observed to be shifted to ca. 5.0 ppm, indicating that ascr#7 [asc-ΔC7] is linked to the 4'-position of ascr#1 [asc-C7] in dasc#16. Black colour boxed signals are derived from dasc#16 (**7**) and green colour boxed signals are derived from dasc#17 (**8**). Dashed black colour boxed signals are from impurities.

supplementary file 1c: NMR spectra of PASC chemicals Pages

Figures 1-4. NMR spectra of pasc#9 (800 MHz, CD_3OD) isolated from *P. taiwanensis*. S76
Figures 5-13. NMR spectra of paso#9 (800 MHz, CD_3OD) isolated from *P. pacificus*. S80

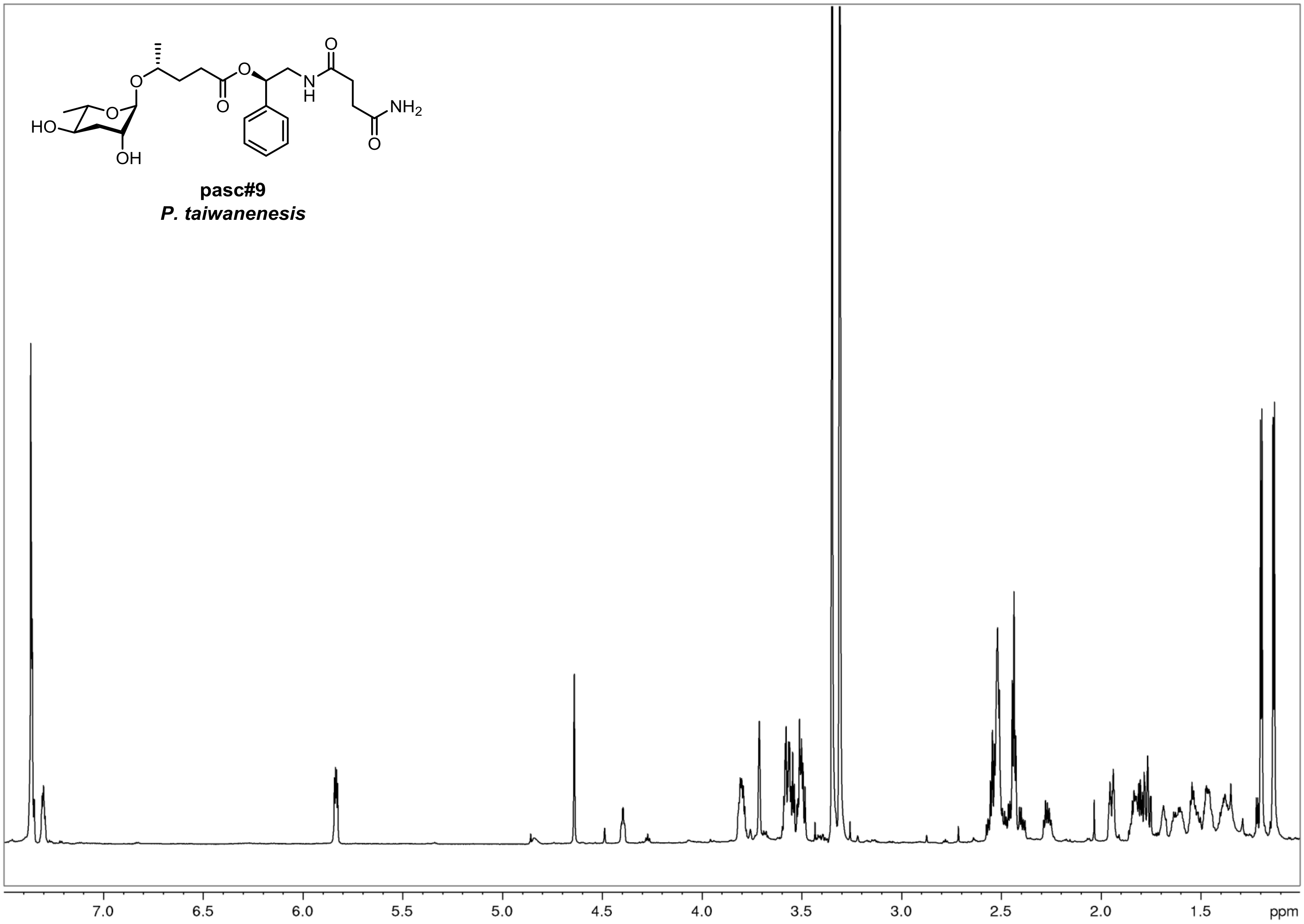

Figure 1. ^{1}H NMR spectrum of pasc#9 (800 MHz, CD_3OD) isolated from the *exo*-metabolome of *P. taiwanensis*.

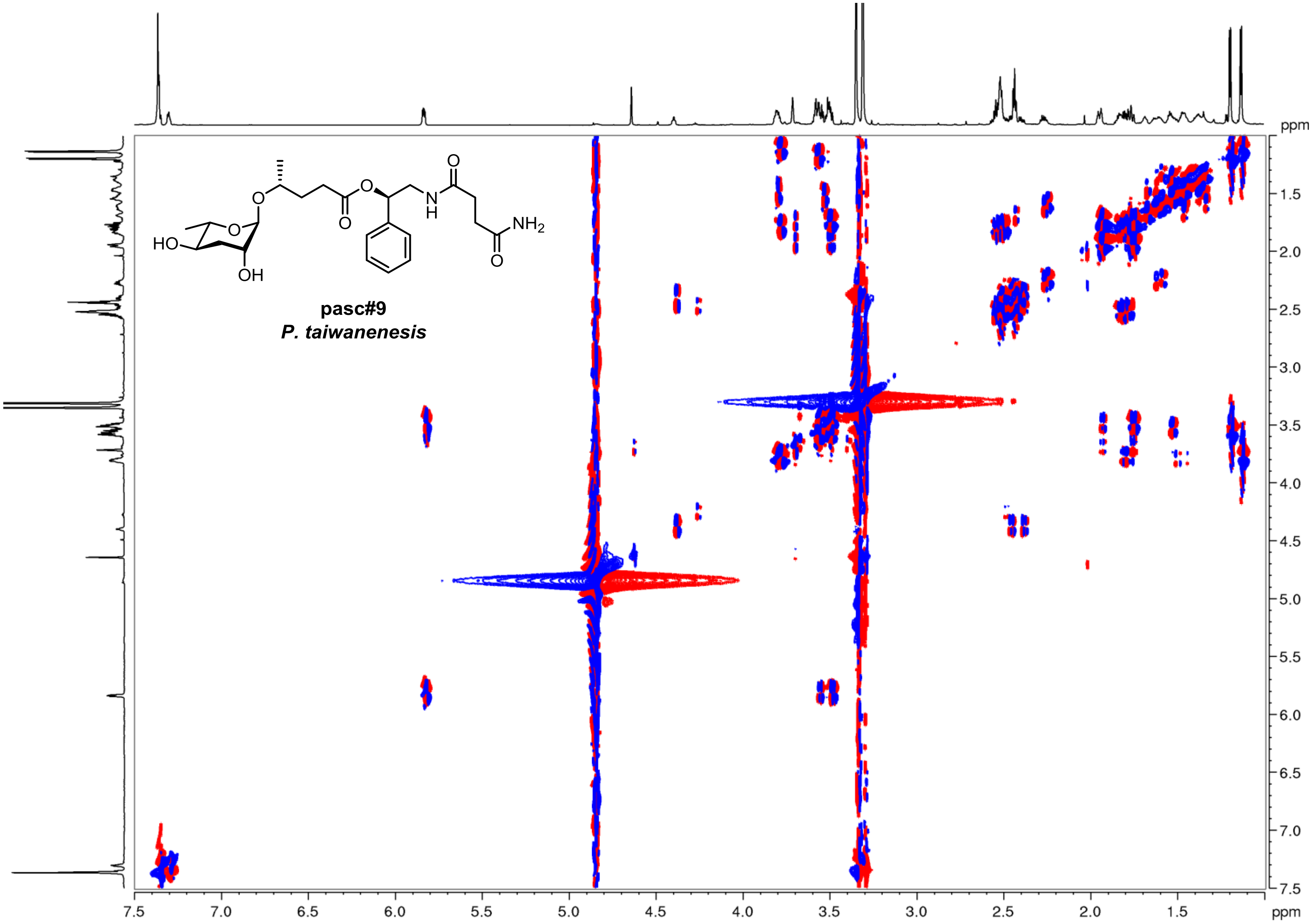

Figure 2. *dqf*-COSY spectrum of pasc#9 (800 MHz, CD_3OD) isolated from the *exo*-metabolome of *P. taiwanensis*.

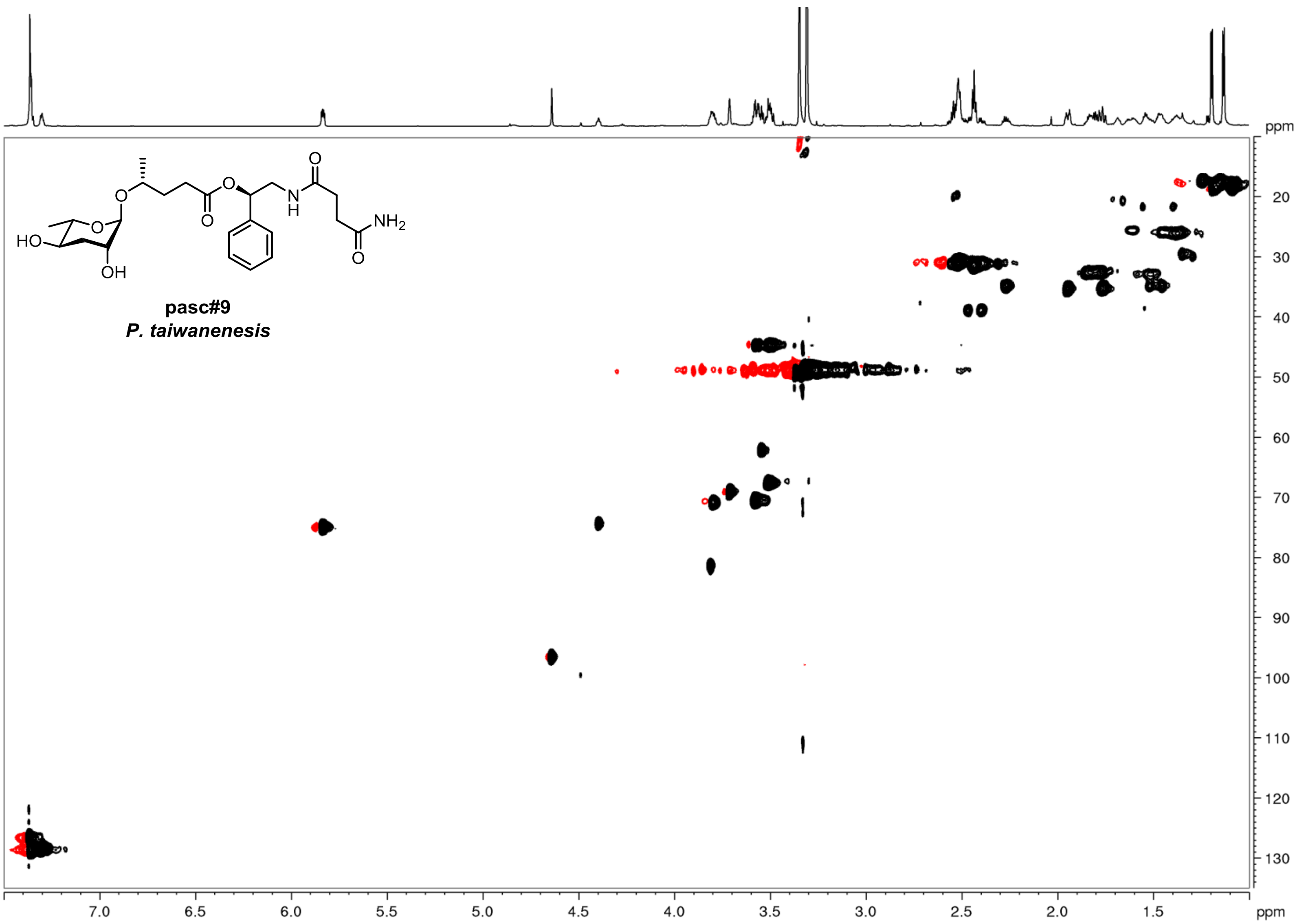

Figure 3. HSQC spectrum of pasc#9 (800 MHz, CD_3OD) isolated from the *exo*-metabolome of *P. taiwanensis*.

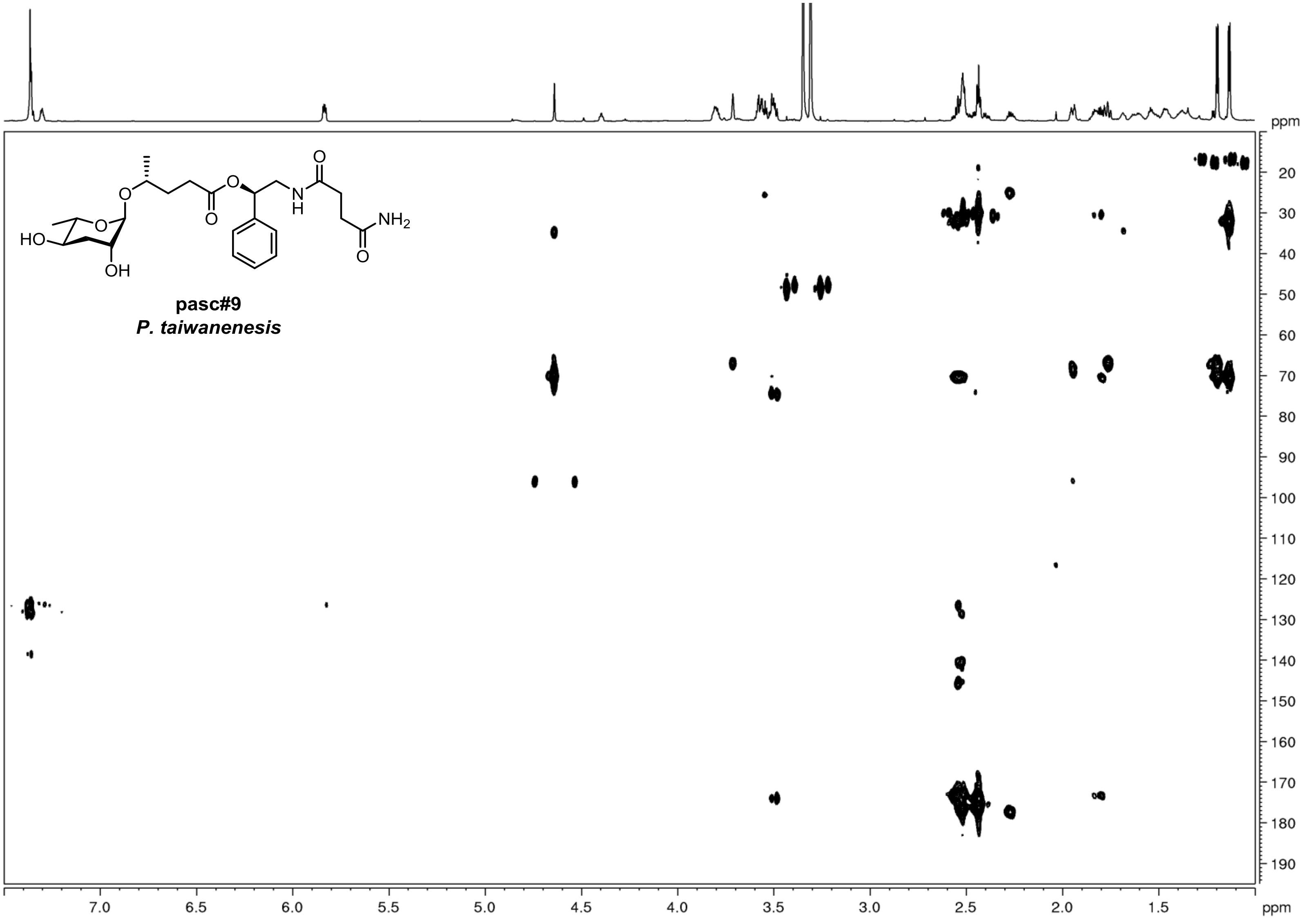

Figure 4. HMBC spectrum of pasc#9 (800 MHz, CD_3OD) isolated from the *exo*-metabolome of *P. taiwanensis*.

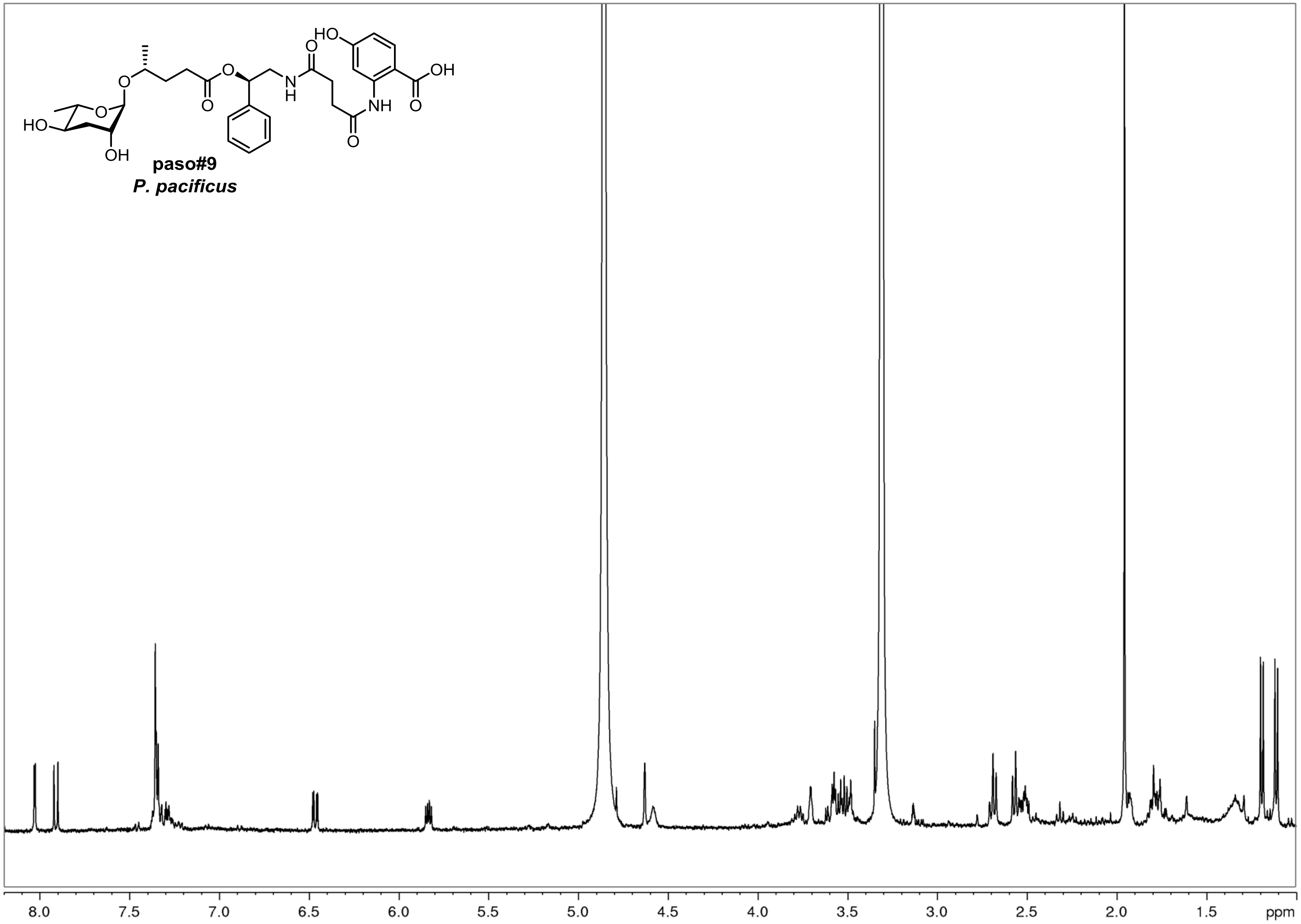

Figure 5. ^{1}H NMR spectrum of paso#9 (400 MHz, CD_3OD) isolated from the *exo*-metabolome of *P. pacificus*.

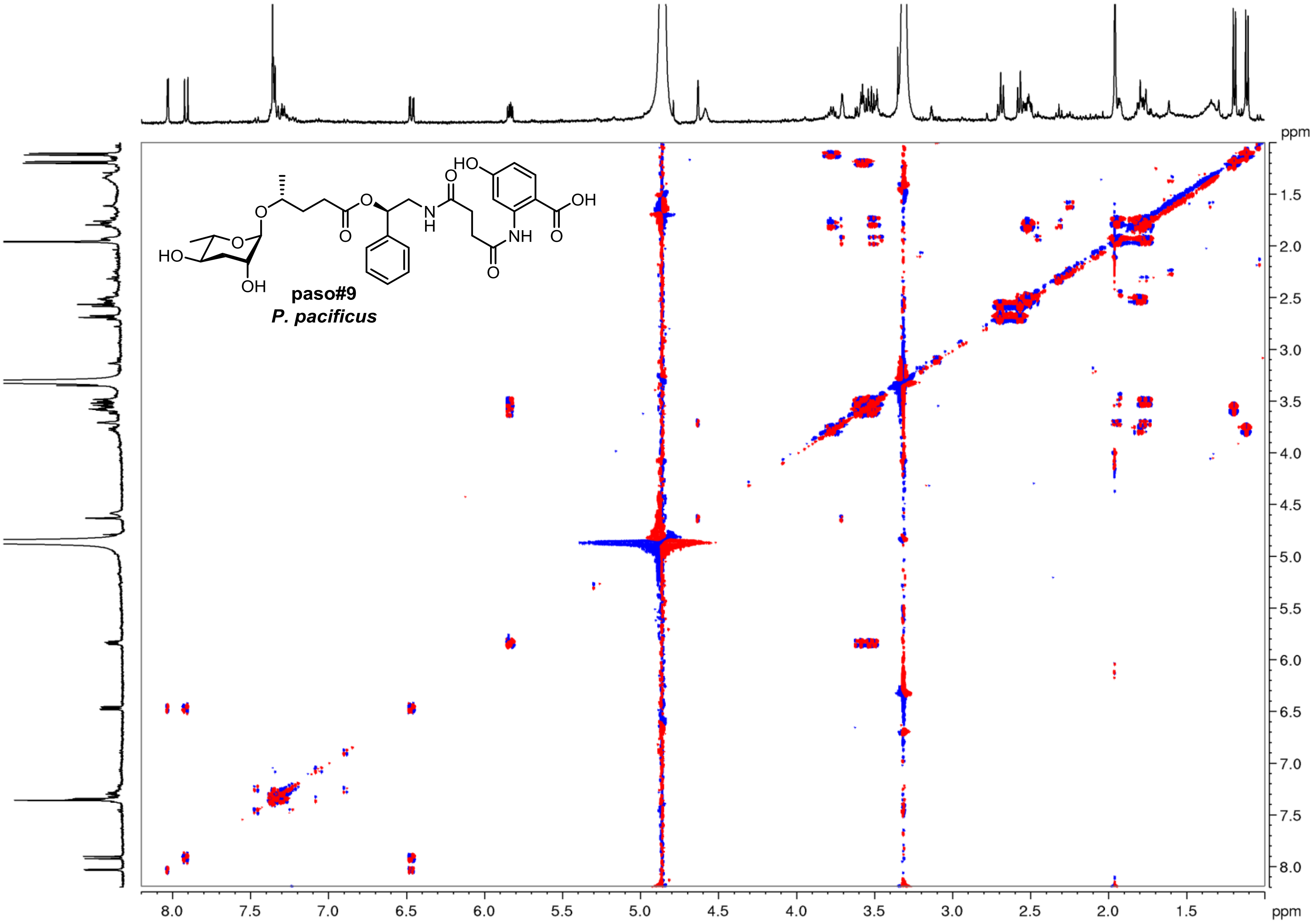

Figure 6. *dqf*-COSY spectrum of paso#9 (400 MHz, CD_3OD) isolated from the *exo*-metabolome of *P. pacificus*.

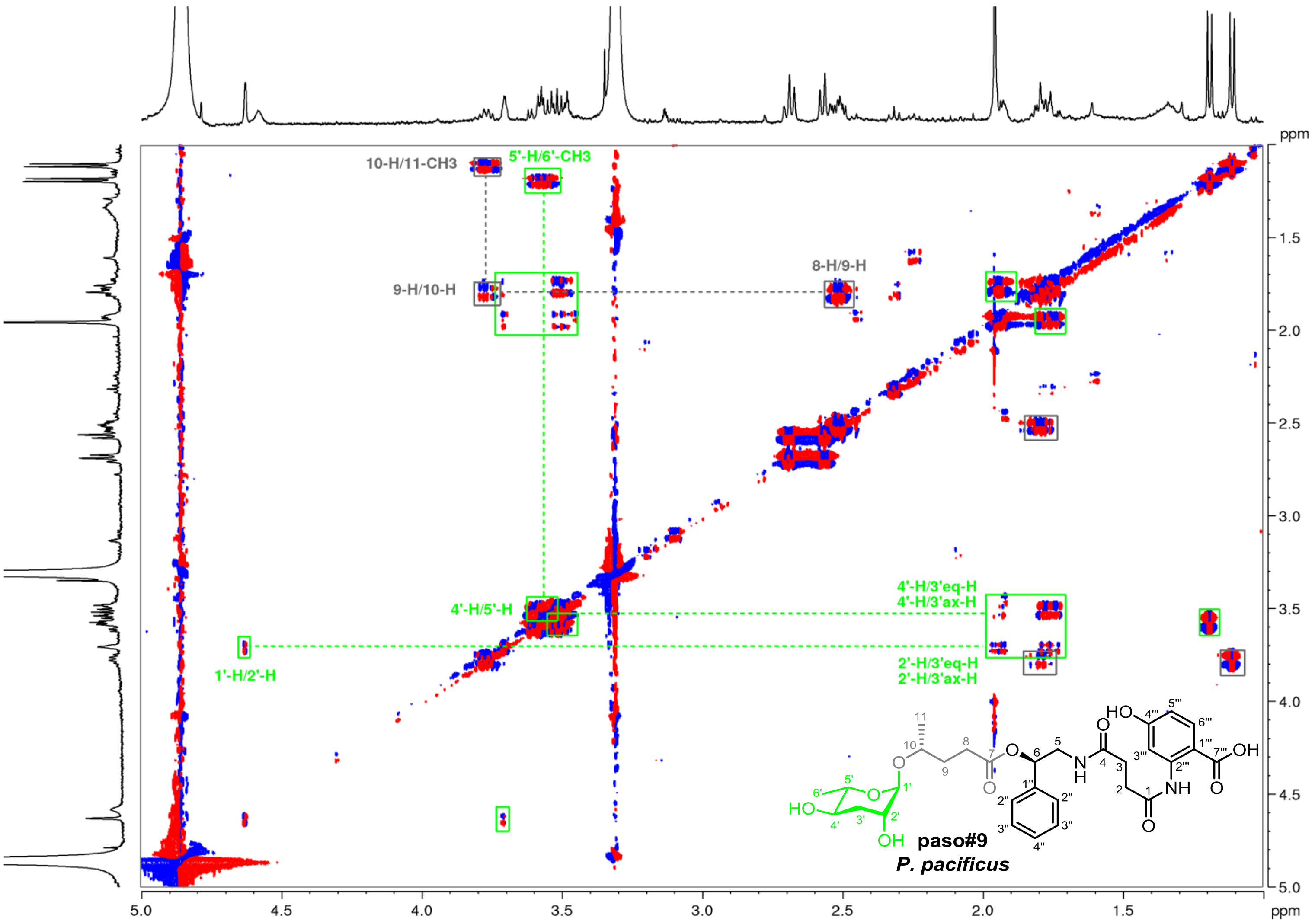

Figure 7. *dqf*-COSY spectrum (amplified region from 1.0 ppm to 5.0 ppm) of paso#9 (400 MHz, CD_3OD) isolated from *P. pacificus* highlingting the characteristic correlation signals derived from ascarylose sugar (green boxed signals) and C5 fatty acid side chain (grey boxed signals).

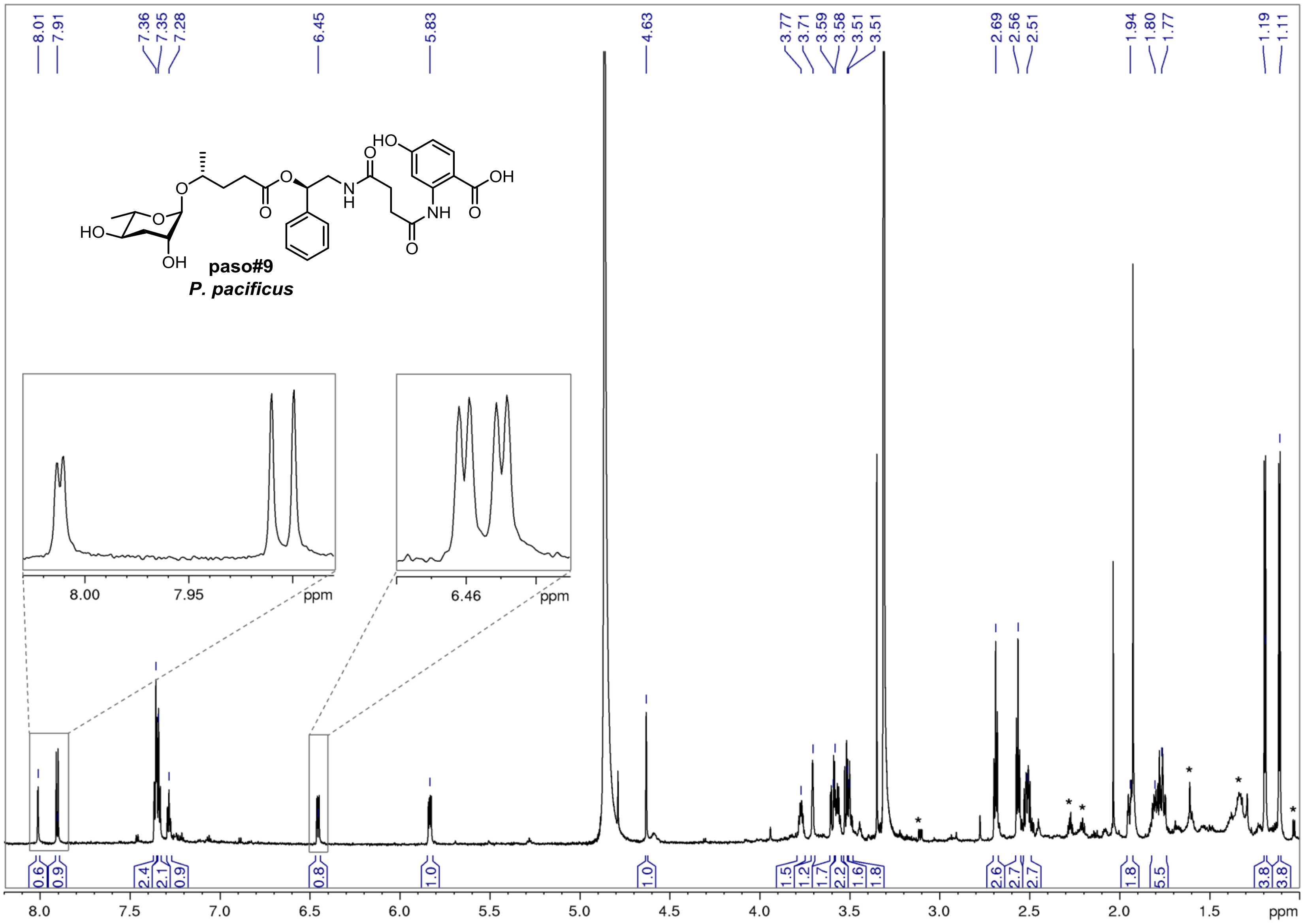

Figure 8. ^{1}H NMR spectrum of paso#9 (800 MHz, CD_3OD) isolated from the *exo*-metabolome of *P. pacificus*.

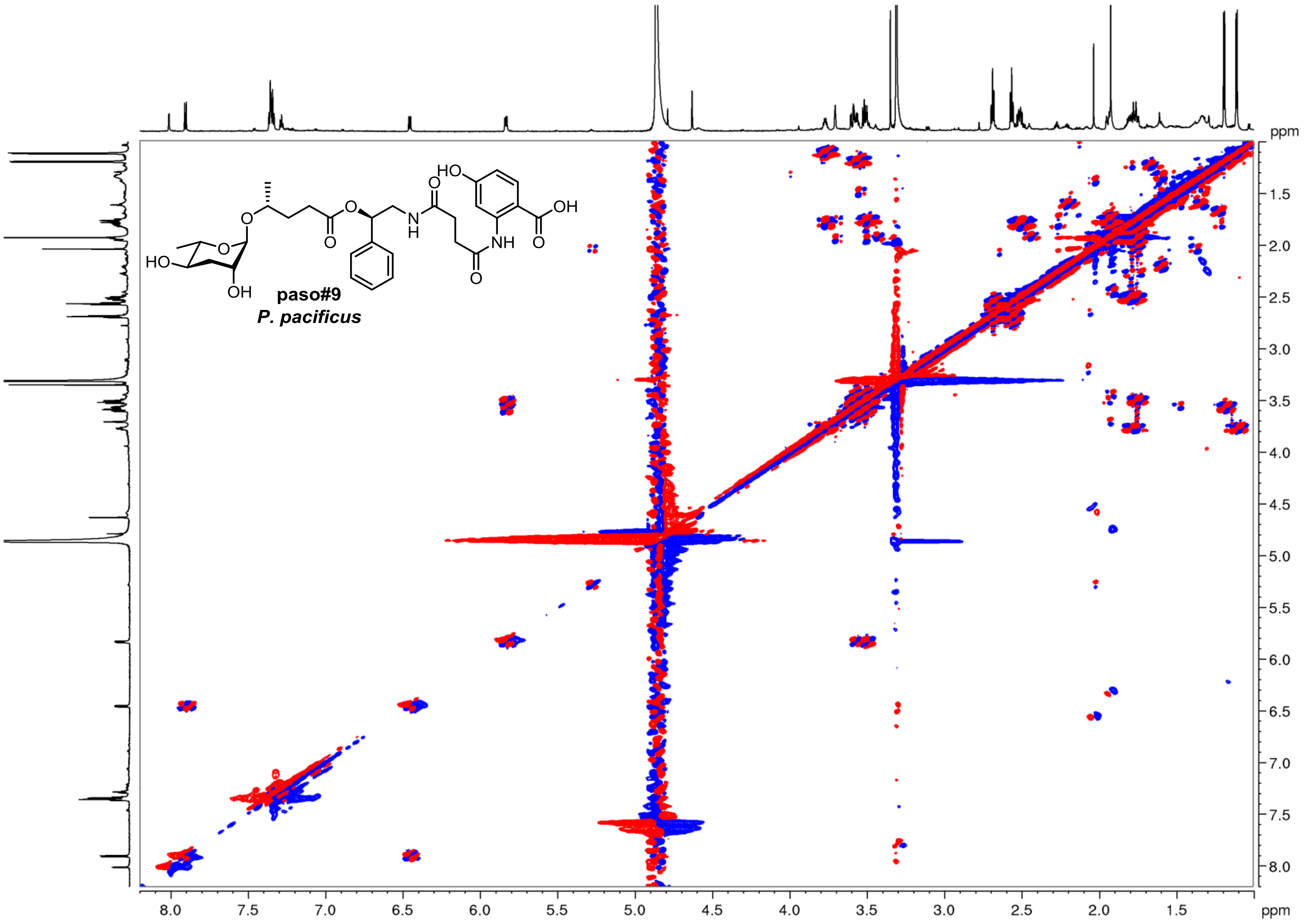

Figure 9. *dqf*-COSY spectrum of paso#9 (800 MHz, CD_3OD) isolated from the *exo*-metabolome of *P. pacificus*.

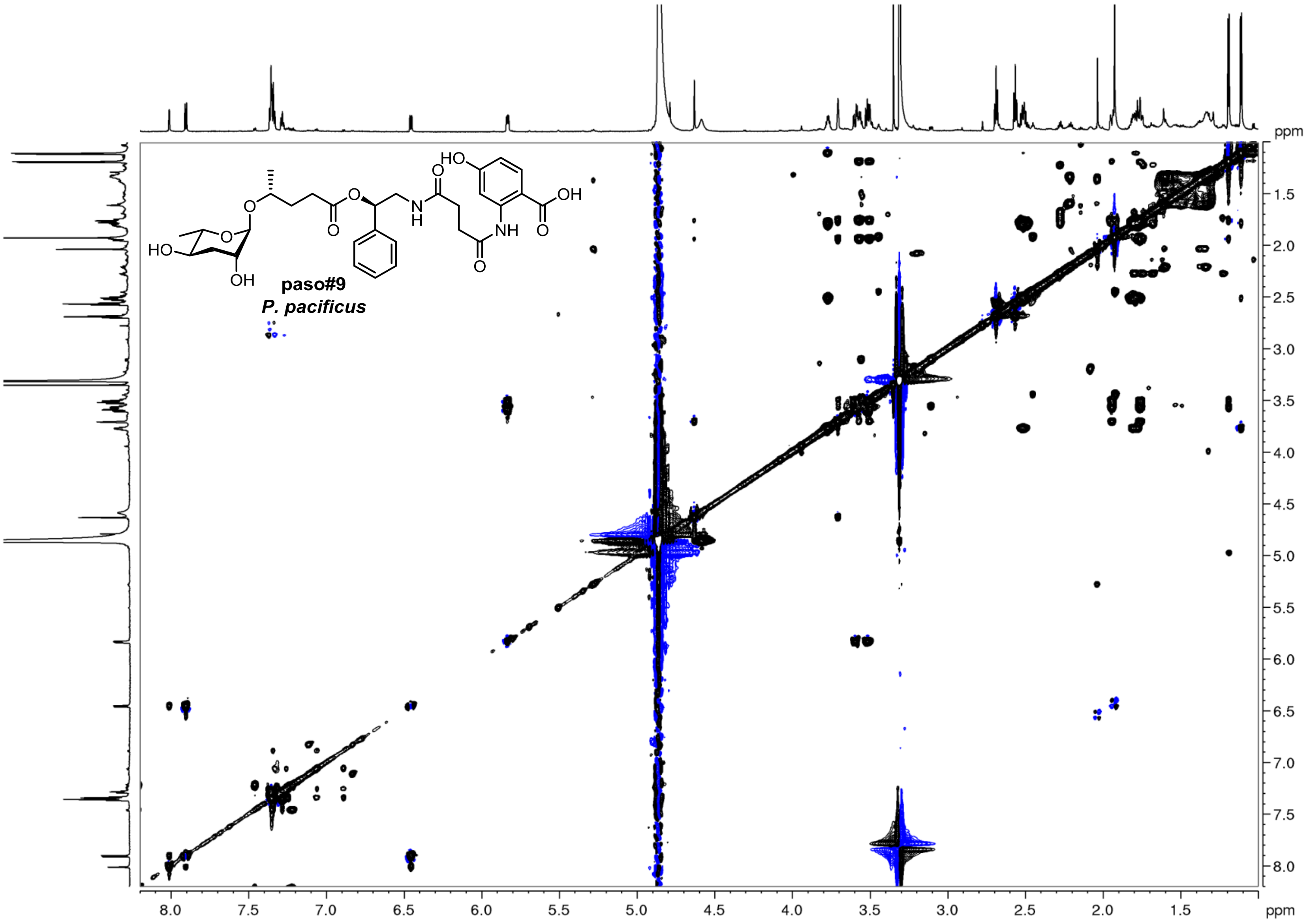

Figure 10. TOCSY spectrum of paso#9 (800 MHz, CD_3OD) isolated from the *exo*-metabolome of *P. pacificus*.

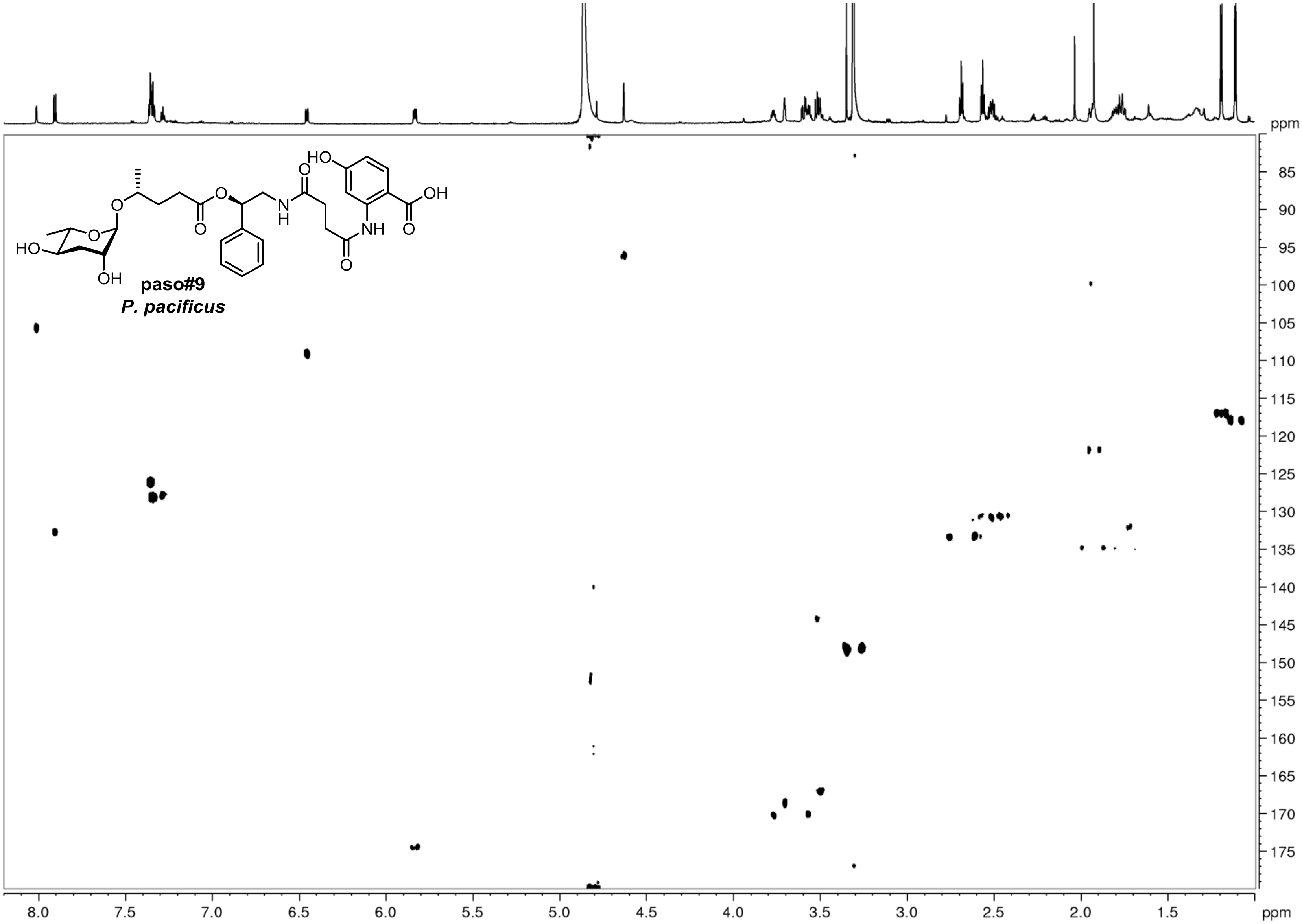

Figure 11. HSQC spectrum of paso#9 (800 MHz, CD_3OD) isolated from the *exo*-metabolome of *P. pacificus*.

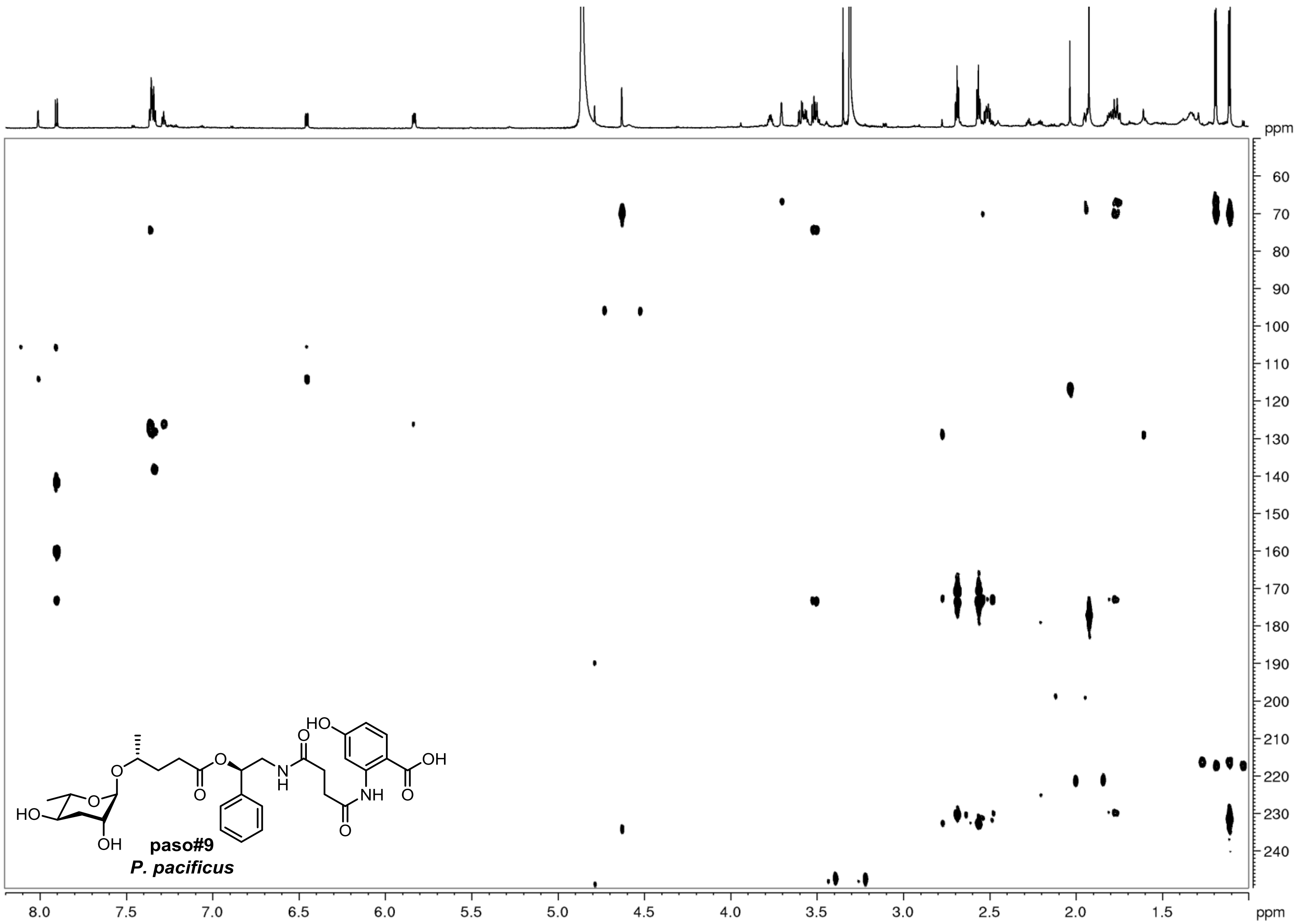

Figure 12. HMBC spectrum of paso#9 (800 MHz, CD_3OD) isolated from the *exo*-metabolome of *P. pacificus*.

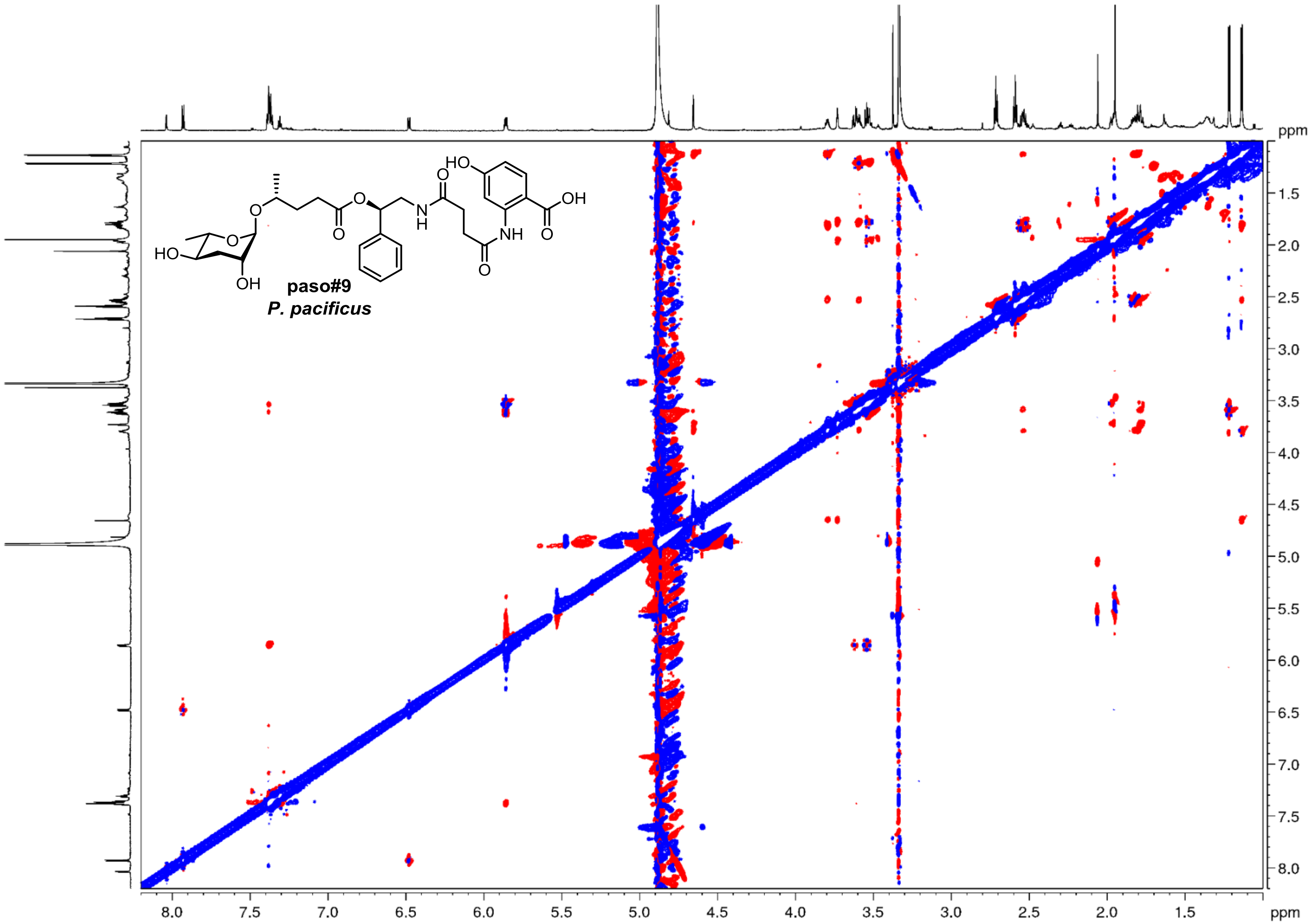

Figure 13. NOESY spectrum of paso#9 (800 MHz, CD_3OD) isolated from the *exo*-metabolome of *P. pacificus*.

supplementary file 1d: NMR spectra of UBAS chemicals Page

Figures 1-8. NMR spectra of ubas#30 (**9**) (800 MHz) isolated from *P. maxplancki*. S90

Figures 9-10. NMR spectra of ubas#1 (800 MHz) isolated from *P. pacificus (ex[eud-1])*. S98

Figure 11. Comparison of proton spectra and retention time between ubas#30 (**9**) and ubas#1. S100

Figures 12-15. NMR spectra of ubas#1 (800 MHz, CD_3OD) isolated from *P. taiwanensis*. S101

Figures 16-19. NMR spectra of ubas#1 (800 MHz, CD_3OD) isolated from *P. laevicollis*. S105

Figure 20. NMR spectra of ubas#32 (**10**) (800 MHz, CD_3OD) isolated from *P. maxplancki*. S109

Figures 21-28. NMR spectra of ubas#34 (**11**) (800 MHz) isolated from *P. quartusdecimus*. S110

Figures 29. NMR spectra of an HPLC enriched mixture of ubas#34 (**11**) and ubas#35 (**12**). S118

Figures 30-35. NMR spectra of ubas#5 (800 MHz, CD_3OD) isolated from *P. triformis*. S119

Figures 36-42. NMR spectra of ubas#28 (**13**) (800 MHz) isolated from *P. fukushimae*. S125

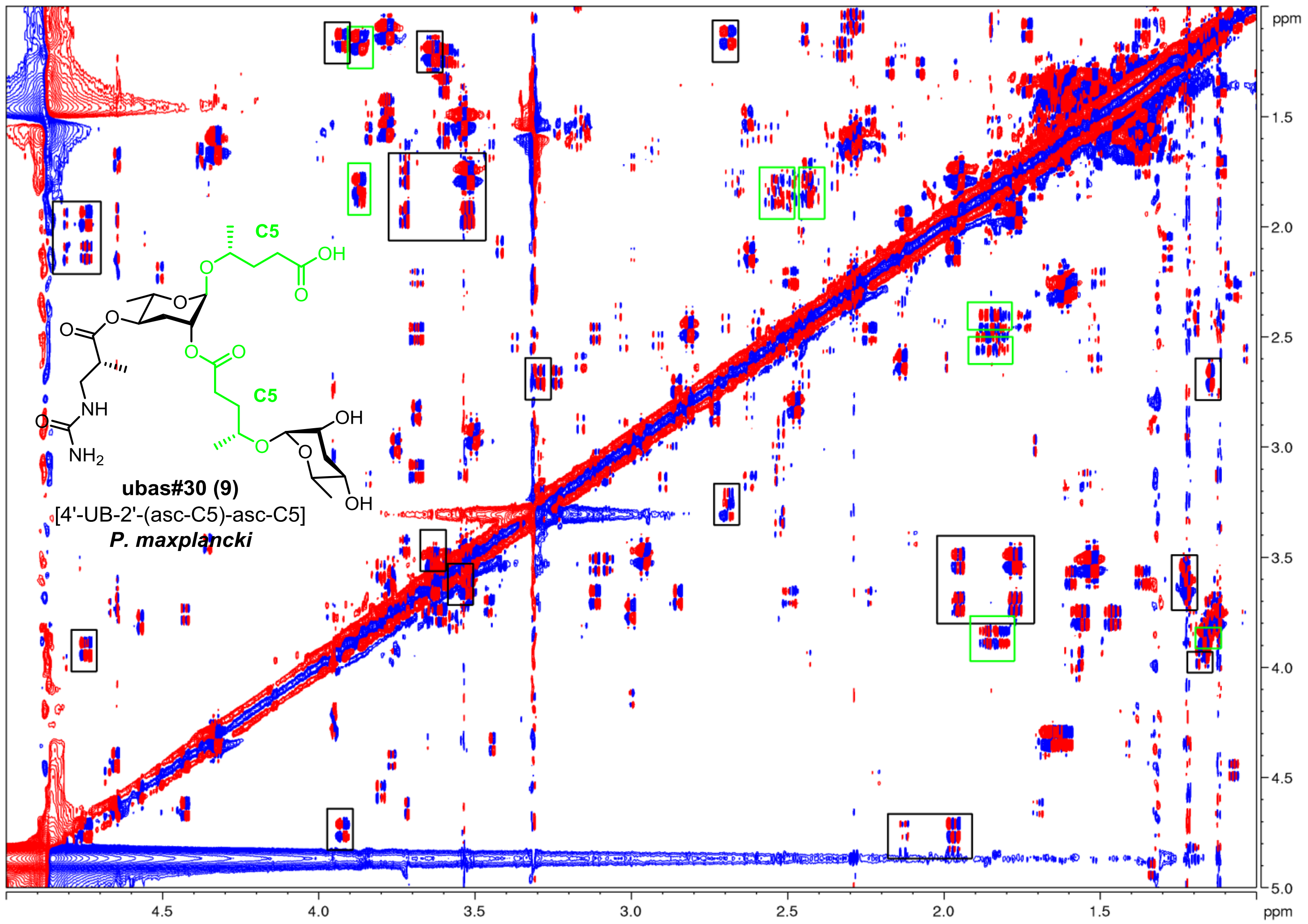

Figure 1. *dqf*-COSY spectrum (800 MHz, CD_3OD) of an enriched SPE fraction containing ubas#30 [4'-UB-2'-(asc-C5)-asc-C5, **9**] derived from the separation of a 2 l liquid culture of *P. maxplancki*. Black color boxed cross peaks suggest that ubas#30 (**9**) contains a ureidoisobutyric acid building block and one free ascarylose sugar, as well as one 2',4'-substituted ascarylose sugar. Green color boxed cross peaks suggest that ubas#30 (**9**) contains two ω-1 style C5 fatty acid side chains. This is quite different from ubas#1 that is known to contain a ω style C5 fatty acid side chain in the first ascaroside unit.

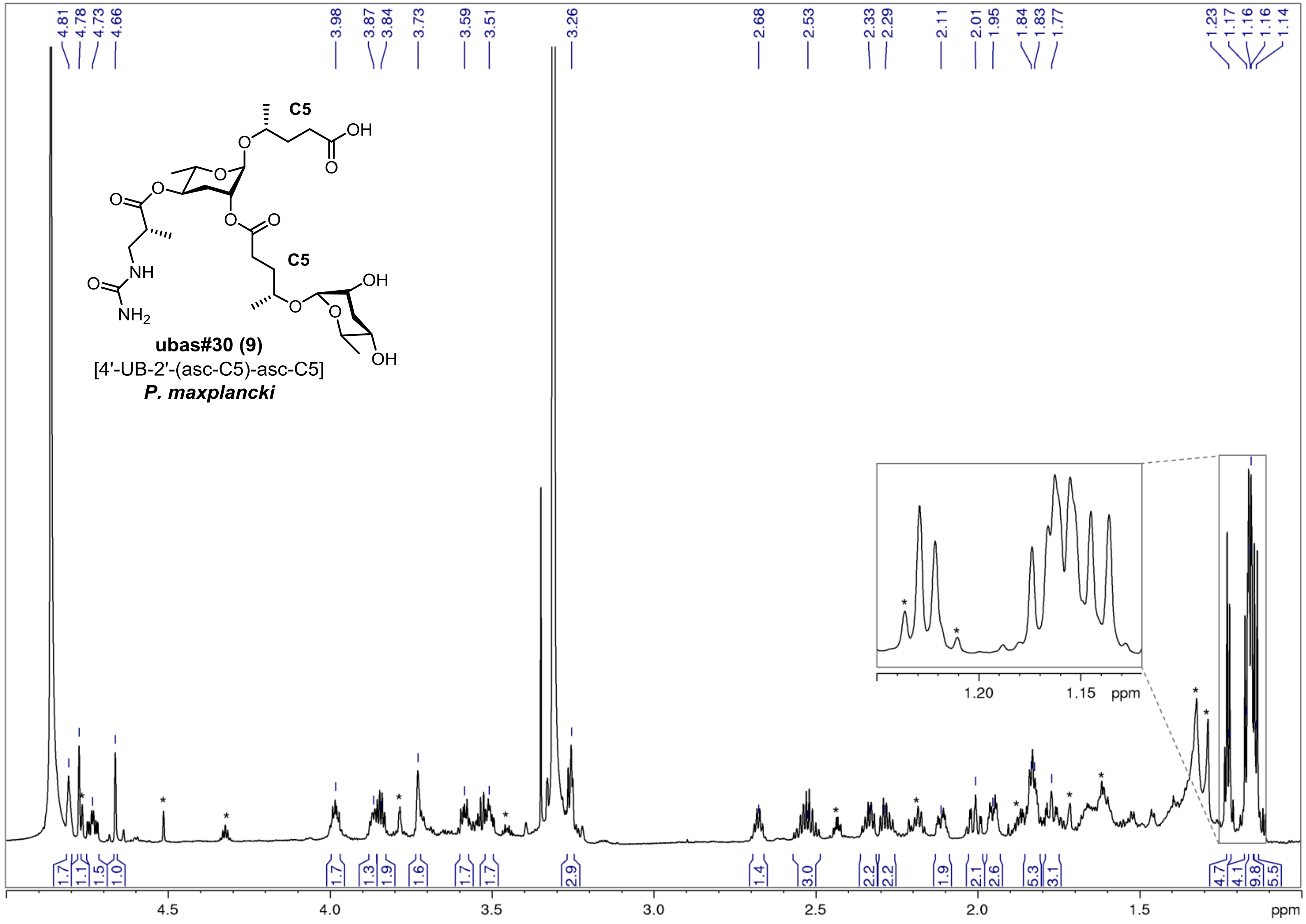

Figure 2. ^{1}H NMR spectrum of ubas#30 [4'-UB-2'-(asc-C5)-asc-C5, **9**] (800 MHz, CD_3OD) isolated from the *exo*-metabolome of *P. maxplancki*. Asterisks marked peaks are derived from impurities.

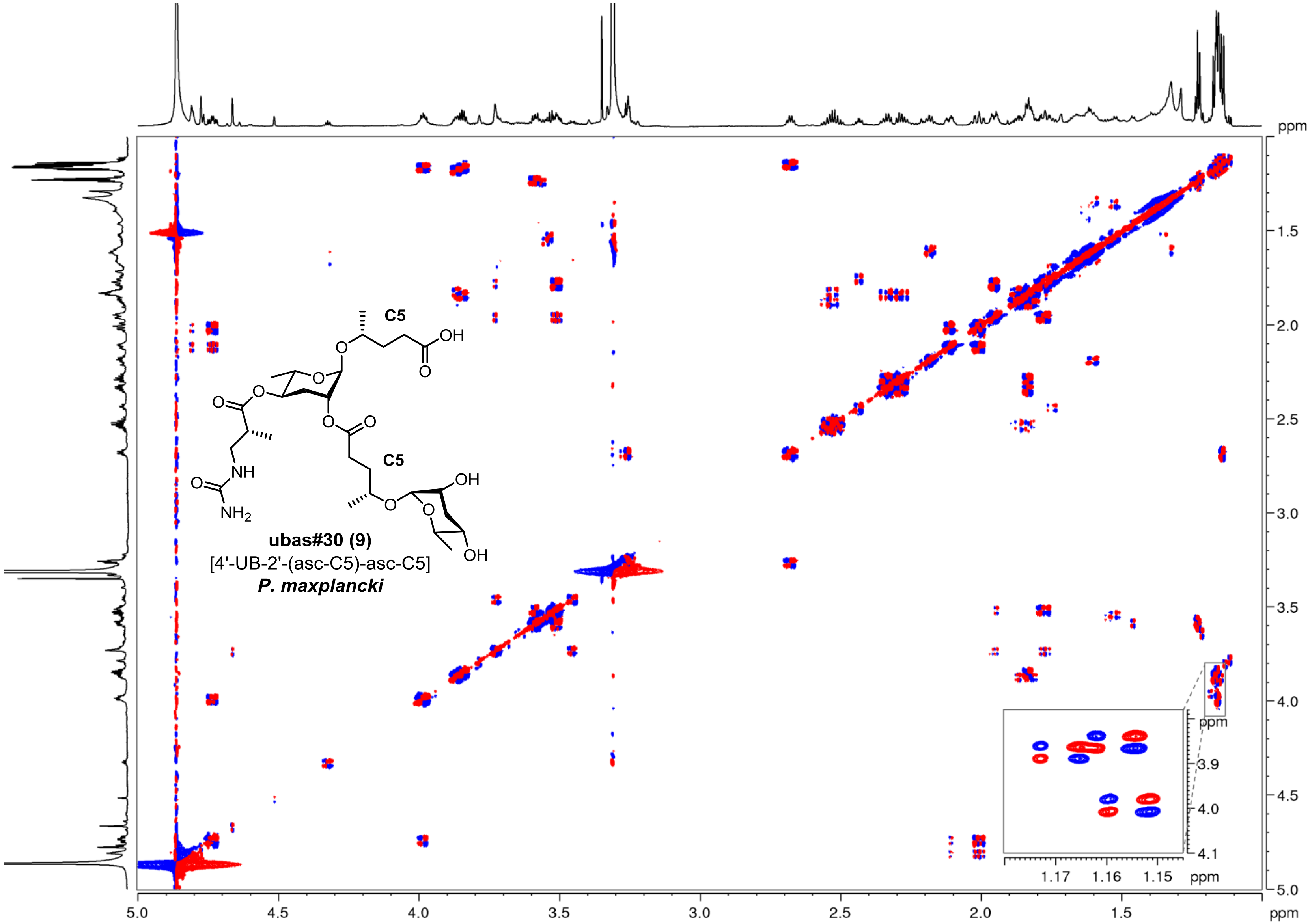

Figure 3. *dqf*-COSY spectrum of ubas#30 [4'-UB-2'-(asc-C5)-asc-C5, **9**] (800 MHz, CD_3OD) isolated from the *exo*-metabolome of *P. maxplancki*.

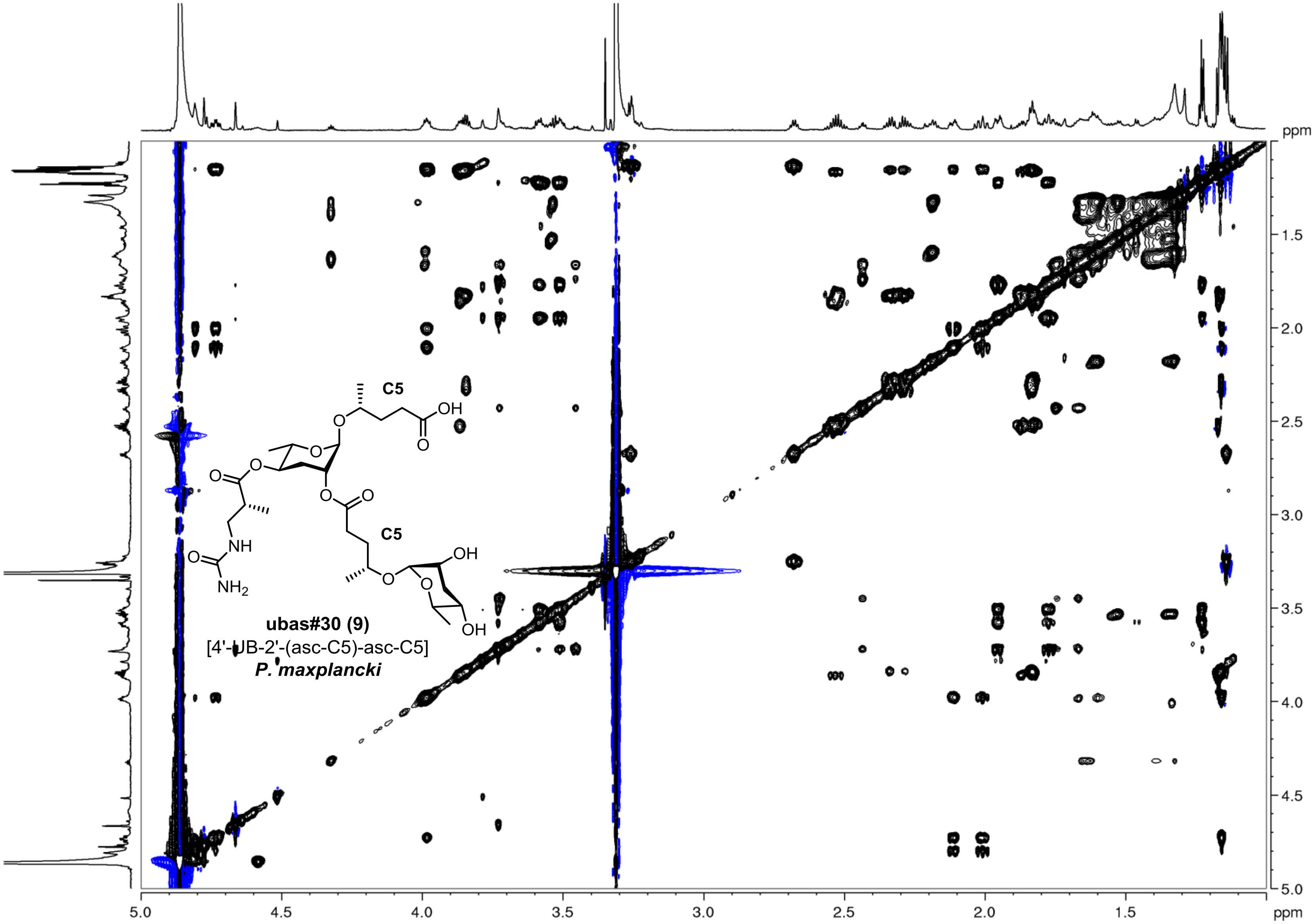

Figure 4. TOCSY spectrum of ubas#30 [4'-UB-2'-(asc-C5)-asc-C5, **9**] (800 MHz, CD_3OD) isolated from the *exo*-metabolome of *P. maxplancki*.

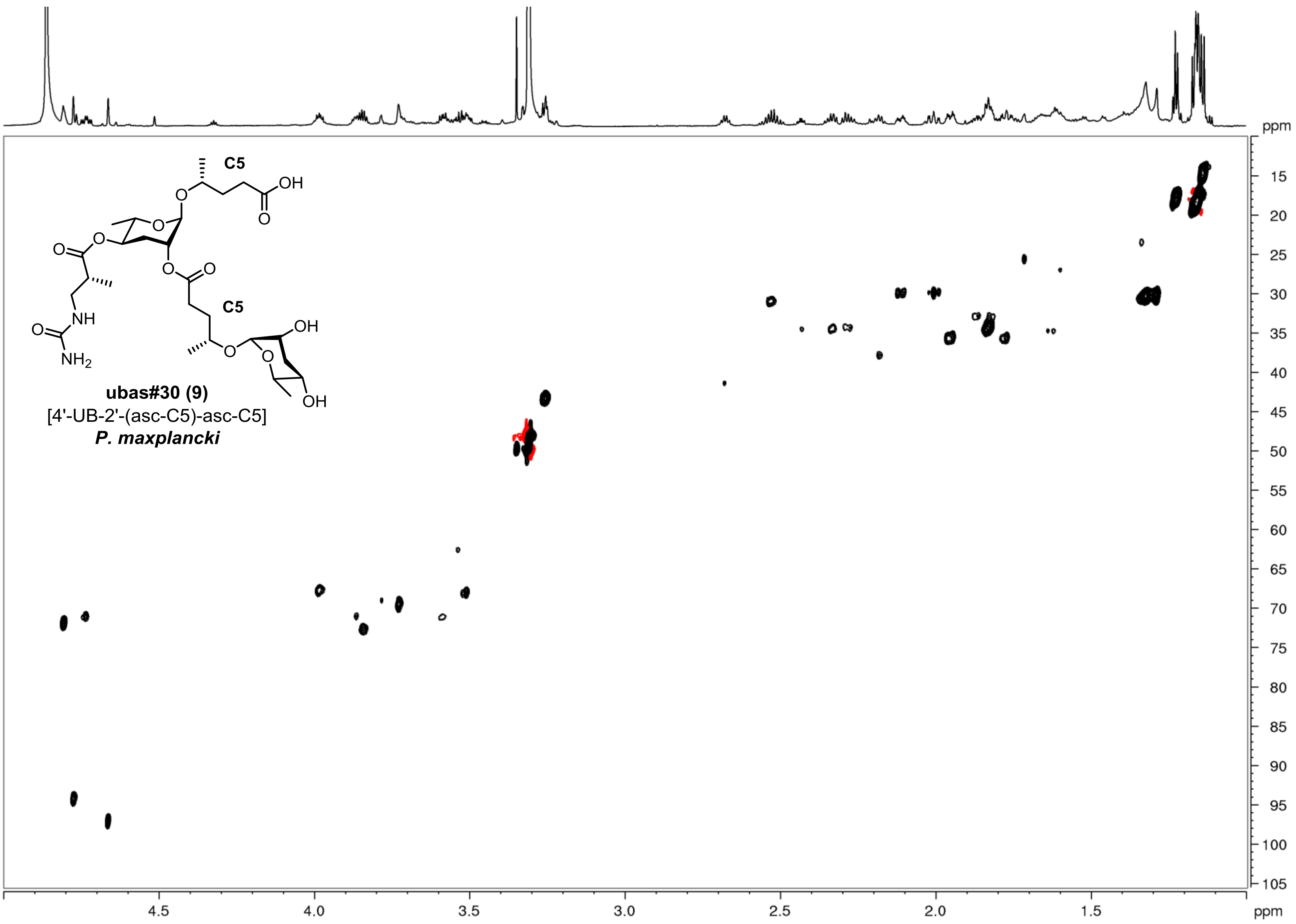

Figure 5. HSQC spectrum of ubas#30 [4'-UB-2'-(asc-C5)-asc-C5, **9**] (800 MHz, CD_3OD) isolated from the *exo*-metabolome of *P. maxplancki*.

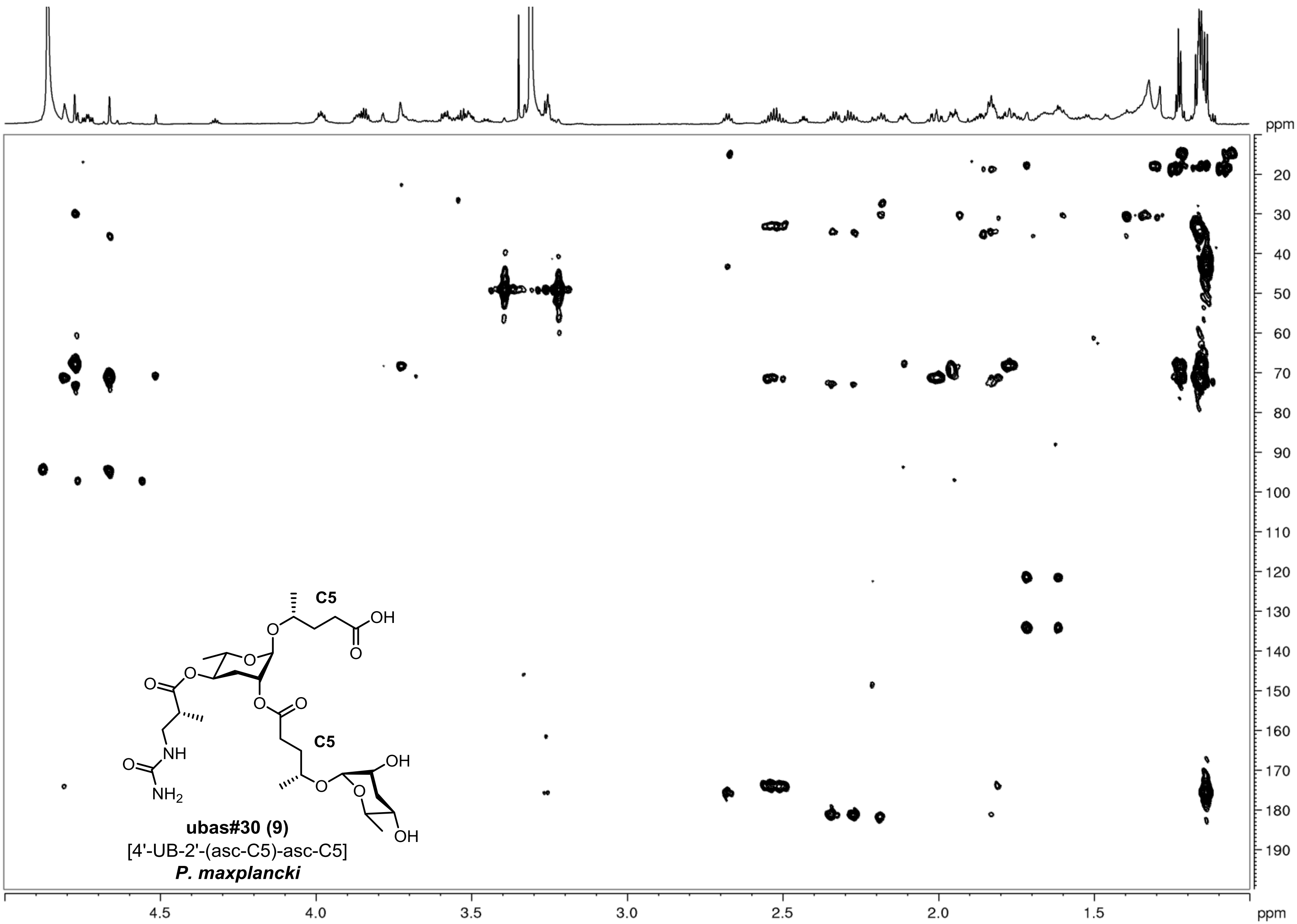

Figure 6. HMBC spectrum of ubas#30 [4'-UB-2'-(asc-C5)-asc-C5, **9**] (800 MHz, CD_3OD) isolated from the *exo*-metabolome of *P. maxplancki*.

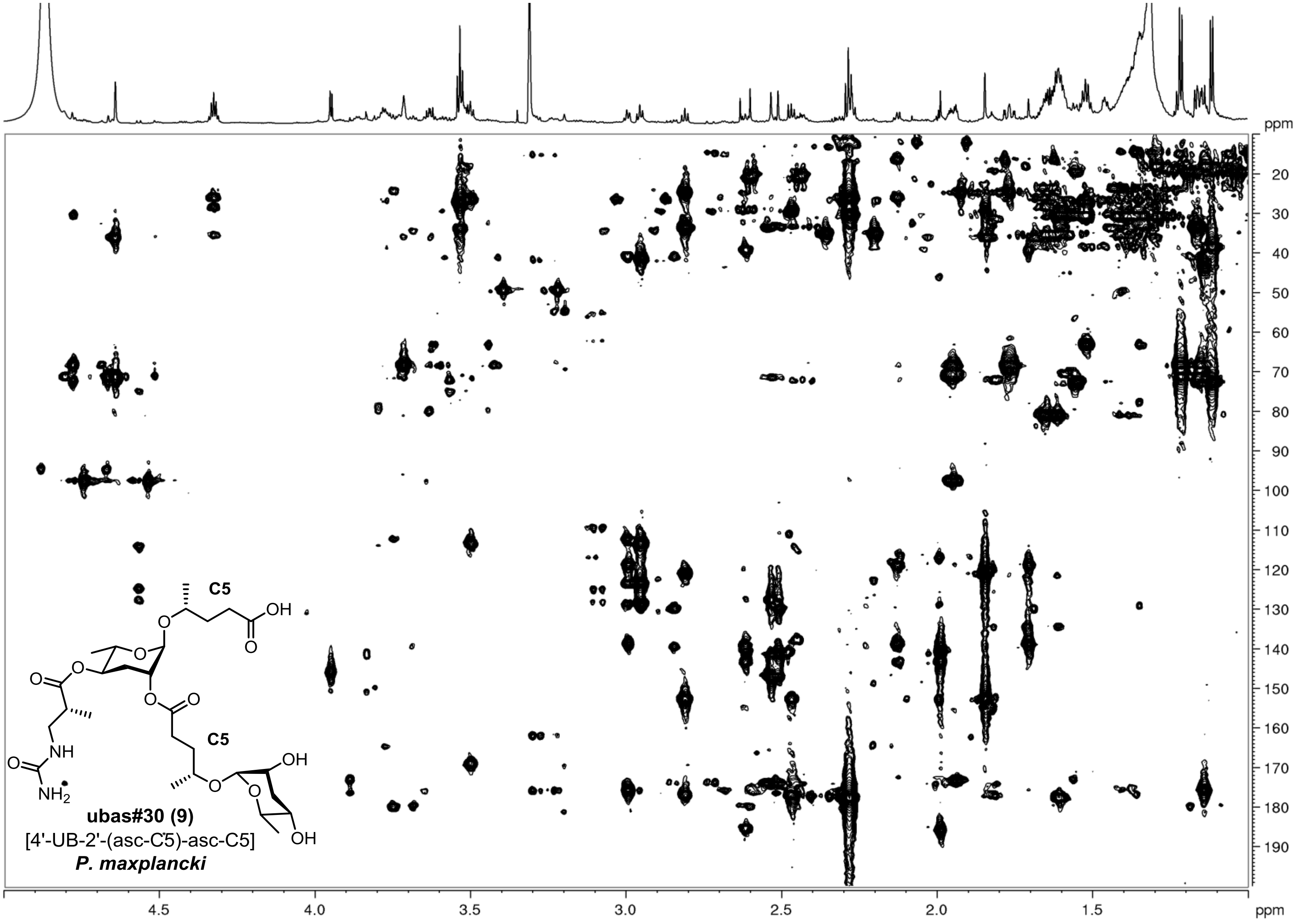

Figure 7. HMBC spectrum of a SPE fraction containing ubas#30 [4'-UB-2'-(asc-C5)-asc-C5, **9**] (800 MHz, CD_3OD) derived from the *exo*-metabolome of *P. maxplancki*.

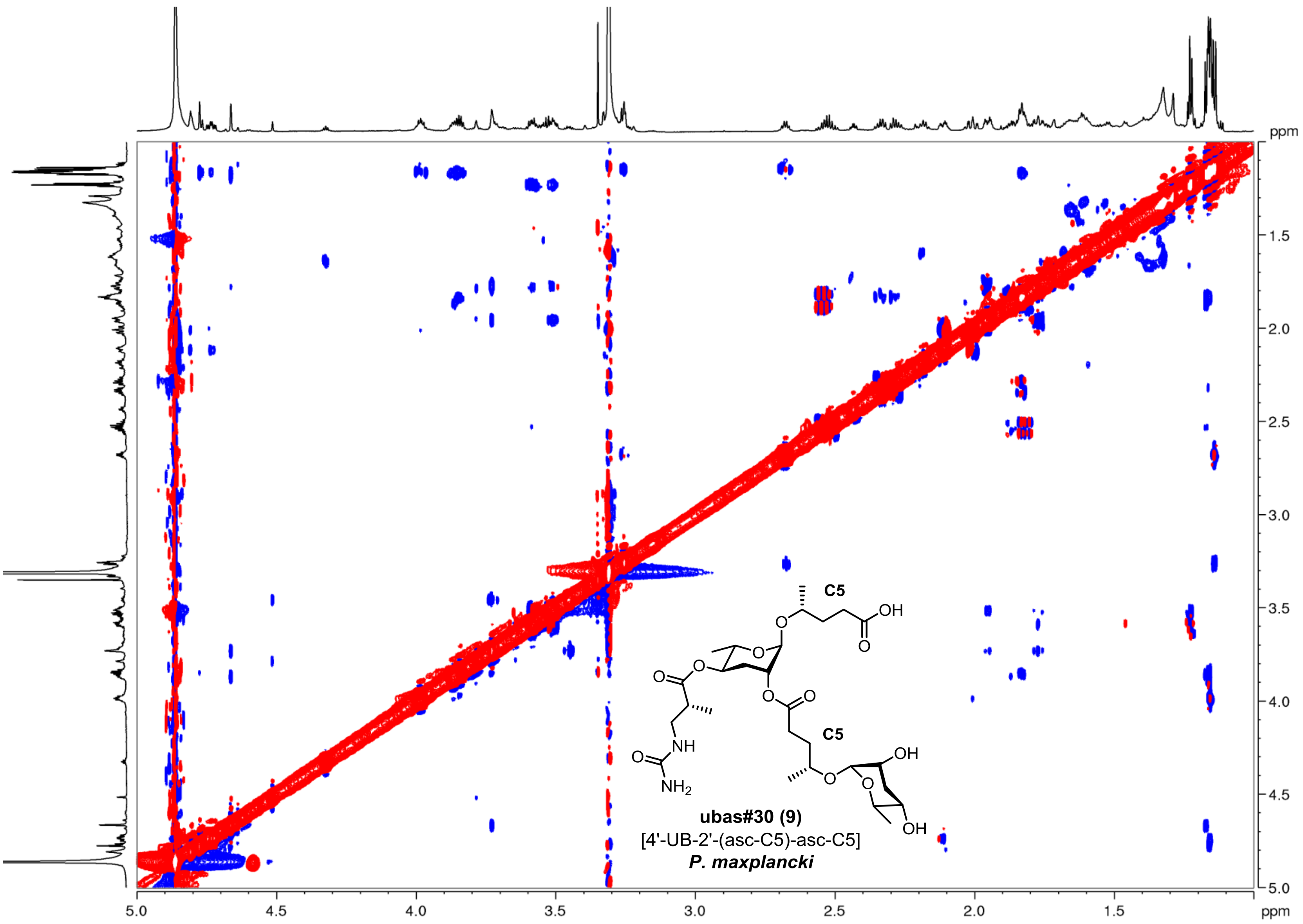

Figure 8. NOESY spectrum of ubas#30 [4'-UB-2'-(asc-C5)-asc-C5, **9**] (800 MHz, CD_3OD) isolated from the *exo*-metabolome of *P. maxplancki*.

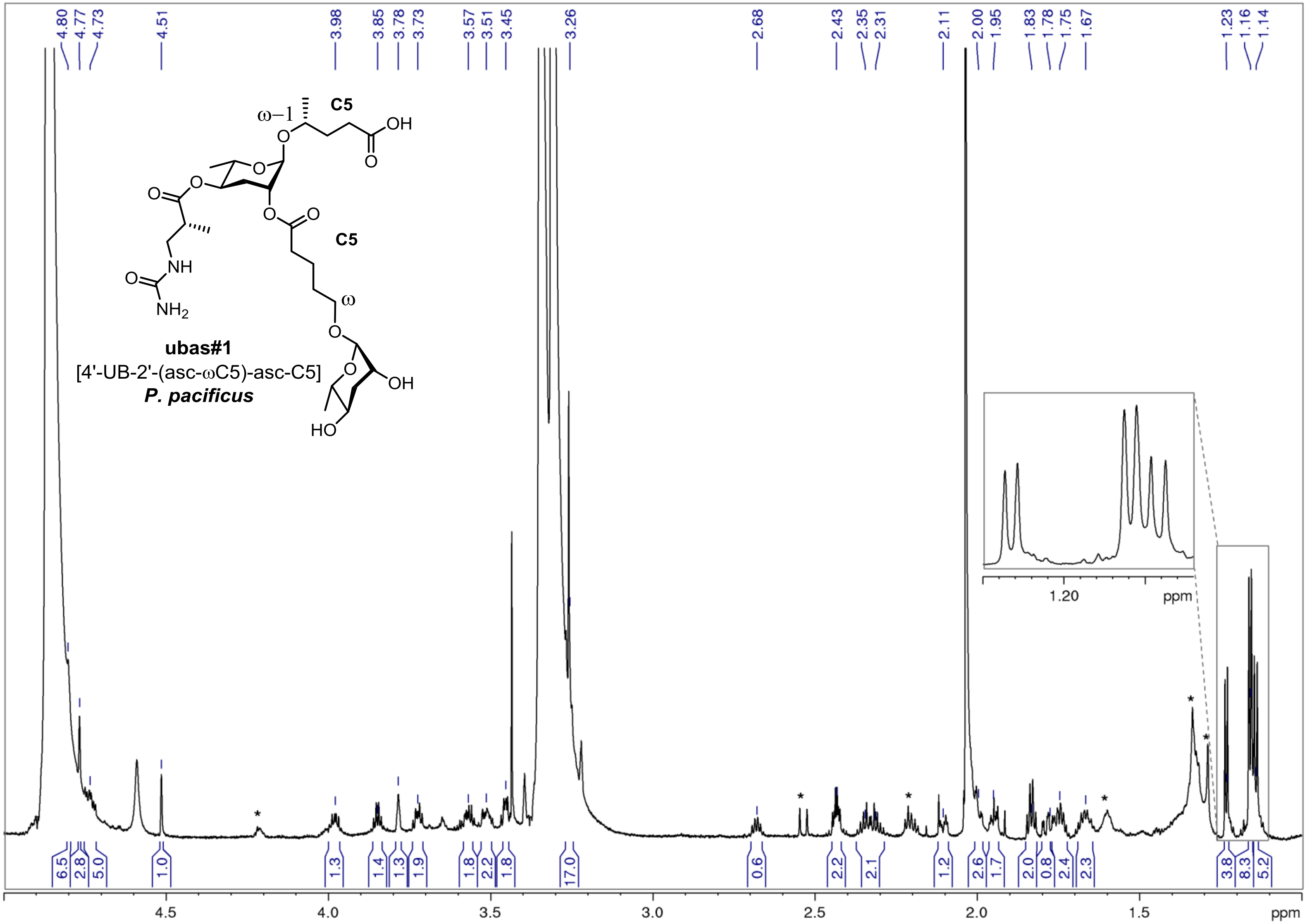

Figure 9. ^{1}H NMR spectrum of ubas#1 [4'-UB-2'-(asc-ωC5)-asc-C5] (800 MHz, CD_3OD) isolated from a mutant strain *Ex[eud-1] exo*-metabolome. Asterisks marked peaks are impurities.

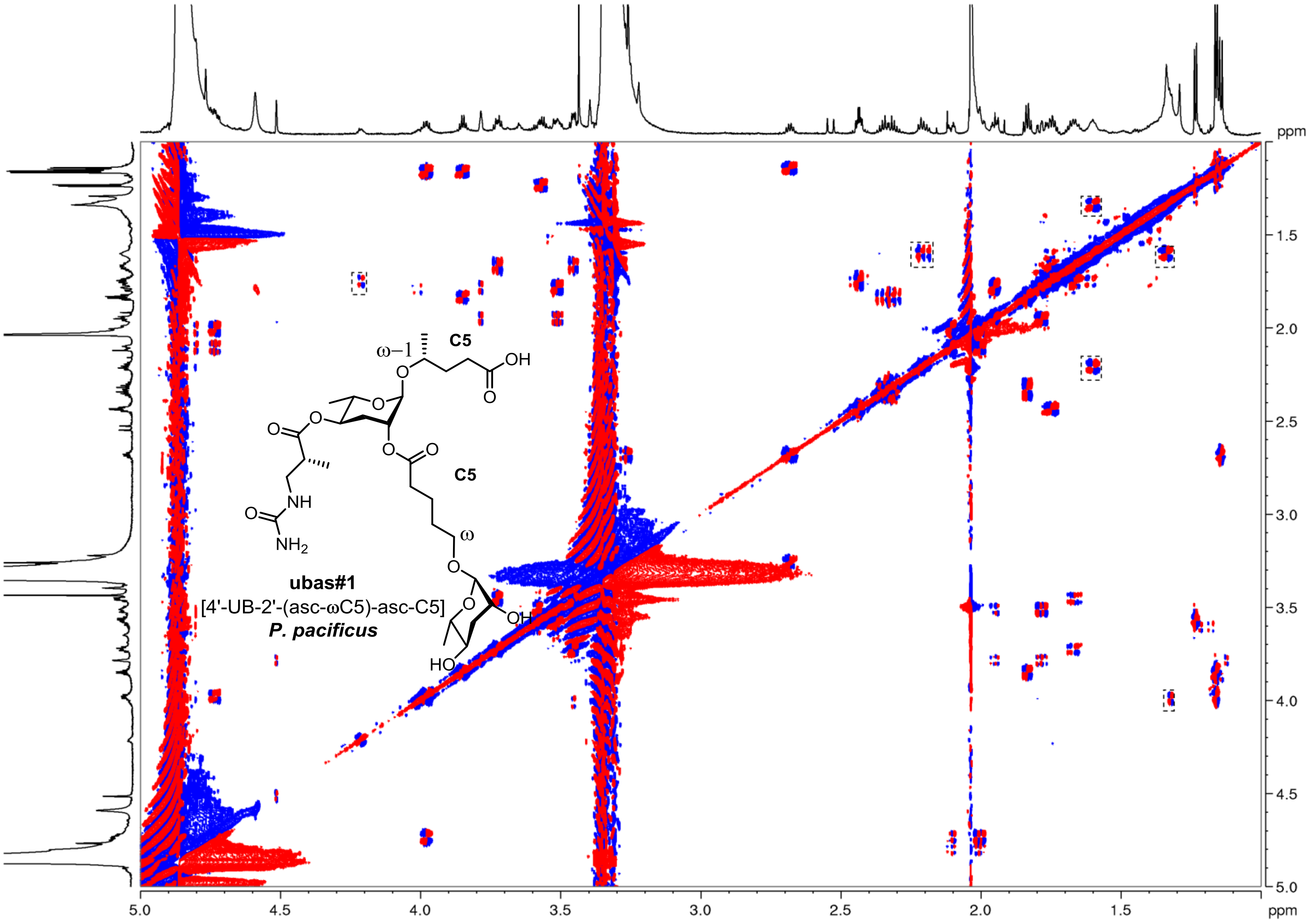

Figure 10. *dqf*-COSY spectrum of ubas#1 [4'-UB-2'-(asc-ωC5)-asc-C5] (800 MHz, CD_3OD) isolated from the *exo*-metabolome of *Ex[eud-1]* worms. Dashed line boxed signals are derived from impurities.

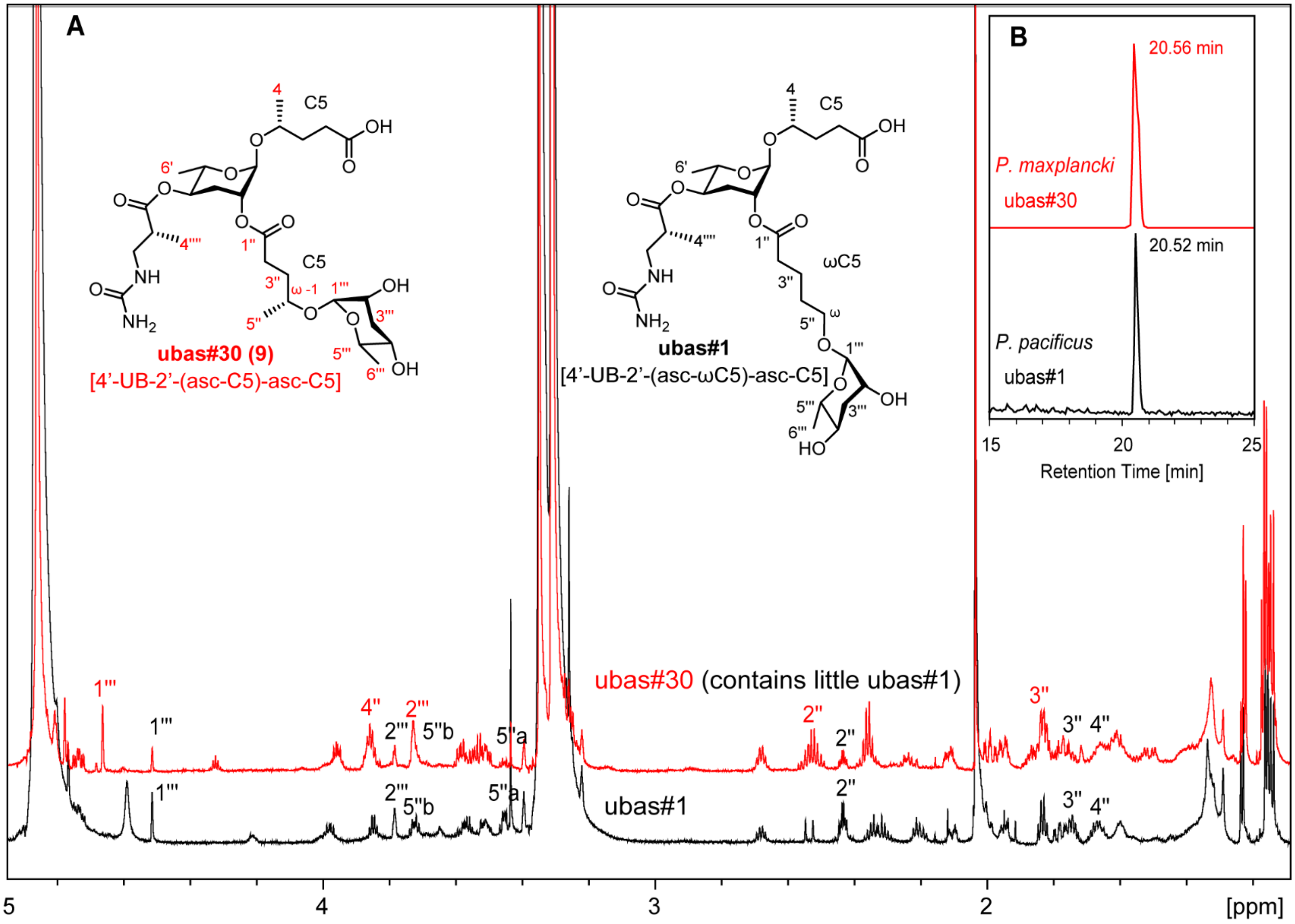

Figure 11. Comparison of proton spectra (**A**) and HPLC retention times (**B**) between ubas#30 [4'-UB-2'-(asc-C5)-asc-C5, **9**] and ubas#1 [4'-UB-2'-(asc-ωC5)-asc-C5]. ubas#30 (**9**) containing two ascr#9 [asc-C5] units is different from ubas#1 harboring one ascr#9 [asc-C5] unit and one oscr#9 [asc-ωC5] unit. Detailed analysis of the proton spectrum of ubas#30 (**9**) isolated from *P. maxplancki* shows that the enriched ubas#30 (**9**) sample contains small amounts of ubas#1, demonstrating that *P. maxplancki* produces a mixture of dominating ubas#30 (**9**) and minor ubas#1.

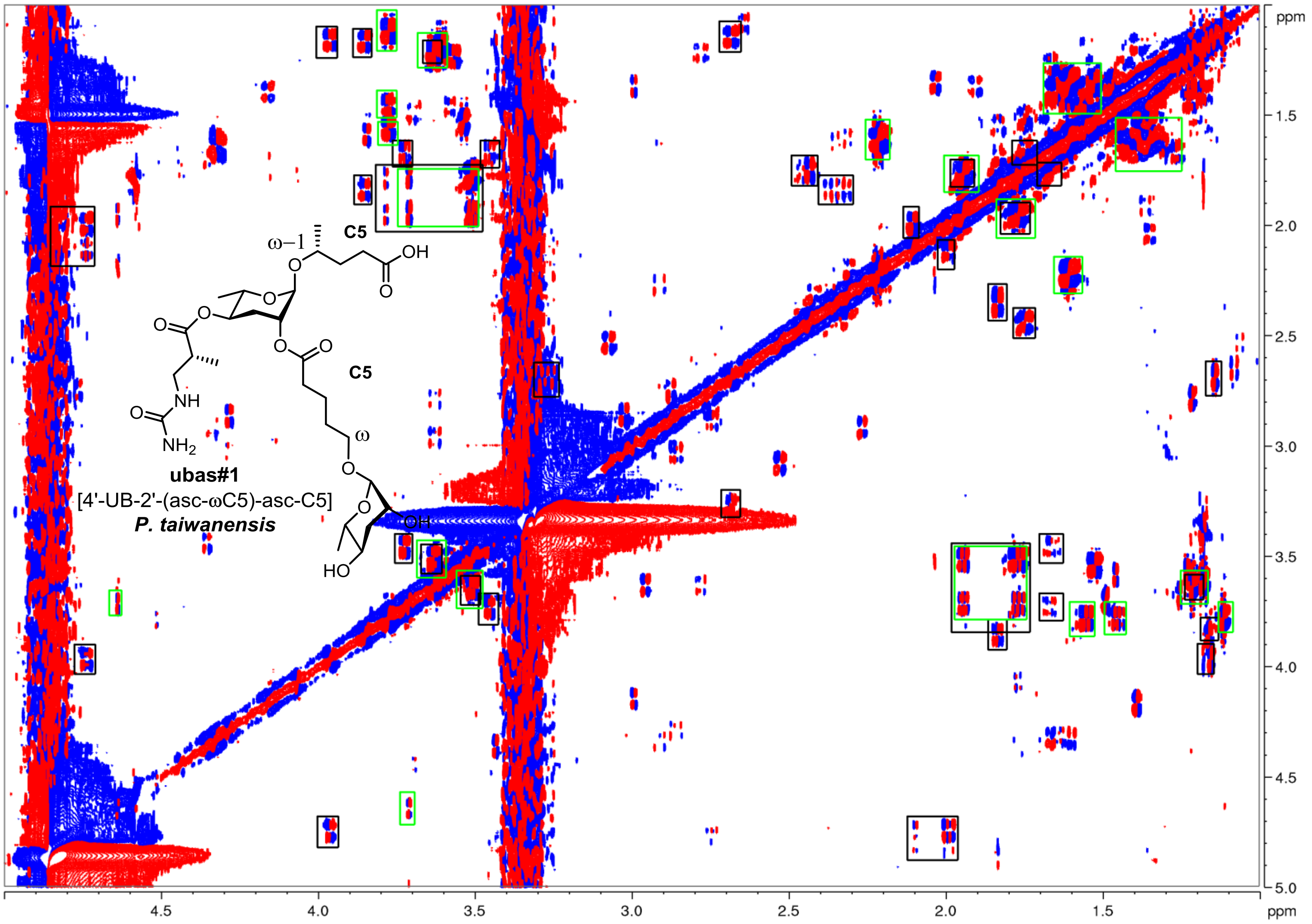

Figure 12. *dqf*-COSY spectrum of HPLC enriched sample containing ubas#1 [4'-UB-2'-(asc-ωC5)-asc-C5] (800 MHz, CD_3OD) isolated from the *exo*-metabolome of *P. taiwanensis*. Green line boxed signals are derived from ascr#10 [asc-C9] which was co-isolated with ubas#1 due to their close HPLC retention times.

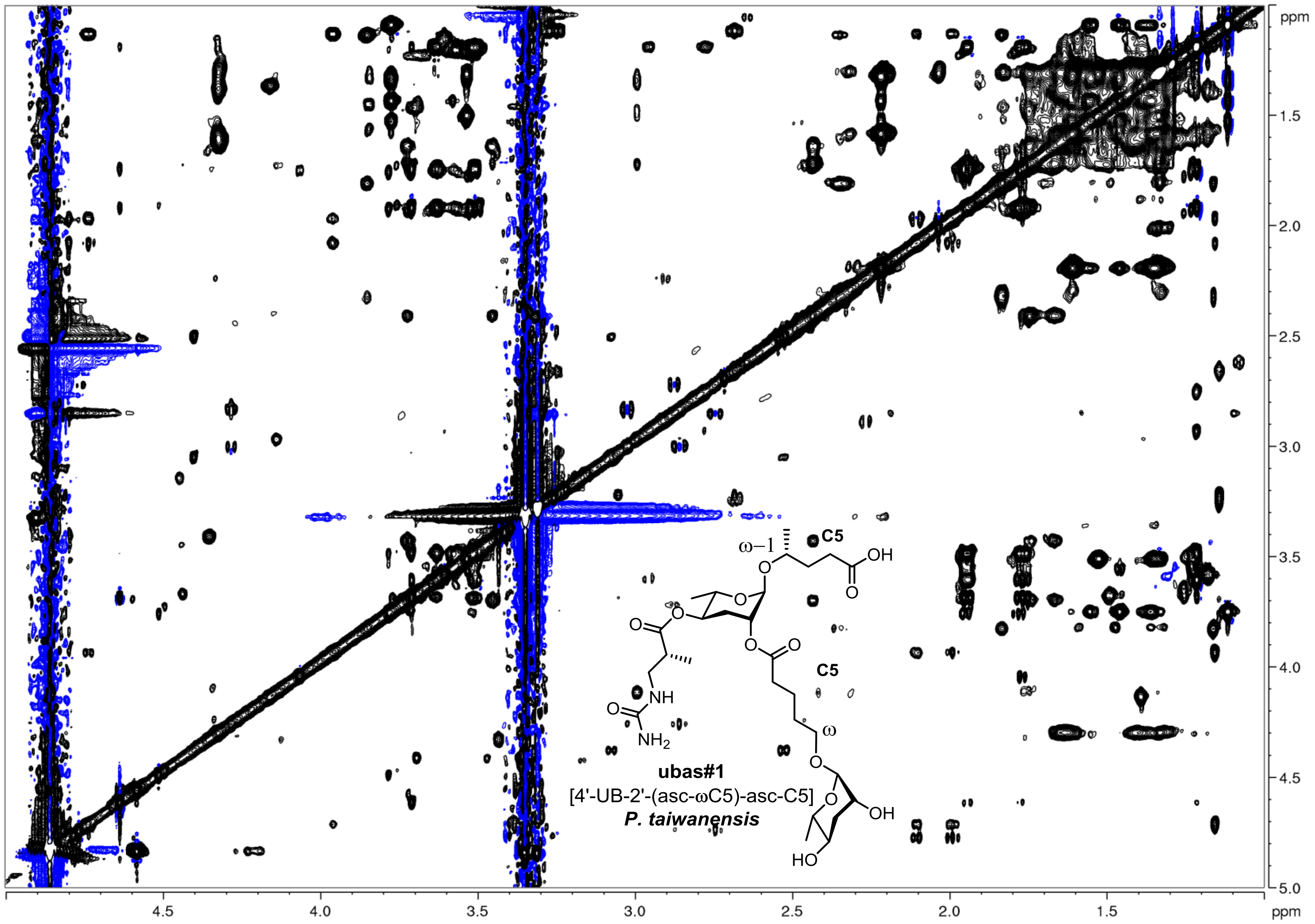

Figure 13. TOCSY spectrum of HPLC enriched sample containing ubas#1 [4'-UB-2'-(asc-ωC5)-asc-C5] (800 MHz, CD_3OD) isolated from the *exo*-metabolome of *P. taiwanensis*.

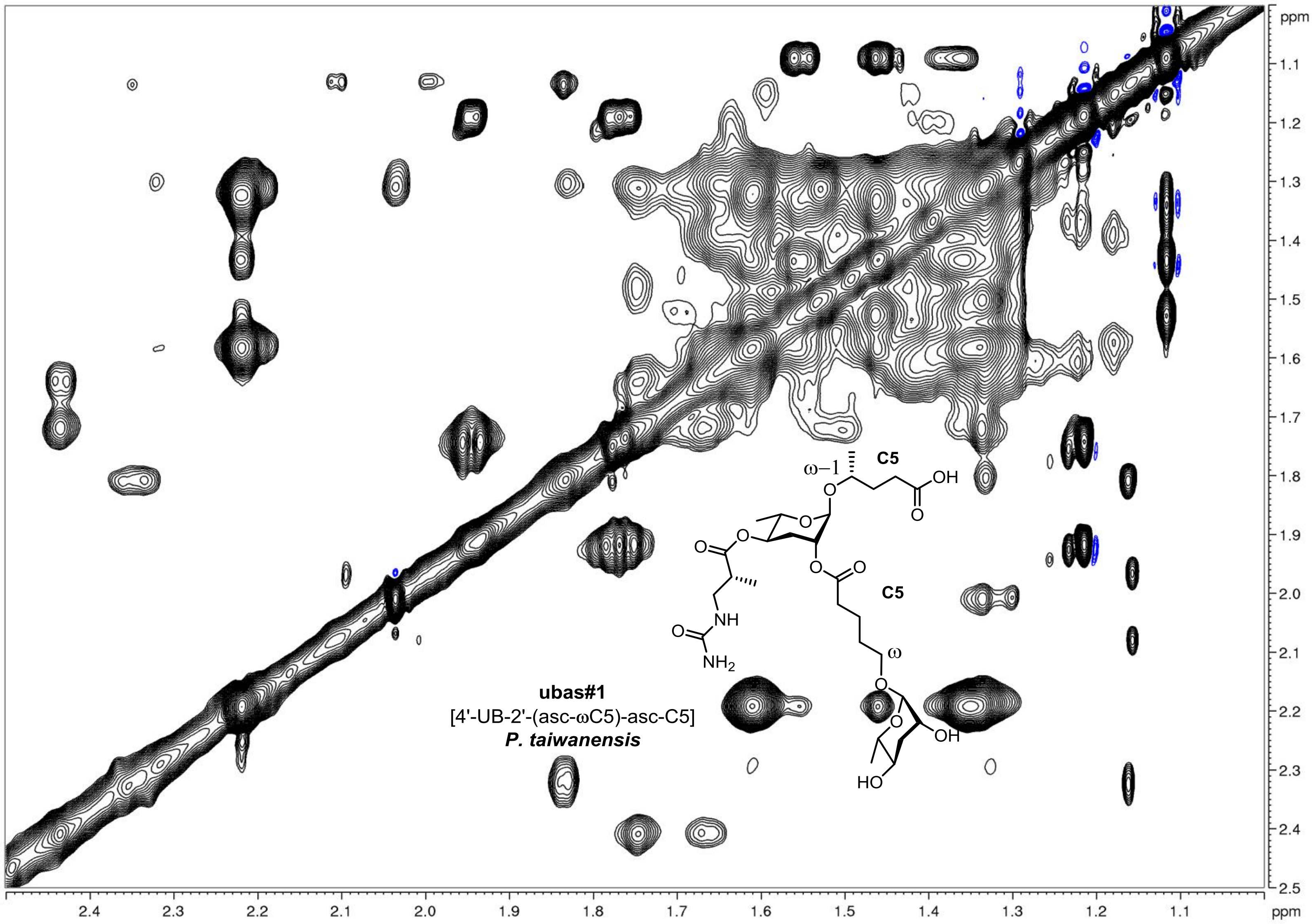

Figure 14. Selective TOCSY spectrum (amplified spectrum ranging from 1.0 ppm to 2.5 ppm) of HPLC enriched sample containing ubas#1 [4'-UB-2'-(asc-ωC5)-asc-C5] (800 MHz, CD_3OD) isolated from the *exo*-metabolome of *P. taiwanensis.*

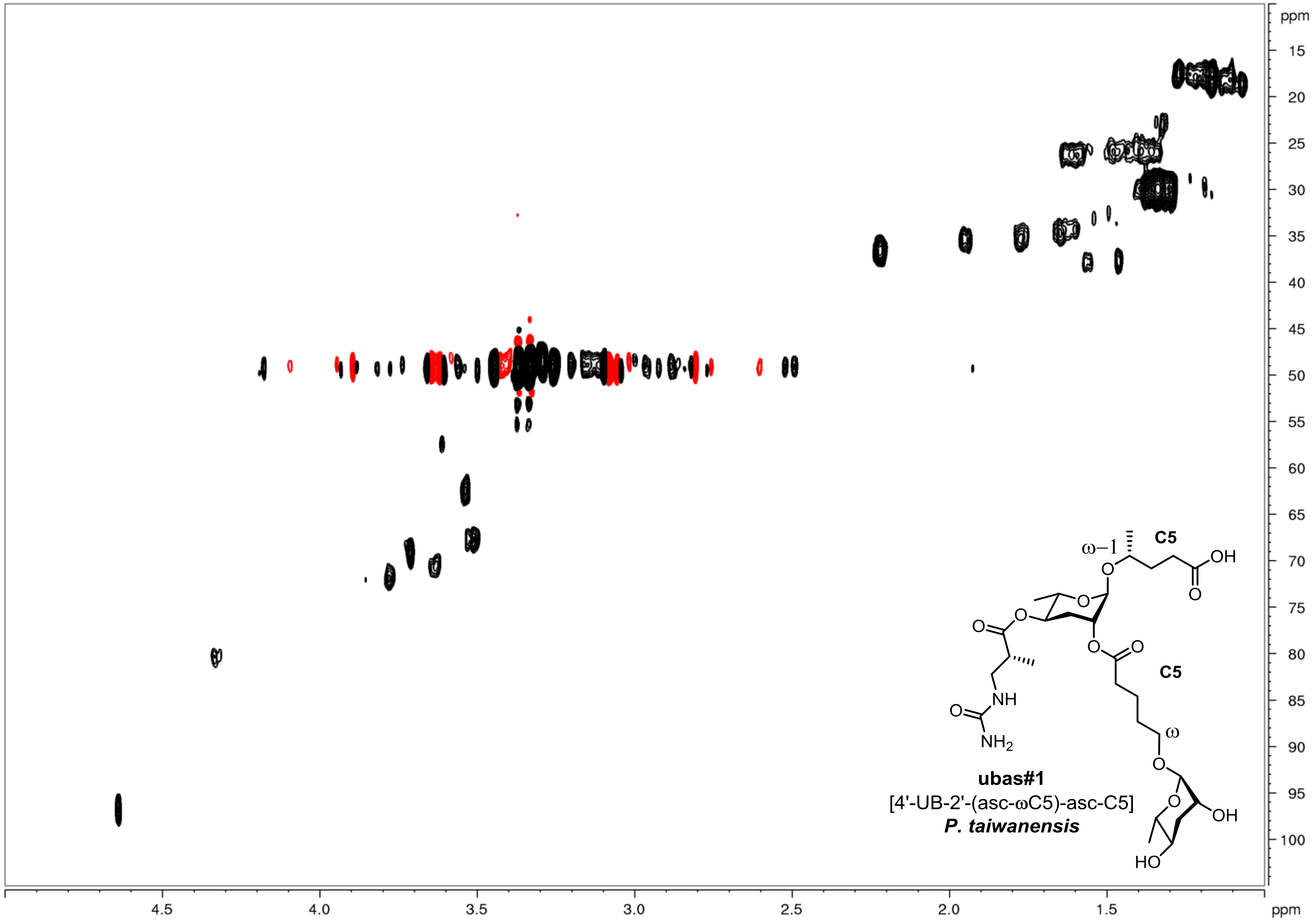

Figure 15. HSQC spectrum of HPLC enriched sample containing ubas#1 [4'-UB-2'-(asc-ωC5)-asc-C5] (800 MHz, CD_3OD) isolated from the *exo*-metabolome of *P. taiwanensis*.

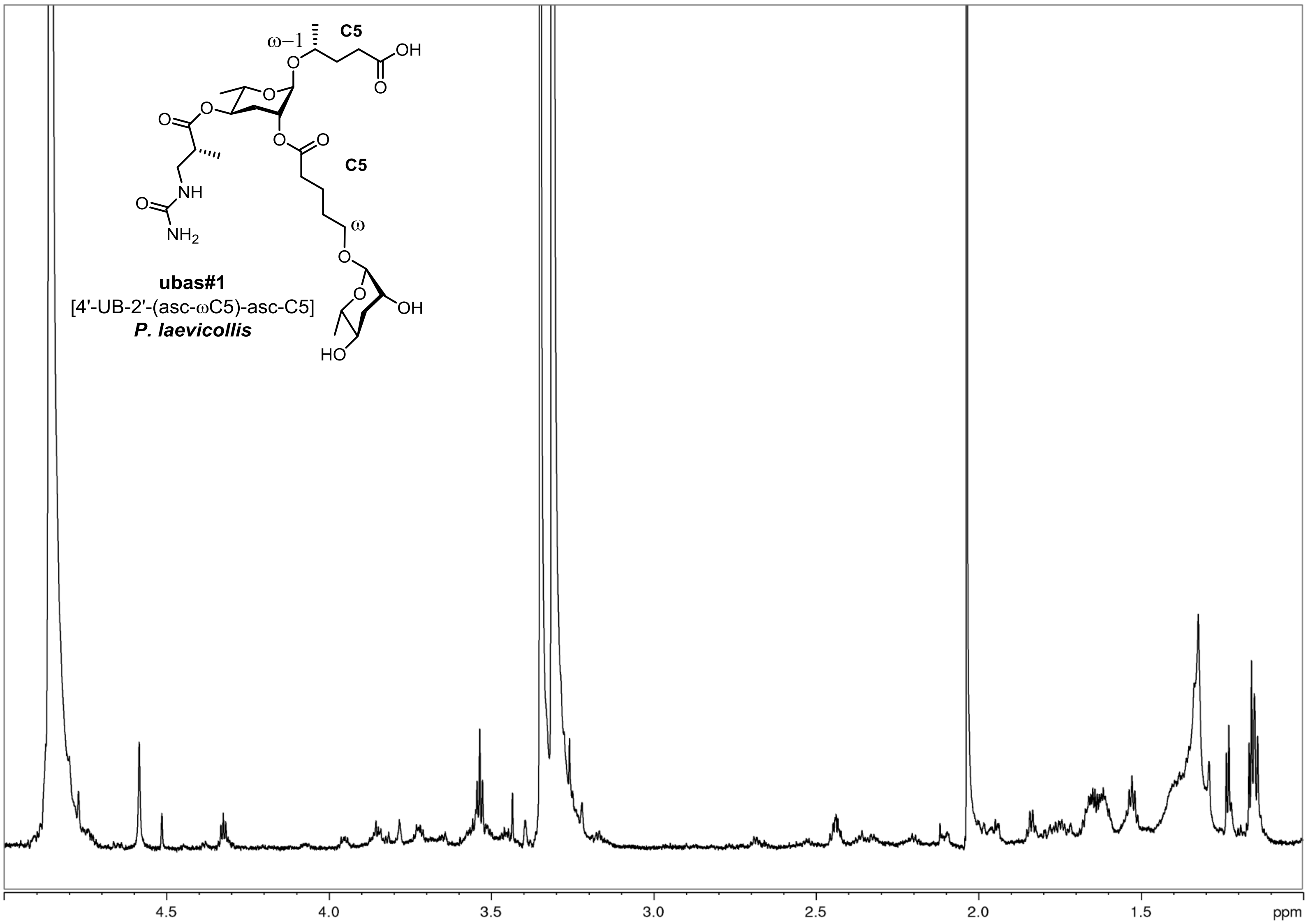

Figure 16. ^{1}H NMR spectrum of HPLC enriched sample containing ubas#1 [4'-UB-2'-(asc-ωC5)-asc-C5] (800 MHz, CD_3OD) isolated from the *exo*-metabolome of *P*. *laevicollis*.

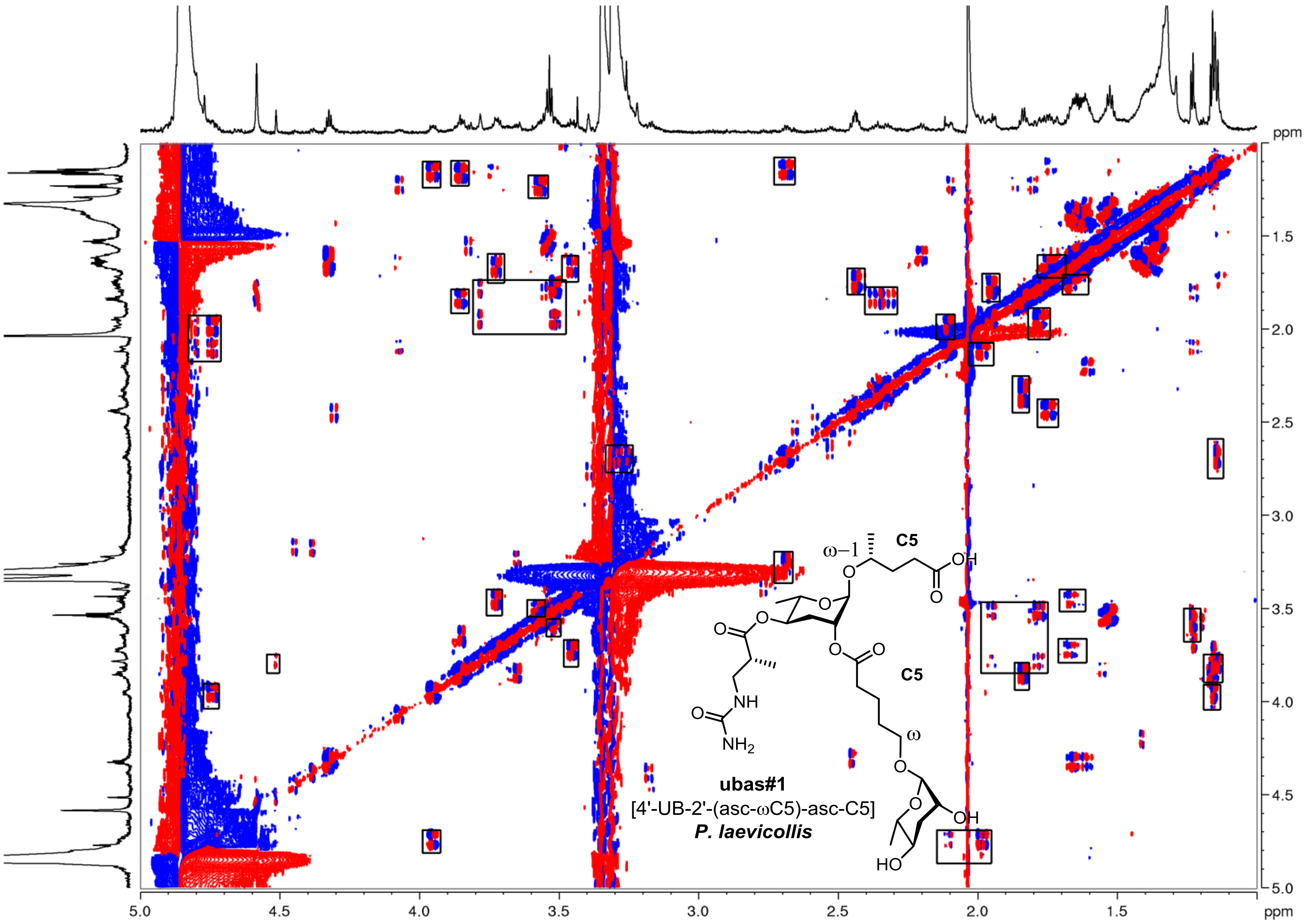

Figure 17. *dqf*-COSY spectrum of HPLC enriched sample containing ubas#1 [4'-UB-2'-(asc-ωC5)-asc-C5] (800 MHz, CD_3OD) isolated from the *exo*-metabolome of *P. laevicollis*.

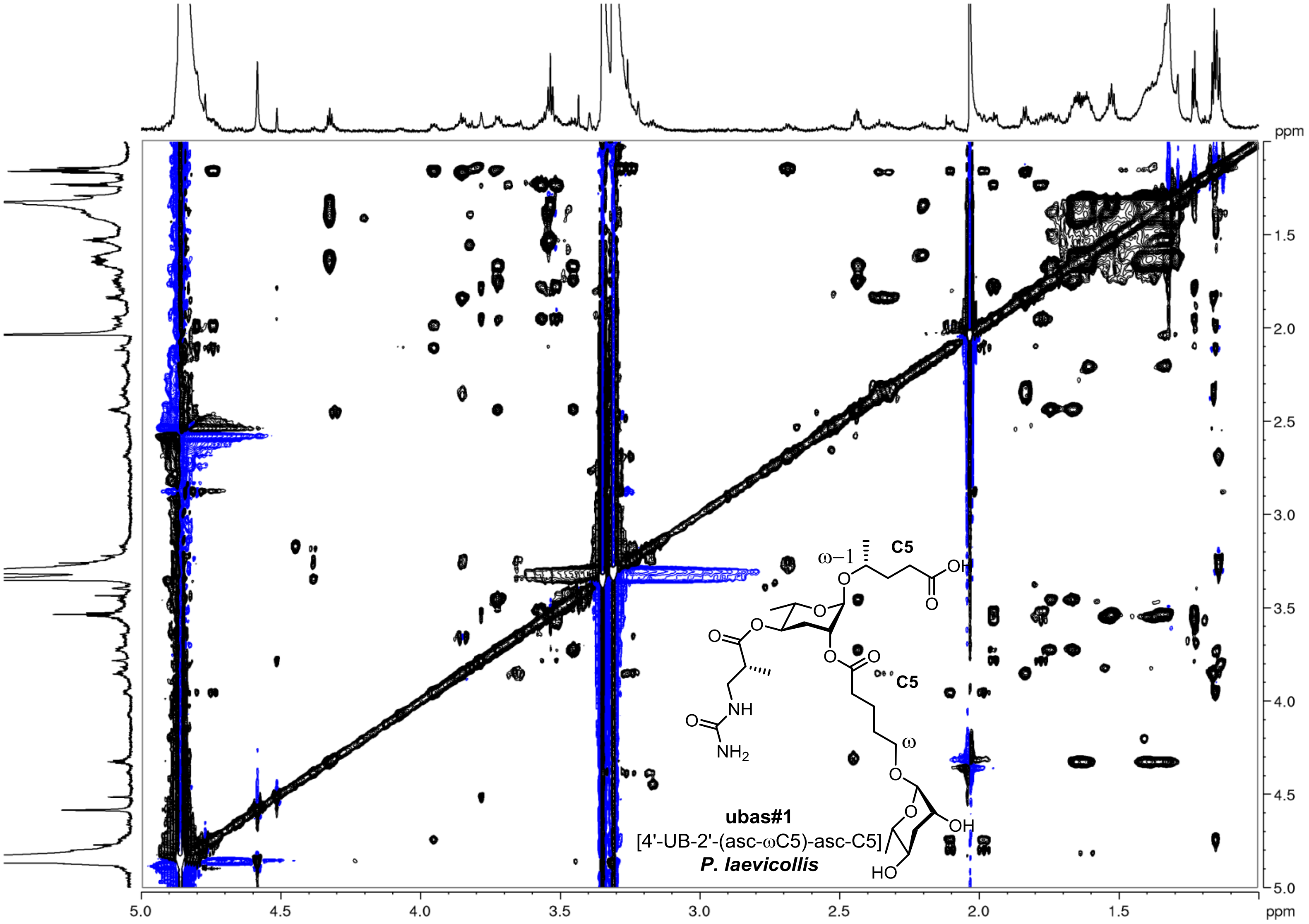

Figure 18. TOCSY spectrum of HPLC enriched sample containing ubas#1 [4'-UB-2'-(asc-ωC5)-asc-C5] (800 MHz, CD_3OD) isolated from the *exo*-metabolome of *P. laevicollis*.

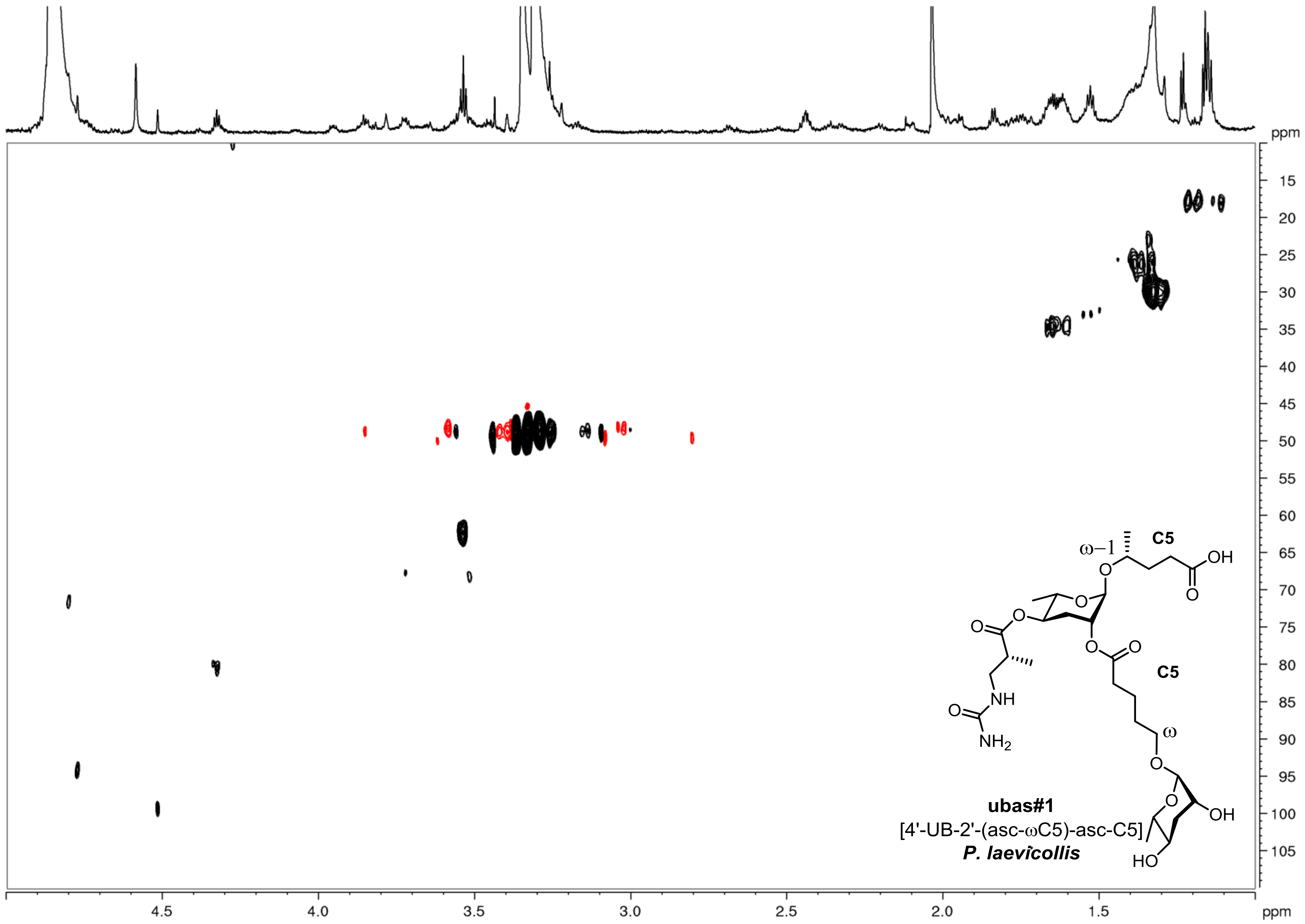

Figure 19. HSQC spectrum of HPLC enriched sample containing ubas#1 [4'-UB-2'-(asc-ωC5)-asc-C5] (800 MHz, CD_3OD) isolated from the *exo*-metabolome of *P. laevicollis*.

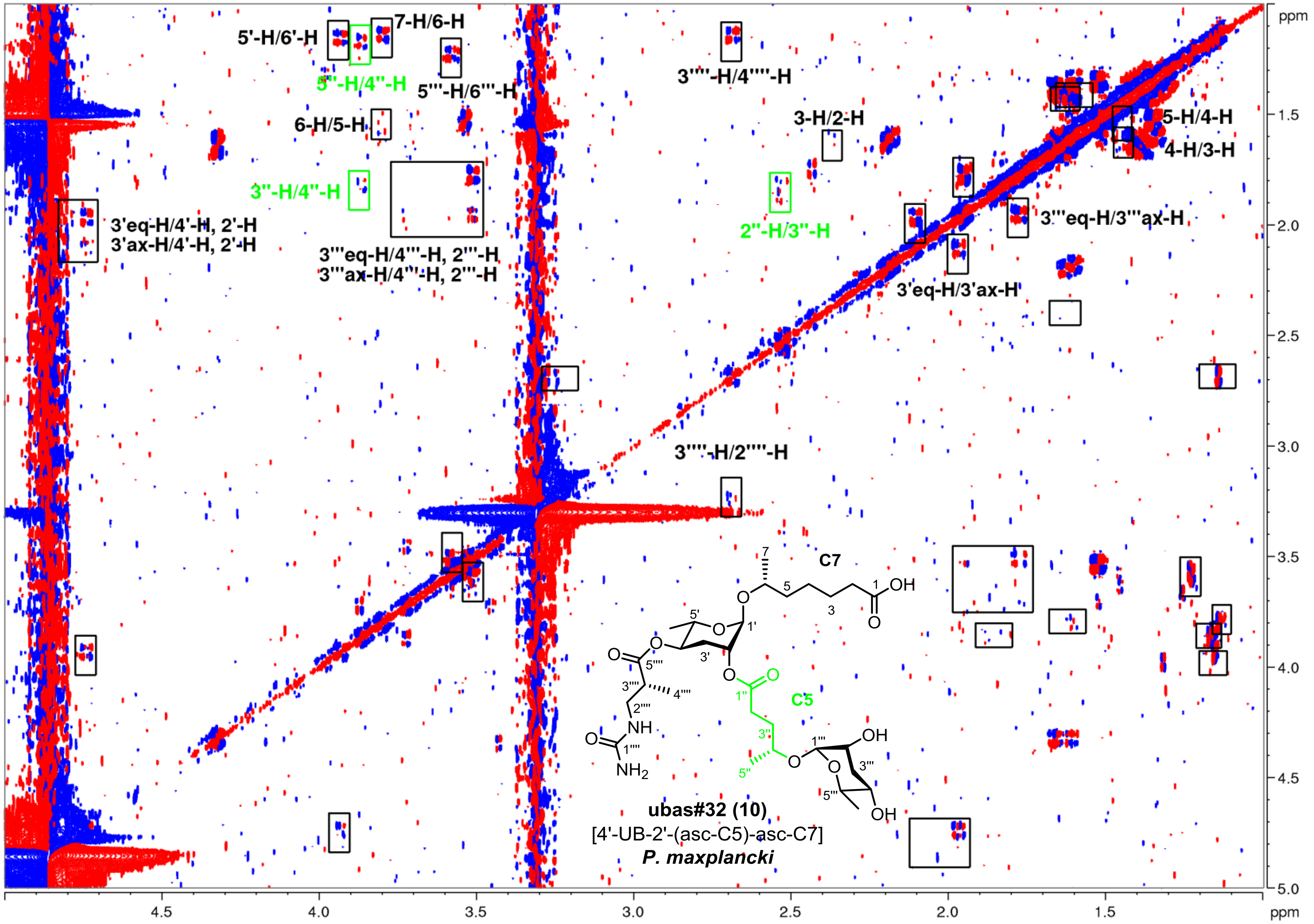

Figure 20. *dqf*-COSY spectrum of ubas#32 [4'-UB-2'-(asc-C5)-asc-C7, **10**] (800 MHz, CD_3OD) isolated from the *exo*-metabolome of *P. maxplancki*. Green colour boxed signals highlight the C5 fatty acid side chain in the first ascaroside.

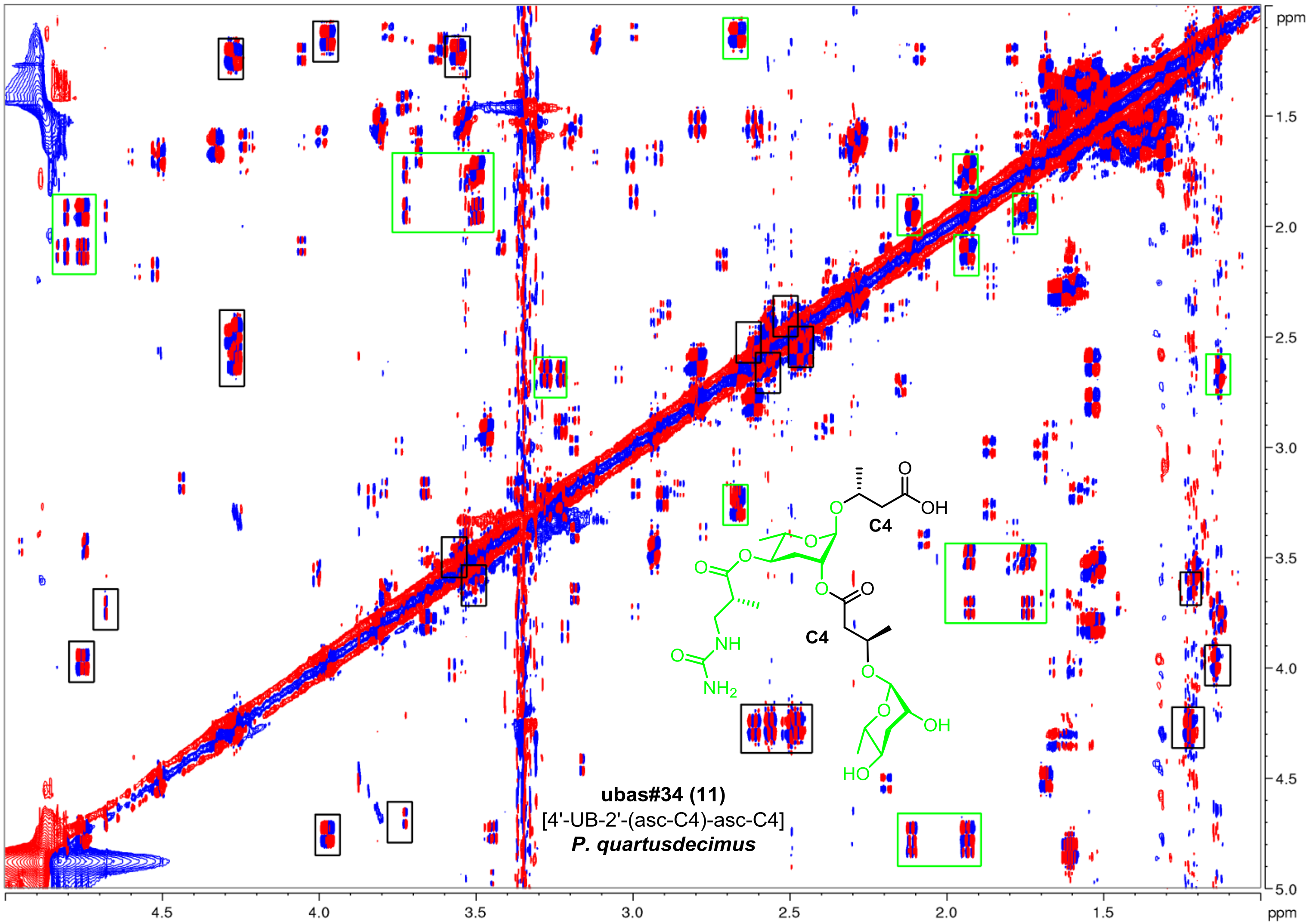

Figure 21. *dqf*-COSY spectrum an enriched SPE fraction containing ubas#34 [4'-UB-2'-(asc-C4)-asc-C4, **11**] (800 MHz, CD_3OD) isolated from the *exo*-metabolome of *P. quartusdecimus*. Green colour boxed sginals suggest that ubas#34 (**11**) contains a ureidoisobutyric acid building block and one free ascarylose sugar, as well as one 2',4'-substituted ascarylose sugar. Black colour boxed sginals suggest that ubas#34 (**11**) contains two ω-1 style C4 fatty acid side chains.

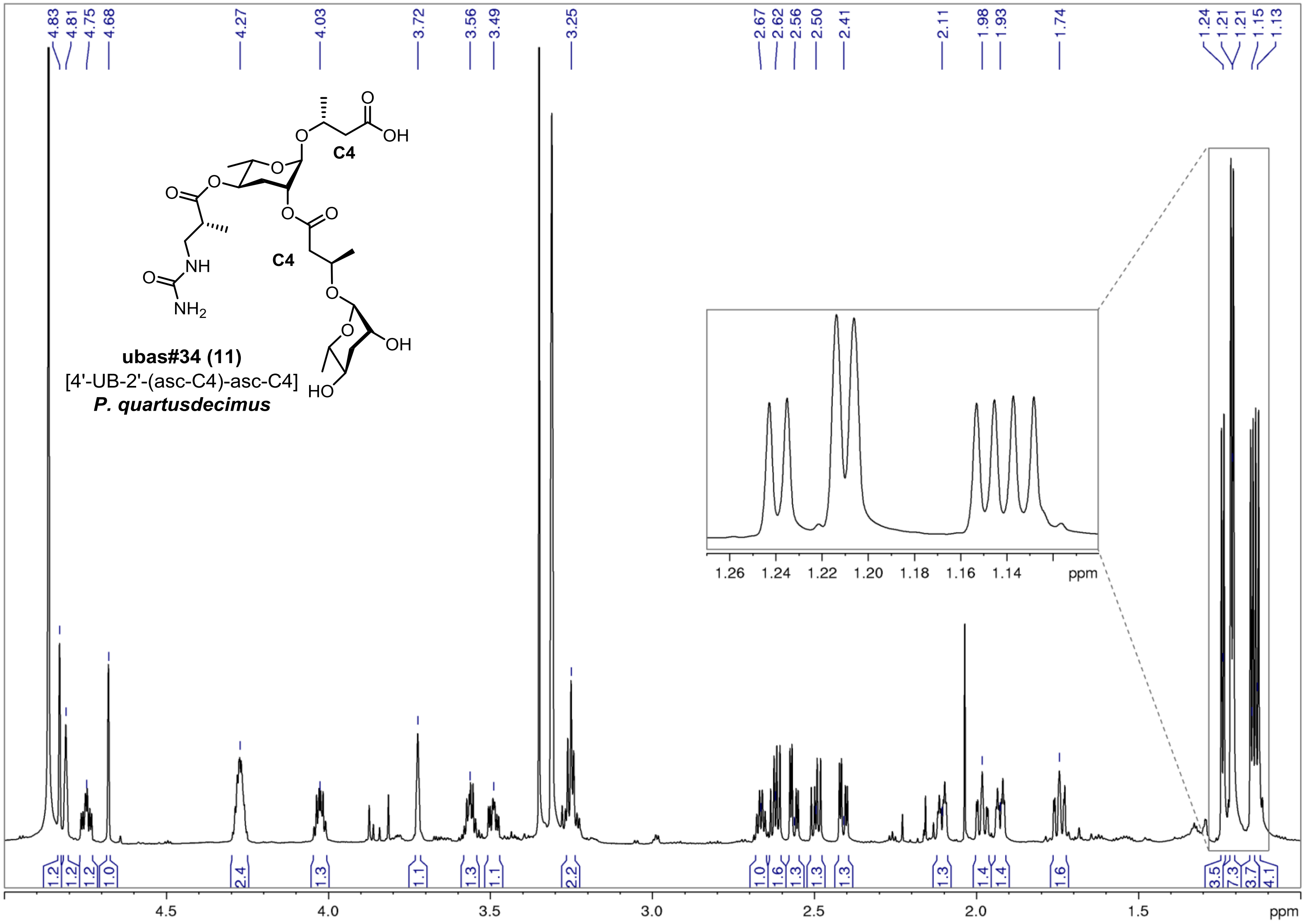

Figure 22. ^{1}H NMR spectrum of ubas#34 [4'-UB-2'-(asc-C4)-asc-C4, **11**] (800 MHz, CD_3OD) isolated from the *exo*-metabolome of *P. quartusdecimus*.

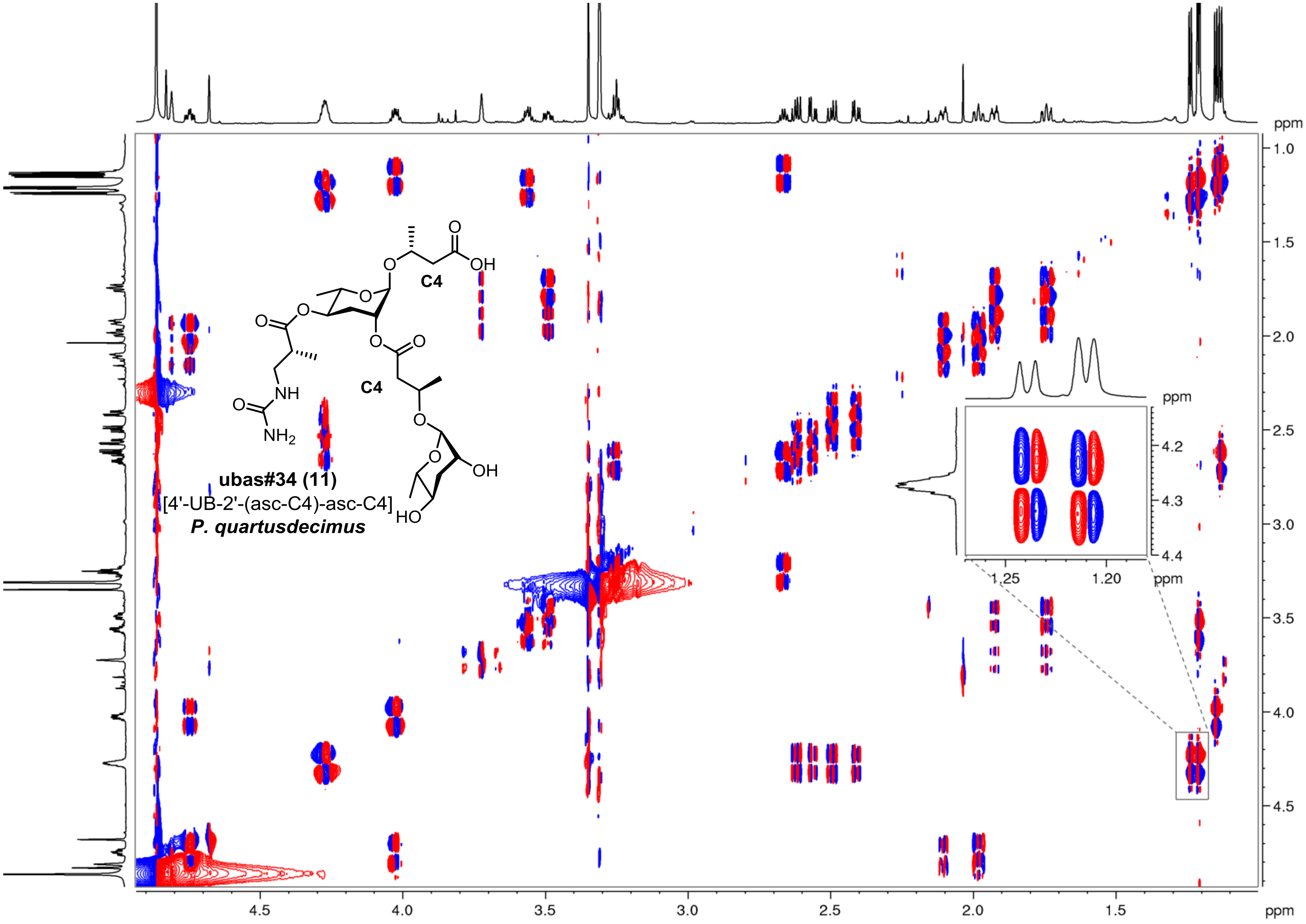

Figure 23. *dqf*-COSY spectrum of ubas#34 [4'-UB-2'-(asc-C4)-asc-C4, **11**] (800 MHz, CD_3OD) isolated from the *exo*-metabolome of *P. quartusdecimus*.

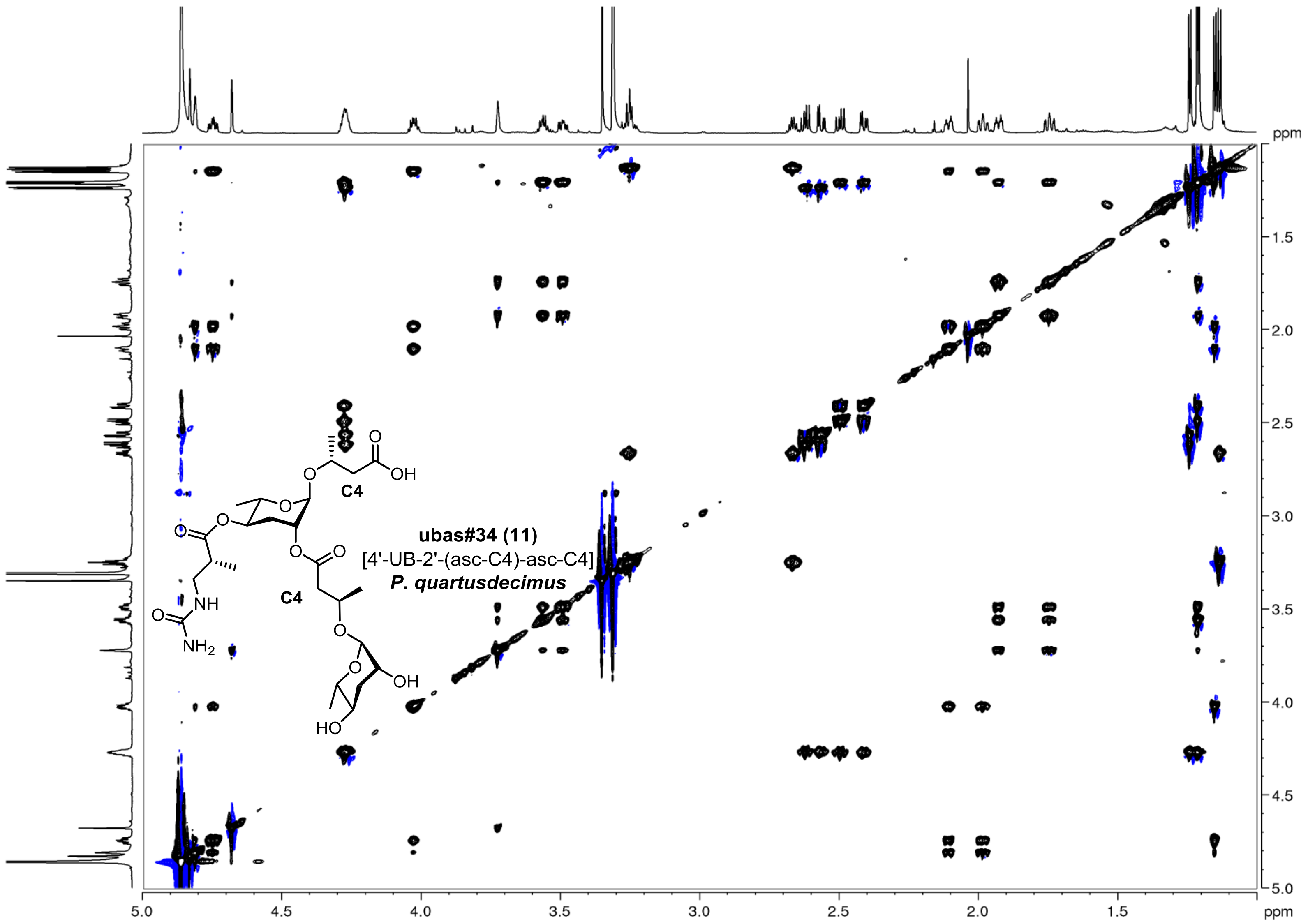

Figure 24. TOCSY spectrum of ubas#34 [4'-UB-2'-(asc-C4)-asc-C4, **11**] (800 MHz, CD_3OD) isolated from the *exo*-metabolome of *P. quartusdecimus*.

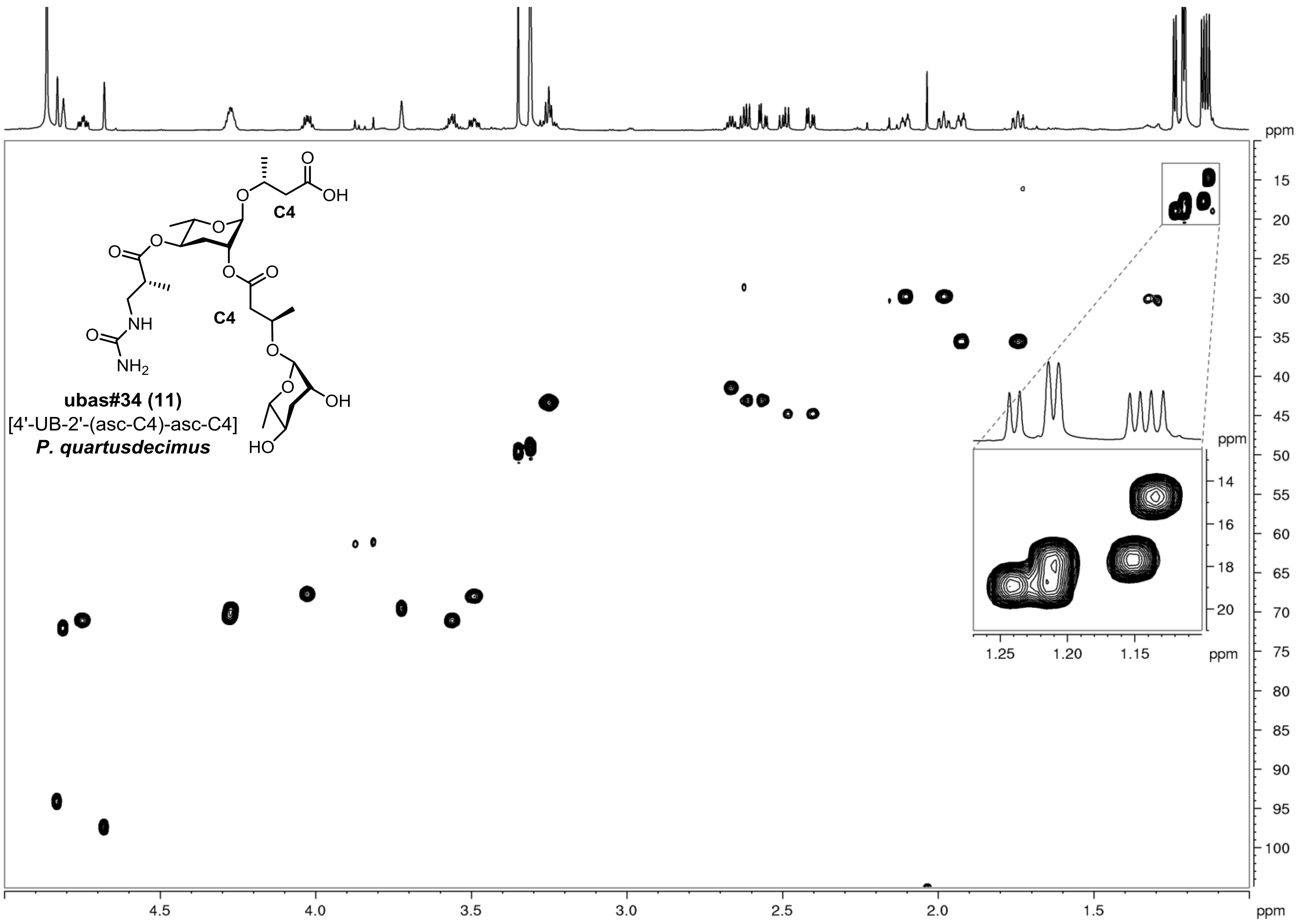

Figure 25. HSQC spectrum of ubas#34 [4'-UB-2'-(asc-C4)-asc-C4, **11**] (800 MHz, CD_3OD) isolated from the *exo*-metabolome of *P. quartusdecimus*.

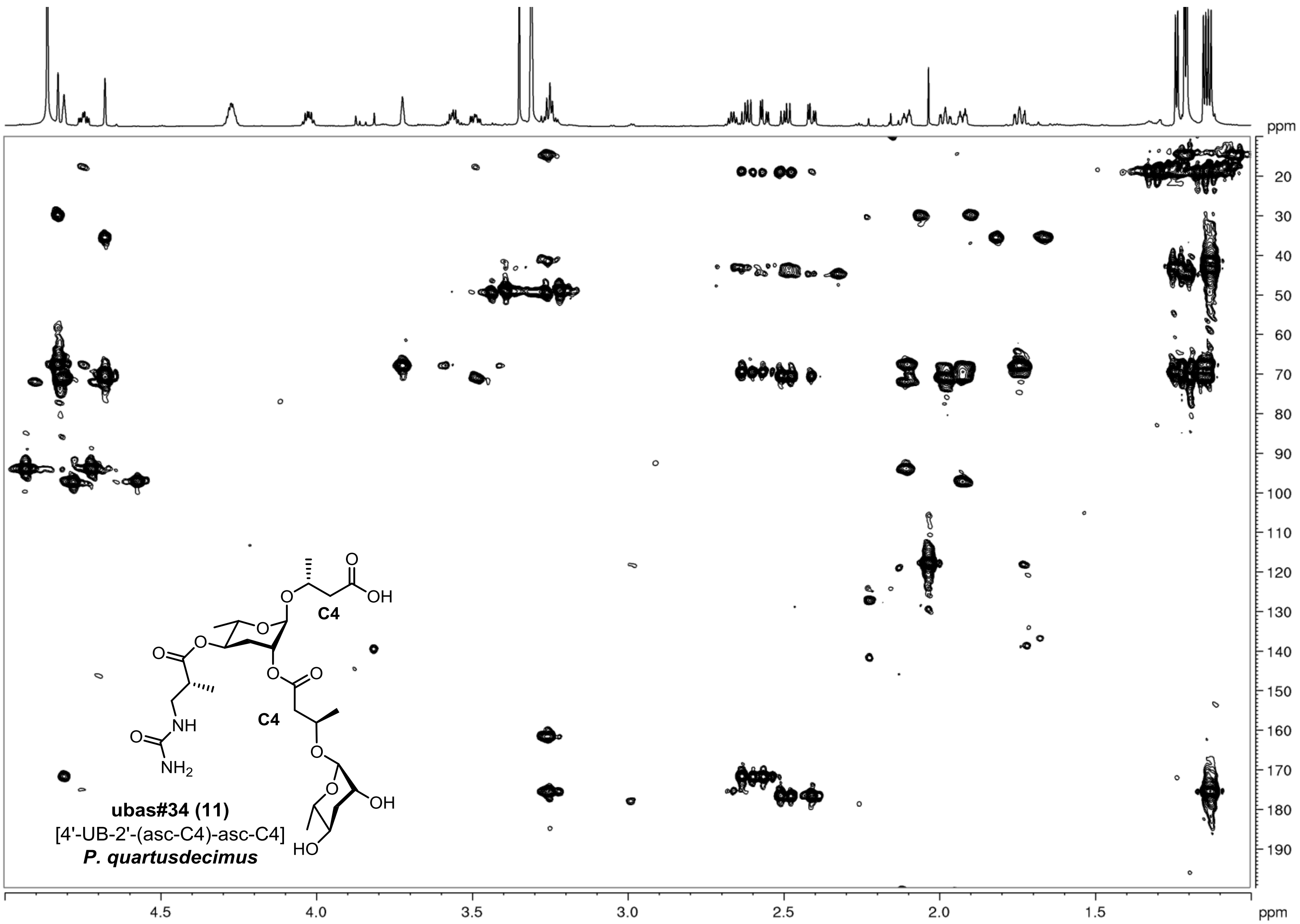

Figure 26. HMBC spectrum of ubas#34 [4'-UB-2'-(asc-C4)-asc-C4, **11**] (800 MHz, CD_3OD) isolated from the *exo*-metabolome of *P. quartusdecimus*.

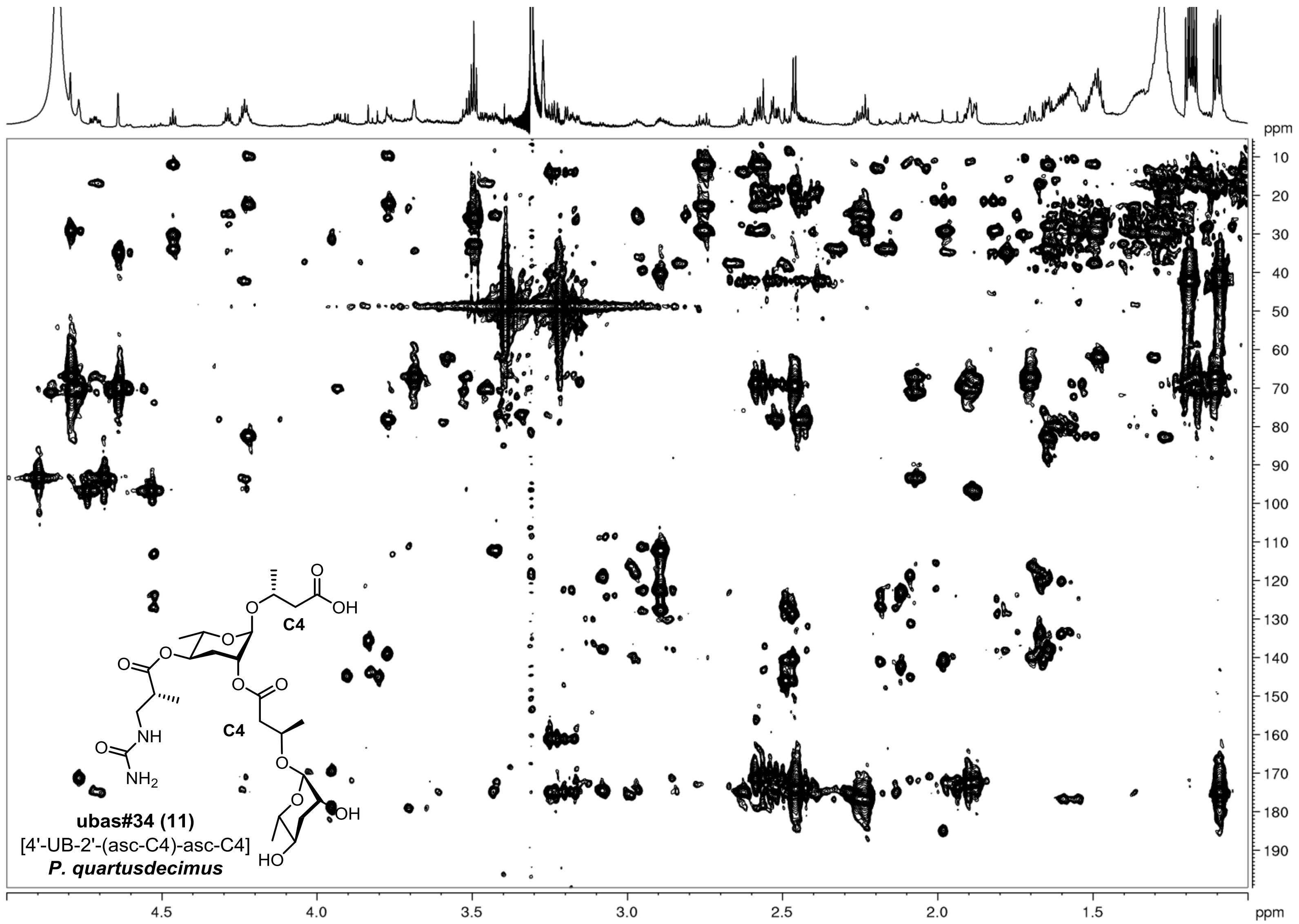

Figure 27. HMBC spectrum of a SPE fraction containing ubas#34 [4'-UB-2'-(asc-C4)-asc-C4, **11**] (800 MHz, CD_3OD) derived from the *exo*-metabolome of *P. quartusdecimus*.

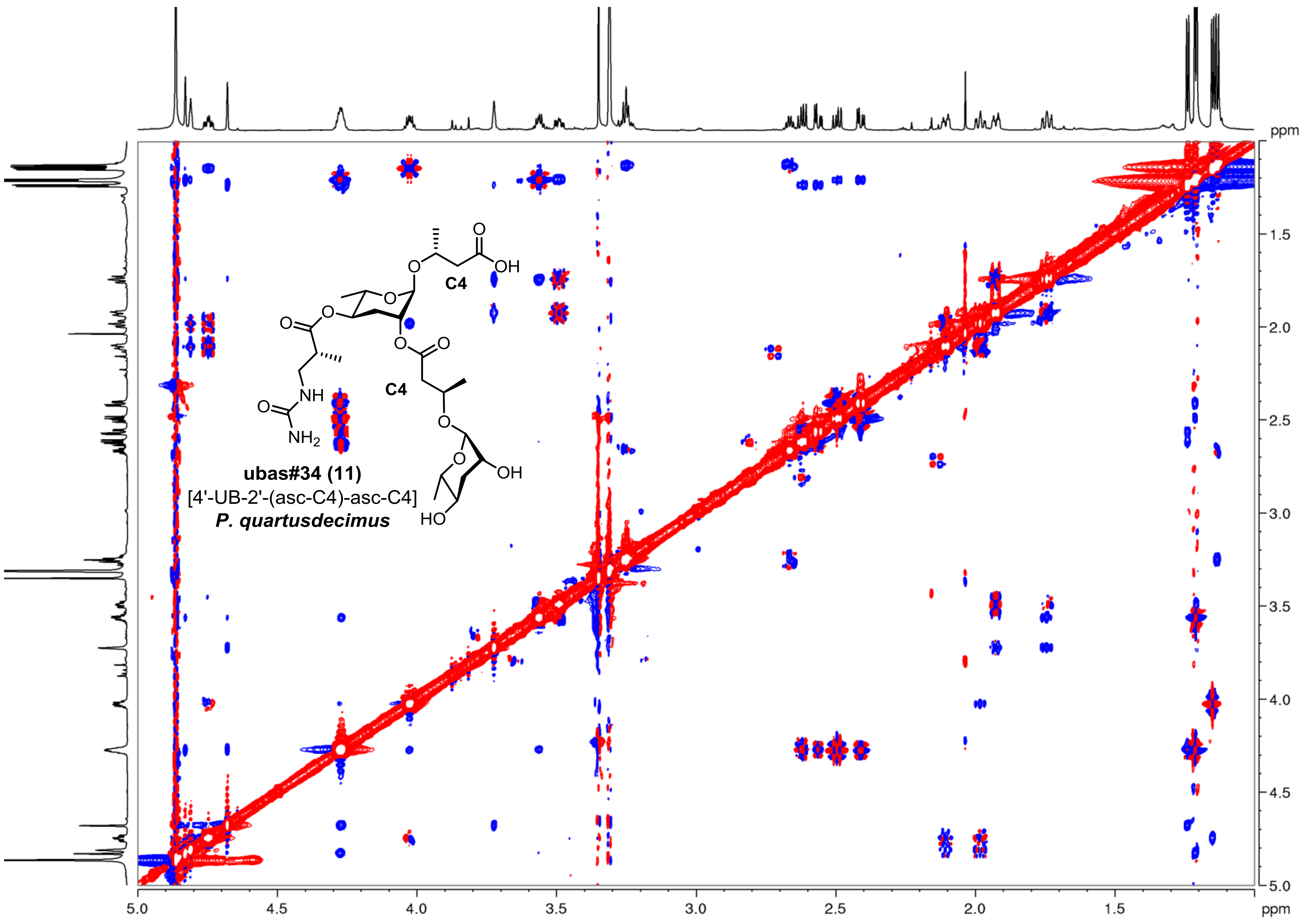

Figure 28. NOESY spectrum of ubas#34 [4'-UB-2'-(asc-C4)-asc-C4, **11**] (800 MHz, CD_3OD) isolated from the *exo*-metabolome of *P. quartusdecimus*.

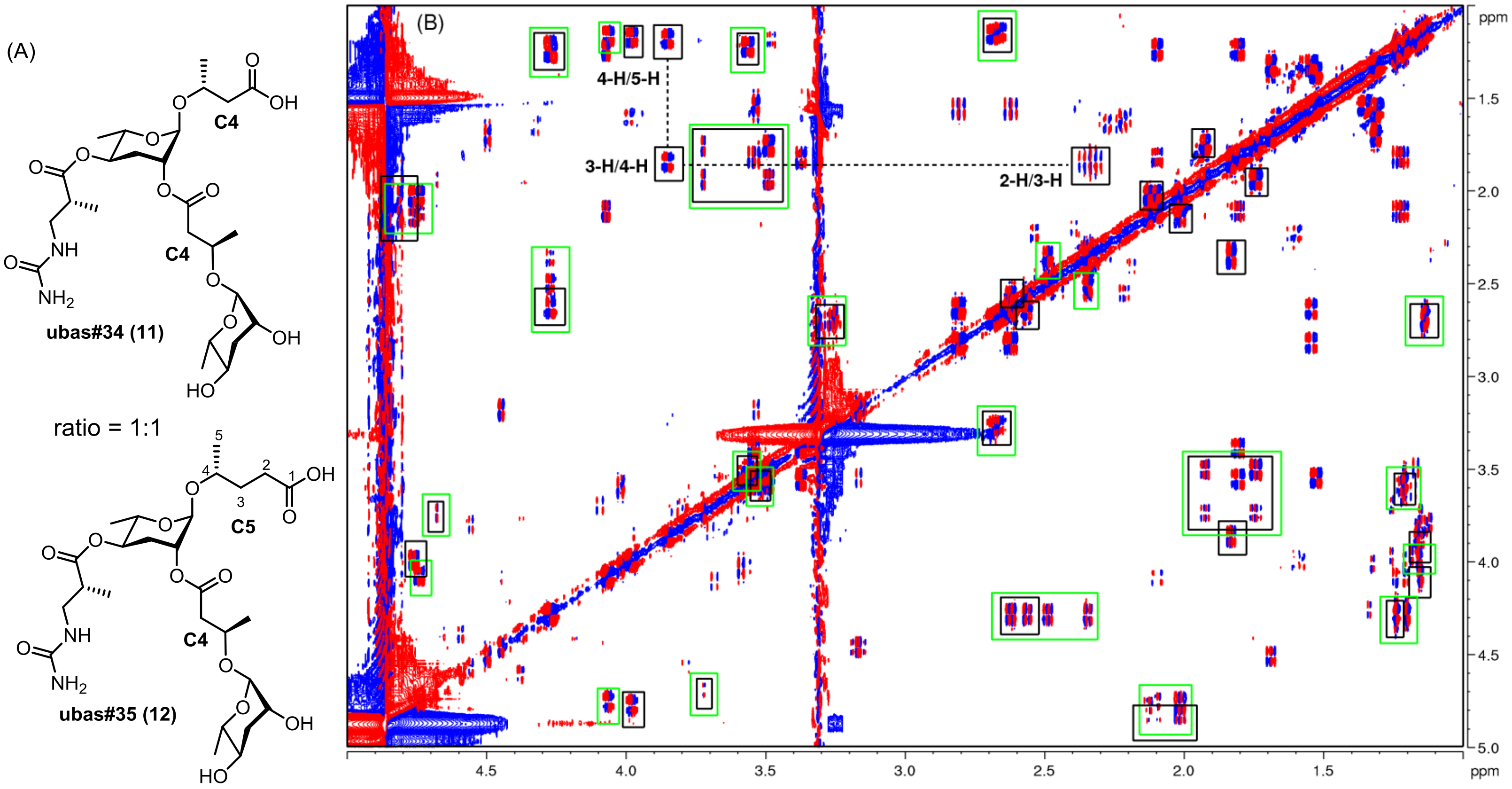

Figure 29. Structural characterization of ubas#35 [4'-UB-2'-(asc-C4)-asc-C5, **12**] which was isolated in a mixture (1:1 ratio) with ubas#34 [4'-UB-2'-(asc-C4)-asc-C4, **11**] (800 MHz, CD_3OD) from the *exo*-metabolome of *P. quartusdecimus*. (**A**) Chemical structures of ubas#34 (**11**) and ubas#35 (**12**). (**B**) *dqf*-COSY spectrum of an HPLC-enriched sample containing a 1:1 mixture of ubas#34 (**11**) and ubas#35 (**12**). Green and black colors boxed correlation signals are corresponding to ubas#34 (**11**) and ubas#35 (**12**), respectively. Signals of C5 fatty acid side chain are observed in the *dqf*-COSY spectrum, in line with the MS/MS fragmentation data of ubas#35 (**12**) (***supplementary file 2c – Figure 10***), confirms that the second ascaroside (ascr#9 "asc-C5") in ubas#35 (**12**) contains a ω-1 style C5 fatty acid side chain. Proton signals assignments for ubas#35 (**12**) are listed in ***supplementary file 3 – Table 11***.

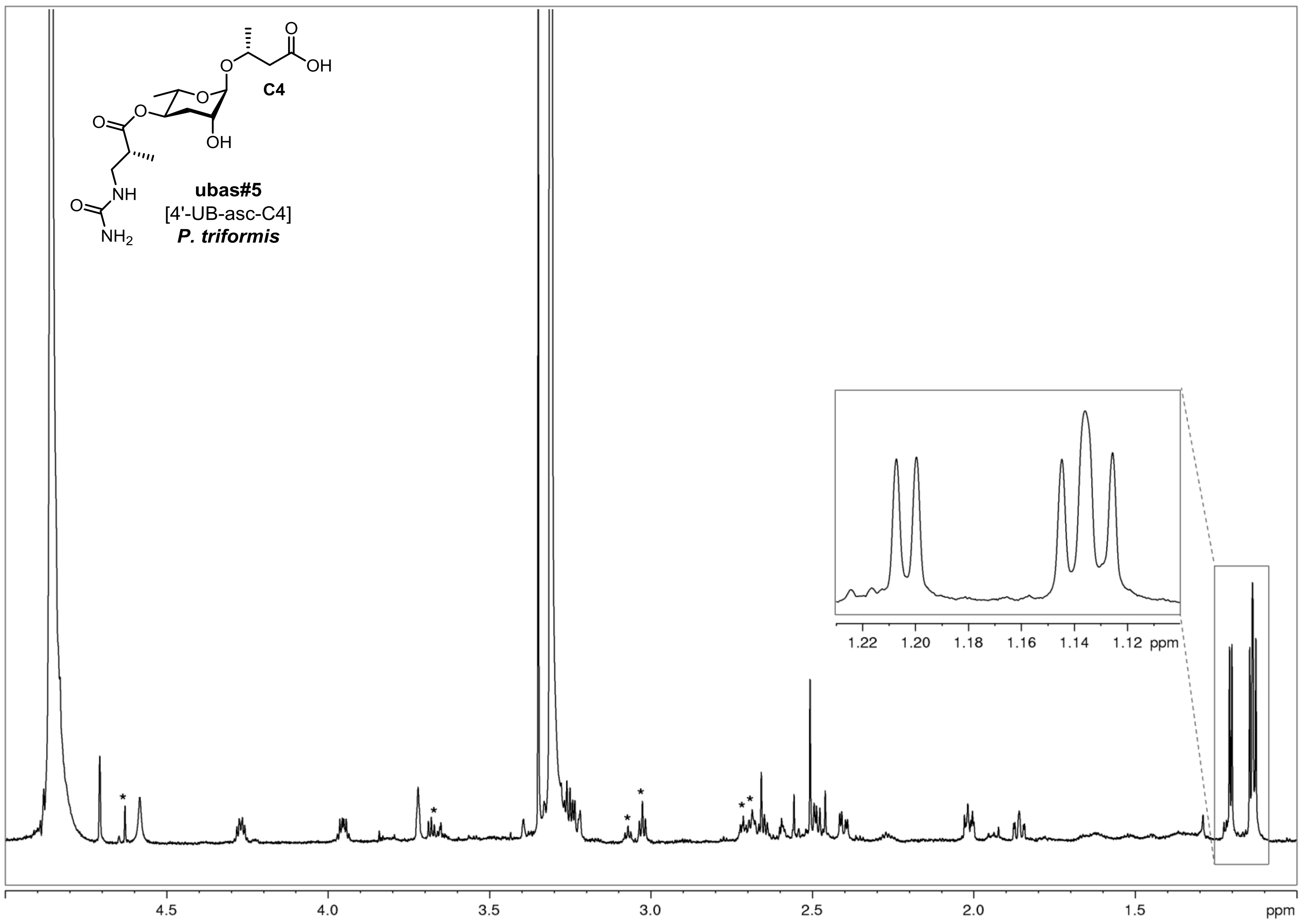

Figure 30. ^{1}H NMR spectrum of ubas#5 [4'-UB-asc-C4] (800 MHz, CD_3OD) isolated from the *exo*-metabolome of *P. triformis*. Asterisks marked signals are derived from impurities.

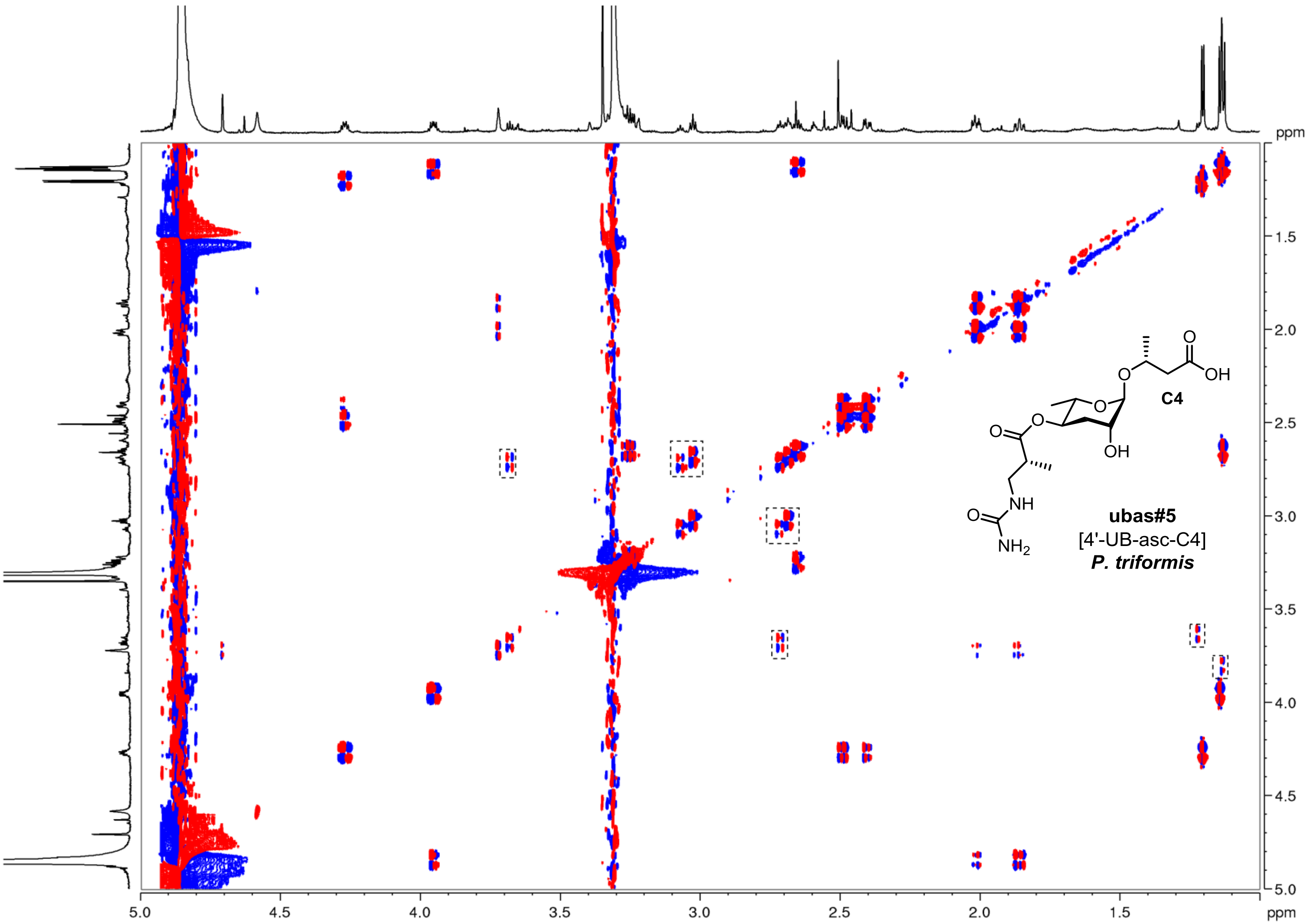

Figure 31. *dqf*-COSY spectrum of ubas#5 [4'-UB-asc-C4] (800MHz, CD_3OD) isolated from the *exo*-metabolome of *P. triformis*. Dashed lined boxed signals are derived from impurities.

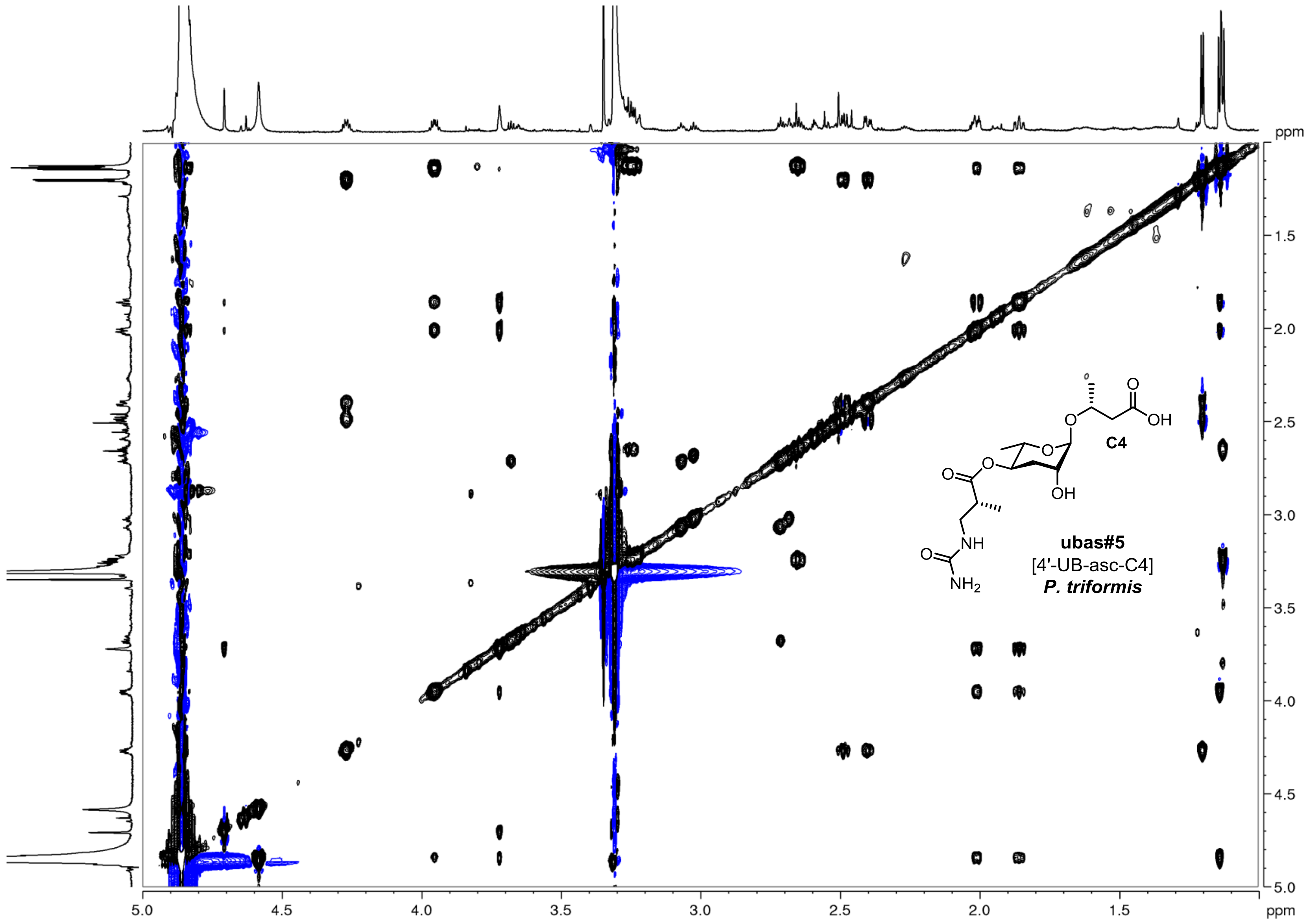

Figure 32. TOCSY spectrum of ubas#5 [4'-UB-asc-C4] (800MHz, CD_3OD) isolated from the *exo*-metabolome of *P. triformis*.

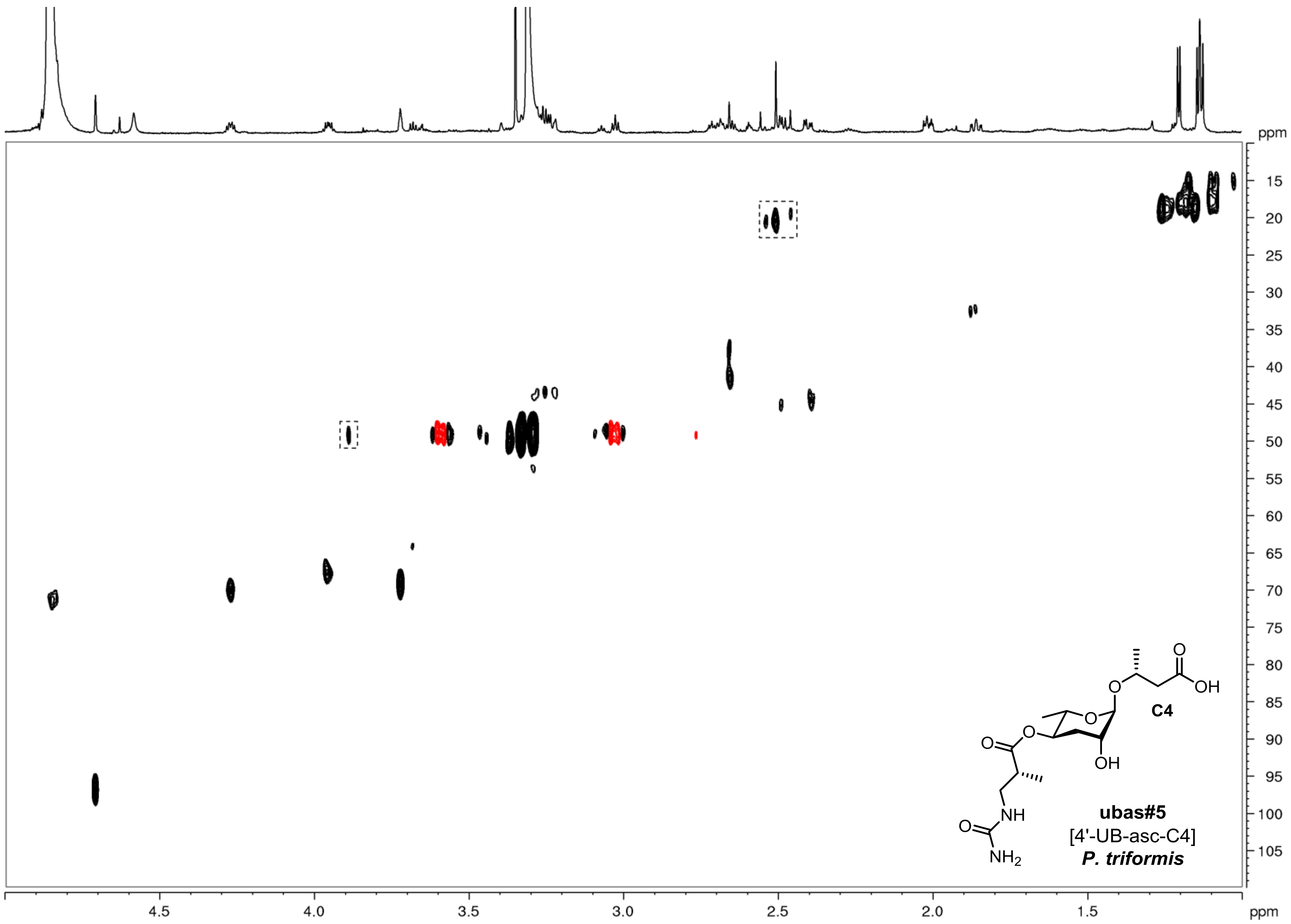

Figure 33. HSQC spectrum of ubas#5 [4'-UB-asc-C4] (800MHz, CD_3OD) isolated from the *exo*-metabolome of *P. triformis*. Boxed signals are from impurities.

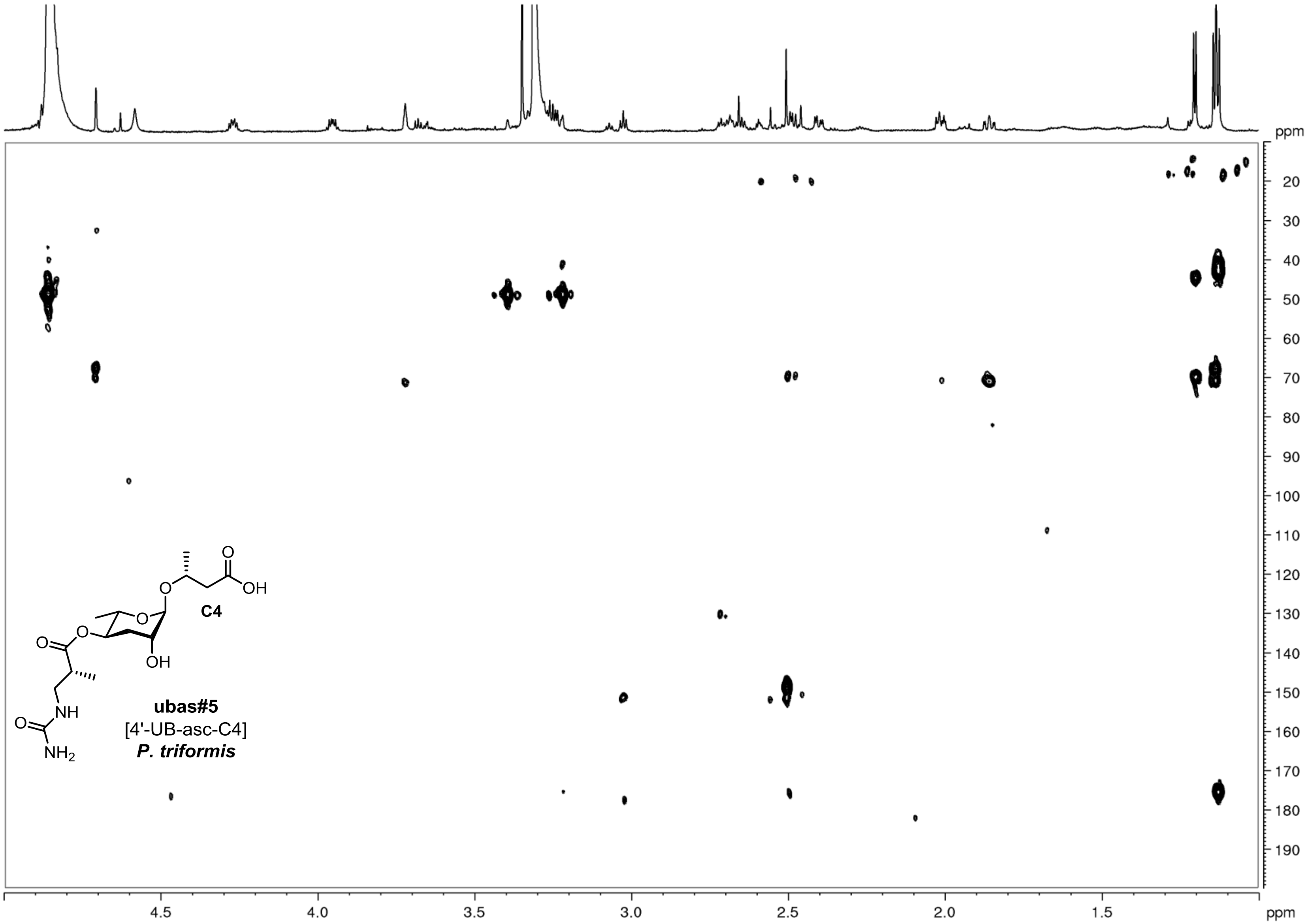

Figure 34. HMBC spectrum of ubas#5 [4'-UB-asc-C4] (800MHz, CD_3OD) isolated from the *exo*-metabolome of *P. triformis*.

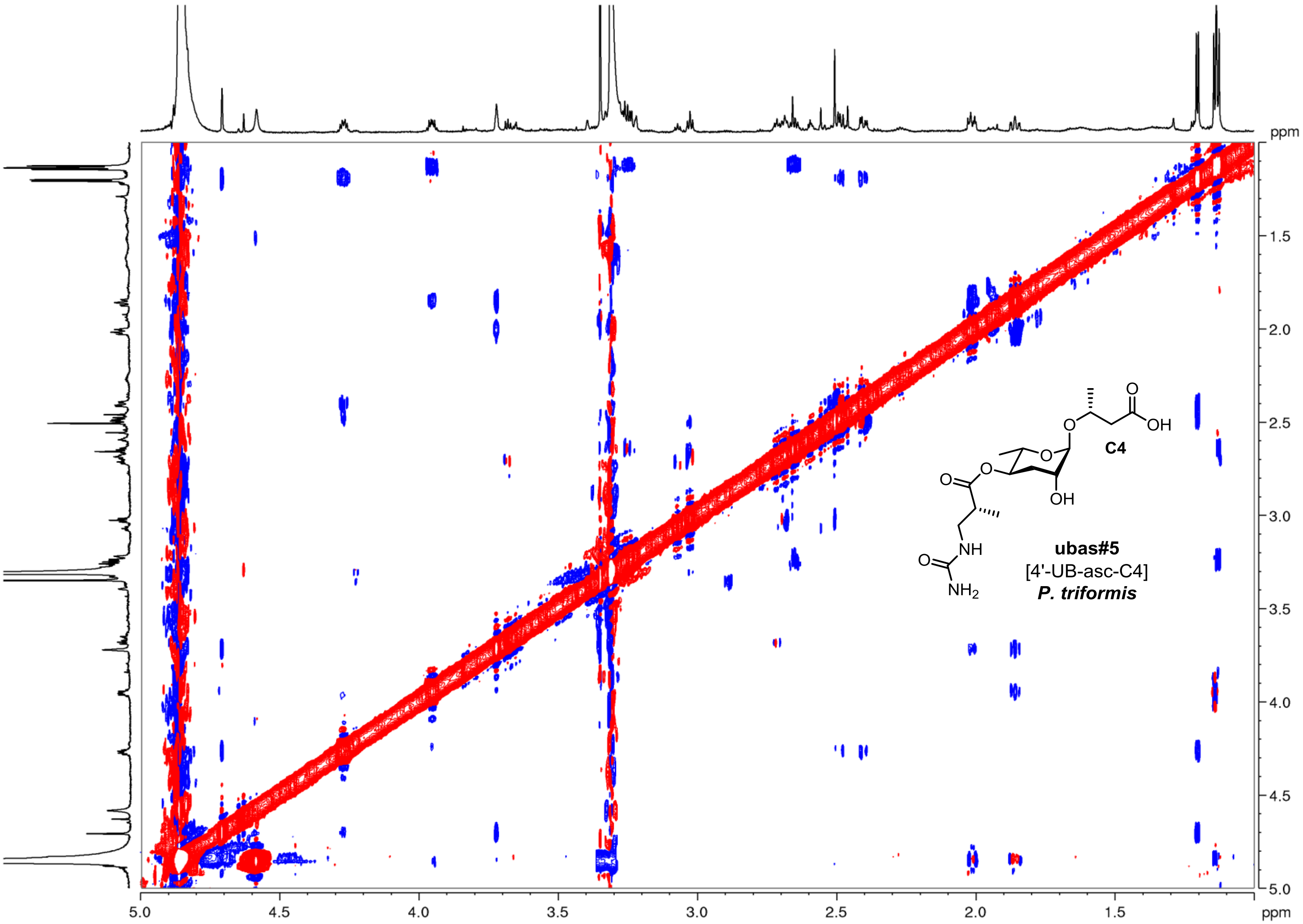

Figure 35. NOESY spectrum of ubas#5 [4'-UB-asc-C4] (800MHz, CD_3OD) isolated from the *exo*-metabolome of *P*. *triformis*.

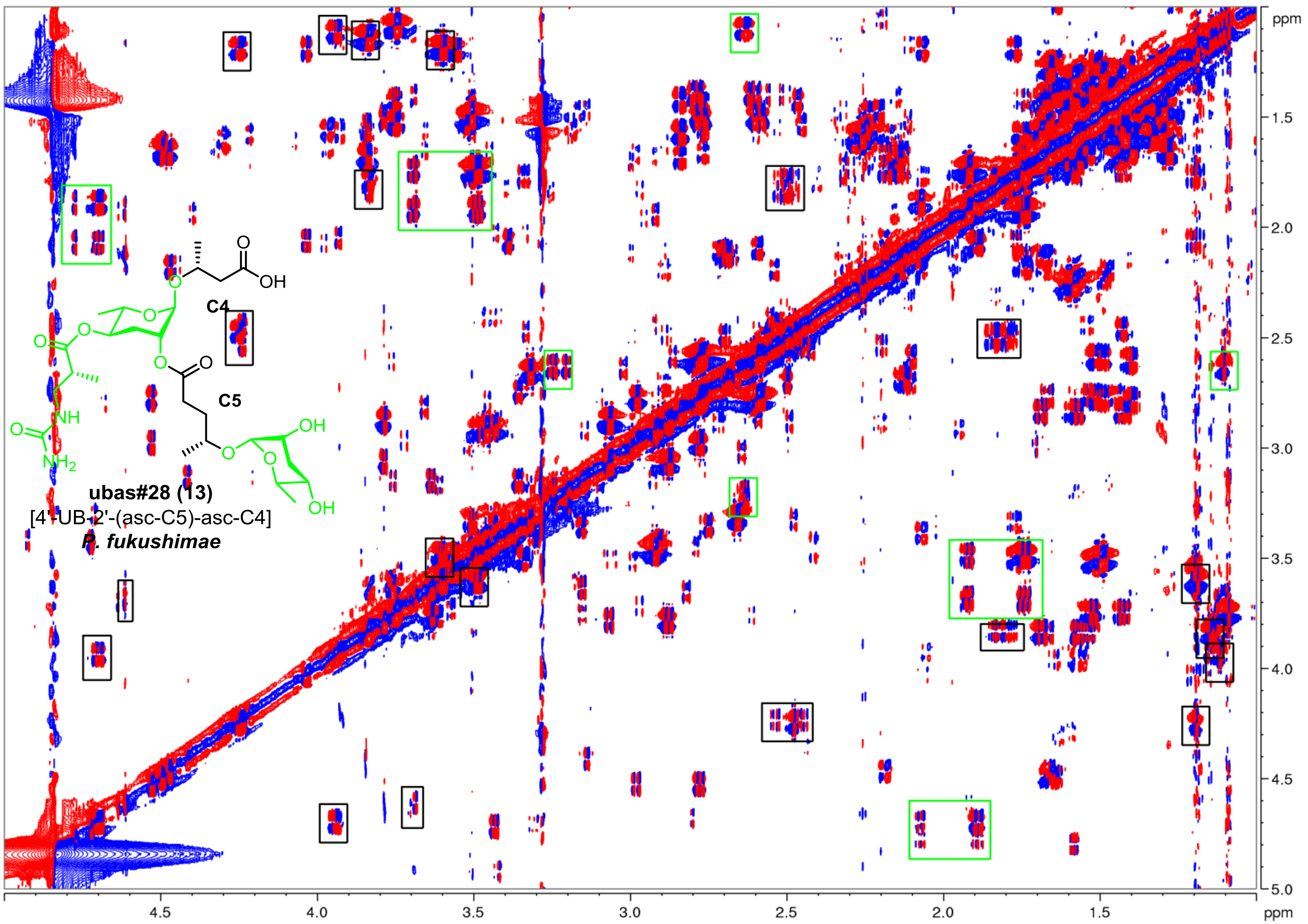

Figure 36. *dqf*-COSY spectrum of an SPE fraction containing ubas#28 [4'-UB-2'-(asc-C5)-asc-C4, **13**] (in CD_3OD) isolated from the *exo*-metabolome of *P. fukushimae*. Green colour boxed sginals suggest that ubas#28 (**13**) contains a ureidoisobutyric acid building block and one free ascarylose sugar, as well as one 2',4'-substituted ascarylose sugar. Black colour boxed signals suggest that the two ascaroside units in ubas#28 (**13**) contain one ω-1 style C4 fatty acid side chain and one ω-1 style C5 fatty acid side chain, respectively.

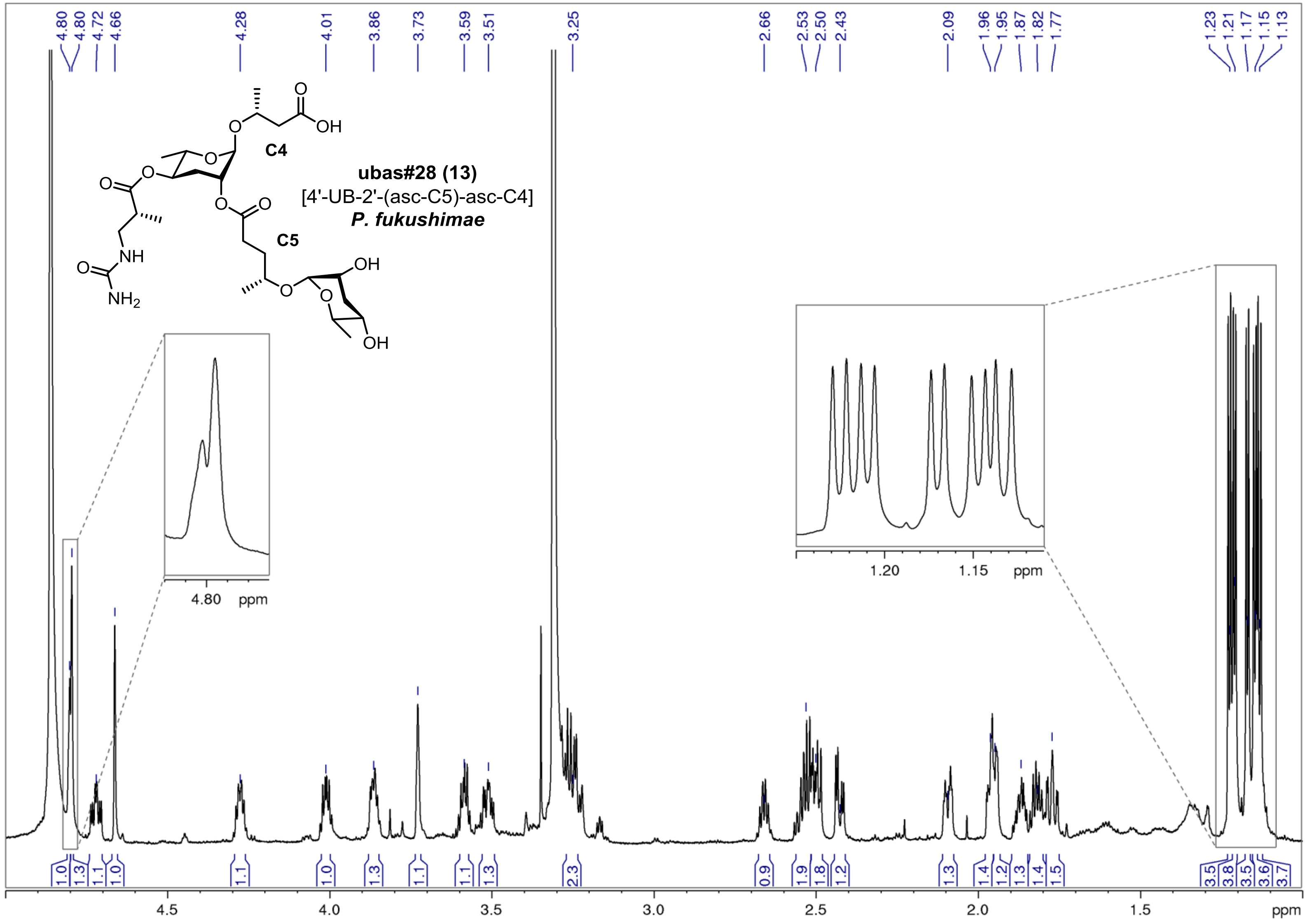

Figure 37. ^{1}H NMR spectrum of ubas#28 [4'-UB-2'-(asc-C5)-asc-C4, **13**] (800 MHz, CD_3OD) isolated from the *exo*-metabolome of *P. fukushimae*.

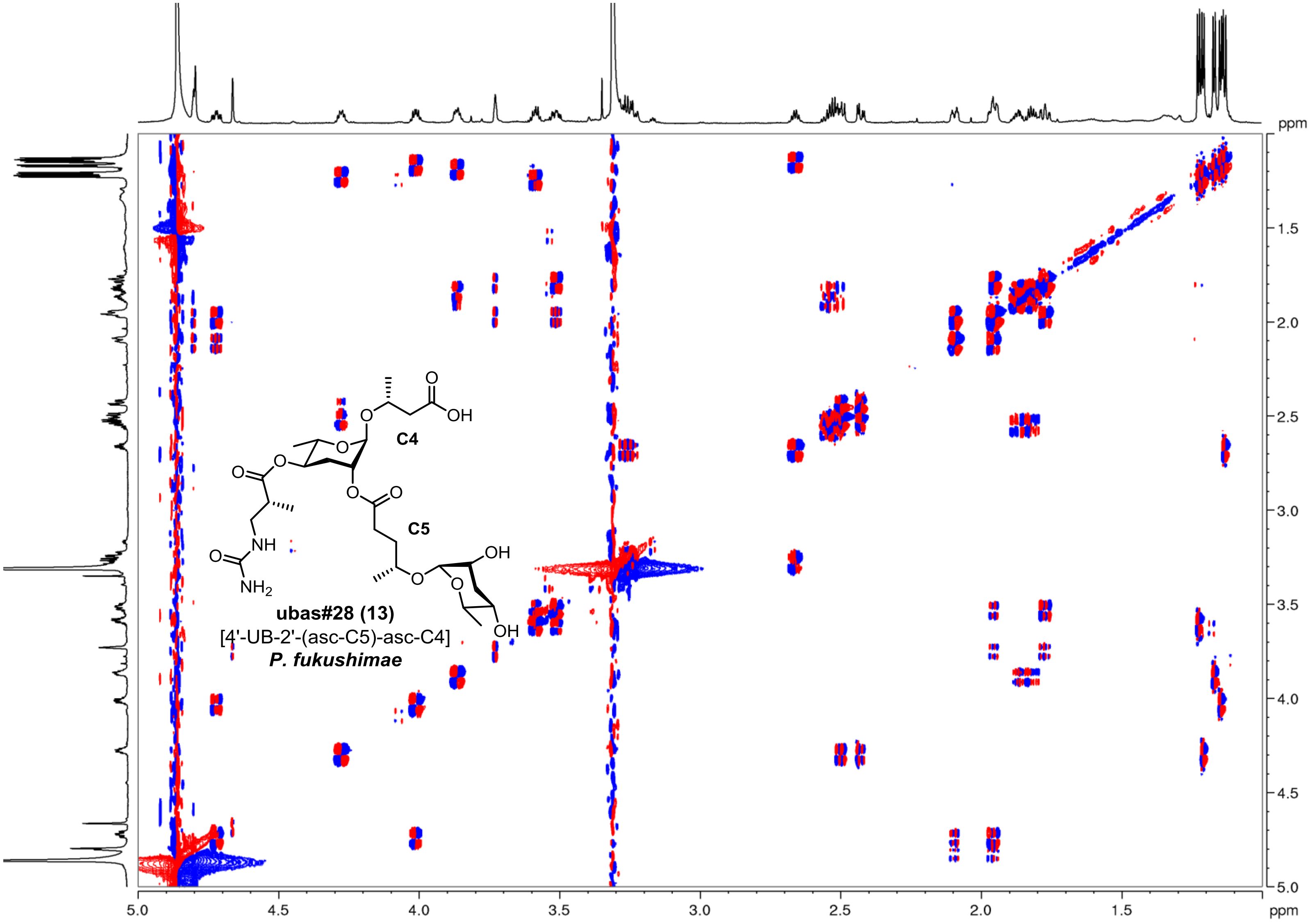

Figure 38. *dqf*-COSY spectrum of ubas#28 [4'-UB-2'-(asc-C5)-asc-C4, **13**] (800 MHz, CD_3OD) isolated from the *exo*-metabolome of *P. fukushimae*.

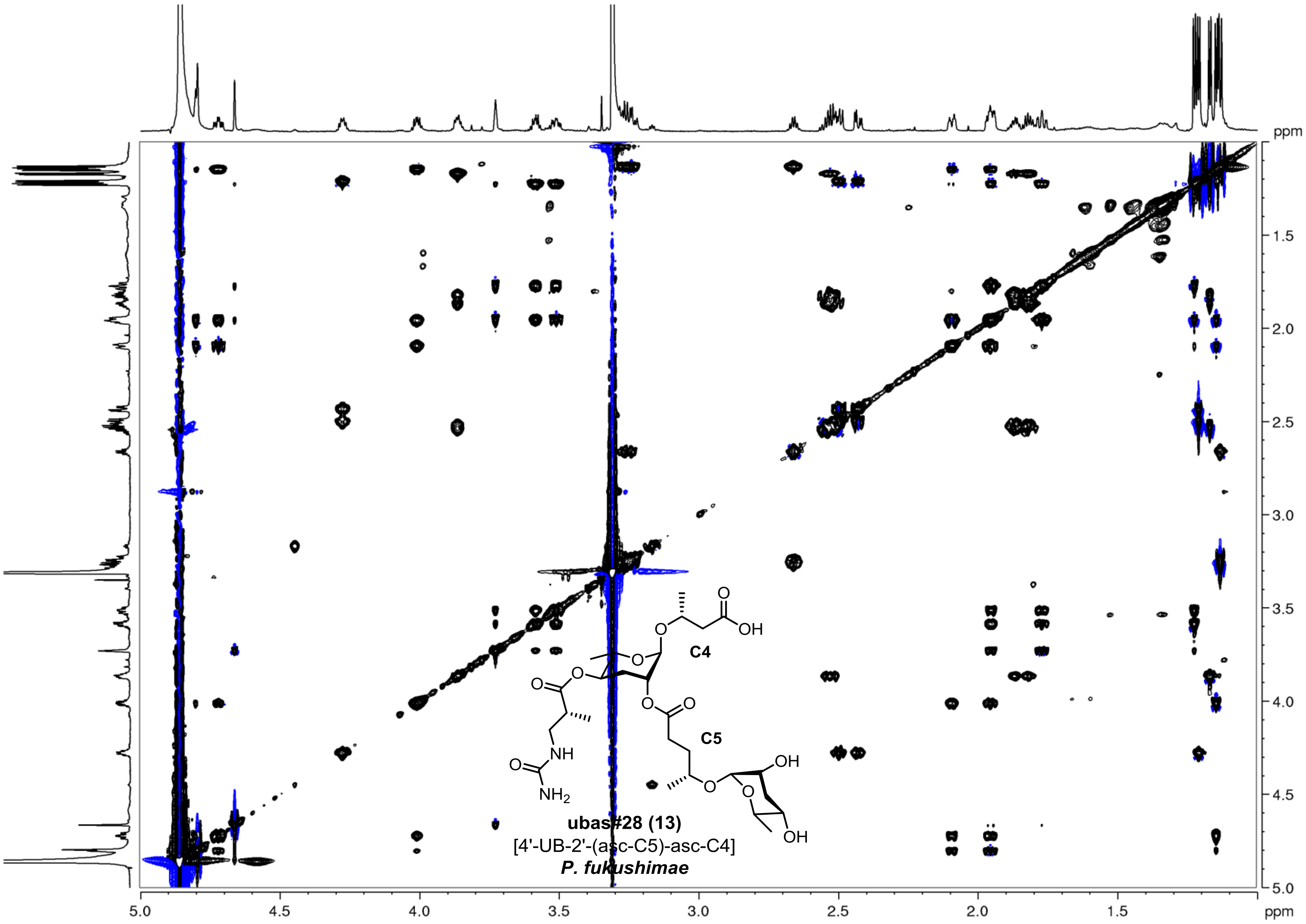

Figure 39. TOCSY spectrum of ubas#28 [4'-UB-2'-(asc-C5)-asc-C4, **13**] (800 MHz, CD_3OD) isolated from the *exo*-metabolome of *P. fukushimae*.

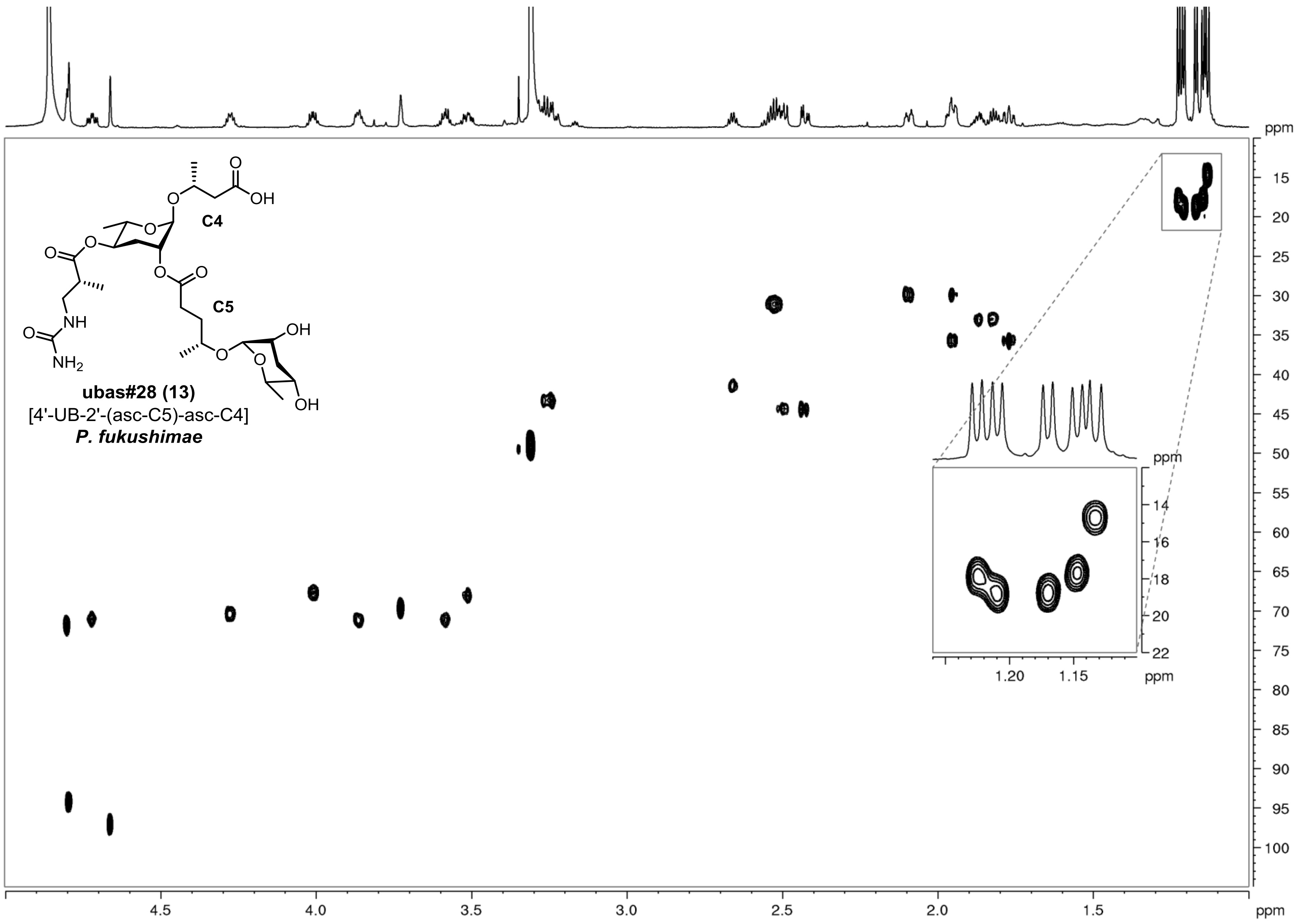

Figure 40. HSQC spectrum of ubas#28 [4'-UB-2'-(asc-C5)-asc-C4, **13**] (800 MHz, CD_3OD) isolated from the *exo*-metabolome of *P. fukushimae*.

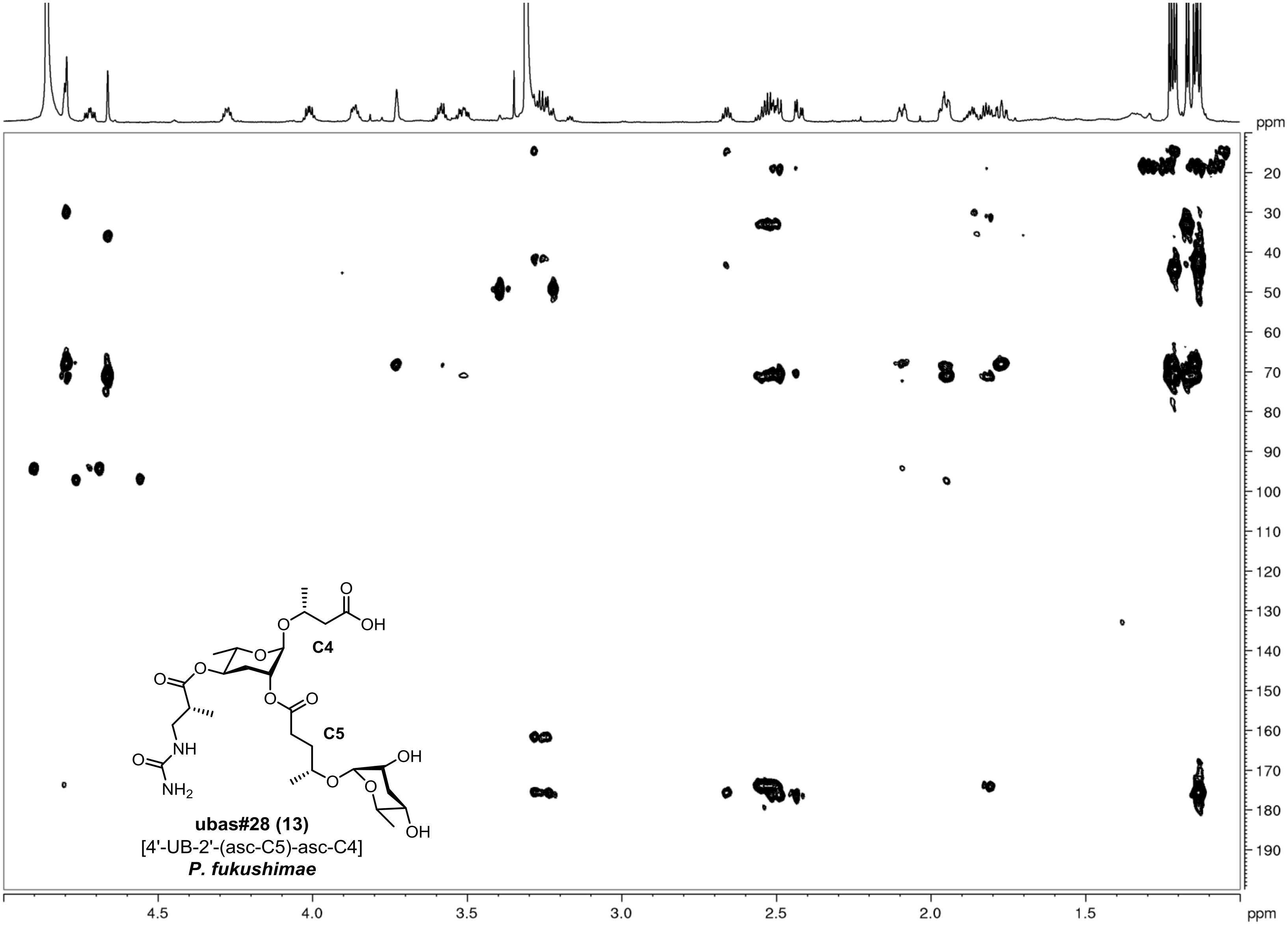

Figure 41. HMBC spectrum of ubas#28 [4'-UB-2'-(asc-C5)-asc-C4, **13**] (800 MHz, CD_3OD) isolated from the *exo*-metabolome of *P. fukushimae*.

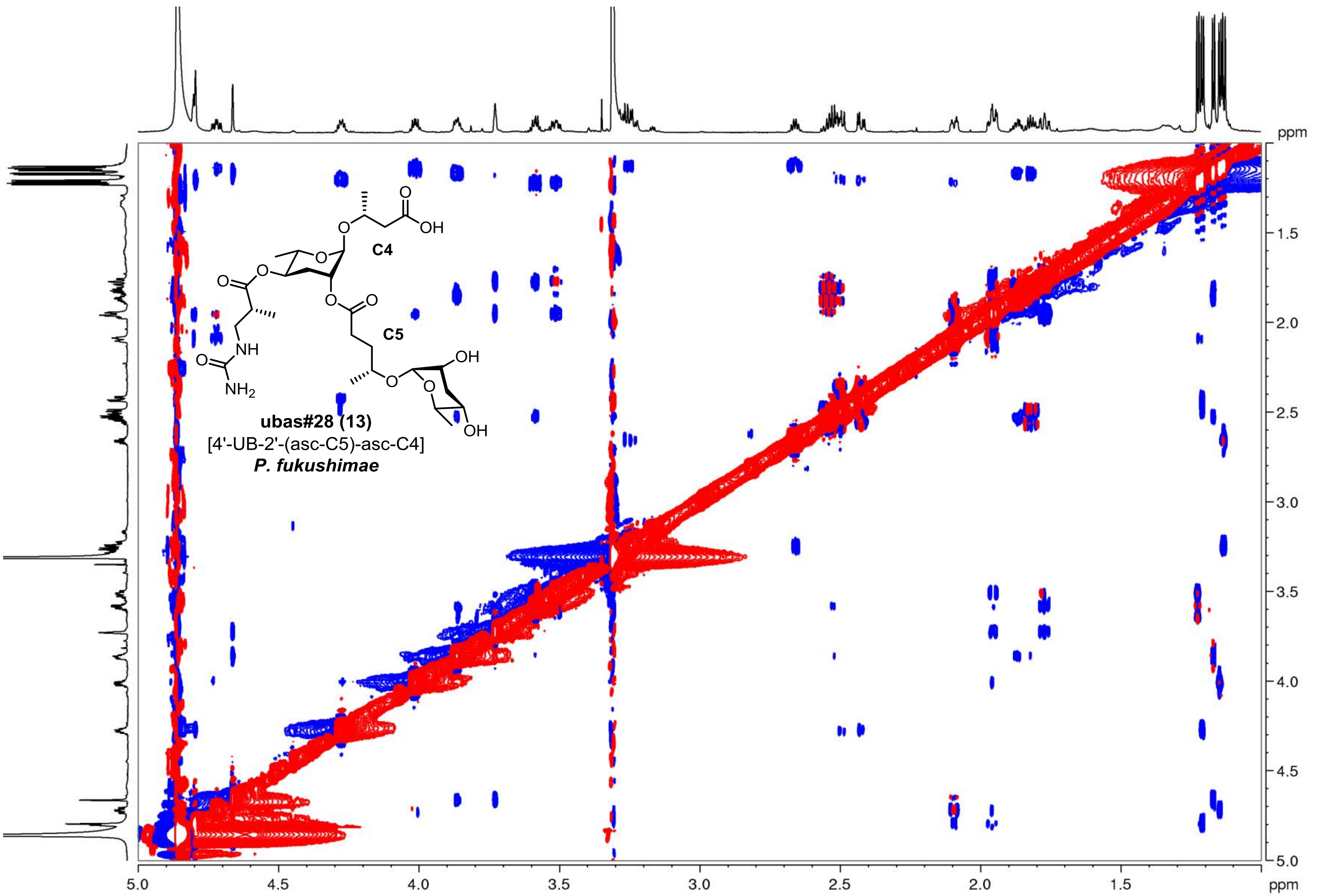

Figure 42. NOESY spectrum of ubas#28 [4'-UB-2'-(asc-C5)-asc-C4, **13**] (800 MHz, CD_3OD) isolated from the *exo*-metabolome of *P. fukushimae*.

supplementary file 1e: NMR spectra of UPAS chemicals Pages

Figure 1. NMR spectra of an HPLC enriched fraction containing upas#34 (**14**). S133

Figure 2. NMR spectra of an HPLC enriched fraction containing upas#28 (**15**). S134

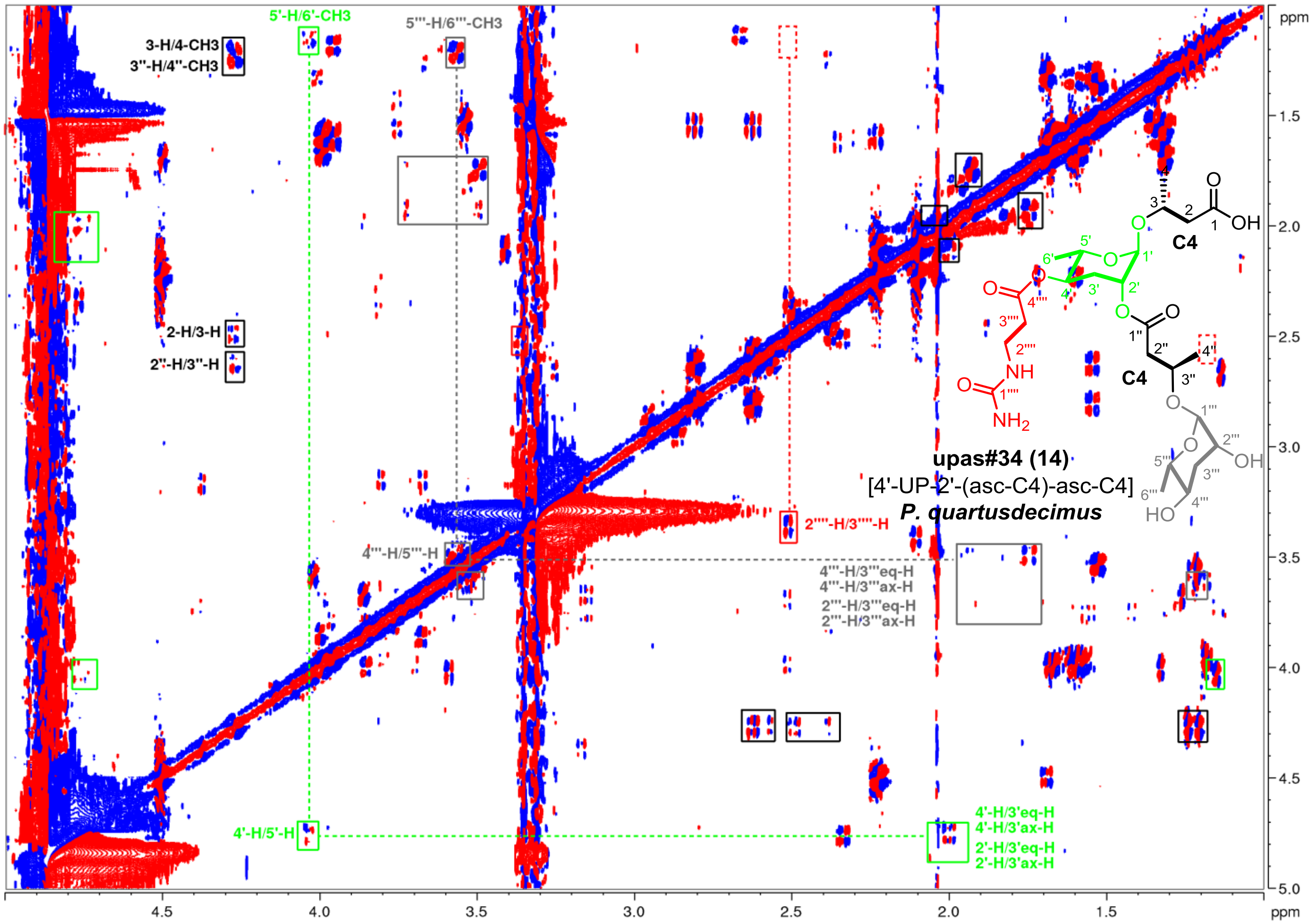

Figure 1. *dqf*-COSY spectrum of an HPLC enriched sample containing upas#34 [4'-UP-2'-(asc-C4)-asc-C4, **14**] (800 MHz, CD_3OD) isolated from the *exo*-metabolome of *P. quartusdecimus*. Key ^1H, ^1H-COSY correlation signals of two ω-1 style C4 fatty acid side chains (black boxed signals) and two characteristic ascarylose sugars (green and grey boxed signals are derived from the 2',4'-substituted ascarylose sugar and free ascarylose sugar, respectively) are observed in the spectrum. The key ^1H, ^1H-COSY correlation signal between H-2'''' at 3.36 ppm and H-3'''' at 2.50 ppm suggests that upas#34 (**14**) contains a ureidopropionic acid building block, which is most likely attached to the 4'-position (red dashed line boxed signals highlight the disappearance of one methyl group by comparing with the ureidoisobutyric acid bulding block from UBAS chemicals).

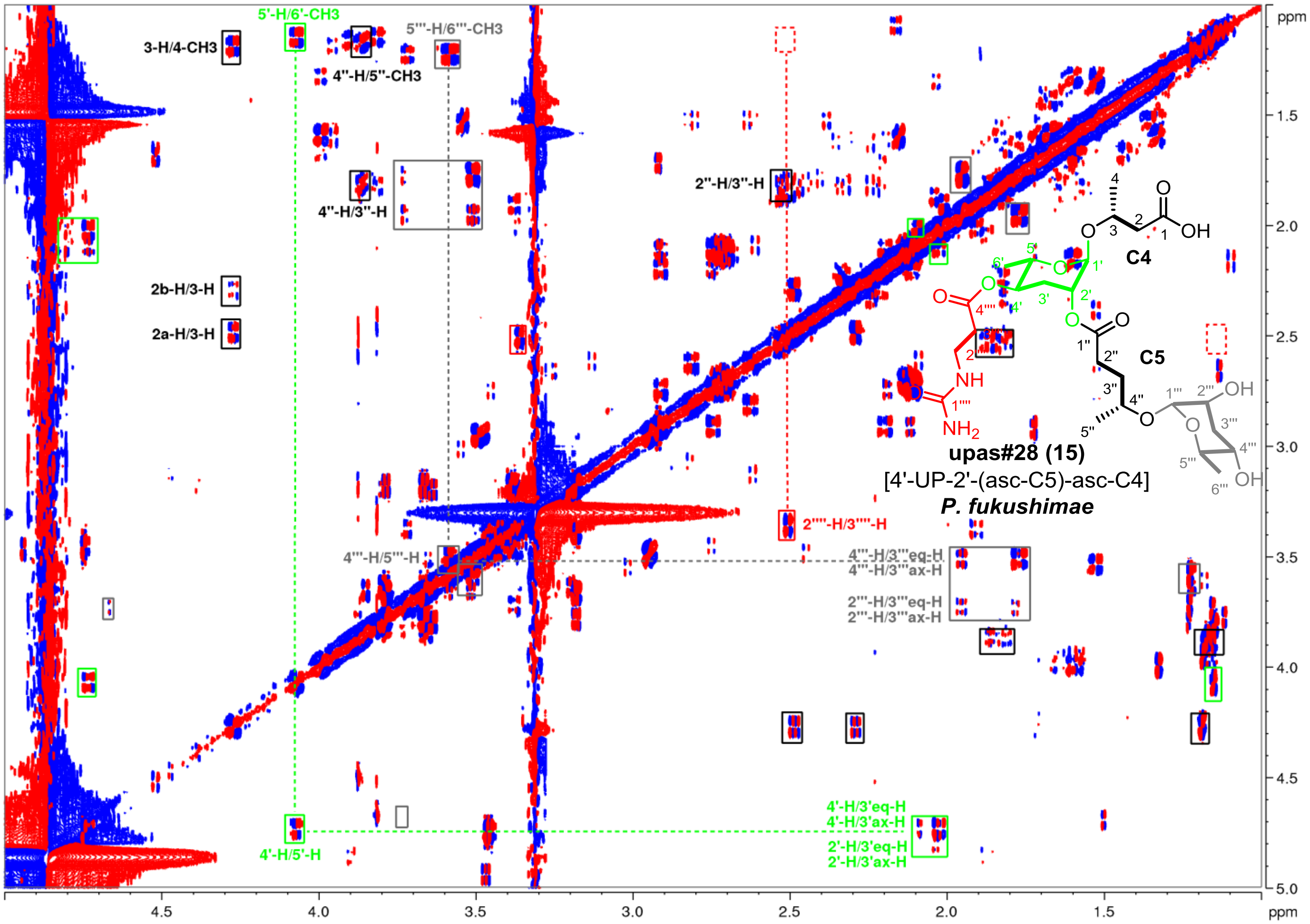

Figure 2. *dqf*-COSY spectrum of an HPLC enriched sample containing upas#28 [4'-UP-2'-(asc-C5)-asc-C4, **15**] (800 MHz, CD_3OD) isolated from *P. fukushimae exo*-metabolome. Key ^1H, ^1H-COSY correlation signals of C4 and C5 fatty acid side chains (black boxed signals) and two characteristic ascarylose sugars (green and grey boxed signals are derived from the 2',4'-substituted ascarylose sugar and free ascarylose sugar, respectively) are observed in the spectrum. The key ^1H, ^1H-COSY correlation signal between H-2'''' at 3.36 ppm and H-3'''' at 2.51 ppm suggests that upas#28 (**15**) contains a ureidopropionic acid building block, which is distinguishable from the ureidoisobutyric acid bulding block in UBAS chemicals by the disapearance of one methyl group in the ureidopropionic acid building block (red dashed line boxed signals).
