# Supplementary material for: Convergent evolution of small molecule pheromones in Pristionchus nematodes
Source: eLife. 2020 Apr 27;9:e55687. doi: 10.7554/eLife.55687 (PMC7224695; doi:10.7554/eLife.55687)
Supplement: Supplementary file 2. — a. MS/MS spectral data of DASC chemicals. b. MS/MS spectral data of PASC chemicals. c. MS/MS spectral data of UBAS chemicals. d. MS/MS spectral data of UPAS chemicals. [file elife-55687-supp2.pdf]

**supplementary file 2: MS/MS spectral data of NDMMs**	Pages
**supplementary file 2a:** MS/MS spectral data of DASC chemicals	S2
**supplementary file 2b:** MS/MS spectral data of PASC chemicals	S23
**supplementary file 2c:** MS/MS spectral data of UBAS chemicals	S25
**supplementary file 2d:** MS/MS spectral data of UPAS chemicals	S47
**References**	S55

supplementary file 2a: MS/MS spectral data of DASC chemicals Pages

Figure 1. LC-HR(ESI$^{-/+}$)-MS/MS spectral data of dasc#3 [2'-(asc-C5)-asc-C4, **1**]. S3
Figure 2. LC-HR(ESI$^{-/+}$)-MS/MS spectral data of dasc#4 (**2**) from *P. taiwanensis*. S4
Figure 3. LC-HR(ESI$^{-/+}$)-MS/MS spectral data of dasc#4 (**2**) from *P. entomophagus*. S5
Figure 4. LC-HR(ESI$^{-/+}$)-MS/MS spectral data of dasc#4 (**2**) from *P. pacificus*. S6
Figure 5. LC-HR(ESI$^{-/+}$)-MS/MS spectral data of dasc#5 (**3**) from *P. mayeri*. S7
Figure 6. LC-HR(ESI$^{-/+}$)-MS/MS spectral data of dasc#5 (**3**) from *D. magnus*. S8
Figure 7. LC-HR(ESI$^{-/+}$)-MS/MS spectral data of dasc#6 [2'-(asc-C6)-asc-C5, **4**]. S9
Figure 8. LC-HR(ESI$^{-/+}$)-MS/MS spectral data of dasc#9 [2'-(asc-C7)-asc-C5]. S10
Figure 9. LC-HR(ESI$^{-/+}$)-MS/MS spectral data of dasc#10 [4'-(asc-C5)-asc-C7, **5**]. S11
Figure 10. LC-HR(ESI$^{-/+}$)-MS/MS spectral data of dasc#12 [4'-(asc-C6)-asc-C7]. S12
Figure 11. LC-HR(ESI$^{-/+}$)-MS/MS spectral data of dasc#1 [4'-(asc-C7)-asc-C7]. S13
Figure 12. LC-HR(ESI$^{-/+}$)-MS/MS spectral data of dasc#2 [2'/4'-(asc-C4)-asc-C4]. S14
Figure 13. LC-HR(ESI$^{-/+}$)-MS/MS spectral data of dasc#7 [2'/4'-(asc-C7)-asc-C4]. S15
Figure 14. LC-HR(ESI$^{-/+}$)-MS/MS spectral data of dasc#8 [2'/4'-(asc-C6)-asc-C6]. S16
Figure 15. LC-HR(ESI$^{-/+}$)-MS/MS spectral data of dasc#11 [2'/4'-(asc-C7)-asc-C6]. S17
Figure 16. LC-HR(ESI$^{-/+}$)-MS/MS spectral data of dasc#13 [2'/4'-(asc-C5)-asc-C9]. S18
Figure 17. LC-HR(ESI$^{-/+}$)-MS/MS spectral data of dasc#14 [4'-(asc-ΔC7)-asc-C5, **6**]. S19
Figure 18. LC-HR(ESI$^{-/+}$)-MS/MS spectral data of dasc#15 [4'-(asc-ΔC7)-asc-C6]. S20
Figure 19. LC-HR(ESI$^{-/+}$)-MS/MS spectral data of dasc#16 [4'-(asc-ΔC7)-asc-C7, **7**]. S21
Figure 20. LC-HR(ESI$^{-/+}$)-MS/MS spectral data of dasc#17 [4'-(asc-ΔC7)-asc-ΔC7, **8**]. S22

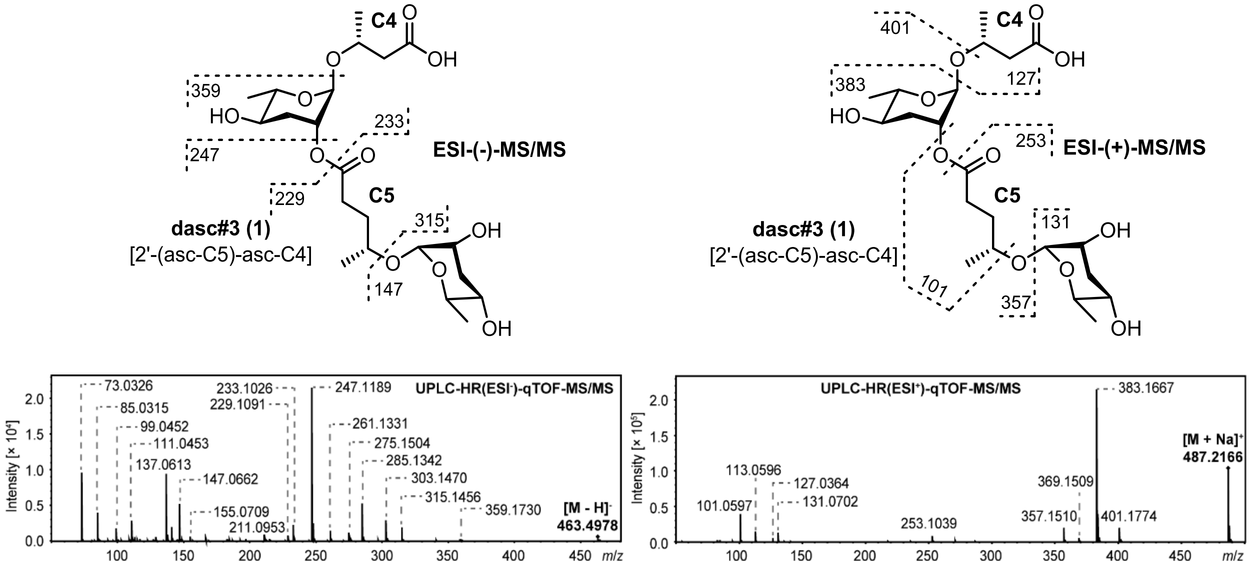

UPLC-HR(ESI⁻)-qTOF-MS/MS			
Obs. [m/z]	Calc. [m/z]	Error [ppm]	Ion Formula $[M-H]^-$
463.2178	463.2185	1.4	$C_{21}H_{35}O_{11}^-$
359.1730	359.1711	-5.2	$C_{17}H_{27}O_8^-$
315.1456	315.1449	-2.1	$C_{15}H_{23}O_7^-$
303.1470	303.1449	-6.8	$C_{14}H_{23}O_7^-$
285.1342	285.1344	0.5	$C_{14}H_{21}O_6^-$
275.1504	275.1500	-1.3	$C_{13}H_{23}O_6^-$
261.1331	261.1344	4.8	$C_{12}H_{21}O_6^-$
247.1189	247.1187	-0.6	$C_{11}H_{19}O_6^-$
233.1026	233.1031	2.2	$C_{10}H_{17}O_6^-$
229.1091	229.1081	-4.2	$C_{11}H_{17}O_5^-$
211.0953	211.0976	11.0	$C_{11}H_{15}O_4^-$
155.0709	155.0714	2.8	$C_8H_{11}O_3^-$
147.0662	147.0663	0.9	$C_6H_{11}O_4^-$
137.0613	137.0608	-3.3	$C_8H_9O_2^-$
111.0453	111.0452	-1.6	$C_6H_7O_2^-$
99.0452	99.0452	-0.7	$C_5H_7O_2^-$
85.0315	85.0295	-	$C_4H_5O_2^-$
73.0326	73.0295	-	$C_3H_5O_2^-$

UPLC-HR(ESI⁺)-qTOF-MS/MS			
Obs. [m/z]	Calc. [m/z]	Error [ppm]	Ion Formula $[M+Na]^+$
487.2137	487.2150	2.6	$C_{21}H_{36}NaO_{11}^+$
401.1774	401.1782	2.1	$C_{17}H_{30}NaO_9^+$
383.1667	383.1676	2.4	$C_{17}H_{28}NaO_8^+$
369.1509	369.1520	2.8	$C_{16}H_{26}NaO_8^+$
357.1510	357.1520	2.9	$C_{15}H_{26}NaO_8^+$
253.1039	253.1046	3.1	$C_{11}H_{18}NaO_5^+$
131.0702	131.0703	0.9	$C_6H_{11}O_3^+$
127.0364	127.0366	0.9	$C_4H_8NaO_3^+$
113.0596	113.0573	-	$C_4H_{10}NaO_2^+$
101.0597	101.0597	0.5	$C_5H_9O_2^+$

Figure 1. UPLC-HR(ESI$^{-/+}$)-qTOF-MS/MS mass spectral data of dasc#3 [2'-(asc-C5)-asc-C4, **1**] in both negative and positive ion modes. MS/MS fragmentation in positive ion mode produced an intensive ion signal of $C_{17}H_{28}NaO_8^+$ (m/z = 383.1667) suggesting that the first ascaroside (LC-MS/MS fragmentation pattern of dimeric ascarosides were described in ***Figure 4 – figure supplement 1C***) in dasc#3 [2'-(asc-C5)-asc-C4, **1**] should be ascr#9 [asc-C5] or oasc#9 [asc-ωC5] (***von Reuss et al., 2012***). *dqf*-COSY spectrum of an HPLC enriched sample containg dasc#3 [2'-(asc-C5)-asc-C4, **1**] confirmed that a unit of ascr#9 [asc-C5] was linked the 2'-position of ascr#11 [asc-C4] (***supplementary file 1b – Figure 1***).

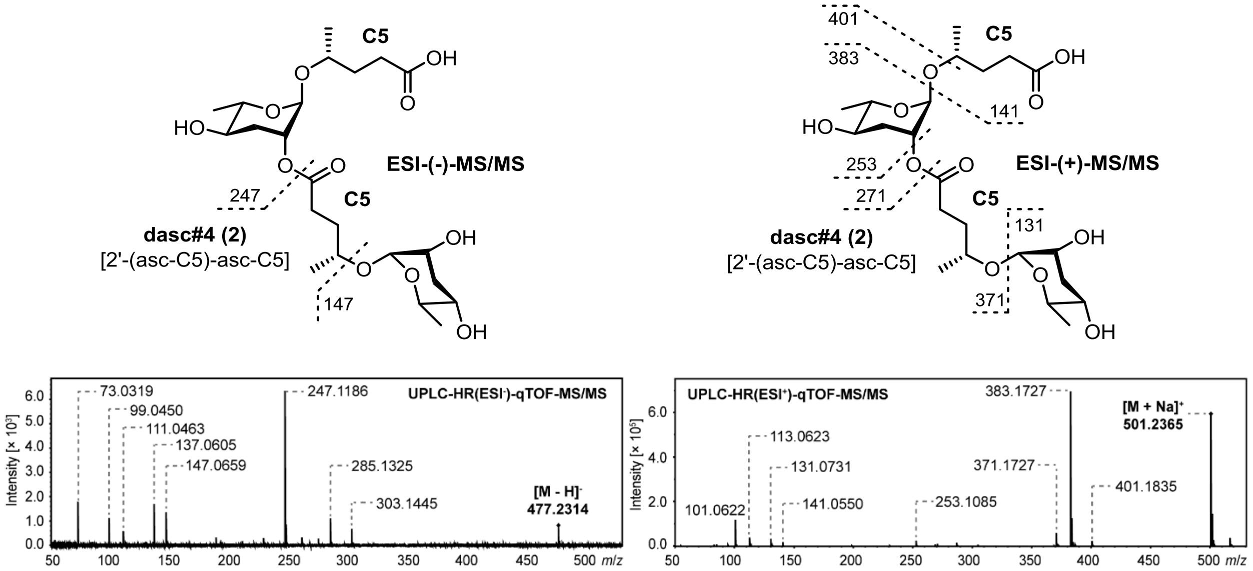

UPLC-HR(ESI⁻)-qTOF-MS/MS			
Obs. [*m*/*z*]	Calc. [*m*/*z*]	Error [ppm]	Ion Formula $[M-H]^-$
477.2314	477.2341	2.8	$C_{22}H_{37}O_{11}^-$
303.1445	303.1449	0.5	$C_{14}H_{23}O_7^-$
285.1325	285.1344	1.8	$C_{14}H_{21}O_6^-$
247.1168	247.1187	1.9	$C_{11}H_{19}O_6^-$
147.0653	147.0663	1.0	$C_6H_{11}O_4^-$
137.0602	137.0608	0.6	$C_8H_9O_2^-$
111.0450	111.0452	0.1	$C_6H_7O_2^-$
99.0451	99.0452	0.1	$C_5H_7O_2^-$
73.0314	73.0295	-1.9	$C_3H_5O_2^-$

UPLC-HR(ESI⁺)-qTOF-MS/MS			
Obs. [*m*/*z*]	Calc. [*m*/*z*]	Error [ppm]	Ion Formula $[M+Na]^+$
501.2365	501.2306	-11.7	$C_{22}H_{38}N_aO_{11}^+$
401.1835	401.1782	-13.2	$C_{17}H_{30}N_aO_9^+$
383.1727	383.1676	-13.1	$C_{17}H_{28}N_aO_8^+$
371.1727	371.1676	-13.5	$C_{16}H_{28}N_aO_8^+$
253.1085	253.1046	-15.4	$C_{11}H_{18}N_aO_5^+$
141.0550	141.0522	-20.1	$C_5H_{10}N_aO_3^+$
131.0731	131.0703	-21.7	$C_6H_{11}O_3^+$
113.0623	113.0597	-23.3	$C_6H_9O_2^+$
101.0622	101.0597	-24.2	$C_5H_9O_2^+$

Figure 2. UPLC-HR(ESI$^{-/+}$)-qTOF-MS/MS mass spectral data of dasc#4 [2'-(asc-C5)-asc-C5, **2**] from *P. taiwanensis* in both negative and positive ion modes.

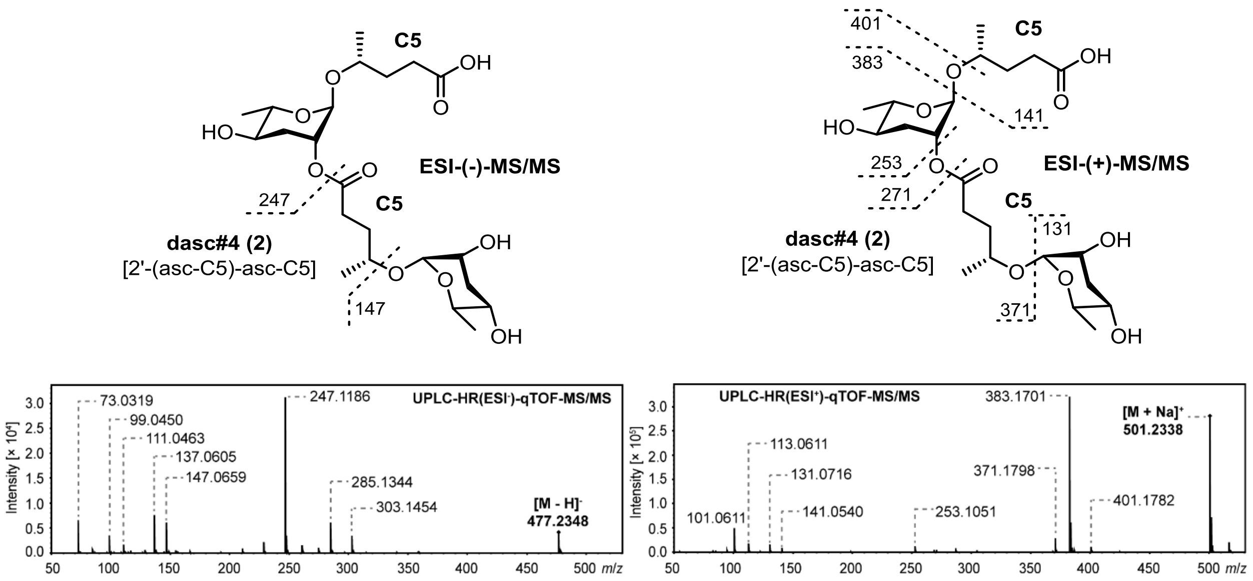

UPLC-HR(ESI$^-$)-qTOF-MS/MS			
Obs. [*m*/*z*]	Calc. [*m*/*z*]	Error [ppm]	Ion Formula [M − H]$^-$
477.2348	477.2341	-1.5	$C_{22}H_{37}O_{11}^-$
303.1454	303.1449	-1.7	$C_{14}H_{23}O_7^-$
285.1344	285.1344	0.0	$C_{14}H_{21}O_6^-$
247.1186	247.1187	0.3	$C_{11}H_{19}O_6^-$
147.0659	147.0663	2.8	$C_6H_{11}O_4^-$
137.0605	137.0608	2.2	$C_8H_9O_2^-$
111.0463	111.0452	-10.3	$C_6H_7O_2^-$
99.0450	99.0452	1.7	$C_5H_7O_2^-$
73.0319	73.0295	-32.6	$C_3H_5O_2^-$

UPLC-HR(ESI$^+$)-qTOF-MS/MS			
Obs. [*m*/*z*]	Calc. [*m*/*z*]	Error [ppm]	Ion Formula [M + Na]$^+$
501.2338	501.2306	-6.3	$C_{22}H_{38}N_aO_{11}^+$
401.1805	401.1782	-5.9	$C_{17}H_{30}N_aO_9^+$
383.1701	383.1676	-6.5	$C_{17}H_{28}N_aO_8^+$
371.1698	371.1676	-5.7	$C_{16}H_{28}N_aO_8^+$
253.1051	253.1046	-1.7	$C_{11}H_{18}N_aO_5^+$
141.0540	141.0522	-12.7	$C_5H_{10}N_aO_3^+$
131.0716	131.0703	-10.1	$C_6H_{11}O_3^+$
113.0611	113.0597	-12.1	$C_6H_9O_2^+$
101.0611	101.0597	-13.7	$C_5H_9O_2^+$

Figure 3. UPLC-HR(ESI$^{-/+}$)-qTOF-MS/MS mass spectral data of dasc#4 [2'-(asc-C5)-asc-C5, **2**] from *P. entomophagus* in both negative and positive ion modes.

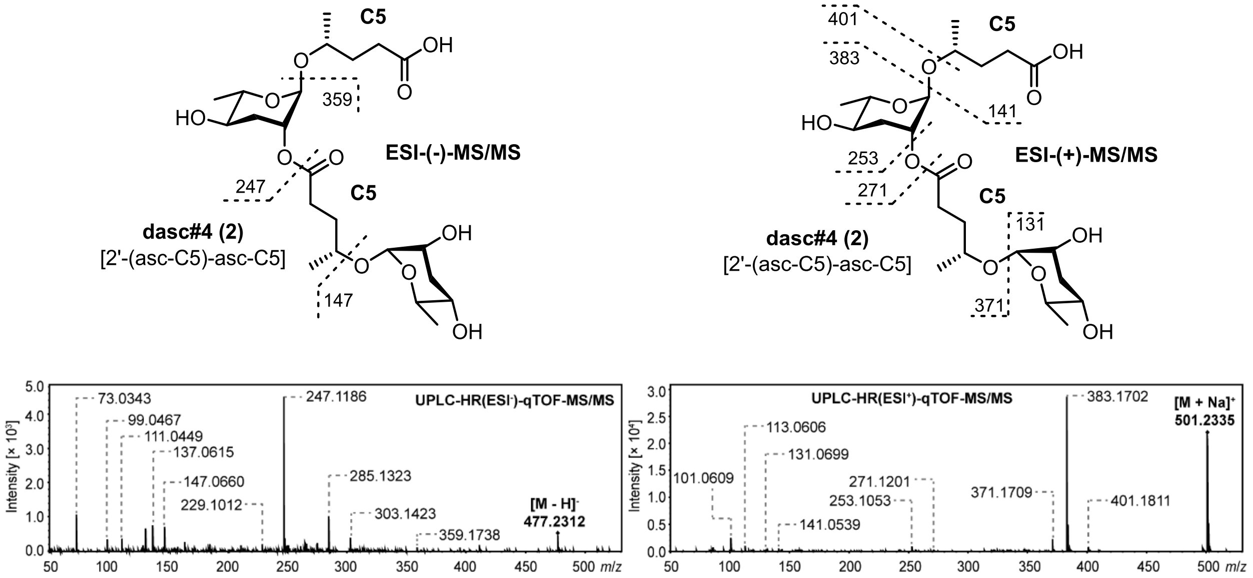

UPLC-HR(ESI⁻)-qTOF-MS/MS			
Obs. [*m*/*z*]	Calc. [*m*/*z*]	Error [ppm]	Ion Formula [M − H]⁻
477.2312	477.2341	-6.2	$C_{22}H_{37}O_{11}^-$
359.1738	359.1711	-7.3	$C_{17}H_{27}O_8^-$
303.1423	303.1449	8.7	$C_{14}H_{23}O_7^-$
285.1323	285.1344	7.2	$C_{14}H_{21}O_6^-$
247.1186	247.1187	0.3	$C_{11}H_{19}O_6^-$
229.1012	229.1081	-	$C_{11}H_{17}O_5^-$
147.0660	147.0663	2.3	$C_6H_{11}O_4^-$
137.0615	137.0608	-4.9	$C_8H_9O_2^-$
111.0449	111.0452	2.4	$C_6H_7O_2^-$
99.0467	99.0452	-	$C_5H_7O_2^-$
73.0343	73.0295	-	$C_3H_5O_2^-$

UPLC-HR(ESI⁺)-qTOF-MS/MS			
Obs. [*m*/*z*]	Calc. [*m*/*z*]	Error [ppm]	Ion Formula [M + Na]⁺
501.2335	501.2306	0.0	$C_{22}H_{38}N_aO_{11}^+$
401.1811	401.1782	-7.1	$C_{17}H_{30}N_aO_9^+$
383.1702	383.1676	-6.8	$C_{17}H_{28}N_aO_8^+$
371.1709	371.1676	-8.7	$C_{16}H_{28}N_aO_8^+$
271.1201	271.1152	-	$C_{11}H_{20}N_aO_6^+$
253.1053	253.1046	-2.7	$C_{11}H_{18}N_aO_5^+$
141.0539	141.0522	-	$C_5H_{10}N_aO_3^+$
131.0699	131.0703	2.5	$C_6H_{11}O_3^+$
113.0606	113.0597	-7.8	$C_6H_9O_2^+$
101.0609	101.0597	-	$C_5H_9O_2^+$

Figure 4. UPLC-HR(ESI$^{-/+}$)-qTOF-MS/MS mass spectral data of dasc#4 [2'-(asc-C5)-asc-C5, **2**] from *P. pacificus* RS2333 in both negative and positive ion modes.

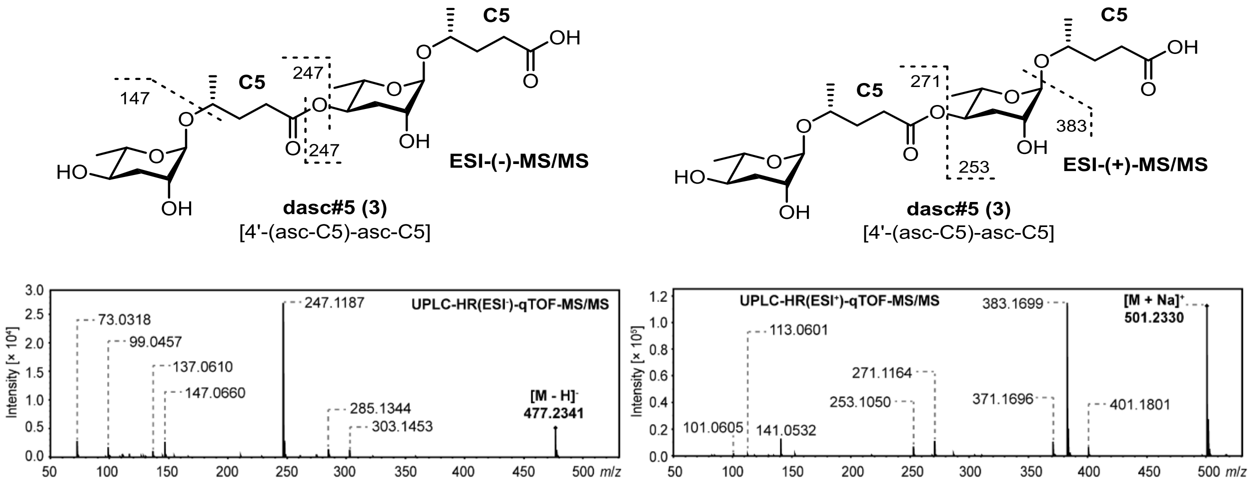

UPLC-HR(ESI$^-$)-qTOF-MS/MS			
Obs. [*m/z*]	Calc. [*m/z*]	Error [ppm]	Ion Formula [M − H]$^-$
477.2341	477.2341	0.1	$C_{22}H_{37}O_{11}^-$
303.1453	303.1449	-0.3	$C_{14}H_{23}O_7^-$
285.1344	285.1344	-0.1	$C_{14}H_{21}O_6^-$
247.1187	247.1187	0.0	$C_{11}H_{19}O_6^-$
147.0660	147.0663	0.2	$C_6H_{11}O_4^-$
137.0610	137.0608	-0.2	$C_8H_9O_2^-$
99.0457	99.0452	-0.6	$C_5H_7O_2^-$
73.0318	73.0295	-2.3	$C_3H_5O_2^-$

UPLC-HR(ESI$^+$)-qTOF-MS/MS			
Obs. [*m/z*]	Calc. [*m/z*]	Error [ppm]	Ion Formula [M + Na]$^+$
501.2330	501.2306	-4.7	$C_{22}H_{38}N_aO_{11}^+$
401.1801	401.1782	-4.8	$C_{17}H_{30}N_aO_9^+$
383.1699	383.1676	-6.0	$C_{17}H_{28}N_aO_8^+$
371.1696	371.1676	-5.3	$C_{16}H_{28}N_aO_8^+$
271.1164	271.1152	-4.2	$C_{11}H_{20}N_aO_6^+$
253.1050	253.1046	-1.4	$C_{11}H_{18}N_aO_5^+$
141.0532	141.0522	-6.7	$C_5H_{10}N_aO_3^+$
113.0601	113.0597	-3.1	$C_6H_9O_2^+$
101.0605	101.0597	-	$C_5H_9O_2^+$

Figure 5. UPLC-HR(ESI$^{-/+}$)-qTOF-MS/MS mass spectral data of dasc#5 [4'-(asc-C5)-asc-C5, **3**] from *P. mayeri* in both negative and positive ion modes.

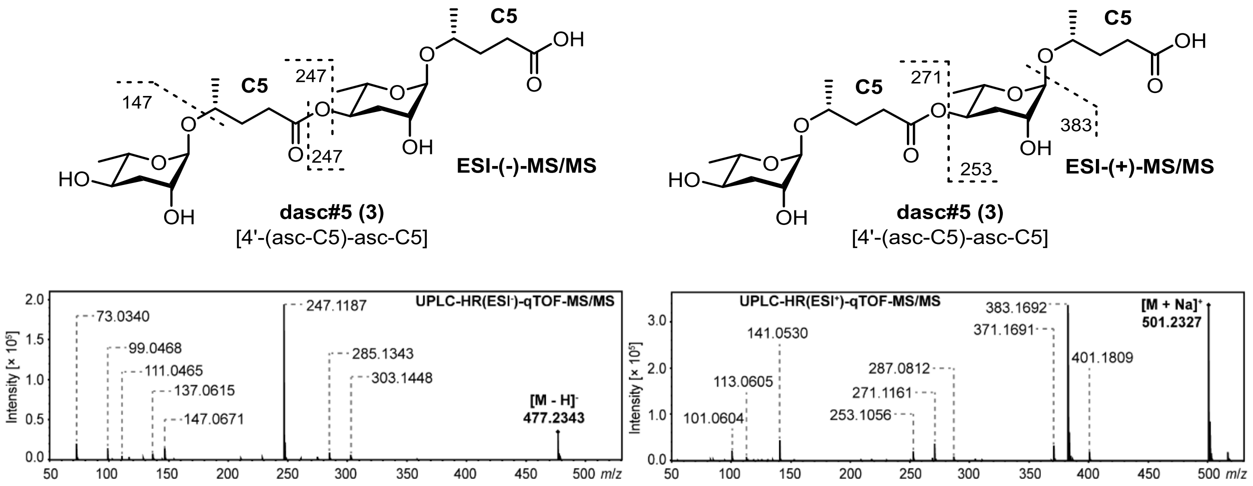

UPLC-HR(ESI⁻)-qTOF-MS/MS			
Obs. [*m*/*z*]	Calc. [*m*/*z*]	Error [ppm]	Ion Formula $[M-H]^-$
477.2343	477.2341	-0.3	$C_{22}H_{37}O_{11}^-$
303.1448	303.1449	0.4	$C_{14}H_{23}O_7^-$
285.1343	285.1344	0.2	$C_{14}H_{21}O_6^-$
247.1187	247.1187	0.2	$C_{11}H_{19}O_6^-$
147.0671	147.0663	-5.4	$C_6H_{11}O_4^-$
137.0615	137.0608	-5.2	$C_8H_9O_2^-$
111.0465	111.0452	-	$C_6H_7O_2^-$
99.0468	99.0452	-	$C_5H_7O_2^-$
73.0340	73.0295	-	$C_3H_5O_2^-$

UPLC-HR(ESI⁺)-qTOF-MS/MS			
Obs. [*m*/*z*]	Calc. [*m*/*z*]	Error [ppm]	Ion Formula $[M+Na]^+$
501.2327	501.2306	-4.1	$C_{22}H_{38}N_aO_{11}^+$
401.1809	401.1782	-6.7	$C_{17}H_{30}N_aO_9^+$
383.1692	383.1676	-4.2	$C_{17}H_{28}N_aO_8^+$
371.1691	371.1676	-4.0	$C_{16}H_{28}N_aO_8^+$
271.1161	271.1152	-3.3	$C_{11}H_{20}N_aO_6^+$
253.1056	253.1046	-3.8	$C_{11}H_{18}N_aO_5^+$
141.0530	141.0522	-5.9	$C_5H_{10}N_aO_3^+$
113.0605	113.0597	-7.4	$C_6H_9O_2^+$
101.0604	101.0597	-6.6	$C_5H_9O_2^+$

Figure 6. UPLC-HR(ESI$^{-/+}$)-qTOF-MS/MS mass spectral data of dasc#5 [4'-(asc-C5)-asc-C5, **3**] from *D. magnus* in both negative and positive ion modes.

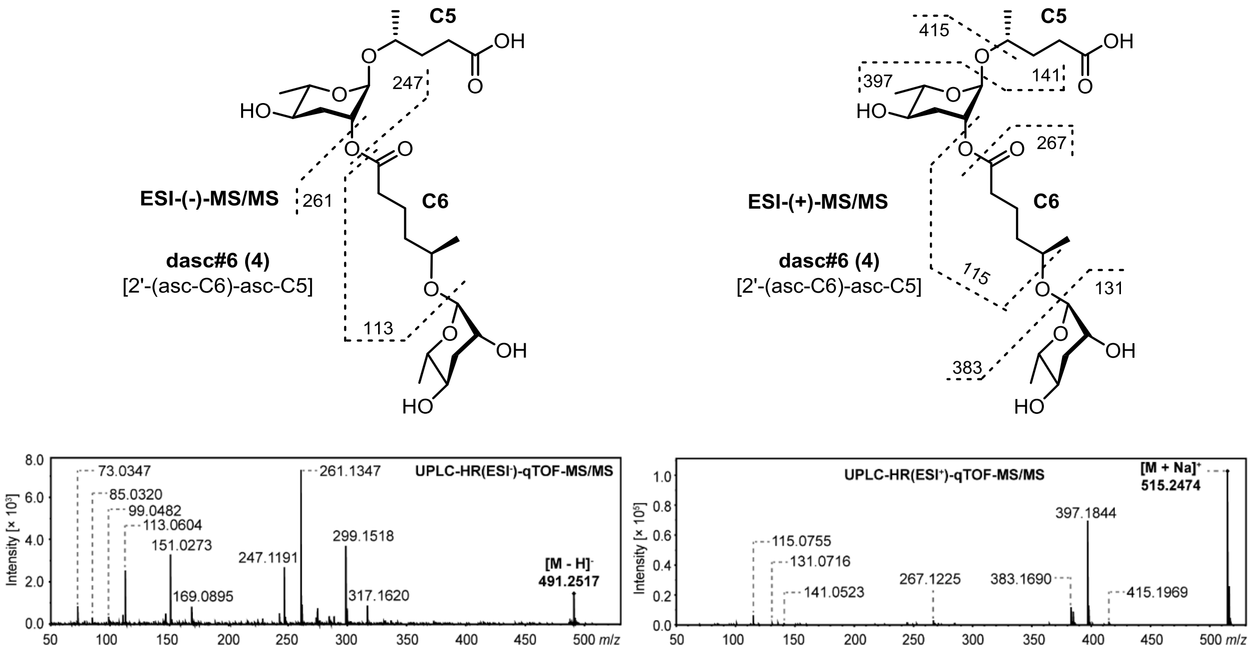

UPLC-HR(ESI$^-$)-qTOF-MS/MS			
Obs. [*m*/*z*]	Calc. [*m*/*z*]	Error [ppm]	Ion Formula [M − H]$^-$
491.2517	491.2498	-3.8	$C_{23}H_{39}O_{11}^-$
317.1620	317.1606	-4.5	$C_{15}H_{25}O_7^-$
299.1518	299.1500	-5.8	$C_{15}H_{23}O_6^-$
261.1347	261.1344	-1.2	$C_{12}H_{21}O_6^-$
247.1191	247.1187	-1.5	$C_{11}H_{19}O_6^-$
169.0895	169.0870	-	$C_9H_{13}O_3^-$
151.0273	151.0248	-	$C_4H_7O_6^-$
113.0604	113.0608	3.8	$C_6H_9O_2^-$
99.0482	99.0452	-	$C_5H_7O_2^-$
85.0320	85.0295	-	$C_4H_5O_2^-$
73.0347	73.0295	-	$C_3H_5O_2^-$

UPLC-HR(ESI$^+$)-qTOF-MS/MS			
Obs. [*m*/*z*]	Calc. [*m*/*z*]	Error [ppm]	Ion Formula [M + Na]$^+$
515.2474	515.2463	-2.2	$C_{23}H_{40}NaO_{11}^+$
415.1969	415.1939	-7.3	$C_{18}H_{32}NaO_9^+$
397.1844	397.1833	-2.8	$C_{18}H_{30}NaO_8^+$
383.1690	383.1676	-3.5	$C_{17}H_{28}NaO_8^+$
267.1225	267.1203	-	$C_{12}H_{20}NaO_5^+$
141.0523	141.0522	-0.4	$C_5H_{10}NaO_3^+$
131.0716	131.0703	-	$C_6H_{11}O_3^+$
115.0755	115.0754	-1.2	$C_6H_{11}O_2^+$

Figure 7. UPLC-HR(ESI$^{-/+}$)-qTOF-MS/MS mass spectral data of dasc#6 [2'-(asc-C6)-asc-C5, **4**] in both negative and positive ion modes.

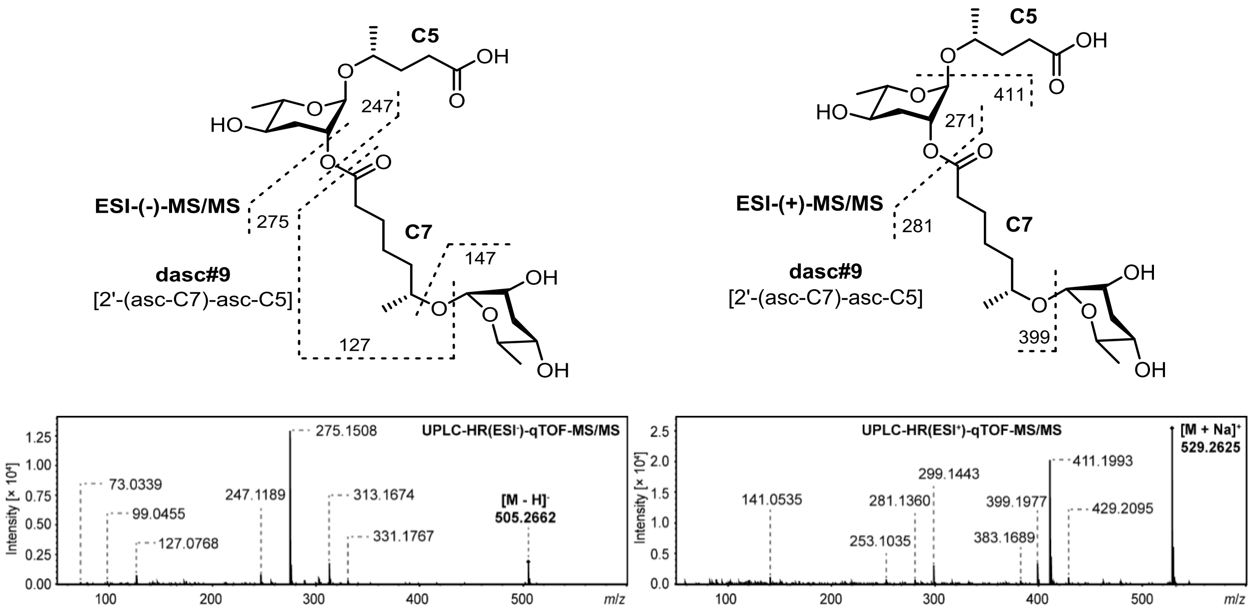

UPLC-HR(ESI⁻)-qTOF-MS/MS			
Obs. [*m/z*]	Calc. [*m/z*]	Error [ppm]	Ion Formula $[M-H]^-$
505.2662	505.2654	-1.5	$C_{24}H_{41}O_{11}^-$
331.1767	331.1762	-1.5	$C_{16}H_{27}O_7^-$
313.1674	313.1657	-5.4	$C_{16}H_{25}O_6^-$
275.1508	275.1500	-3.0	$C_{13}H_{23}O_6^-$
247.1189	247.1187	-0.9	$C_{11}H_{19}O_6^-$
127.0768	127.0765	-2.4	$C_7H_{11}O_2^-$
99.0455	99.0452	-3.3	$C_5H_7O_2^-$
73.0339	73.0295	-	$C_3H_5O_2^-$

UPLC-HR(ESI⁺)-qTOF-MS/MS			
Obs. [*m/z*]	Calc. [*m/z*]	Error [ppm]	Ion Formula $[M+Na]^+$
529.2625	529.2619	-1.0	$C_{24}H_{42}NaO_{11}^+$
429.2095	429.2095	-0.0	$C_{19}H_{34}NaO_9^+$
411.1993	411.1989	-0.8	$C_{19}H_{32}NaO_8^+$
399.1977	399.1989	3.2	$C_{18}H_{32}NaO_8^+$
383.1689	383.1676	-3.4	$C_{17}H_{28}NaO_8^+$
299.1443	299.1465	7.2	$C_{13}H_{24}NaO_6^+$
281.1360	281.1359	-0.2	$C_{13}H_{22}NaO_5^+$
253.1035	253.1046	4.7	$C_{11}H_{18}NaO_5^+$
141.0535	141.0522	-	$C_5H_{10}NaO_3^+$

Figure 8. UPLC-HR(ESI$^{-/+}$)-qTOF-MS/MS mass spectral data of dasc#9 [2'-(asc-C7)-asc-C5] in both negative and positive ion modes. Note that dasc#9 [2'-(asc-C7)-asc-C5] is tentatively assigned to be linked at the 2'-position, but its final structure has not yet been elucidated.

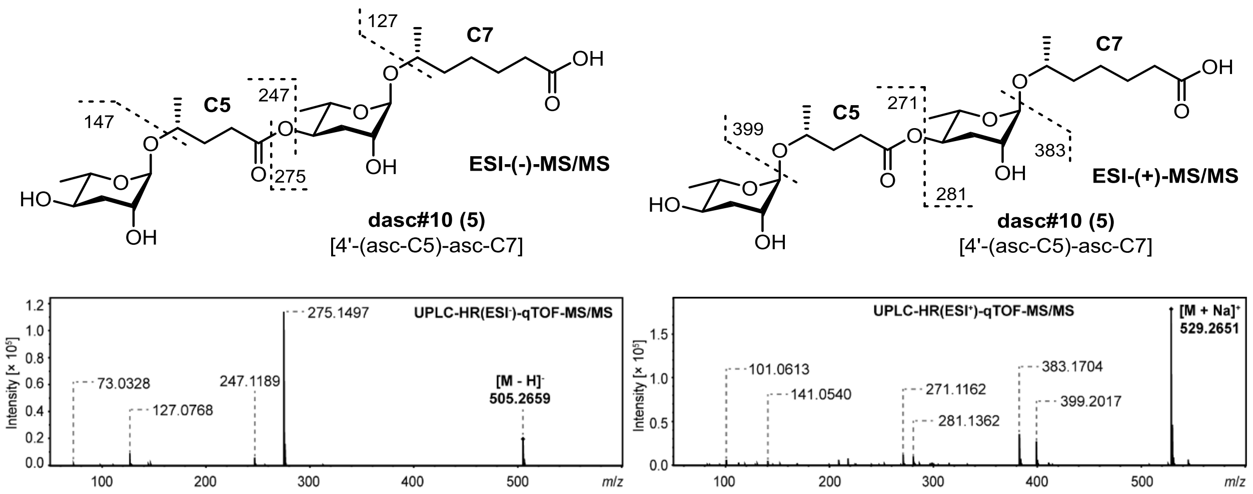

UPLC-HR(ESI⁻)-qTOF-MS/MS			
Obs. [*m*/*z*]	Calc. [*m*/*z*]	Error [ppm]	Ion Formula [M − H]$^-$
505.2659	505.2654	-0.9	$C_{24}H_{41}O_{11}^-$
275.1497	275.1500	1.0	$C_{13}H_{23}O_6^-$
247.1189	247.1187	-0.6	$C_{11}H_{19}O_6^-$
127.0768	127.0765	-2.8	$C_7H_{11}O_2^-$
73.0328	73.0295	-	$C_3H_5O_2^-$

UPLC-HR(ESI⁺)-qTOF-MS/MS			
Obs. [*m*/*z*]	Calc. [*m*/*z*]	Error [ppm]	Ion Formula [M + Na]$^+$
529.2651	529.2619	-6.0	$C_{24}H_{42}NaO_{11}^+$
399.2017	399.1989	-6.8	$C_{18}H_{32}NaO_8^+$
383.1704	383.1676	-7.1	$C_{17}H_{28}NaO_8^+$
281.1362	281.1359	-1.0	$C_{13}H_{22}NaO_5^+$
271.1162	271.1152	-3.7	$C_{11}H_{20}NaO_6^+$
141.0540	141.0522	-	$C_5H_{10}NaO_3^+$
101.0613	101.0597	-	$C_5H_9O_2^+$

Figure 9. UPLC-HR(ESI$^{-/+}$)-qTOF-MS/MS mass spectral data of dasc#10 [4'-(asc-C5)-asc-C7, **5**] in both negative and positive ion modes.

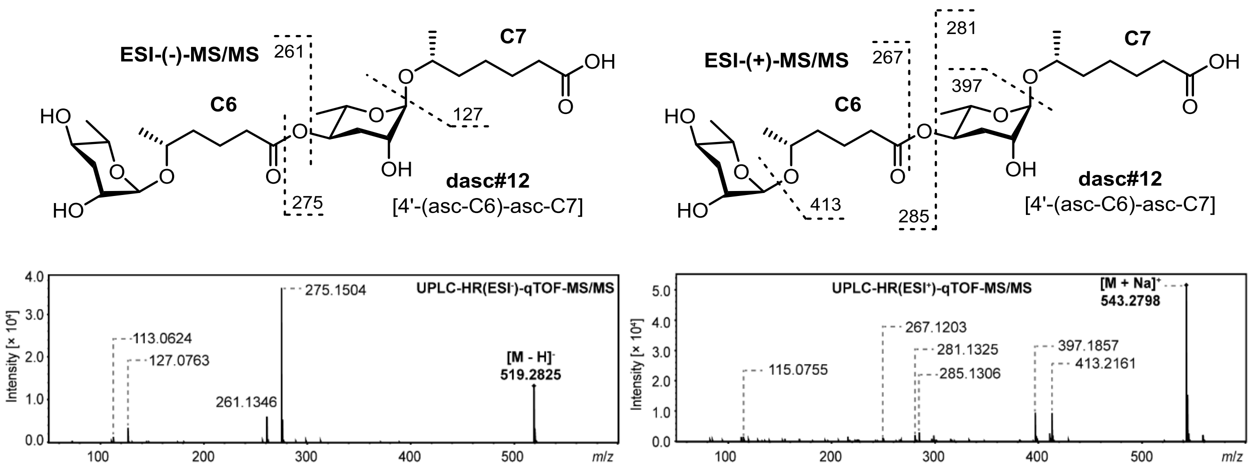

UPLC-HR(ESI^{-})-qTOF-MS/MS			
Obs. [*m*/*z*]	Calc. [*m*/*z*]	Error [ppm]	Ion Formula [M − H]$^{-}$
519.2825	519.2811	-2.8	$C_{25}H_{43}O_{11}^{-}$
275.1504	275.1500	-1.3	$C_{13}H_{23}O_{6}^{-}$
261.1346	261.1344	-0.8	$C_{12}H_{21}O_{6}^{-}$
127.0763	127.0765	1.0	$C_{7}H_{11}O_{2}^{-}$
113.0624	113.0608	-	$C_{6}H_{9}O_{2}^{-}$

UPLC-HR(ESI^{+})-qTOF-MS/MS			
Obs. [*m*/*z*]	Calc. [*m*/*z*]	Error [ppm]	Ion Formula [M + Na]$^{+}$
543.2798	543.2776	-4.1	$C_{25}H_{44}NaO_{11}^{+}$
413.2161	413.2146	-3.7	$C_{19}H_{34}NaO_{8}^{+}$
397.1857	397.1833	-6.1	$C_{18}H_{30}NaO_{8}^{+}$
285.1306	285.1309	0.9	$C_{12}H_{22}NaO_{6}^{+}$
281.1325	281.1359	12.2	$C_{13}H_{22}NaO_{5}^{+}$
267.1203	267.1203	0.0	$C_{12}H_{20}NaO_{5}^{+}$
115.0755	115.0754	-1.6	$C_{6}H_{11}O_{2}^{+}$

Figure 10. UPLC-HR(ESI$^{-/+}$)-qTOF-MS/MS mass spectral data of dasc#12 [4'-(asc-C6)-asc-C7] in both negative and positive ion modes. Note that dasc#12 [4'-(asc-C6)-asc-C7] is tentatively assigned to be linked at the 4'-position, but its absolute structure has not yet been elucidated.

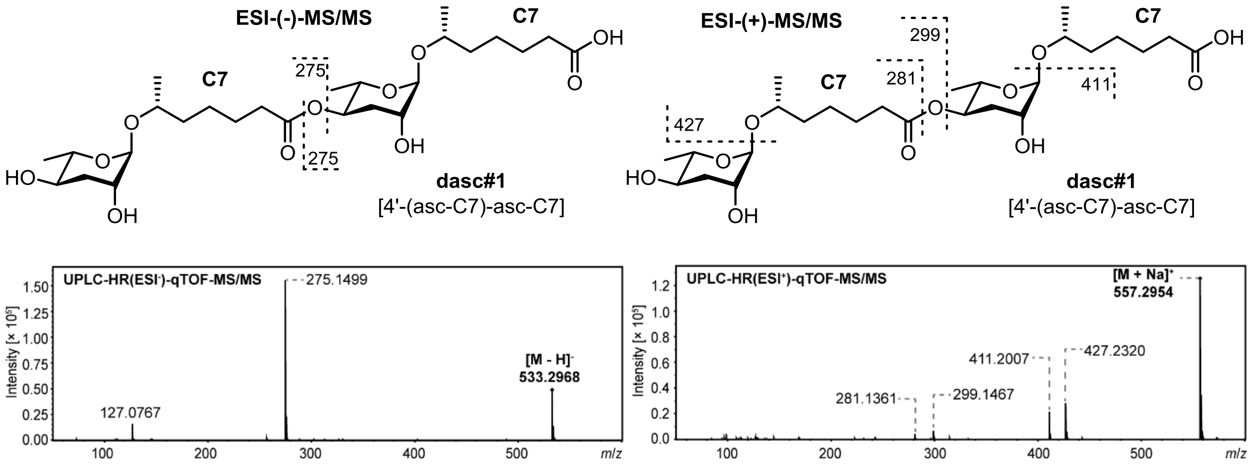

UPLC-HR(ESI$^-$)-qTOF-MS/MS			
Obs. [*m*/*z*]	Calc. [*m*/*z*]	Error [ppm]	Ion Formula [M − H]$^-$
533.2968	533.2967	-0.1	$C_{26}H_{45}O_{11}^-$
275.1499	275.1500	0.4	$C_{13}H_{23}O_6^-$
127.0767	127.0765	-2.2	$C_7H_{11}O_6^-$

UPLC-HR(ESI$^+$)-qTOF-MS/MS			
Obs. [*m*/*z*]	Calc. [*m*/*z*]	Error [ppm]	Ion Formula [M + Na]$^+$
557.2954	557.2932	-3.9	$C_{26}H_{46}NaO_{11}^+$
427.2320	427.2302	-4.2	$C_{20}H_{36}NaO_8^+$
411.2007	411.1989	-4.3	$C_{19}H_{32}NaO_8^+$
299.1467	299.1465	-0.6	$C_{13}H_{24}NaO_6^+$
281.1361	281.1359	-0.4	$C_{13}H_{22}NaO_5^+$

Figure 11. UPLC-HR(ESI$^{-/+}$)-qTOF-MS/MS mass spectral data of known dasc#1 [4'-(asc-C7)-asc-C7] (***Bose et al., 2012***) in both negative and positive ion modes.

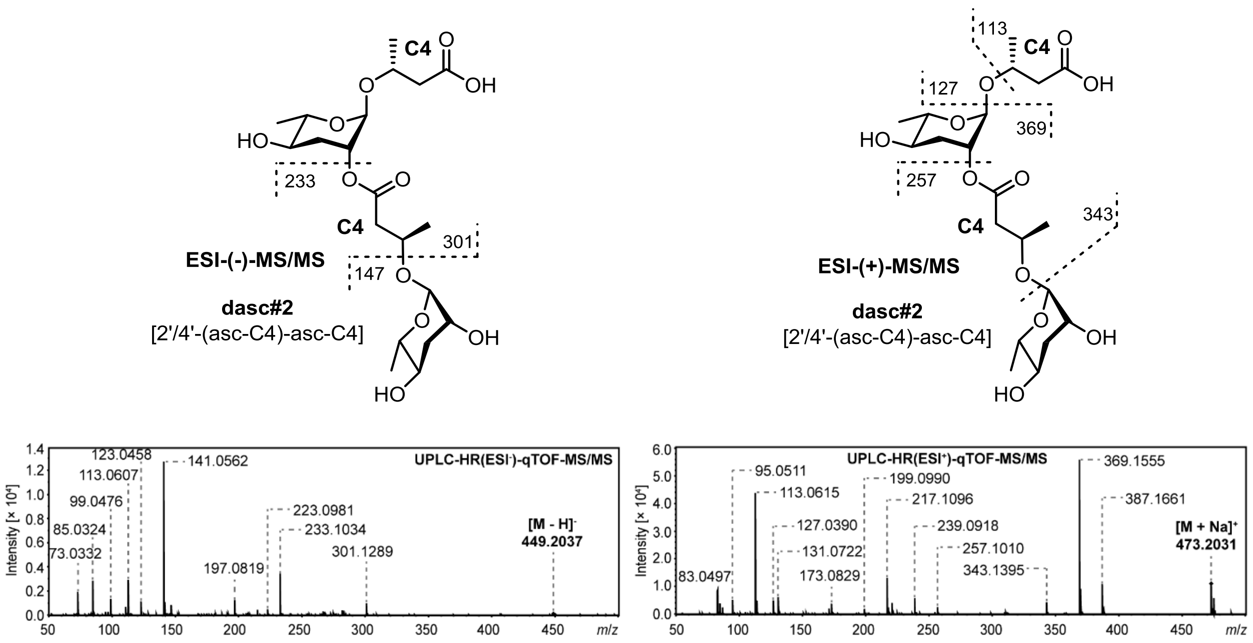

UPLC-HR(ESI⁻)-qTOF-MS/MS			
Obs. [*m*/*z*]	Calc. [*m*/*z*]	Error [ppm]	Ion Formula $[M-H]^-$
449.2037	449.2028	-1.8	$C_{20}H_{33}O_{11}^-$
301.1289	301.1293	1.3	$C_{14}H_{21}O_7^-$
233.1034	233.1031	-1.6	$C_{10}H_{17}O_6^-$
223.0981	223.0976	-2.2	$C_{12}H_{15}O_4^-$
197.0819	197.0819	0.3	$C_{10}H_{13}O_4^-$
141.0562	141.0557	-3.2	$C_7H_9O_3^-$
123.0458	123.0452	-5.2	$C_7H_7O_2^-$
113.0607	113.0608	2.6	$C_6H_9O_2^-$
99.0476	99.0452	-	$C_5H_7O_2^-$
85.0324	85.0295	-	$C_4H_5O_2^-$
73.0332	73.0295	-	$C_3H_5O_2^-$

UPLC-HR(ESI⁺)-qTOF-MS/MS			
Obs. [*m*/*z*]	Calc. [*m*/*z*]	Error [ppm]	Ion Formula $[M+Na]^+$
473.2031	473.1993	-8.1	$C_{20}H_{34}NaO_{11}^+$
387.1661	387.1626	-9.2	$C_{16}H_{28}NaO_9^+$
369.1555	369.1520	-9.4	$C_{16}H_{26}NaO_8^+$
343.1395	343.1363	-9.2	$C_{14}H_{24}NaO_8^+$
257.1010	257.0996	-5.7	$C_{10}H_{18}NaO_6^+$
239.0918	239.0890	-	$C_{10}H_{16}NaO_5^+$
217.1096	217.1046	-	$C_8H_{18}NaO_5^+$
199.0990	199.0841	-	$C_8H_{16}NaO_4^+$
131.0722	131.0703	-	$C_6H_{11}O_3^+$
127.0390	127.0366	-	$C_4H_8NaO_3^+$
113.0615	113.0573	-	$C_4H_{10}NaO_2^+$
95.0511	95.0467	-	$C_4H_8NaO^+$
83.0497	83.0467	-	$C_3H_8NaO^+$

Figure 12. UPLC-HR(ESI$^{-/+}$)-qTOF-MS/MS mass spectral data of dasc#2 [2'/4'-(asc-C4)-asc-C4] in both negative and positive ion modes. Note that dasc#2 [2'/4'-(asc-C4)-asc-C4] is proposed and illustrated to be modified at the 2'-position, but its absolute structure has not yet been elucidated.

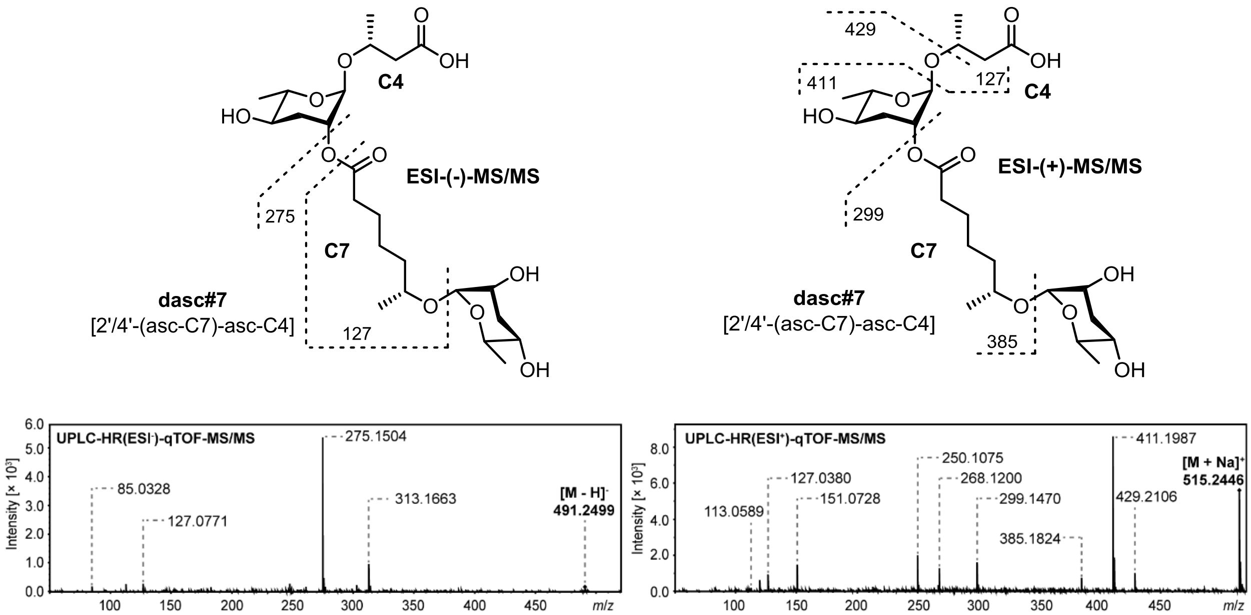

UPLC-HR(ESI⁻)-qTOF-MS/MS			
Obs. [*m/z*]	Calc. [*m/z*]	Error [ppm]	Ion Formula [M − H]⁻
491.2499	491.2498	-0.2	$C_{23}H_{39}O_{11}^-$
313.1663	313.1657	-2.0	$C_{16}H_{25}O_6^-$
275.1504	275.1500	-1.5	$C_{13}H_{23}O_6^-$
127.0771	127.0765	-4.9	$C_7H_{11}O_2^-$
85.0328	85.0295	-	$C_4H_5O_2^-$

UPLC-HR(ESI⁺)-qTOF-MS/MS			
Obs. [*m/z*]	Calc. [*m/z*]	Error [ppm]	Ion Formula [M + Na]⁺
515.2446	515.2463	3.3	$C_{23}H_{40}NaO_{11}^+$
429.2106	429.2095	-2.6	$C_{19}H_{34}NaO_9^+$
411.1987	411.1989	0.6	$C_{19}H_{32}NaO_8^+$
385.1824	385.1833	2.3	$C_{17}H_{30}NaO_8^+$
299.1470	299.1465	-1.6	$C_{13}H_{24}NaO_6^+$
268.1200	268.1179	-7.5	$C_{13}H_{18}NO_5^+$
250.1075	250.1074	-0.6	$C_{13}H_{16}NO_4^+$
151.0728	151.0730	1.1	$C_7H_{12}NaO_2^+$
127.0380	127.0366	-	$C_4H_8NaO_3^+$
113.0589	113.0573	-	$C_4H_{10}NaO_2^+$

Figure 13. UPLC-HR(ESI$^{-/+}$)-qTOF-MS/MS mass spectral data of dasc#7 [2'/4'-(asc-C7)-asc-C4] in both negative and positive ion modes. Note that dasc#7 [2'/4'-(asc-C7)-asc-C4] is tentatively assigned to be linked at the 2'-position, but its absolute structure has not yet been elucidated.

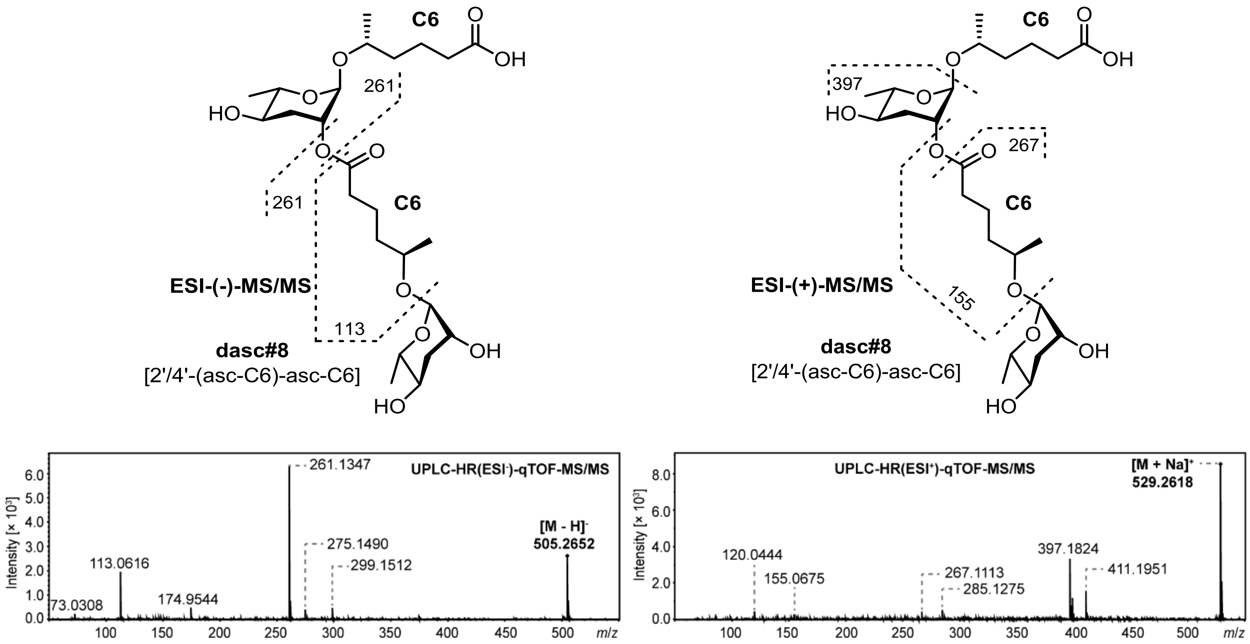

UPLC-HR(ESI⁻)-qTOF-MS/MS			
Obs. [*m/z*]	Calc. [*m/z*]	Error [ppm]	Ion Formula $[M-H]^-$
505.2652	505.2654	0.4	$C_{24}H_{41}O_{11}^-$
299.1512	299.1500	-4.1	$C_{15}H_{23}O_6^-$
275.1490	275.1500	3.6	$C_{13}H_{23}O_6^-$
261.1347	261.1344	-1.5	$C_{12}H_{21}O_6^-$
113.0616	113.0608	-	$C_6H_9O_2^-$
73.0308	73.0295	-	$C_3H_5O_2^-$

UPLC-HR(ESI⁺)-qTOF-MS/MS			
Obs. [*m/z*]	Calc. [*m/z*]	Error [ppm]	Ion Formula $[M+Na]^+$
529.2618	529.2619	0.2	$C_{24}H_{42}NaO_{11}^+$
411.1951	411.1989	-	$C_{19}H_{32}NaO_8^+$
397.1824	397.1833	2.2	$C_{18}H_{30}NaO_8^+$
285.1275	285.1309	-	$C_{12}H_{22}NaO_6^+$
267.1113	267.1203	-	$C_{12}H_{20}NaO_5^+$
155.0675	155.0679	2.3	$C_6H_{12}NaO_3^+$

Figure 14. UPLC-HR(ESI$^{-/+}$)-qTOF-MS/MS mass spectral data of dasc#8 [2'/4'-(asc-C6)-asc-C6] in both negative and positive ion modes. Note that dasc#8 [2'/4'-(asc-C6)-asc-C6] is tentatively assigned to be linked at the 2'-position, but its absolute structure has not yet been elucidated.

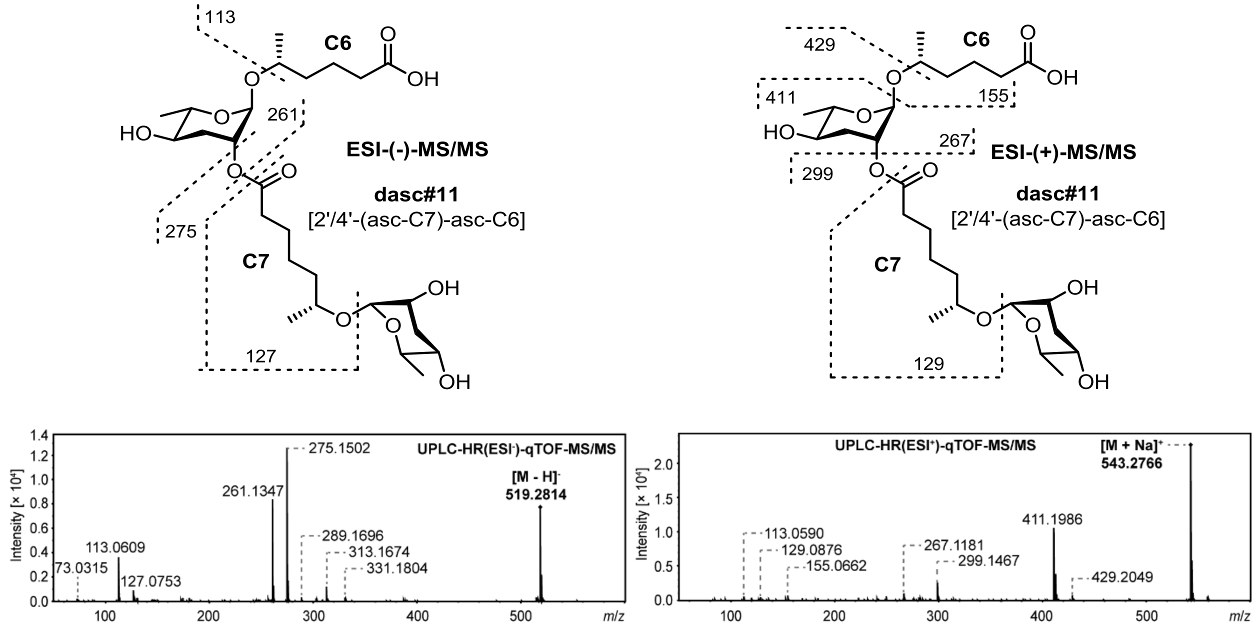

UPLC-HR(ESI$^-$)-qTOF-MS/MS			
Obs. [*m*/*z*]	Calc. [*m*/*z*]	Error [ppm]	Ion Formula [M − H]$^-$
519.2814	519.2811	-0.6	$C_{25}H_{43}O_{11}^-$
331.1804	331.1762	-	$C_{16}H_{27}O_7^-$
313.1674	313.1657	-5.4	$C_{16}H_{25}O_6^-$
289.1696	289.1657	-	$C_{14}H_{25}O_6^-$
275.1502	275.1500	-0.7	$C_{13}H_{23}O_6^-$
261.1347	261.1344	-1.4	$C_{12}H_{21}O_6^-$
127.0753	127.0765	8.8	$C_7H_{11}O_2^-$
113.0609	113.0608	-1.3	$C_6H_9O_2^-$
73.0315	73.0295	-	$C_3H_5O_2^-$

UPLC-HR(ESI$^+$)-qTOF-MS/MS			
Obs. [*m*/*z*]	Calc. [*m*/*z*]	Error [ppm]	Ion Formula [M + Na]$^+$
543.2766	543.2776	1.7	$C_{25}H_{44}NaO_{11}^+$
429.2049	429.2095	-	$C_{19}H_{34}NaO_9^+$
411.1986	411.1989	0.7	$C_{19}H_{32}NaO_8^+$
299.1467	299.1465	-0.6	$C_{13}H_{24}NaO_6^+$
281.1377	281.1359	-6.1	$C_{13}H_{22}NaO_5^+$
267.1181	267.1203	-	$C_{12}H_{20}NaO_5^+$
155.0662	155.0679	-	$C_6H_{12}NaO_3^+$
129.0876	129.0910	-	$C_7H_{13}O_2^+$
113.0590	113.0597	-	$C_6H_9O_2^+$

Figure 15. UPLC-HR(ESI$^{-/+}$)-qTOF-MS/MS mass spectral data of dasc#11 [2'/4'-(asc-C7)-asc-C6] in both negative and positive ion modes. Note that dasc#11 [2'/4'-(asc-C7)-asc-C6] is tentatively assigned to be linked at the 2'-position, but its absolute structure has not yet been elucidated.

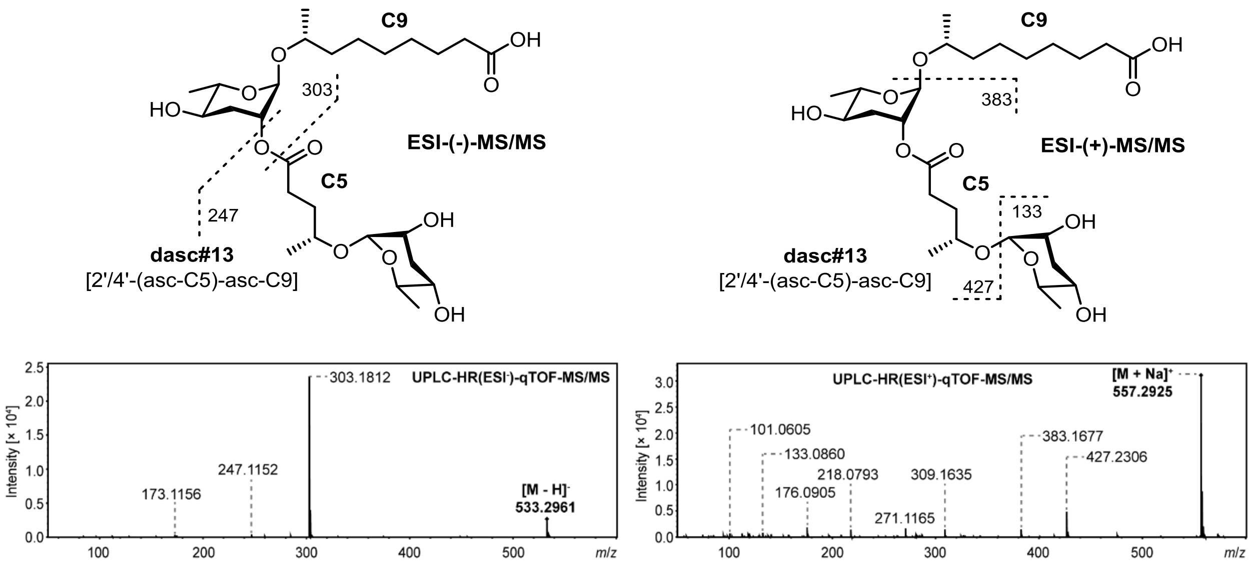

UPLC-HR(ESI⁻)-qTOF-MS/MS			
Obs. [*m*/*z*]	Calc. [*m*/*z*]	Error [ppm]	Ion Formula $[M-H]^-$
533.2961	533.2967	1.3	$C_{26}H_{45}O_{11}^-$
303.1812	303.1813	0.3	$C_{15}H_{27}O_6^-$
247.1152	247.1187	-	$C_{11}H_{19}O_6^-$
173.1156	173.1183	-	$C_9H_{17}O_3^-$

UPLC-HR(ESI⁺)-qTOF-MS/MS			
Obs. [*m*/*z*]	Calc. [*m*/*z*]	Error [ppm]	Ion Formula $[M+Na]^+$
557.2925	557.2932	1.4	$C_{26}H_{46}N_aO_{11}^+$
427.2306	427.2302	-0.9	$C_{20}H_{36}N_aO_8^+$
383.1677	383.1676	-0.2	$C_{17}H_{28}N_aO_8^+$
309.1635	309.1672	-	$C_{15}H_{26}N_aO_5^+$
271.1165	271.1152	-4.7	$C_{11}H_{20}N_aO_6^+$
133.0860	133.0859	-0.4	$C_6H_{13}O_3^+$
113.0597	113.0597	-0.2	$C_6H_9O_2^+$

Figure 16. UPLC-HR(ESI$^{-/+}$)-qTOF-MS/MS mass spectral data of dasc#13 [2'/4'-(asc-C5)-asc-C9] in both negative and positive ion modes. Note that dasc#13 [2'/4'-(asc-C5)-asc-C9] is tentatively assigned to be linked at the 2'-position, but its absolute structure has not yet been elucidated.

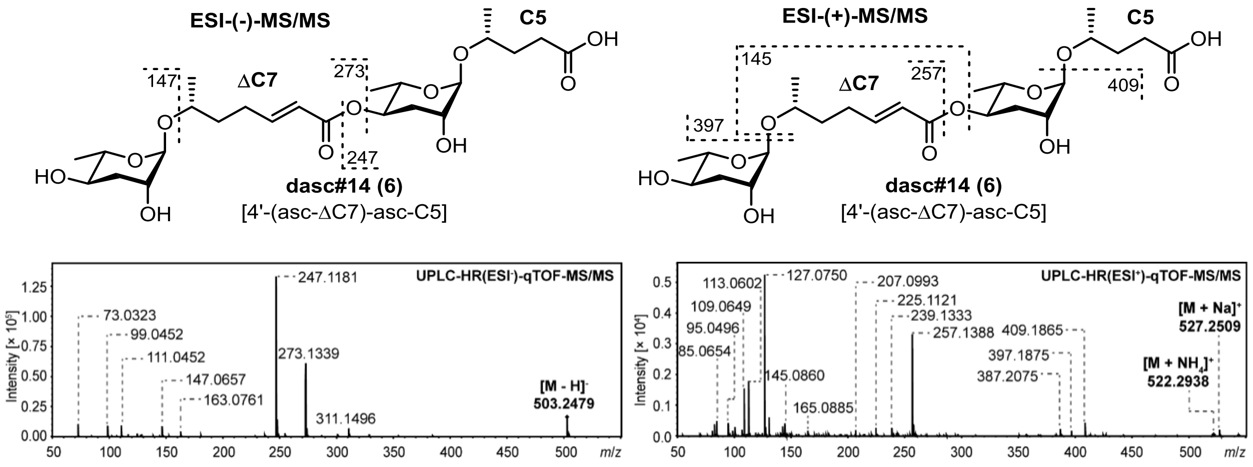

UPLC-HR(ESI⁻)-qTOF-MS/MS			
Obs. [*m*/*z*]	Calc. [*m*/*z*]	Error [ppm]	Ion Formula $[M-H]^-$
503.2479	503.2498	3.8	$C_{24}H_{39}O_{11}^-$
311.1496	311.1500	1.3	$C_{16}H_{23}O_6^-$
273.1339	273.1344	1.7	$C_{13}H_{21}O_6^-$
247.1181	247.1187	2.4	$C_{11}H_{19}O_6^-$
163.0761	163.0765	2.4	$C_{10}H_{11}O_2^-$
147.0657	147.0663	4.2	$C_6H_{11}O_4^-$
111.0452	111.0452	0.0	$C_6H_7O_2^-$
99.0452	99.0452	-0.1	$C_5H_7O_2^-$
73.0323	73.0295	-	$C_3H_5O_2^-$

UPLC-HR(ESI⁺)-qTOF-MS/MS			
Obs. [*m*/*z*]	Calc. [*m*/*z*]	Error [ppm]	Ion Formula $[M+NH_4]^+$
522.2938	522.2909	-5.5	$C_{24}H_{44}NO_{11}^+$
409.1865	409.1833	-7.9	$C_{19}H_{30}NaO_8^+$
397.1875	397.1833	-	$C_{18}H_{30}NaO_8^+$
387.2075	387.2126	-	$C_{19}H_{31}O_8^+$
257.1388	257.1384	-1.6	$C_{13}H_{21}O_5^+$
239.1333	239.1278	-	$C_{13}H_{19}O_4^+$
225.1121	225.1121	0.1	$C_{10}H_{18}NaO_4^+$
207.0993	207.0992	-0.7	$C_{10}H_{16}NaO_3^+$
165.0885	165.0886	0.4	$C_8H_{14}NaO_2^+$
145.0860	145.0859	-0.8	$C_7H_{13}O_3^+$
127.0750	127.0754	2.9	$C_7H_{11}O_2^+$
113.0602	113.0597	-4.5	$C_6H_9O_2^+$
109.0649	109.0648	-1.1	$C_7H_9O^+$
95.0496	95.0491	-	$C_6H_7O^+$
85.0654	85.0648	-	$C_5H_9O^+$

Figure 17. UPLC-HR(ESI$^{-/+}$)-qTOF-MS/MS mass spectral data of dasc#14 [4'-(asc-ΔC7)-asc-C5, **6**] in both negative and positive ion modes.

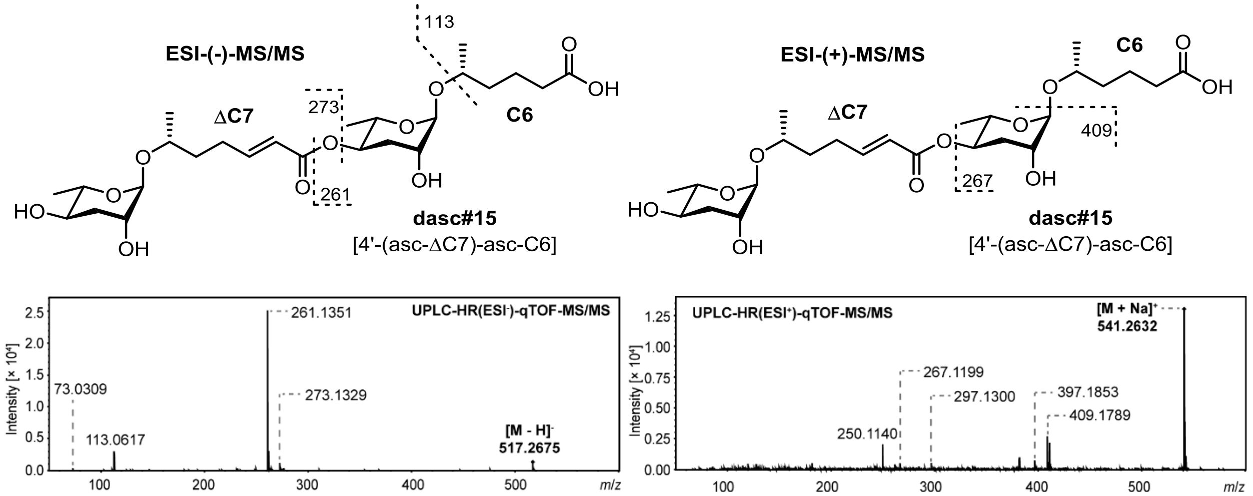

UPLC-HR(ESI^{-})-qTOF-MS/MS			
Obs. [*m*/*z*]	Calc. [*m*/*z*]	Error [ppm]	Ion Formula [M − H]$^{-}$
517.2675	517.2654	-4.1	$C_{25}H_{41}O_{11}^{-}$
273.1329	273.1344	5.4	$C_{13}H_{21}O_{6}^{-}$
261.1351	261.1344	-2.9	$C_{12}H_{21}O_{6}^{-}$
113.0617	113.0608	-	$C_{6}H_{9}O_{2}^{-}$
73.0309	73.0295	-	$C_{3}H_{5}O_{2}^{-}$

UPLC-HR(ESI^{+})-qTOF-MS/MS			
Obs. [*m*/*z*]	Calc. [*m*/*z*]	Error [ppm]	Ion Formula [M + Na]$^{+}$
541.2632	541.2619	-2.3	$C_{25}H_{42}NaO_{11}^{+}$
409.1789	409.1833	-	$C_{19}H_{30}NaO_{8}^{+}$
397.1853	397.1833	-5.2	$C_{18}H_{30}NaO_{8}^{+}$
297.1300	297.1309	2.9	$C_{13}H_{22}NaO_{6}^{+}$
267.1199	267.1203	1.4	$C_{12}H_{20}NaO_{5}^{+}$

Figure 18. UPLC-HR(ESI$^{-/+}$)-qTOF-MS/MS mass spectral data of dasc#15 [4'-(asc-ΔC7)-asc-C6] in both negative and positive ion modes. Note that dasc#15 [4'-(asc-ΔC7)-asc-C6] is proposed to be linked at the 4'-position, but its absolute structure has not yet been elucidated.

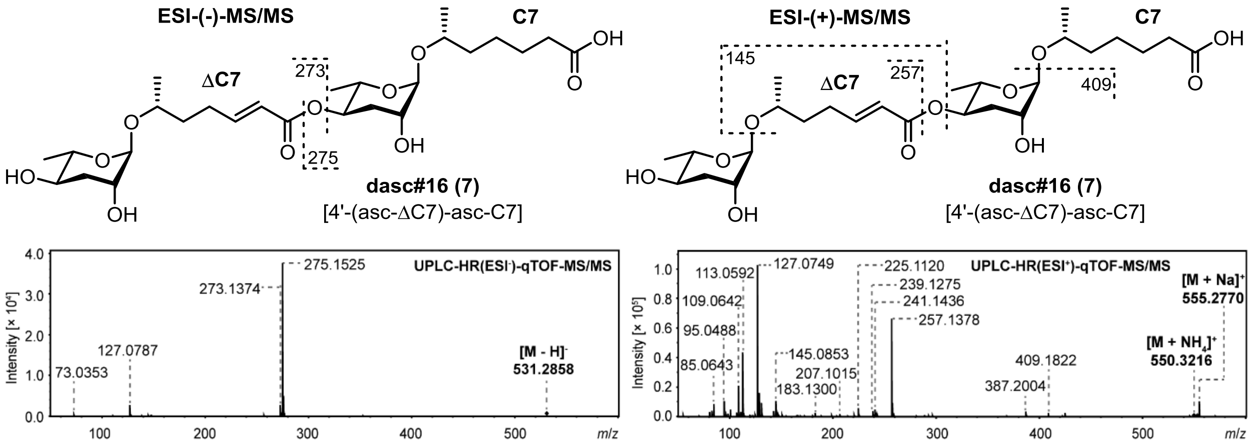

UPLC-HR(ESI$^-$)-qTOF-MS/MS			
Obs. [*m*/*z*]	Calc. [*m*/*z*]	Error [ppm]	Ion Formula [M − H]$^-$
531.2858	531.2811	-8.8	$C_{26}H_{43}O_{11}^-$
275.1525	275.1500	-9.0	$C_{13}H_{23}O_6^-$
273.1374	273.1344	-	$C_{13}H_{21}O_6^-$
127.0787	127.0765	-	$C_7H_{11}O_2^-$
73.0353	73.0295	-	$C_3H_5O_2^-$

UPLC-HR(ESI$^+$)-qTOF-MS/MS			
Obs. [*m*/*z*]	Calc. [*m*/*z*]	Error [ppm]	Ion Formula [M + NH$_4$]$^+$
550.3216	550.3222	1.0	$C_{26}H_{48}NO_{11}^+$
555.2770	555.2776	1.0	$C_{26}H_{44}NaO_{11}^+$
409.1822	409.1833	2.6	$C_{19}H_{30}NaO_8^+$
387.2004	387.2013	2.4	$C_{19}H_{31}O_8^+$
257.1378	257.1384	2.1	$C_{13}H_{21}O_5^+$
241.1436	241.1434	-0.7	$C_{13}H_{21}O_4^+$
239.1275	239.1278	1.0	$C_{13}H_{19}O_4^+$
225.1120	225.1121	0.7	$C_{12}H_{17}O_4^+$
207.1015	207.0992	0.5	$C_{12}H_{15}O_3^+$
145.0853	145.0859	4.2	$C_7H_{13}O_3^+$
127.0749	127.0754	4.0	$C_7H_{11}O_2^+$
113.0592	113.0597	4.5	$C_6H_9O_2^+$
109.0642	109.0648	5.9	$C_7H_9O^+$
95.0488	95.0491	3.3	$C_6H_7O^+$
85.0643	85.0648	5.3	$C_5H_9O^+$

Figure 19. UPLC-HR(ESI$^{-/+}$)-qTOF-MS/MS mass spectral data of dasc#16 [4'-(asc-ΔC7)-asc-C7, **7**] in both negative and positive ion modes.

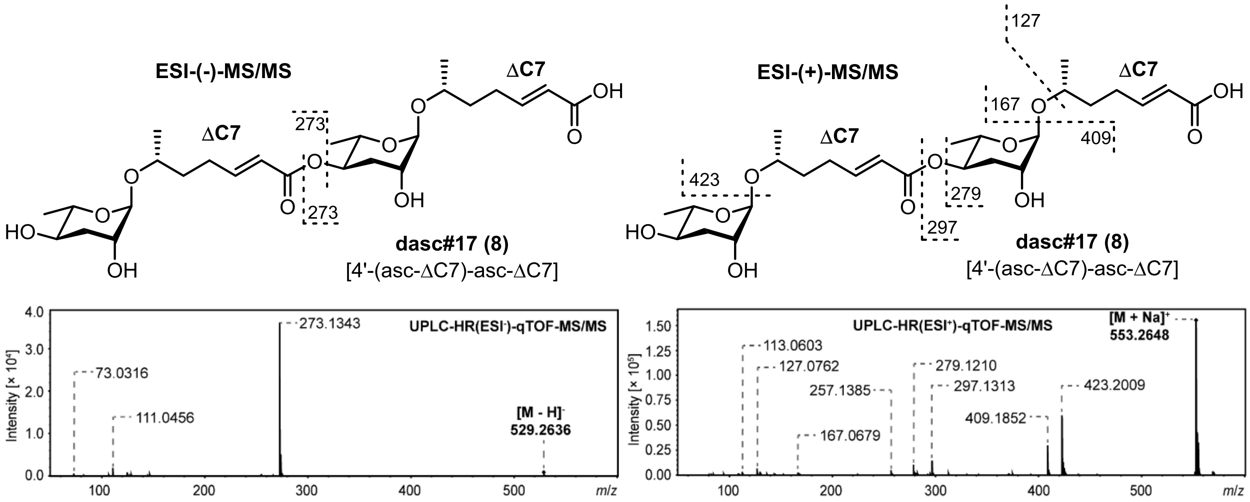

UPLC-HR(ESI$^-$)-qTOF-MS/MS			
Obs. [m/z]	Calc. [m/z]	Error [ppm]	Ion Formula [M − H]$^-$
529.2636	529.2654	3.5	$C_{26}H_{41}O_{11}^-$
273.1343	273.1344	0.3	$C_{13}H_{21}O_6^-$
111.0456	111.0452	-4.2	$C_6H_7O_2^-$
73.0316	73.0295	-	$C_3H_5O_2^-$

UPLC-HR(ESI$^+$)-qTOF-MS/MS			
Obs. [m/z]	Calc. [m/z]	Error [ppm]	Ion Formula [M + Na]$^+$
553.2648	552.2619	-5.2	$C_{26}H_{42}NaO_{11}^+$
423.2009	423.2013	-4.6	$C_{20}H_{32}NaO_8^+$
409.1852	409.1833	-4.7	$C_{19}H_{30}NaO_8^+$
297.1313	297.1309	-1.5	$C_{13}H_{22}NaO_6^+$
279.1210	279.1203	-2.5	$C_{13}H_{20}NaO_5^+$
257.1385	257.1384	-0.6	$C_{13}H_{21}O_5^+$
167.0679	167.0679	-0.2	$C_7H_{13}NaO_3^+$
127.0762	127.0754	-6.4	$C_7H_{11}O_2^+$
113.0603	113.0597	-5.4	$C_6H_9O_2^+$

Figure 20. UPLC-HR(ESI$^{-/+}$)-qTOF-MS/MS mass spectral data of dasc#17 [4'-(asc-ΔC7)-asc-ΔC7, **8**] in both negative and positive ion modes.

supplementary file 2b: MS/MS spectral data of paso#9

	Pages
**Figure 1.** LC-HR($ESI^{-/+}$)-MS/MS spectral data of paso#9.	S24

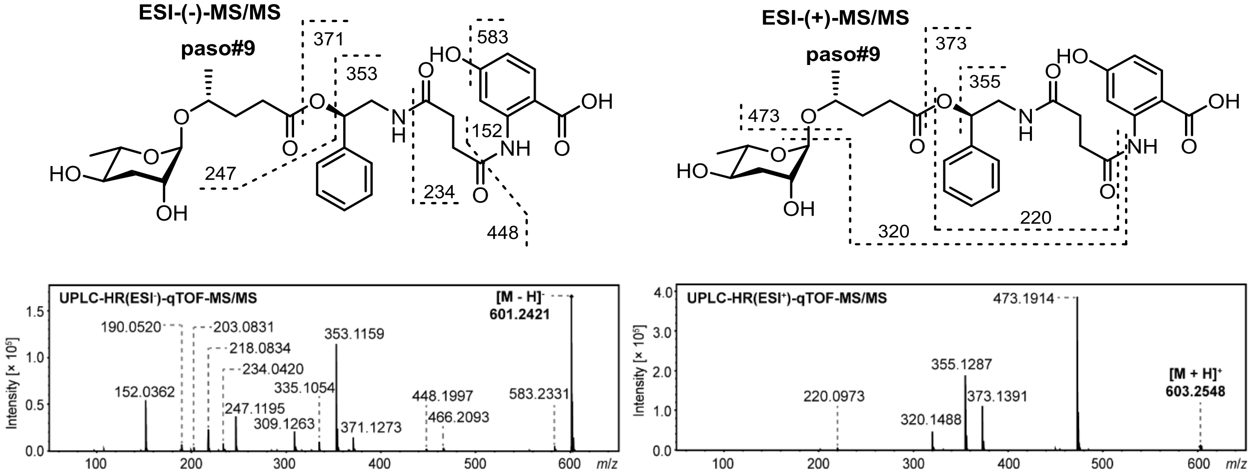

UPLC-HR(ESI⁻)-qTOF-MS/MS			
Obs. [*m*/*z*]	Calc. [*m*/*z*]	Error [ppm]	Ion Formula [M − H]⁻
601.2421	601.2403	-1.9	$C_{30}H_{37}N_2O_{11}^-$
583.2331	583.2297	-3.3	$C_{30}H_{35}N_2O_{10}^-$
466.2093	466.2083	-1.0	$C_{23}H_{32}NO_9^-$
448.1997	448.1977	-2.0	$C_{23}H_{30}NO_8^-$
371.1273	371.1249	-2.5	$C_{19}H_{19}N_2O_6^-$
353.1159	353.1143	-1.6	$C_{19}H_{17}N_2O_5^-$
335.1054	335.1037	-1.7	$C_{19}H_{15}N_2O_4^-$
309.1263	309.1245	-1.8	$C_{18}H_{17}N_2O_3^-$
247.1195	247.1187	-0.8	$C_{11}H_{19}O_6^-$
234.0420	234.0408	-1.2	$C_{11}H_8NO_5^-$
218.0834	218.0823	-1.2	$C_{12}H_{12}NO_3^-$
203.0831	203.0826	-0.5	$C_{11}H_{11}N_2O_2^-$
190.0520	190.0510	-1.1	$C_{10}H_8NO_3^-$
152.0362	152.0353	-0.9	$C_7H_6NO_3^-$

UPLC-HR(ESI⁺)-qTOF-MS/MS			
Obs. [*m*/*z*]	Calc. [*m*/*z*]	Error [ppm]	Ion Formula [M + H]⁺
603.2548	603.2548	0.1	$C_{30}H_{39}N_2O_{11}^+$
473.1914	473.1918	0.4	$C_{24}H_{29}N_2O_8^+$
373.1391	373.1394	0.4	$C_{19}H_{21}N_2O_6^+$
355.1287	355.1288	0.2	$C_{19}H_{19}N_2O_5^+$
320.1488	320.1492	0.5	$C_{17}H_{22}NO_5^+$
220.0973	220.0968	-0.5	$C_{12}H_{14}NO_3^+$

Figure 1. UPLC-HR(ESI$^{-/+}$)-qTOF-MS/MS mass spectral data of paso#9 in both negative and positive ion modes. Analysis of NMR data of paso#9 suggests a 4-hydroxyanthranilic acid moiety in paso#9 (***supplementary file 1c – Figures 5-13*** and ***supplementary file 3 – Table 14***).

supplementary file 2c: MS/MS spectral data of UBAS chemicals

Pages

Figure 1. LC-HR(ESI$^{-/+}$)-MS/MS spectral data of ubas#5 [4'-UB-asc-C4] from *P. pacificus*. S26
Figure 2. LC-HR(ESI$^{-/+}$)-MS/MS spectral data of ubas#3 [4'-UB-asc-C5] from *P. pacificus*. S27
Figure 3. LC-HR(ESI$^{-/+}$)-MS/MS spectral data of ubas#12 [4'-UB-asc-C6] from *P. pacificus*. S28
Figure 4. LC-HR(ESI$^{-/+}$)-MS/MS spectral data of ubas#4 [4'-UB-asc-C7] from *P. pacificus*. S29
Figure 5. LC-HR(ESI$^{-/+}$)-MS/MS spectral data of ubas#1 from *P. pacificus*. S30
Figure 6. LC-HR(ESI$^{-/+}$)-MS/MS spectral data of ubas#2 from *P. pacificus*. S31
Figure 7. LC-HR(ESI$^{-/+}$)-MS/MS spectral data of ubas#26 [4'-UB-asc-ωC3]. S32
Figure 8. LC-HR(ESI$^{-/+}$)-MS/MS spectral data of ubas#33 [4'-UB-2'-(asc-ωC3)-asc-C4]. S33
Figure 9. LC-HR(ESI$^{-/+}$)-MS/MS spectral data of ubas#34 [4'-UB-2'-(asc-C4)-asc-C4, **11**]. S34
Figure 10. LC-HR(ESI$^{-/+}$)-MS/MS spectral data of ubas#35 [4'-UB-2'-(asc-C4)-asc-C5, **12**]. S35
Figure 11. LC-HR(ESI$^{-/+}$)-MS/MS spectral data of ubas#36 [4'-UB-2'-(asc-C4)-asc-C6]. S36
Figure 12. LC-HR(ESI$^{-/+}$)-MS/MS spectral data of ubas#37 [4'-UB-2'-(asc-C4)-asc-C7]. S37
Figure 13. LC-HR(ESI$^{-/+}$)-MS/MS spectral data of ubas#27 [4'-UB-2'-(asc-C5)-asc-ωC3]. S38
Figure 14. LC-HR(ESI$^{-/+}$)-MS/MS spectral data of ubas#28 [4'-UB-2'-(asc-C5)-asc-C4, **13**]. S39
Figure 15. LC-HR(ESI$^{-/+}$)-MS/MS spectral data of ubas#1 from *P. taiwanensis*. S40
Figure 16. LC-HR(ESI$^{-/+}$)-MS/MS spectral data of ubas#1 from *P. laevicollis*. S41
Figure 17. LC-HR(ESI$^{-/+}$)-MS/MS spectral data of ubas#30 (**9**) from *P. maxplancki*. S42
Figure 18. LC-HR(ESI$^{-/+}$)-MS/MS spectral data of ubas#30 (**9**) from *P. fukushimae*. S43
Figure 19. LC-HR(ESI$^{-/+}$)-MS/MS spectral data of ubas#29 [4'-UB-2'-(asc-C6)-asc-C4]. S44
Figure 20. LC-HR(ESI$^{-/+}$)-MS/MS spectral data of ubas#31 [4'-UB-2'-(asc-C6)-asc-C6]. S45
Figure 21. LC-HR(ESI$^{-/+}$)-MS/MS spectral data of ubas#32 [4'-UB-2'-(asc-C5)-asc-C7, **10**]. S46

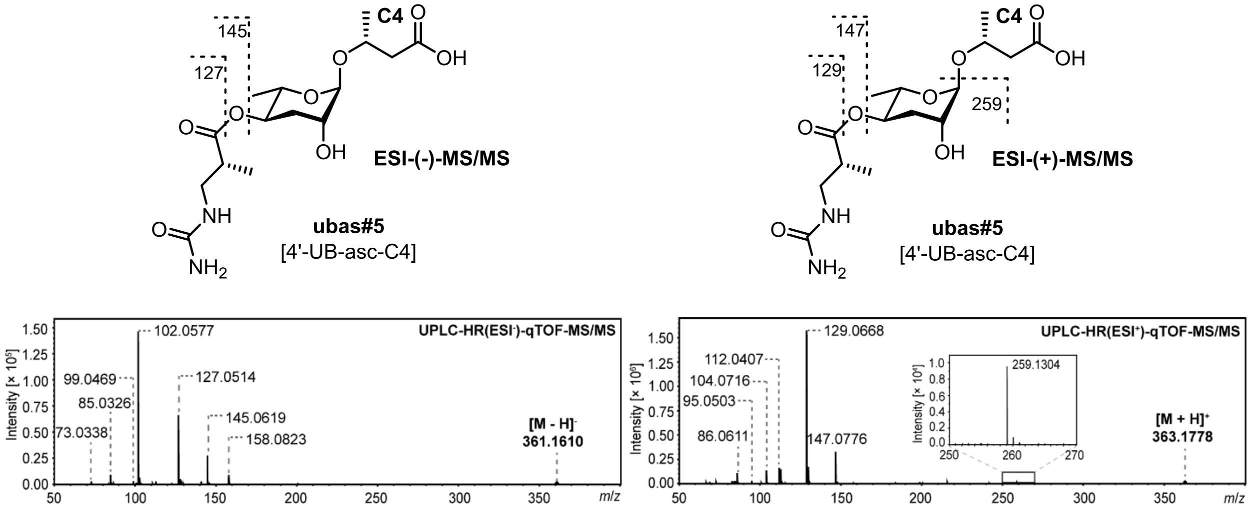

UPLC-HR(ESI⁻)-qTOF-MS/MS			
Obs. [*m*/*z*]	Calc. [*m*/*z*]	Error [ppm]	Ion Formula $[M - H]^-$
361.1610	361.1616	1.9	$C_{15}H_{25}N_2O_8^-$
158.0823	158.0823	-0.2	$C_7H_{12}NO_3^-$
145.0619	145.0619	-0.2	$C_5H_9N_2O_3^-$
127.0514	127.0513	-0.5	$C_5H_7N_2O_2^-$
102.0577	102.0561	-	$C_4H_8NO_2^-$
99.0469	99.0452	-	$C_5H_7O_2^-$
85.0326	85.0295	-	$C_4H_5O_2^-$
73.0338	73.0295	-	$C_3H_5O_2^-$

UPLC-HR(ESI⁺)-qTOF-MS/MS			
Obs. [*m*/*z*]	Calc. [*m*/*z*]	Error [ppm]	Ion Formula $[M + H]^+$
363.1778	363.1762	-4.4	$C_{15}H_{27}N_2O_8^+$
259.1304	259.1288	-6.1	$C_{11}H_{19}N_2O_5^+$
147.0776	147.0764	-8.3	$C_5H_{11}N_2O_3^+$
129.0668	129.0659	-7.5	$C_5H_9N_2O_2^+$
112.0407	112.0393	-	$C_5H_6NO_2^+$
104.0716	104.0706	-	$C_4H_{10}NO_2^+$
95.0503	95.0491	-	$C_6H_7O^+$
86.0611	86.0600	-	$C_4H_8NO^+$

Figure 1. UPLC-HR(ESI$^{-/+}$)-qTOF-MS/MS mass spectral data of known ubas#5 [4'-UB-asc-C4] (***Falcke et al., 2018***) from *P. pacificus* in both negative and positive ion modes. Characteristic ion signal for *m*/*z* 259.1304 containing an ureidoisobutyric acid moiety and an ascarylose unit was used for targeted MS/MS screening. *P. triformis* also produced large amounts of ubas#5 [4'-UB-asc-C4] (***supplementary file 1d – Figures 30-35***).

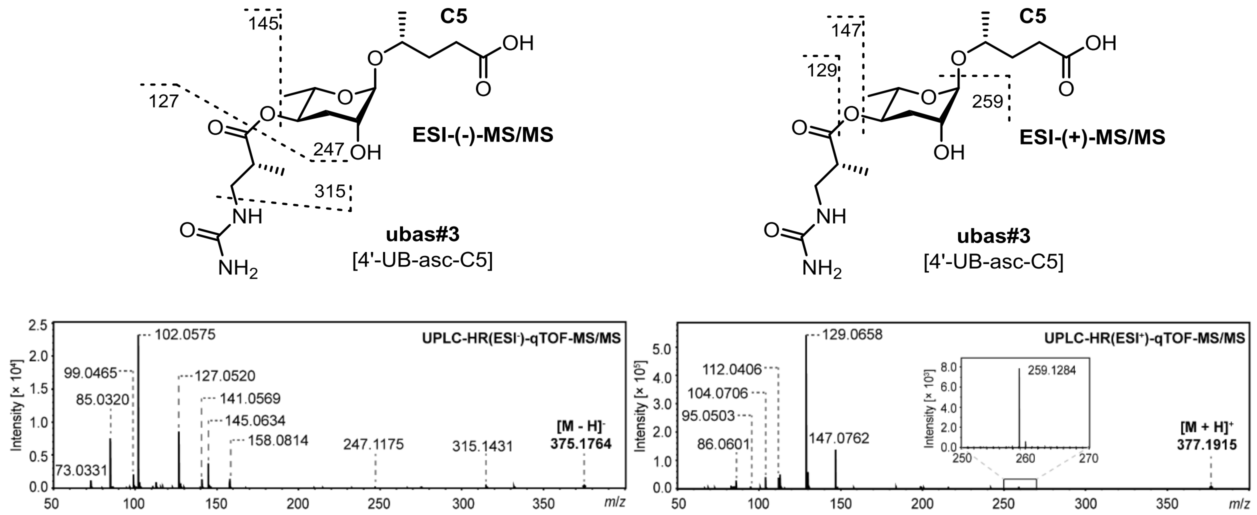

UPLC-HR(ESI$^-$)-qTOF-MS/MS			
Obs. [*m/z*]	Calc. [*m/z*]	Error [ppm]	Ion Formula [M − H]$^-$
375.1764	375.1773	2.3	$C_{16}H_{27}N_2O_8^-$
315.1431	315.1449	5.8	$C_{15}H_{23}O_7^-$
247.1175	247.1187	4.8	$C_{11}H_{19}O_6^-$
158.0814	158.0823	5.3	$C_7H_{12}NO_3^-$
145.0634	145.0619	-	$C_5H_9N_2O_3^-$
141.0569	141.0557	-	$C_7H_9O_3^-$
127.0520	127.0513	-5.8	$C_5H_7N_2O_2^-$
102.0575	102.0561	-	$C_4H_8NO_2^-$
99.0465	99.0452	-	$C_5H_7O_2^-$
85.0320	85.0295	-	$C_4H_5O_2^-$
73.0331	73.0295	-	$C_3H_5O_2^-$

UPLC-HR(ESI$^+$)-qTOF-MS/MS			
Obs. [*m/z*]	Calc. [*m/z*]	Error [ppm]	Ion Formula [M + H]$^+$
377.1915	377.1918	0.8	$C_{16}H_{29}N_2O_8^+$
259.1284	259.1288	1.6	$C_{11}H_{19}N_2O_5^+$
147.0762	147.0764	1.5	$C_5H_{11}N_2O_3^+$
129.0658	129.0659	0.6	$C_5H_9N_2O_2^+$
112.0406	112.0393	-	$C_5H_6NO_2^+$
104.0706	104.0706	-0.4	$C_4H_{10}NO_2^+$
95.0503	95.0491	-	$C_6H_7O^+$
86.0601	86.0600	-0.3	$C_4H_8NO^+$

Figure 2. UPLC-HR(ESI$^{-/+}$)-qTOF-MS/MS mass spectral data of known ubas#3 [4'-UB-asc-C5] (***Falcke et al., 2018***) from *P. pacificus* in both negative and positive ion modes. Characteristic ion signal for *m/z* 259.1304 containing an ureidoisobutyric acid moiety and an ascarylose unit was used for targeted MS/MS screening.

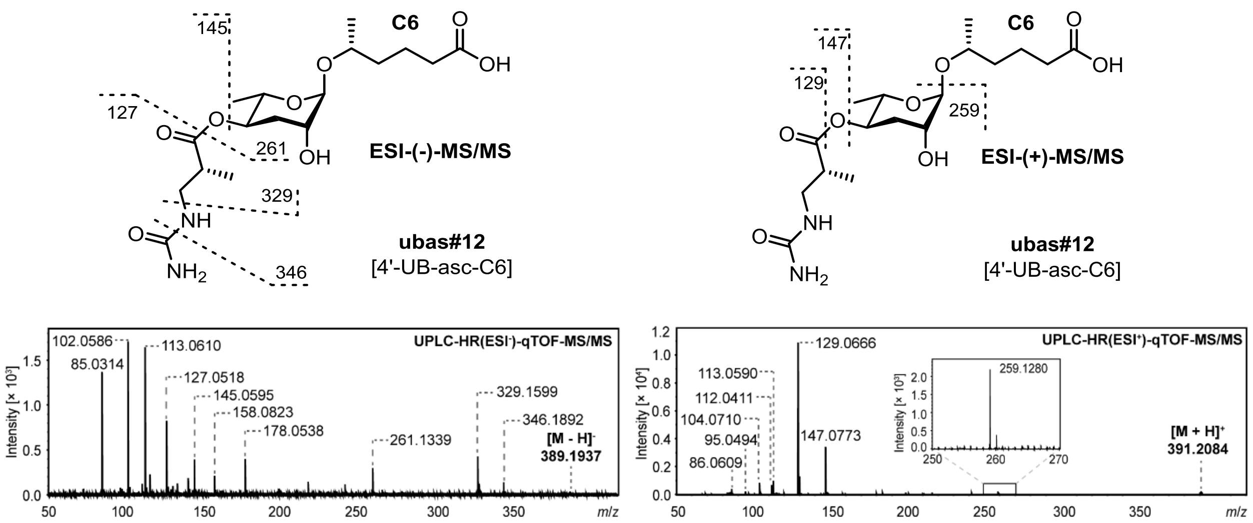

UPLC-HR(ESI⁻)-qTOF-MS/MS			
Obs. [*m*/*z*]	Calc. [*m*/*z*]	Error [ppm]	Ion Formula $[M - H]^-$
389.1937	389.1929	-2.0	$C_{17}H_{29}N_2O_8^-$
346.1892	346.1871	-6.0	$C_{16}H_{28}NO_7^-$
329.1599	329.1606	2.0	$C_{16}H_{25}O_7^-$
261.1339	261.1344	1.6	$C_{12}H_{21}O_6^-$
178.0538	178.0510	-	$C_9H_8NO_3^-$
158.0823	158.0823	0.0	$C_7H_{12}NO_3^-$
145.0595	145.0619	-	$C_5H_9N_2O_3^-$
127.0518	127.0513	-4.3	$C_5H_7N_2O_2^-$
113.0610	113.0608	-1.3	$C_6H_9O_2^-$
102.0586	102.0561	-	$C_4H_8NO_2^-$
85.0314	85.0295	-	$C_4H_5O_2^-$

UPLC-HR(ESI⁺)-qTOF-MS/MS			
Obs. [*m*/*z*]	Calc. [*m*/*z*]	Error [ppm]	Ion Formula $[M + H]^+$
391.2084	391.2075	-2.3	$C_{17}H_{31}N_2O_8^+$
259.1280	259.1288	3.1	$C_{11}H_{19}N_2O_5^+$
147.0773	147.0764	-6.2	$C_5H_{11}N_2O_3^+$
129.0666	129.0659	-5.8	$C_5H_9N_2O_2^+$
113.0590	113.0597	6.0	$C_6H_9O_2^+$
112.0411	112.0393	-	$C_5H_6NO_2^+$
104.0710	104.0706	-3.5	$C_4H_{10}NO_2^+$
95.0494	95.0491	-2.3	$C_6H_7O^+$
86.0609	86.0600	-	$C_4H_8NO^+$

Figure 3. UPLC-HR(ESI$^{-/+}$)-qTOF-MS/MS mass spectral data of known ubas#12 [4'-UB-asc-C6] (***Falcke et al., 2018***) from *P. pacificus* in both negative and positive ion modes. Characteristic ion signal for *m*/*z* 259.1304 containing an ureidoisobutyric acid moiety and an ascarylose unit was used for targeted MS/MS screening.

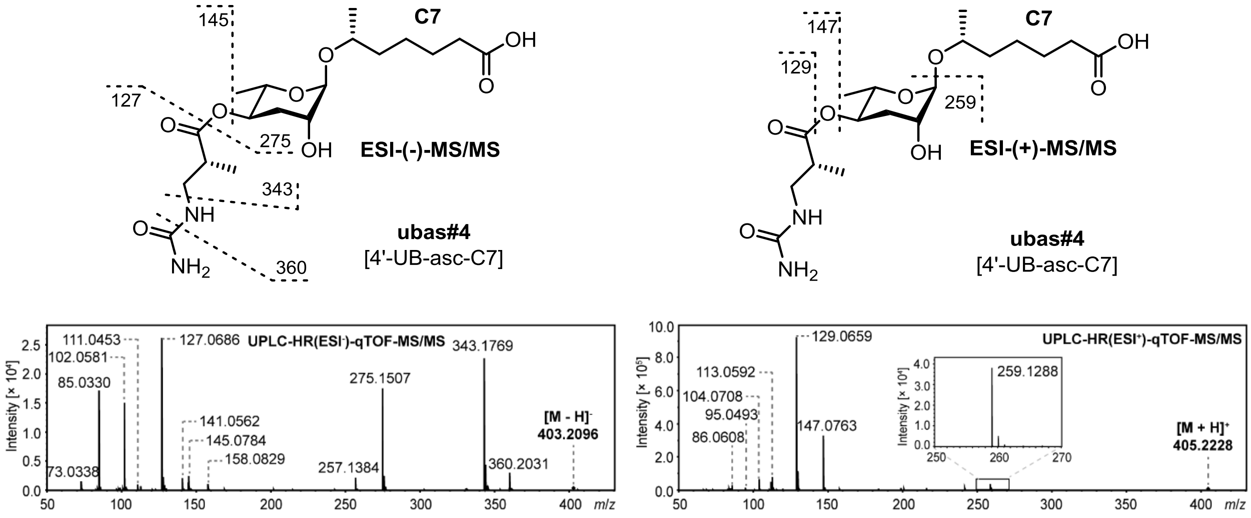

UPLC-HR(ESI⁻)-qTOF-MS/MS			
Obs. [*m/z*]	Calc. [*m/z*]	Error [ppm]	Ion Formula $[M-H]^-$
403.2096	403.2086	0.0	$C_{18}H_{31}N_2O_8^-$
360.2031	360.2028	-0.8	$C_{17}H_{30}NO_7^-$
343.1769	343.1762	-2.0	$C_{17}H_{27}O_7^-$
275.1507	275.1500	-2.6	$C_{13}H_{23}O_6^-$
257.1384	257.1394	3.9	$C_{13}H_{21}O_5^-$
158.0829	158.0823	-3.7	$C_7H_{12}NO_3^-$
145.0784	145.0619	-	$C_5H_9N_2O_3^-$
141.0562	141.0557	-3.4	$C_7H_9O_3^-$
127.0686	127.0513	-	$C_5H_7N_2O_2^-$
111.0453	111.0452	-1.5	$C_6H_7O_2^-$
102.0581	102.0561	-	$C_4H_8NO_2^-$
85.0330	85.0295	-	$C_4H_5O_2^-$
73.0338	73.0295	-	$C_3H_5O_2^-$

UPLC-HR(ESI⁺)-qTOF-MS/MS			
Obs. [*m/z*]	Calc. [*m/z*]	Error [ppm]	Ion Formula $[M+H]^+$
405.2228	405.2231	0.8	$C_{18}H_{33}N_2O_8^+$
259.1288	259.1288	0.3	$C_{11}H_{19}N_2O_5^+$
147.0763	147.0764	0.7	$C_5H_{11}N_2O_3^+$
129.0659	129.0659	0.0	$C_5H_9N_2O_2^+$
113.0592	113.0597	4.1	$C_6H_9O_2^+$
104.0708	104.0706	-1.8	$C_4H_{10}NO_2^+$
95.0493	95.0491	-1.2	$C_6H_7O^+$
86.0608	86.0600	-	$C_4H_8NO^+$

Figure 4. UPLC-HR(ESI$^{-/+}$)-qTOF-MS/MS mass spectral data of known ubas#4 [4'-UB-asc-C7] (***Falcke et al., 2018***) from *P. pacificus* in both negative and positive ion modes. Characteristic ion signal for *m/z* 259.1304 containing an ureidoisobutyric acid moiety and an ascarylose unit was used for targeted MS/MS screening.

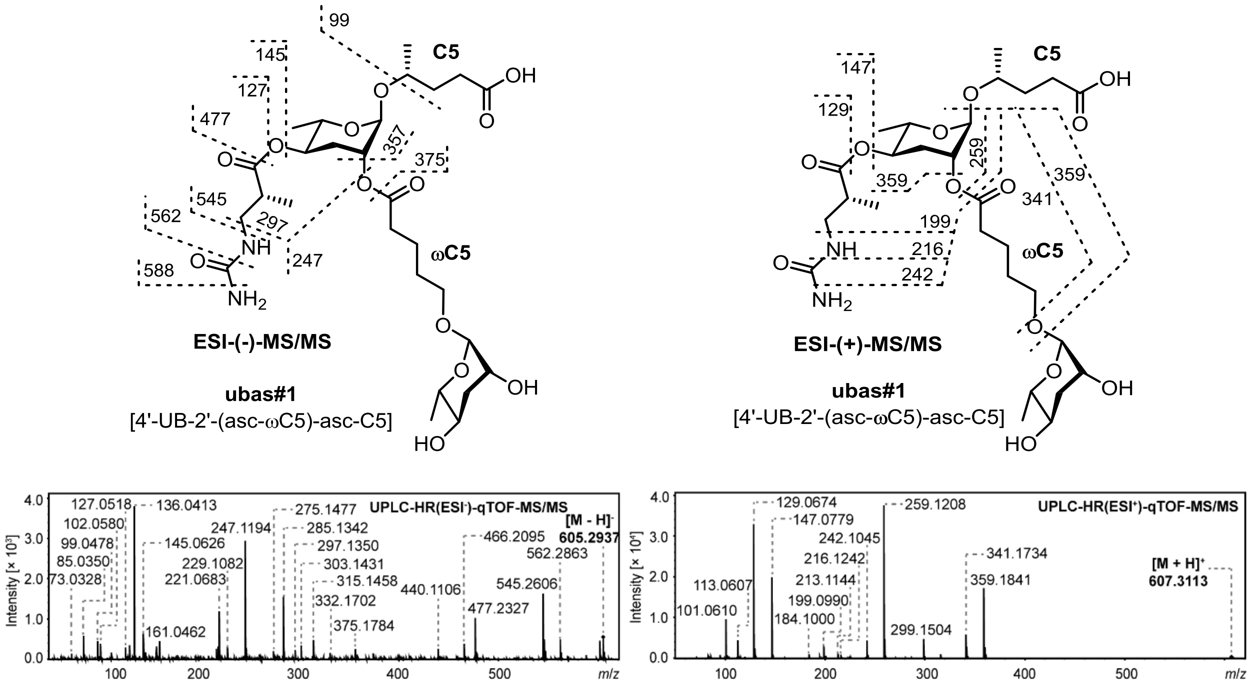

UPLC-HR(ESI^{-})-qTOF-MS/MS			
Obs. [*m/z*]	Calc. [*m/z*]	Error [ppm]	Ion Formula [M − H]$^{-}$
605.2937	605.2927	-1.7	$C_{27}H_{45}N_2O_{13}^{-}$
588.2668	588.2662	-1.2	$C_{27}H_{42}NO_{13}^{-}$
562.2863	562.2869	-5.1	$C_{26}H_{44}NO_{12}^{-}$
545.2606	545.2604	-0.4	$C_{26}H_{41}O_{12}^{-}$
477.2327	477.2341	2.9	$C_{22}H_{37}O_{11}^{-}$
466.2095	466.2083	-2.6	$C_{23}H_{32}NO_9^{-}$
440.1106	440.1198	20.9	$C_{19}H_{22}NO_{11}^{-}$
375.1784	375.1773	-3.1	$C_{16}H_{27}N_2O_8^{-}$
357.1662	357.1667	1.5	$C_{16}H_{25}N_2O_7^{-}$
332.1702	332.1715	3.7	$C_{15}H_{26}NO_7^{-}$
315.1458	315.1449	-2.8	$C_{15}H_{23}O_7^{-}$
303.1431	303.1449	6.1	$C_{14}H_{23}O_7^{-}$
297.1350	297.1344	-2.2	$C_{15}H_{21}O_6^{-}$
285.1342	285.1344	0.7	$C_{14}H_{21}O_6^{-}$
275.1477	275.1500	8.3	$C_{13}H_{23}O_6^{-}$
247.1194	247.1187	-2.8	$C_{11}H_{19}O_6^{-}$
229.1082	229.1081	-0.2	$C_{11}H_{17}O_5^{-}$
221.0683	221.0667	-7.5	$C_8H_{13}O_7^{-}$
161.0462	161.0455	-4.2	$C_6H_9O_5^{-}$
145.0626	145.0619	-5.0	$C_5H_9N_2O_3^{-}$
127.0518	127.0513	-3.9	$C_5H_7N_2O_2^{-}$
102.0580	102.0561	-	$C_4H_8NO_2^{-}$
99.0478	99.0452	-	$C_5H_7O_2^{-}$
85.0350	85.0295	-	$C_4H_5O_2^{-}$
73.0328	73.0295	-	$C_3H_5O_2^{-}$

UPLC-HR(ESI^{+})-qTOF-MS/MS			
Obs. [*m/z*]	Calc. [*m/z*]	Error [ppm]	Ion Formula [M + H]$^{+}$
607.3113	607.3073	-6.7	$C_{27}H_{47}N_2O_{13}^{+}$
359.1841	359.1813	-7.9	$C_{16}H_{27}N_2O_7^{+}$
341.1734	341.1707	-7.8	$C_{16}H_{25}N_2O_6^{+}$
316.1771	316.1755	-5.1	$C_{16}H_{26}NO_6^{+}$
299.1504	299.1489	-5.0	$C_{15}H_{23}O_6^{+}$
259.1208	259.1288	-7.6	$C_{11}H_{19}N_2O_5^{+}$
242.1045	242.1023	-9.3	$C_{11}H_{16}NO_5^{+}$
216.1242	216.1230	-5.5	$C_{10}H_{18}NO_4^{+}$
213.1144	213.1234	-	$C_{10}H_{17}N_2O_3^{+}$
199.0990	199.0965	-	$C_{10}H_{15}O_4^{+}$
184.1000	184.0968	-	$C_9H_{14}NO_3^{+}$
147.0779	147.0764	-	$C_5H_{11}N_2O_3^{+}$
129.0674	129.0659	-3.5	$C_5H_9N_2O_2^{+}$
113.0607	113.0597	-4.0	$C_6H_9O_2^{+}$
101.0610	101.0597	-	$C_5H_9O_2^{+}$

Figure 5. UPLC-HR(ESI$^{-/+}$)-qTOF-MS/MS mass spectral data of known ubas#1 [4'-UB-2'-(asc-ωC5)-asc-C5) (***supplementary file 1d – Figures 9-10***) (***Bose et al., 2012***) from *P. pacificus*.

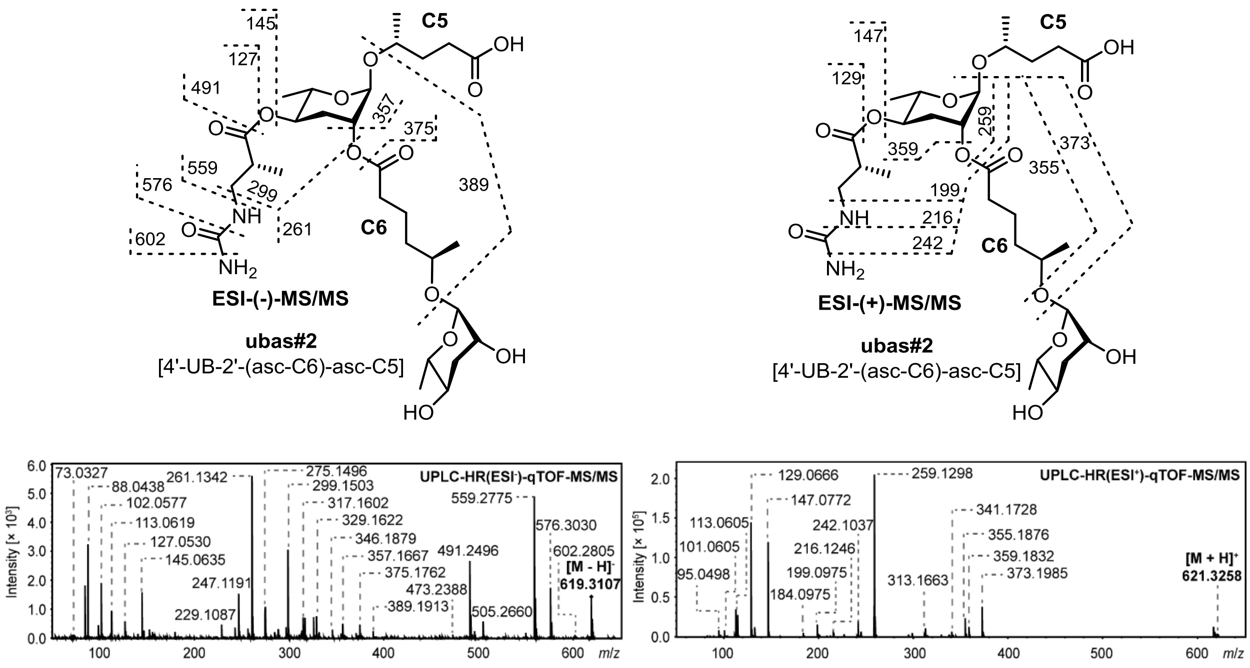

UPLC-HR(ESI$^-$)-qTOF-MS/MS			
Obs. [*m*/*z*]	Calc. [*m*/*z*]	Error [ppm]	Ion Formula [M − H]$^-$
619.3107	619.3084	-3.8	$C_{28}H_{47}N_2O_{13}^-$
602.2805	602.2818	2.2	$C_{28}H_{44}NO_{13}^-$
576.3030	576.3025	-0.7	$C_{27}H_{46}NO_{12}^-$
559.2775	559.2750	-2.6	$C_{27}H_{43}O_{12}^-$
505.2660	505.2654	-1.2	$C_{24}H_{41}O_{11}^-$
491.2496	491.2498	0.4	$C_{23}H_{39}O_{11}^-$
473.2388	473.2392	0.9	$C_{23}H_{37}O_{10}^-$
389.1913	389.1929	4.3	$C_{17}H_{29}N_2O_8^-$
375.1762	375.1773	2.9	$C_{16}H_{27}N_2O_8^-$
357.1667	357.1667	0.1	$C_{16}H_{25}N_2O_7^-$
346.1879	346.1871	-2.4	$C_{16}H_{28}NO_7^-$
329.1622	329.1606	-4.9	$C_{16}H_{25}O_7^-$
317.1602	317.1606	1.2	$C_{15}H_{25}O_7^-$
299.1503	299.1500	-1.1	$C_{15}H_{23}O_6^-$
275.1496	275.1500	1.4	$C_{13}H_{23}O_6^-$
261.1342	261.1344	0.5	$C_{12}H_{21}O_6^-$
247.1191	247.1187	-1.4	$C_{11}H_{19}O_6^-$
229.1087	229.1081	-2.6	$C_{11}H_{17}O_5^-$
145.0635	145.0619	-	$C_5H_9N_2O_3^-$
127.0530	127.0513	-	$C_5H_7N_2O_2^-$
113.0619	113.0608	-	$C_6H_9O_2^-$
102.0577	102.0561	-	$C_4H_8NO_2^-$
88.0438	88.0404	-	$C_3H_6NO_2^-$
73.0327	73.0295	-	$C_3H_5O_2^-$

UPLC-HR(ESI$^+$)-qTOF-MS/MS			
Obs. [*m*/*z*]	Calc. [*m*/*z*]	Error [ppm]	Ion Formula [M + H]$^+$
621.3258	621.3229	-4.7	$C_{28}H_{49}N_2O_{13}^+$
373.1985	373.1969	-4.2	$C_{17}H_{29}N_2O_7^+$
359.1832	359.1813	-5.3	$C_{16}H_{27}N_2O_7^+$
355.1876	355.1864	-3.6	$C_{17}H_{27}N_2O_6^+$
341.1728	341.1707	-6.1	$C_{16}H_{25}N_2O_6^+$
313.1663	313.1646	-5.7	$C_{16}H_{25}O_6^+$
259.1298	259.1288	-3.6	$C_{11}H_{19}N_2O_5^+$
242.1037	242.1023	-5.6	$C_{11}H_{16}NO_5^+$
216.1246	216.1230	-7.5	$C_{10}H_{18}NO_4^+$
199.0975	199.0965	-5.2	$C_{10}H_{15}O_4^+$
184.0975	184.0968	-3.8	$C_9H_{14}NO_3^+$
147.0772	147.0764	-5.4	$C_5H_{11}N_2O_3^+$
129.0666	129.0659	-6.1	$C_5H_9N_2O_2^+$
113.0605	113.0597	-6.8	$C_6H_9O_2^+$
101.0605	101.0597	-8.2	$C_5H_9O_2^+$
95.0498	95.0491	-6.6	$C_6H_7O^+$

Figure 6. UPLC-HR(ESI$^{-/+}$)-qTOF-MS/MS mass spectral data of known ubas#2 [4'-UB-2'-(asc-C6)-asc-C5] (***Bose et al., 2012***) from *P. pacificus* in both negative and positive ion modes.

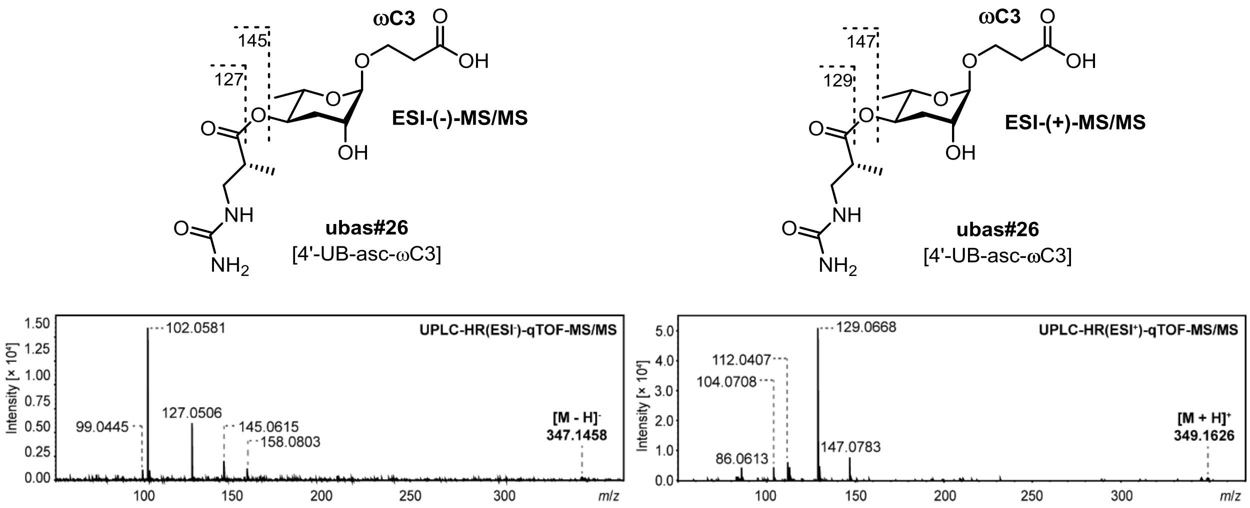

UPLC-HR(ESI⁻)-qTOF-MS/MS			
Obs. [*m*/*z*]	Calc. [*m*/*z*]	Error [ppm]	Ion Formula $[M-H]^-$
347.1458	347.1460	0.6	$C_{14}H_{23}N_2O_8^-$
158.0803	158.0823	12.1	$C_7H_{12}NO_3^-$
145.0615	145.0619	2.2	$C_5H_9N_2O_3^-$
127.0506	127.0513	5.6	$C_5H_7N_2O_2^-$
102.0581	102.0561	-	$C_4H_8NO_2^-$
99.0445	99.0452	-	$C_5H_7O_2^-$
85.0307	85.0295	-	$C_4H_5O_2^-$
73.0347	73.0295	-	$C_3H_5O_2^-$

UPLC-HR(ESI⁺)-qTOF-MS/MS			
Obs. [*m*/*z*]	Calc. [*m*/*z*]	Error [ppm]	Ion Formula $[M+H]^+$
349.1626	349.1605	-5.8	$C_{14}H_{25}N_2O_8^+$
147.0783	147.0764	-12.7	$C_5H_{11}N_2O_3^+$
129.0668	129.0659	-7.7	$C_5H_9N_2O_2^+$
112.0407	112.0393	-12.2	$C_5H_6NO_2^+$
104.0708	104.0706	-2.3	$C_4H_{10}NO_2^+$
86.0613	86.0600	-	$C_4H_8NO^+$

Figure 7. UPLC-HR(ESI$^{-/+}$)-qTOF-MS/MS mass spectral data of newly identified ubas#26 [4'-UB-asc-ωC3] in both negative and positive ion modes.

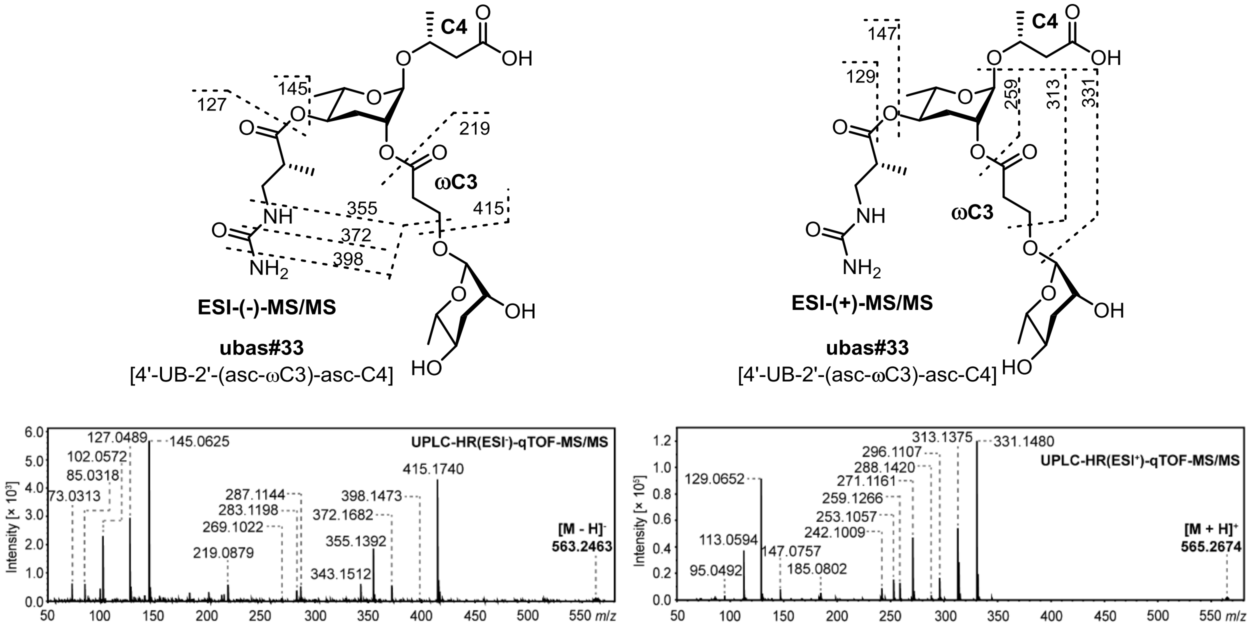

UPLC-HR(ESI$^-$)-qTOF-MS/MS			
Obs. [m/z]	Calc. [m/z]	Error [ppm]	Ion Formula [M − H]$^-$
563.2463	563.2458	-1.0	$C_{24}H_{39}N_2O_{13}^-$
415.1740	415.1722	-4.3	$C_{18}H_{27}N_2O_9^-$
398.1473	398.1457	-4.1	$C_{18}H_{23}NO_9^-$
372.1682	372.1664	-4.9	$C_{17}H_{26}NO_8^-$
355.1392	355.1398	1.7	$C_{17}H_{23}O_8^-$
343.1512	347.1511	-0.3	$C_{15}H_{23}N_2O_7^-$
287.1144	287.1136	-2.8	$C_{13}H_{19}O_7^-$
283.1198	283.1187	-3.8	$C_{14}H_{19}O_6^-$
269.1022	269.1031	3.2	$C_{13}H_{17}O_6^-$
219.0879	219.0874	-2.1	$C_9H_{15}O_6^-$
145.0625	145.0619	-4.2	$C_5H_9N_2O_3^-$
127.0489	127.0513	-	$C_5H_7N_2O_2^-$
102.0572	102.0561	-	$C_4H_8NO_2^-$
85.0318	85.0295	-	$C_4H_5O_2^-$
73.0313	73.0295	-	$C_3H_5O_2^-$

UPLC-HR(ESI$^+$)-qTOF-MS/MS			
Obs. [m/z]	Calc. [m/z]	Error [ppm]	Ion Formula [M + H]$^+$
565.2674	565.2603	5.1	$C_{24}H_{41}N_2O_{13}^+$
331.1480	331.1500	6.0	$C_{14}H_{23}N_2O_7^+$
313.1375	313.1394	6.2	$C_{14}H_{21}N_2O_6^+$
296.1107	296.1129	7.2	$C_{14}H_{18}NO_6^+$
288.1420	288.1442	7.4	$C_{13}H_{22}NO_6^+$
271.1161	271.1176	5.5	$C_{13}H_{19}O_6^+$
259.1266	259.1288	8.7	$C_{11}H_{19}N_2O_5^+$
253.1057	253.1071	5.5	$C_{13}H_{17}O_5^+$
242.1009	242.1023	5.6	$C_{11}H_{16}NO_5^+$
185.0802	185.0808	3.5	$C_9H_{13}O_4^+$
147.0757	147.0764	5.2	$C_5H_{11}N_2O_3^+$
129.0652	129.0659	5.1	$C_5H_9N_2O_2^+$
113.0594	113.0597	3.0	$C_6H_9O_2^+$
95.0492	95.0491	-0.9	$C_6H_7O^+$

Figure 8. UPLC-HR(ESI$^{-/+}$)-qTOF-MS/MS mass spectral data of ubas#33 [4'-UB-2'-(asc-ωC3)-asc-C4] in both negative and positive ion modes.

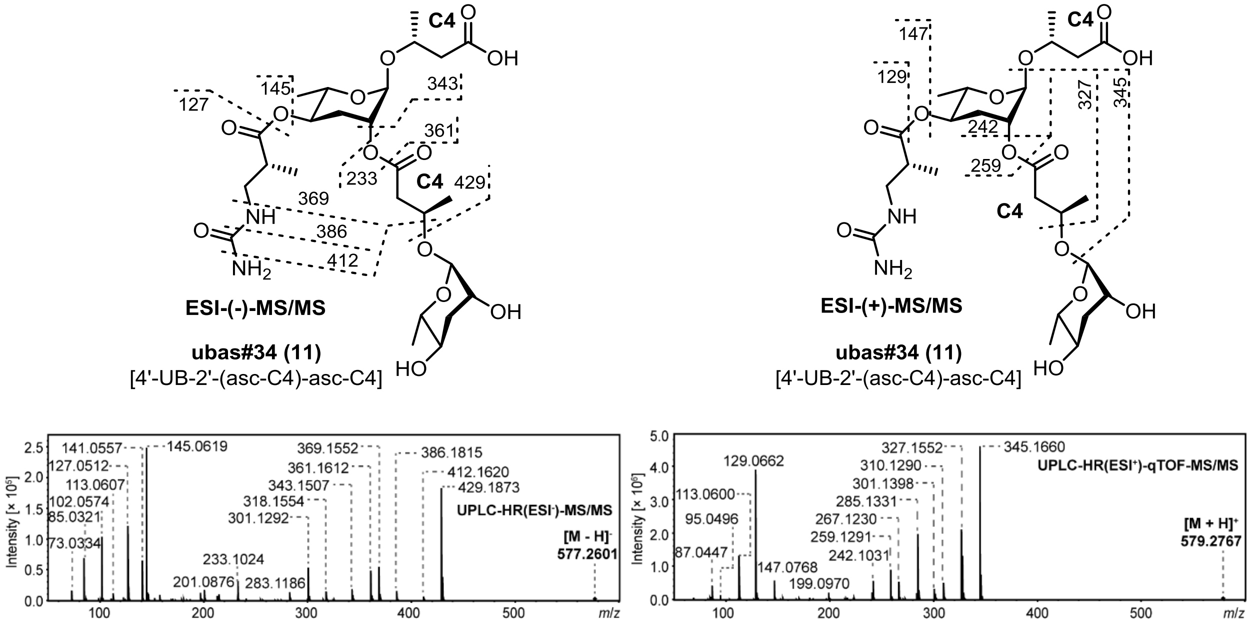

UPLC-HR(ESI⁻)-qTOF-MS/MS			
Obs. [*m*/*z*]	Calc. [*m*/*z*]	Error [ppm]	Ion Formula [M − H]⁻
577.2601	577.2614	-2.3	$C_{25}H_{41}N_2O_{13}^-$
429.1873	429.1879	1.3	$C_{19}H_{29}N_2O_9^-$
412.1620	412.1613	-1.7	$C_{19}H_{26}NO_9^-$
386.1815	386.1820	1.3	$C_{18}H_{28}NO_8^-$
369.1552	369.1555	0.8	$C_{18}H_{25}O_8^-$
361.1612	361.1616	1.2	$C_{15}H_{25}N_2O_8^-$
343.1507	343.1511	1.0	$C_{15}H_{23}N_2O_7^-$
318.1554	318.1558	1.2	$C_{14}H_{24}NO_7^-$
301.1292	301.1293	0.1	$C_{14}H_{21}O_7^-$
283.1186	283.1187	0.5	$C_{14}H_{19}O_6^-$
233.1024	233.1031	2.7	$C_{10}H_{17}O_6^-$
201.0876	201.0881	2.4	$C_8H_{13}N_2O_4^-$
145.0619	145.0619	0.0	$C_5H_9N_2O_3^-$
141.0557	141.0557	0.0	$C_5H_9O_3^-$
127.0512	127.0513	0.6	$C_5H_7N_2O_2^-$
113.0607	113.0608	0.9	$C_6H_9O_2^-$
102.0574	102.0561	-	$C_4H_8NO_2^-$
85.0321	85.0295	-	$C_4H_5O_2^-$
73.0334	73.0295	-	$C_3H_5O_2^-$

UPLC-HR(ESI⁺)-qTOF-MS/MS			
Obs. [*m*/*z*]	Calc. [*m*/*z*]	Error [ppm]	Ion Formula [M + H]⁺
579.2767	579.2760	-1.2	$C_{25}H_{43}N_2O_{13}^+$
345.1660	345.1656	-1.0	$C_{15}H_{25}N_2O_7^+$
327.1552	327.1551	-0.4	$C_{15}H_{23}N_2O_6^+$
310.1290	310.1285	-1.4	$C_{15}H_{20}NO_6^+$
301.1398	301.1394	-1.3	$C_{13}H_{21}N_2O_6^+$
285.1331	285.1333	0.5	$C_{14}H_{21}O_6^+$
267.1230	267.1227	-1.0	$C_{14}H_{19}O_5^+$
259.1291	259.1288	-0.8	$C_{11}H_{19}N_2O_5^+$
242.1031	242.1023	-3.4	$C_{11}H_{16}NO_5^+$
199.0970	199.0965	-2.5	$C_{10}H_{15}O_4^+$
147.0768	147.0764	-2.5	$C_5H_{11}N_2O_3^+$
129.0662	129.0659	-2.4	$C_5H_9N_2O_2^+$
113.0600	113.0597	-3.0	$C_6H_9O_2^+$
95.0496	95.0491	-5.3	$C_6H_7O^+$
87.0447	87.0441	-	$C_4H_7O_2^+$

Figure 9. UPLC-HR(ESI⁻/⁺)-qTOF-MS/MS mass spectral data of ubas#34 [4'-UB-2'-(asc-C4)-asc-C4, **11**] in both negative and positive ion modes. UPLC-HR(ESI⁺)-qTOF-MS/MS fragmentation produced a fragment ion signal for $C_{15}H_{25}N_2O_7^+$ (*m/z* 345.1660 [M + H]⁺, *Δ* -1.0 ppm) (***Figure 5 – figure supplement 3***), indicating that ascr#11 [asc-C4] represents the first ascaroside in ubas#34 [4'-UB-2'-(asc-C4)-asc-C4, **11**].

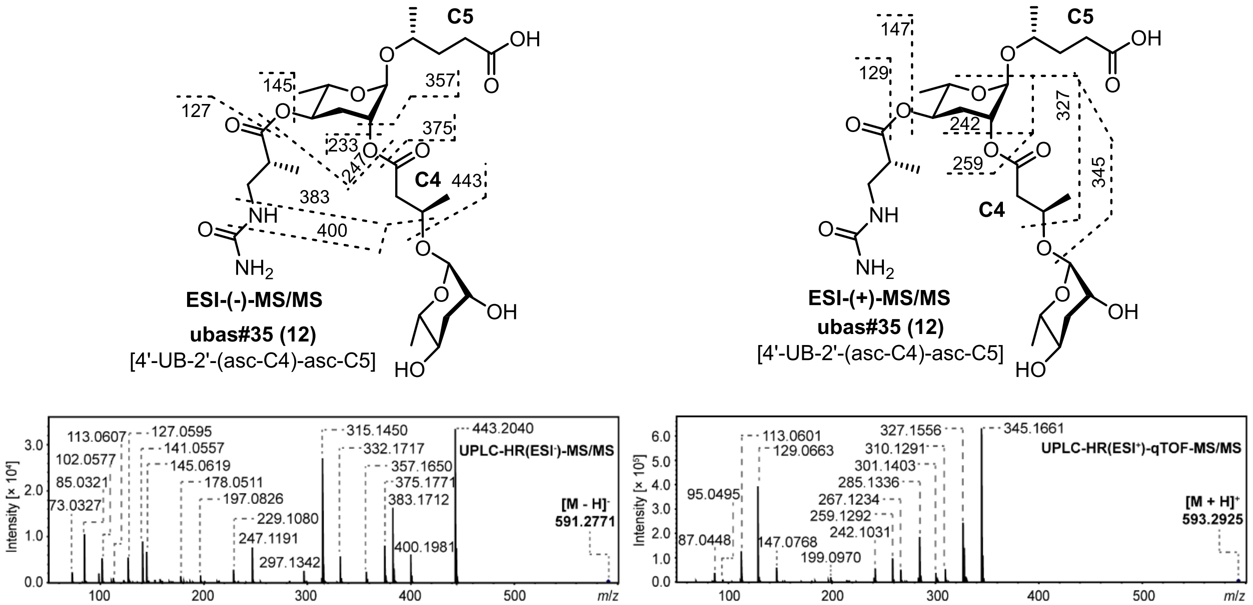

UPLC-HR(ESI⁻)-qTOF-MS/MS			
Obs. [m/z]	Calc. [m/z]	Error [ppm]	Ion Formula [M − H]$^-$
591.2771	591.2771	0.0	$C_{26}H_{43}N_2O_{13}^-$
443.2040	443.2035	-1.2	$C_{20}H_{31}N_2O_9^-$
400.1981	400.1977	-0.9	$C_{19}H_{30}NO_8^-$
383.1712	383.1711	-0.3	$C_{19}H_{27}O_8^-$
375.1771	375.1773	0.5	$C_{16}H_{27}N_2O_8^-$
357.1650	357.1667	4.7	$C_{16}H_{25}N_2O_7^-$
332.1717	332.1715	-0.8	$C_{15}H_{26}NO_7^-$
315.1450	315.1449	-0.2	$C_{15}H_{23}O_7^-$
297.1342	297.1344	0.6	$C_{15}H_{21}O_6^-$
247.1191	247.1187	-1.5	$C_{11}H_{19}O_6^-$
229.1080	229.1081	0.5	$C_{11}H_{17}O_5^-$
197.0826	197.0819	-3.1	$C_{10}H_{13}O_4^-$
178.0511	178.0510	-0.8	$C_9H_8NO_3^-$
145.0619	145.0619	-0.1	$C_5H_9N_2O_3^-$
141.0557	141.0557	0.3	$C_7H_9O_3^-$
127.0595	127.0608	-0.8	$C_5H_7N_2O_2^-$
113.0607	113.0608	-	$C_6H_9O_2^-$
102.0577	102.0561	-	$C_4H_8NO_2^-$
85.0321	85.0295	-	$C_4H_5O_2^-$
73.0327	73.0295	-	$C_3H_5O_2^-$

UPLC-HR(ESI⁺)-qTOF-MS/MS			
Obs. [m/z]	Calc. [m/z]	Error [ppm]	Ion Formula [M + H]$^+$
593.2925	593.2916	-1.4	$C_{26}H_{45}N_2O_{13}^+$
345.1661	345.1656	-1.3	$C_{15}H_{25}N_2O_7^+$
327.1556	327.1551	-1.7	$C_{15}H_{23}N_2O_6^+$
310.1291	310.1285	-1.7	$C_{15}H_{20}NO_6^+$
301.1403	301.1394	-3.1	$C_{13}H_{21}N_2O_6^+$
285.1336	285.1333	-1.2	$C_{14}H_{21}O_6^+$
267.1234	267.1227	-2.6	$C_{14}H_{19}O_5^+$
259.1292	259.1288	-1.3	$C_{11}H_{19}N_2O_5^+$
242.1031	242.1023	-3.5	$C_{11}H_{16}NO_5^+$
199.0970	199.0965	-2.5	$C_{10}H_{15}O_4^+$
147.0768	147.0764	-2.8	$C_5H_{11}N_2O_3^+$
129.0663	129.0659	-3.4	$C_5H_9N_2O_2^+$
113.0601	113.0597	-3.3	$C_6H_9O_2^+$
95.0495	95.0491	-5.3	$C_6H_7O^+$
87.0448	87.0441	-	$C_4H_7O_2^+$

Figure 10. UPLC-HR(ESI$^{-/+}$)-qTOF-MS/MS mass spectral data of ubas#35 [4'-UB-2'-(asc-C4)-asc-C5, **12**] in both negative and positive ion modes. UPLC-HR(ESI$^+$)-qTOF-MS/MS fragmentation produced a fragment ion signal for $C_{15}H_{25}N_2O_7^+$ (*m/z* 345.1660 [M + H]$^+$, *Δ* -1.0 ppm) (***Figure 5 – figure supplement 3***), indicating that ascr#11 [asc-C4] represents the first ascaroside in ubas#35 [4'-UB-2'-(asc-C4)-asc-C5, **12**].

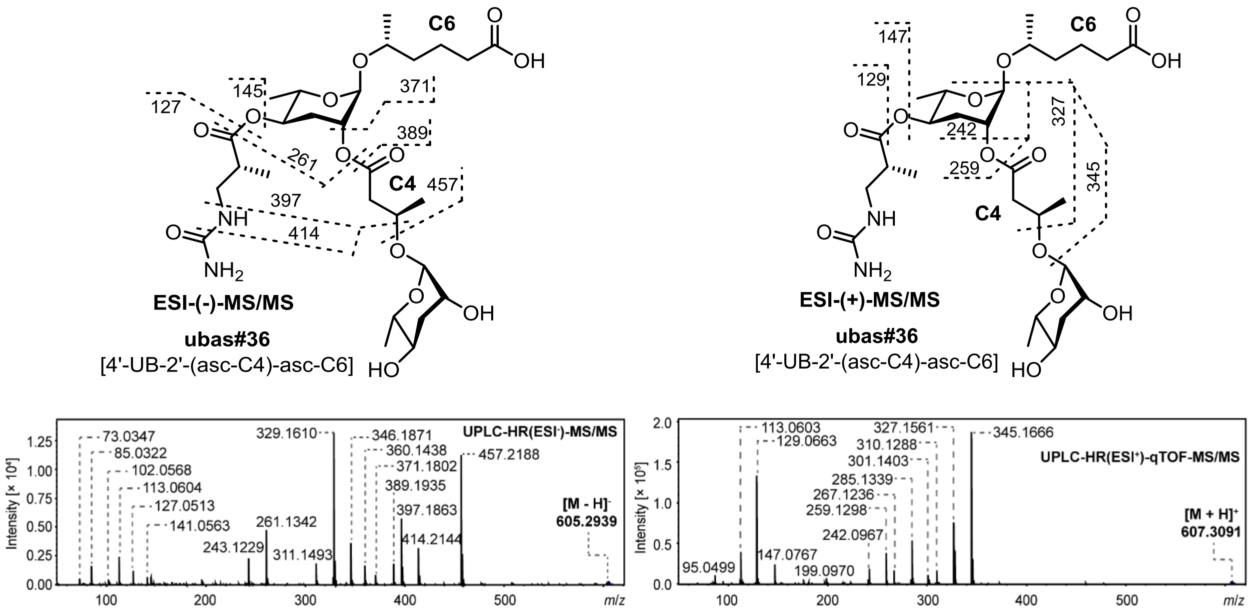

UPLC-HR(ESI⁻)-qTOF-MS/MS			
Obs. [m/z]	Calc. [m/z]	Error [ppm]	Ion Formula $[M-H]^-$
605.2939	605.2927	-2.0	$C_{27}H_{45}N_2O_{13}^-$
457.2188	457.2192	0.7	$C_{21}H_{33}N_2O_9^-$
414.2144	414.2133	-2.5	$C_{20}H_{32}NO_8^-$
397.1863	397.1868	1.2	$C_{20}H_{29}O_8^-$
389.1935	389.1929	-1.3	$C_{17}H_{29}N_2O_8^-$
371.1802	371.1824	5.7	$C_{17}H_{27}N_2O_7^-$
360.1438	360.1453	4.2	$C_{19}H_{22}NO_6^-$
346.1871	346.1871	0.2	$C_{16}H_{28}NO_7^-$
329.1610	329.1606	-1.3	$C_{16}H_{25}O_7^-$
311.1493	311.1500	2.4	$C_{16}H_{23}O_6^-$
261.1342	261.1344	0.6	$C_{12}H_{21}O_6^-$
243.1229	243.1238	3.8	$C_{12}H_{19}O_5^-$
141.0563	141.0557	-4.2	$C_7H_9O_3^-$
127.0513	127.0513	0.4	$C_5H_7N_2O_2^-$
113.0604	113.0608	3.1	$C_6H_9O_2^-$
102.0568	102.0561	-	$C_4H_8NO_2^-$
85.0322	85.0295	-	$C_4H_5O_2^-$
73.0347	73.0295	-	$C_3H_5O_2^-$

UPLC-HR(ESI⁺)-qTOF-MS/MS			
Obs. [m/z]	Calc. [m/z]	Error [ppm]	Ion Formula $[M+H]^+$
607.3091	607.3073	-3.0	$C_{27}H_{47}N_2O_{13}^+$
345.1666	345.1656	-2.8	$C_{15}H_{25}N_2O_7^+$
327.1561	327.1551	-3.1	$C_{15}H_{23}N_2O_6^+$
310.1288	310.1285	-1.0	$C_{15}H_{20}NO_6^+$
301.1403	301.1394	-2.8	$C_{13}H_{21}N_2O_6^+$
285.1339	285.1333	-2.1	$C_{14}H_{21}O_6^+$
267.1236	267.1227	-2.6	$C_{14}H_{19}O_5^+$
259.1298	259.1288	-1.3	$C_{11}H_{19}N_2O_5^+$
242.0967	242.1023	23.3	$C_{11}H_{16}NO_5^+$
199.0970	199.0965	-2.6	$C_{10}H_{15}O_4^+$
147.0767	147.0764	-2.1	$C_5H_{11}N_2O_3^+$
129.0663	129.0659	-3.4	$C_5H_9N_2O_2^+$
113.0603	113.0597	-5.1	$C_6H_9O_2^+$
95.0499	95.0491	-	$C_6H_7O^+$

Figure 11. UPLC-HR($ESI^{-/+}$)-qTOF-MS/MS mass spectral data of ubas#36 [4'-UB-2'-(asc-C4)-asc-C6] in both negative and positive ion modes. UPLC-HR(ESI^+)-qTOF-MS/MS fragmentation produced a fragment ion signal for $C_{15}H_{25}N_2O_7^+$ (*m/z* 345.1660 $[M+H]^+$, *Δ* -1.0 ppm) (***Figure 5 – figure supplement 3***), indicating that ascr#11 [asc-C4] represents the first ascaroside in ubas#36 [4'-UB-2'-(asc-C4)-asc-C6].

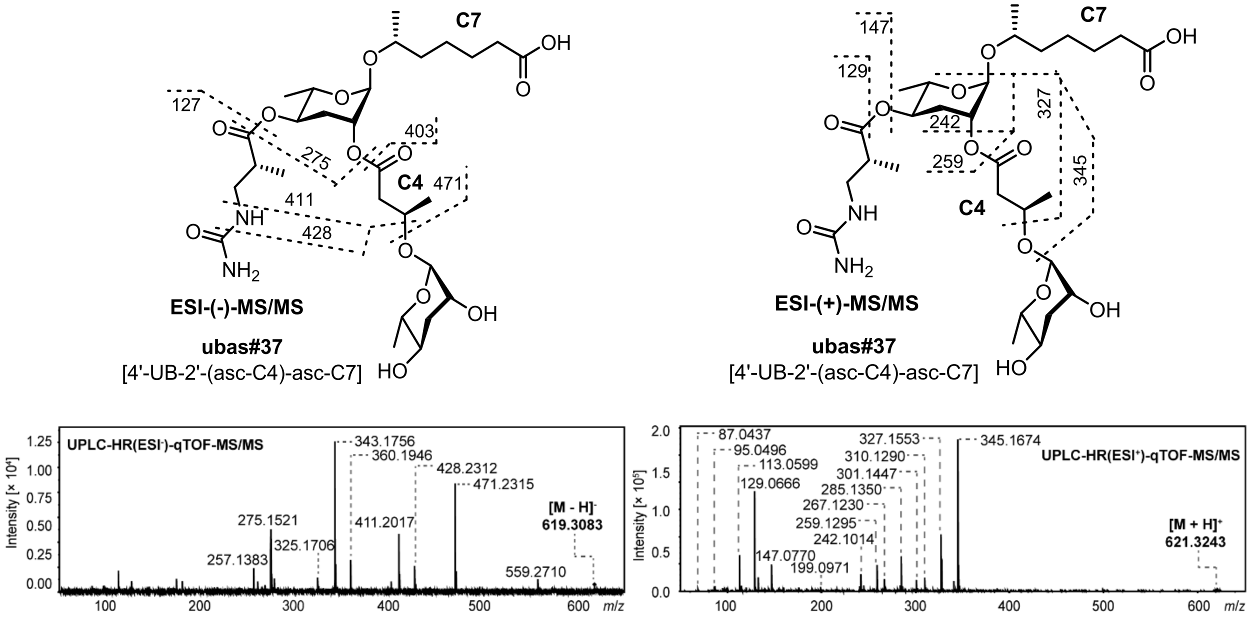

UPLC-HR(ESI⁻)-qTOF-MS/MS			
Obs. [*m/z*]	Calc. [*m/z*]	Error [ppm]	Ion Formula $[M-H]^-$
619.3083	619.3084	0.2	$C_{28}H_{47}N_2O_{13}^-$
471.2315	471.2348	7.1	$C_{22}H_{35}N_2O_9^-$
428.2312	428.2290	-5.2	$C_{21}H_{34}NO_8^-$
411.2017	411.2024	1.7	$C_{21}H_{31}O_8^-$
343.1756	343.1762	1.7	$C_{17}H_{27}O_7^-$
325.1706	325.1657	-	$C_{17}H_{25}O_6^-$
275.1521	275.1500	-7.4	$C_{13}H_{23}O_6^-$
257.1383	257.1394	4.3	$C_{13}H_{21}O_5^-$

UPLC-HR(ESI⁺)-qTOF-MS/MS			
Obs. [*m/z*]	Calc. [*m/z*]	Error [ppm]	Ion Formula $[M+H]^+$
621.3243	621.3229	-2.2	$C_{28}H_{49}N_2O_{13}^+$
345.1674	345.1656	-5.1	$C_{15}H_{25}N_2O_7^+$
327.1553	327.1551	-0.8	$C_{15}H_{23}N_2O_6^+$
310.1290	310.1285	-1.6	$C_{15}H_{20}NO_6^+$
301.1447	301.1394	-17.4	$C_{13}H_{21}N_2O_6^+$
285.1350	285.1333	-6.0	$C_{14}H_{21}O_6^+$
267.1230	267.1227	-1.1	$C_{14}H_{19}O_5^+$
259.1295	259.1288	-2.6	$C_{11}H_{19}N_2O_5^+$
242.1014	242.1023	3.6	$C_{11}H_{16}NO_5^+$
199.0971	199.0965	-3.0	$C_{10}H_{15}O_4^+$
147.0770	147.0764	-3.7	$C_5H_{11}N_2O_3^+$
129.0666	129.0659	-5.6	$C_5H_9N_2O_2^+$
113.0599	113.0597	-2.1	$C_6H_9O_2^+$
95.0496	95.0491	-4.6	$C_6H_7O^+$
87.0437	87.0441	-3.9	$C_4H_7O_2^+$

Figure 12. UPLC-HR(ESI$^{-/+}$)-qTOF-MS/MS mass spectral data of ubas#37 [4'-UB-2'-(asc-C4)-asc-C7] in both negative and positive ion modes. UPLC-HR(ESI$^+$)-qTOF-MS/MS fragmentation produced a fragment ion signal for $C_{15}H_{25}N_2O_7^+$ (*m/z* 345.1660 $[M+H]^+$, *Δ* -1.0 ppm) (***Figure 5 – figure supplement 3***), indicating that ascr#11 [asc-C4] represents the first ascaroside in ubas#37 [4'-UB-2'-(asc-C4)-asc-C7].

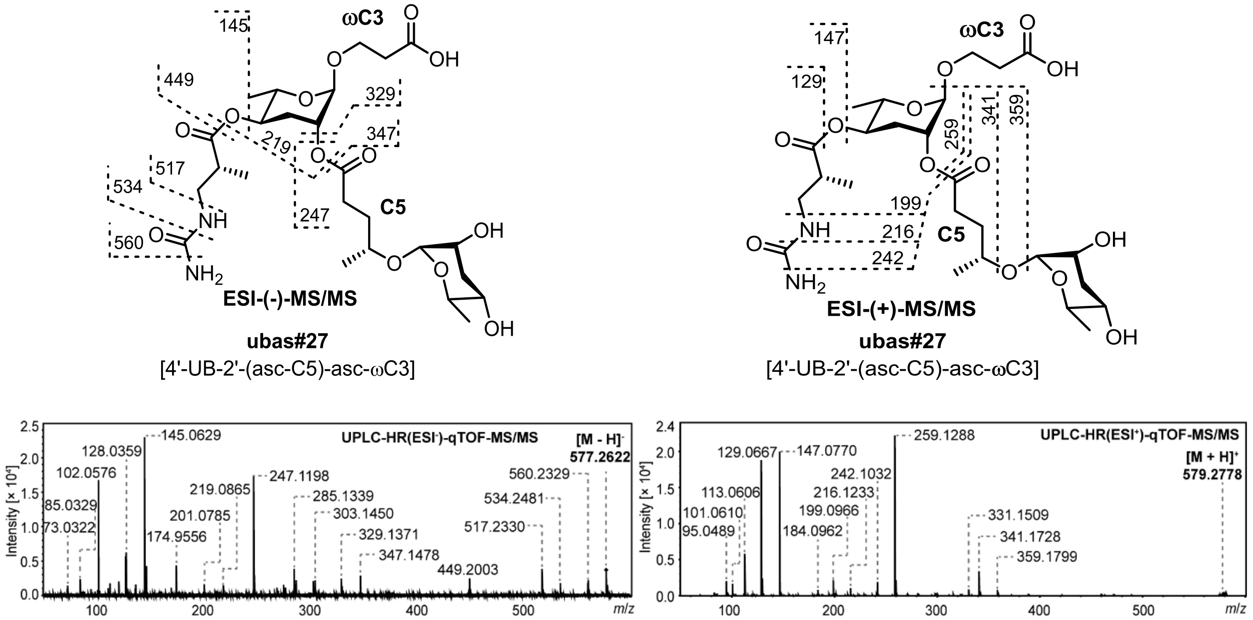

UPLC-HR(ESI$^-$)-qTOF-MS/MS			
Obs. [*m*/*z*]	Calc. [*m*/*z*]	Error [ppm]	Ion Formula [M − H]$^-$
577.2622	577.2614	-1.3	$C_{25}H_{41}N_2O_{13}^-$
560.2329	560.2349	3.5	$C_{25}H_{38}NO_{13}^-$
534.2481	534.2556	14.0	$C_{24}H_{40}NO_{12}^-$
517.2330	517.2291	-7.7	$C_{24}H_{37}O_{12}^-$
449.2003	449.2028	5.6	$C_{20}H_{33}O_{11}^-$
347.1478	347.1460	-5.3	$C_{14}H_{23}N_2O_8^-$
329.1371	329.1354	-5.0	$C_{14}H_{21}N_2O_7^-$
303.1450	303.1449	-0.3	$C_{14}H_{23}O_7^-$
285.1339	285.1344	1.5	$C_{14}H_{21}O_6^-$
247.1198	247.1187	-4.5	$C_{11}H_{19}O_6^-$
219.0865	219.0874	4.2	$C_9H_{15}O_6^-$
201.0785	201.0768	-8.4	$C_9H_{13}O_5^-$
145.0629	145.0619	-7.1	$C_5H_9N_2O_3^-$
128.0359	128.0353	-4.8	$C_5H_6NO_3^-$
102.0576	102.1561	-	$C_4H_8NO_2^-$
85.0329	85.0407	-	$C_4H_5O_2^-$
73.0322	73.0295	-	$C_3H_5O_2^-$

UPLC-HR(ESI$^+$)-qTOF-MS/MS			
Obs. [*m*/*z*]	Calc. [*m*/*z*]	Error [ppm]	Ion Formula [M + H]$^+$
579.2778	579.2760	-3.1	$C_{25}H_{43}N_2O_{13}^+$
359.1799	359.1813	3.8	$C_{16}H_{27}N_2O_7^+$
341.1728	341.1707	-6.1	$C_{16}H_{25}N_2O_6^+$
331.1509	331.1500	-2.7	$C_{14}H_{23}N_2O_7^+$
259.1296	259.1288	-3.0	$C_{11}H_{19}N_2O_5^+$
242.1032	242.1023	-3.9	$C_{11}H_{16}NO_5^+$
216.1233	216.1230	-1.0	$C_{10}H_{18}NO_4^+$
199.0966	199.0965	-0.5	$C_{10}H_{15}O_4^+$
184.0962	184.0968	3.5	$C_9H_{14}NO_3^+$
147.0770	147.0764	-3.9	$C_5H_{11}N_2O_3^+$
129.0667	129.0659	-	$C_5H_9N_2O_2^+$
113.0606	113.0597	-	$C_6H_9O_2^+$
101.0610	101.0597	-	$C_5H_9O_2^+$
95.0489	95.0491	2.3	$C_6H_7O^+$

Figure 13. UPLC-HR(ESI$^{-/+}$)-qTOF-MS/MS mass spectral data of ubas#27 [4'-UB-2'-(asc-C5)-asc-ωC3] in both negative and positive ion modes.

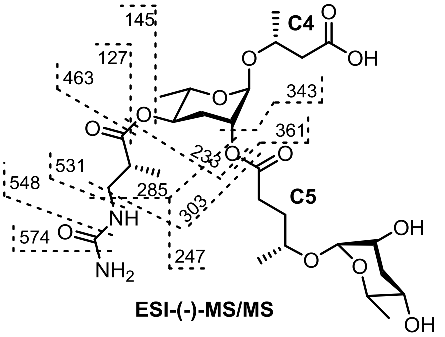

ubas#28 (13)
[4'-UB-2'-(asc-C5)-asc-C4]

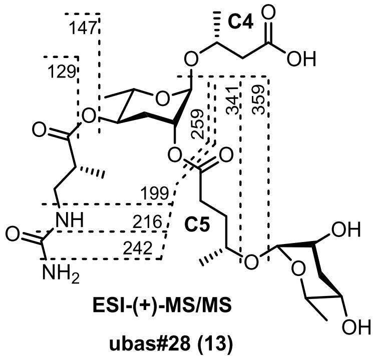

ubas#28 (13)
[4'-UB-2'-(asc-C5)-asc-C4]

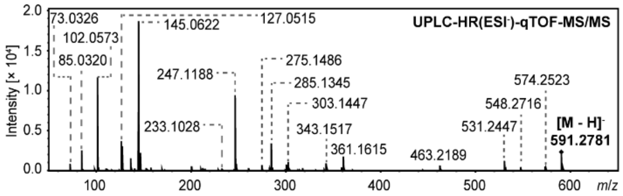

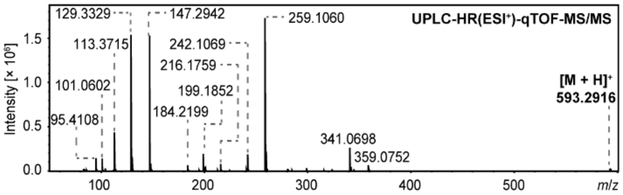

UPLC-HR(ESI⁻)-qTOF-MS/MS			
Obs. [*m*/*z*]	Calc. [*m*/*z*]	Error [ppm]	Ion Formula [M − H]-
591.2781	591.2771	-1.7	$C_{26}H_{43}N_2O_{13}^-$
574.2523	574.2505	-3.1	$C_{26}H_{40}NO_{13}^-$
548.2716	548.2712	-0.6	$C_{25}H_{42}NO_{12}^-$
531.2447	531.2447	0.0	$C_{25}H_{39}O_{12}^-$
463.2189	463.2185	-0.9	$C_{21}H_{35}O_{11}^-$
361.1615	361.1616	0.5	$C_{15}H_{25}N_2O_8^-$
343.1517	343.1511	-1.9	$C_{15}H_{23}N_2O_7^-$
303.1447	303.1449	0.7	$C_{14}H_{23}O_7^-$
285.1345	285.1344	-0.3	$C_{14}H_{21}O_6^-$
275.1486	275.1500	5.0	$C_{13}H_{23}O_6^-$
247.1188	247.1187	-0.2	$C_{11}H_{19}O_6^-$
233.1028	233.1030	1.1	$C_{10}H_{17}O_6^-$
213.0881	213.0881	0.1	$C_9H_{13}N_2O_4^-$
145.0622	145.0619	-2.5	$C_5H_9N_2O_3^-$
127.0515	127.0513	-1.9	$C_5H_7N_2O_2^-$
102.0573	102.0561	-	$C_4H_8NO_2^-$
85.0320	85.0295	-	$C_4H_5O_2^-$
73.0326	73.0295	-	$C_3H_5O_2^-$

UPLC-HR(ESI⁺)-qTOF-MS/MS			
Obs. [*m*/*z*]	Calc. [*m*/*z*]	Error [ppm]	Ion Formula [M + H]+
593.2916	593.1916	0.1	$C_{26}H_{45}N_2O_{13}^+$
359.1818	359.1813	-1.5	$C_{16}H_{27}N_2O_7^+$
341.1710	341.1707	-0.7	$C_{16}H_{25}N_2O_6^+$
259.1290	259.1288	-0.5	$C_{11}H_{19}N_2O_5^+$
242.1028	242.1023	-2.1	$C_{11}H_{16}NO_5^+$
216.1234	216.1230	-1.8	$C_{10}H_{18}NO_4^+$
199.0967	199.0965	-1.0	$C_{10}H_{15}O_4^+$
184.0971	184.0968	-1.5	$C_9H_{14}NO_3^+$
147.0766	147.0780	-1.5	$C_5H_{11}N_2O_3^+$
129.0662	129.0659	-2.3	$C_5H_9N_2O_2^+$
113.0600	113.0597	-3.0	$C_6H_9O_2^+$
101.0602	101.0597	-5.4	$C_5H_9O_2^+$
95.0496	95.0491	-4.8	$C_6H_7O^+$

Figure 14. UPLC-HR(ESI$^{-/+}$)-qTOF-MS/MS mass spectral data of ubas#28 [4'-UB-2'-(asc-C5)-asc-C4, **13**] in both negative and positive ion modes.

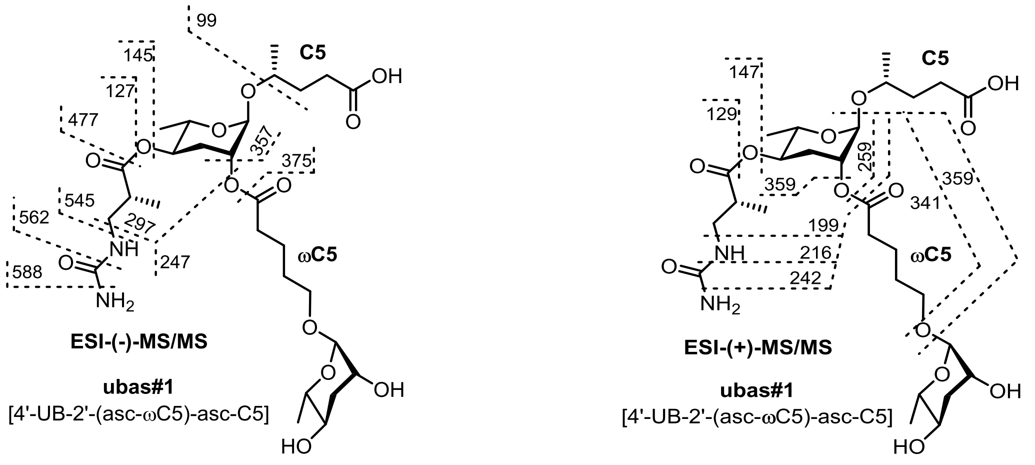

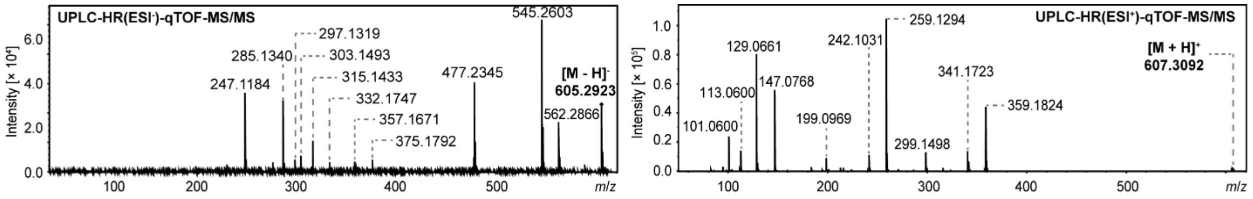

UPLC-HR(ESI⁻)-qTOF-MS/MS			
Obs. [*m/z*]	Calc. [*m/z*]	Error [ppm]	Obs. [*m/z*]
605.2923	605.2927	0.5	$C_{27}H_{45}N_2O_{13}^-$
562.2866	562.2869	0.3	$C_{26}H_{44}NO_{12}^-$
545.2603	545.2604	0.0	$C_{26}H_{41}O_{12}^-$
477.2345	477.2341	-0.3	$C_{22}H_{37}O_{11}^-$
375.1792	375.1773	-1.9	$C_{16}H_{27}N_2O_8^-$
357.1671	357.1667	-0.4	$C_{16}H_{25}N_2O_7^-$
332.1747	332.1715	-3.3	$C_{15}H_{26}NO_7^-$
315.1433	315.1449	1.6	$C_{15}H_{23}O_7^-$
303.1593	303.1449	0.9	$C_{14}H_{23}O_7^-$
297.1319	297.1370	2.5	$C_{15}H_{21}O_6^-$
285.1340	285.1344	0.4	$C_{14}H_{21}O_6^-$
275.1537	275.1500	-3.7	$C_{13}H_{23}O_6^-$
247.1184	247.1088	0.3	$C_{11}H_{19}O_6^-$
229.1101	229.1081	-2.0	$C_{11}H_{17}O_5^-$

UPLC-HR(ESI⁺)-qTOF-MS/MS			
Error [ppm]	Obs. [*m/z*]	Calc. [*m/z*]	Error [ppm]
607.3040	607.3073	5.3	$C_{27}H_{47}N_2O_{13}^+$
359.1801	359.1813	3.4	$C_{16}H_{27}N_2O_7^+$
341.1701	341.1707	1.7	$C_{16}H_{25}N_2O_6^+$
316.1749	316.1755	1.9	$C_{15}H_{26}NO_6^+$
299.1483	299.1489	2.1	$C_{15}H_{23}O_6^+$
259.1284	259.1288	-3.1	$C_{11}H_{19}N_2O_5^+$
242.1024	242.1023	-0.4	$C_{11}H_{16}NO_5^+$
199.0968	199.0965	-1.4	$C_{10}H_{15}O_4^+$
184.0965	184.0968	1.5	$C_9H_{14}NO_3^+$
147.0769	147.0764	-3.2	$C_5H_{11}N_2O_3^+$
129.0664	129.0659	-4.4	$C_5H_9N_2O_2^+$
113.0602	113.0597	-4.4	$C_6H_9O_2^+$
101.0606	101.0597	-8.7	$C_5H_9O_2^+$
95.0500	95.0491	-8.7	$C_6H_7O^+$

Figure 15. UPLC-HR(ESI$^{-/+}$)-qTOF-MS/MS mass spectral data of ubas#1 [4'-UB-2'-(asc-ωC5)-asc-C5] (from *P. taiwanensis*) in both negative and positive ion modes. Analysis of NMR data of ubas#1 isolated from the *exo*-metabolome of *P. taiwanensis* has confirmed its chemical identity (***supplementary file 1d – Figures 12-15***).

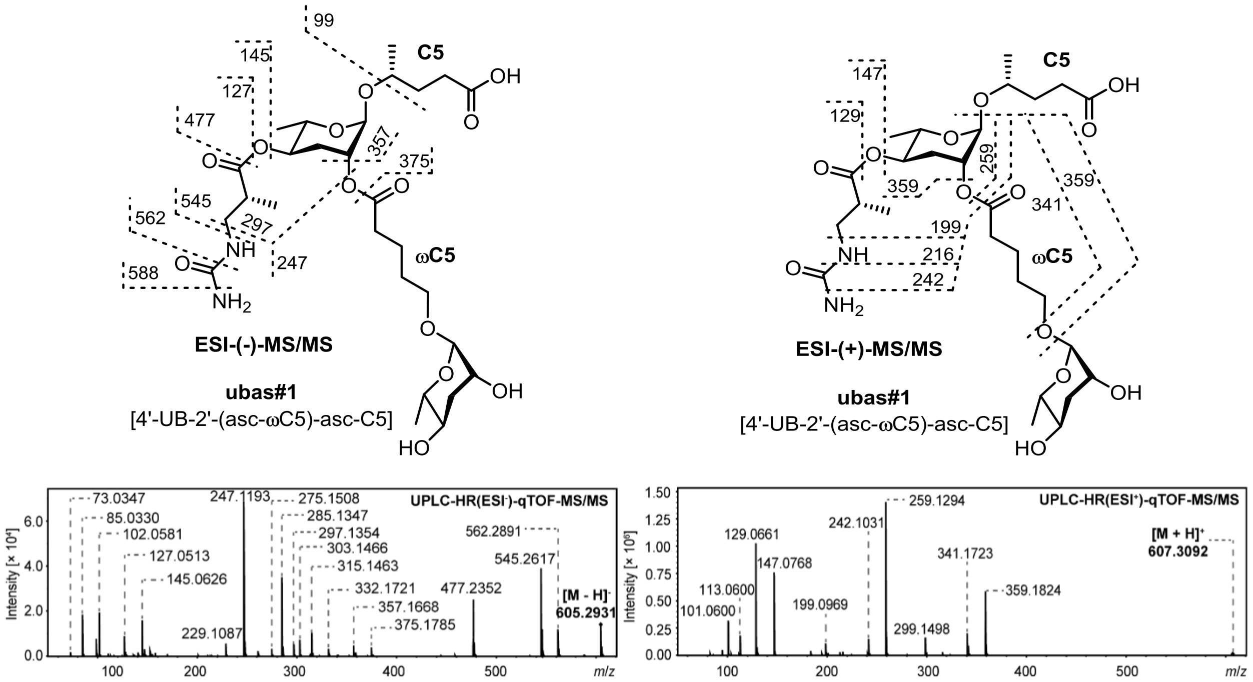

UPLC-HR(ESI⁻)-qTOF-MS/MS			
Obs. [*m/z*]	Calc. [*m/z*]	Error [ppm]	Ion Formula [M − H]⁻
605.2931	605.2927	-0.7	$C_{27}H_{45}N_2O_{13}^-$
562.2891	562.2869	-3.8	$C_{26}H_{44}NO_{12}^-$
545.2617	545.2604	-2.4	$C_{26}H_{41}O_{12}^-$
477.2352	477.2341	-2.2	$C_{22}H_{37}O_{11}^-$
375.1785	375.1773	-3.2	$C_{16}H_{27}N_2O_8^-$
357.1668	357.1667	-0.3	$C_{16}H_{25}N_2O_7^-$
332.1721	332.1715	-1.9	$C_{15}H_{26}NO_7^-$
315.1463	315.1449	-4.4	$C_{15}H_{23}O_7^-$
303.1466	303.1449	-5.6	$C_{14}H_{23}O_7^-$
297.1354	297.1370	-3.3	$C_{15}H_{21}O_6^-$
285.1347	285.1344	-1.1	$C_{14}H_{21}O_6^-$
275.1508	275.1500	-2.8	$C_{13}H_{23}O_6^-$
247.1193	247.1088	-2.5	$C_{11}H_{19}O_6^-$
229.1087	229.1081	-2.3	$C_{11}H_{17}O_5^-$
145.0626	145.0619	-5.1	$C_5H_9N_2O_3^-$
127.0513	127.0513	-0.3	$C_5H_7N_2O_2^-$
102.0581	102.0561	-19.7	$C_4H_8NO_2^-$
85.0330	85.0295	-41.2	$C_4H_5O_2^-$
73.0347	73.0295	-70.8	$C_3H_5O_2^-$

UPLC-HR(ESI⁺)-qTOF-MS/MS			
Obs. [*m/z*]	Calc. [*m/z*]	Error [ppm]	Ion Formula [M + H]⁺
607.3092	607.3073	-3.1	$C_{27}H_{47}N_2O_{13}^+$
359.1824	359.1813	-4.7	$C_{16}H_{27}N_2O_7^+$
341.1723	341.1707	-1.8	$C_{16}H_{25}N_2O_6^+$
316.1771	316.1755	-5.0	$C_{15}H_{26}NO_6^+$
299.1500	299.1489	-3.5	$C_{15}H_{23}O_6^+$
259.1294	259.1288	-2.0	$C_{11}H_{19}N_2O_5^+$
242.1031	242.1023	-3.3	$C_{11}H_{16}NO_5^+$
199.0969	199.0965	-2.1	$C_{10}H_{15}O_4^+$
147.0768	147.0764	-2.3	$C_5H_{11}N_2O_3^+$
129.0661	129.0659	-1.9	$C_5H_9N_2O_2^+$
113.0600	113.0597	-3.0	$C_6H_9O_2^+$
101.0600	101.0597	-3.0	$C_5H_9O_2^+$
95.0493	95.0491	-1.8	$C_6H_7O^+$

Figure 16. UPLC-HR(ESI⁻/⁺)-qTOF-MS/MS mass spectral data of ubas#1 [4'-UB-2'-(asc-ωC5)-asc-C5] (from *P. laevicollis*) in both negative and positive ion modes. Analysis of NMR data of ubas#1 isolated from the *exo*-metabolome of *P. laevicollis* confirmed its chemical identity (***supplementary file 1d – Figures 16-19***).

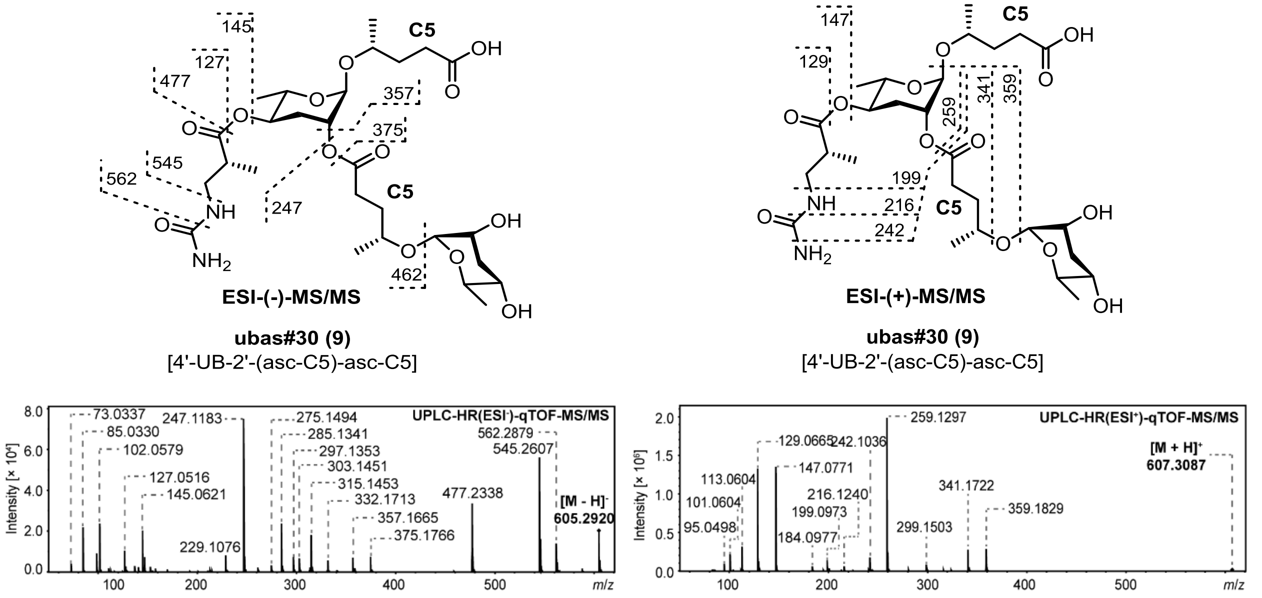

UPLC-HR(ESI$^-$)-qTOF-MS/MS			
Obs. [*m*/*z*]	Calc. [*m*/*z*]	Error [ppm]	Ion Formula [M − H]$^-$
605.2920	605.2927	1.2	$C_{27}H_{45}N_2O_{13}^-$
562.2879	562.2869	-1.8	$C_{26}H_{44}NO_{12}^-$
545.2607	545.2604	-0.6	$C_{26}H_{41}O_{12}^-$
477.2338	477.2341	0.7	$C_{22}H_{37}O_{11}^-$
375.1766	375.1773	2.0	$C_{16}H_{27}N_2O_8^-$
357.1665	357.1667	0.6	$C_{16}H_{25}N_2O_7^-$
332.1713	332.1715	0.6	$C_{15}H_{26}NO_7^-$
315.1453	315.1449	-1.3	$C_{15}H_{23}O_7^-$
303.1451	303.1449	-0.4	$C_{14}H_{23}O_7^-$
297.1353	297.1370	-3.1	$C_{15}H_{21}O_6^-$
285.1341	285.1344	-0.8	$C_{14}H_{21}O_6^-$
275.1494	275.1500	2.1	$C_{13}H_{23}O_6^-$
247.1183	247.1088	1.5	$C_{11}H_{19}O_6^-$
229.1076	229.1081	2.4	$C_{11}H_{17}O_5^-$
145.0621	145.0619	-1.9	$C_5H_9N_2O_3^-$
127.0516	127.0513	-2.6	$C_5H_7N_2O_2^-$
102.0579	102.0561	-18.2	$C_4H_8NO_2^-$
85.0330	85.0295	-40.6	$C_4H_5O_2^-$
73.0337	73.0295	-57.2	$C_3H_5O_2^-$

UPLC-HR(ESI$^+$)-qTOF-MS/MS			
Obs. [*m*/*z*]	Calc. [*m*/*z*]	Error [ppm]	Ion Formula [M + H]$^+$
607.3087	607.3073	-2.3	$C_{27}H_{47}N_2O_{13}^+$
359.1829	359.1813	-4.7	$C_{16}H_{27}N_2O_7^+$
341.1722	341.1707	-4.3	$C_{16}H_{25}N_2O_6^+$
299.1503	299.1489	-4.6	$C_{15}H_{23}O_6^+$
259.1297	259.1288	-3.1	$C_{11}H_{19}N_2O_5^+$
242.1036	242.1023	-5.5	$C_{11}H_{16}NO_5^+$
216.1240	216.1230	-4.3	$C_{10}H_{18}NO_4^+$
199.0973	199.0965	-4.3	$C_{10}H_{15}O_4^+$
184.0977	184.0968	-4.6	$C_{14}H_{14}NO_3^+$
147.0771	147.0764	-4.6	$C_5H_{11}N_2O_3^+$
129.0665	129.0659	-5.1	$C_5H_9N_2O_2^+$
113.0604	113.0597	-6.1	$C_6H_9O_2^+$
101.0604	101.0597	-6.9	$C_5H_9O_2^+$
95.0498	95.0491	-7.2	$C_6H_7O^+$

Figure 17. UPLC-HR(ESI$^{-/+}$)-qTOF-MS/MS mass spectral data of ubas#30 [4'-UB-2'-(asc-C5)-asc-C5, **9**] (from *P. maxplancki*) in both negative and positive ion modes.

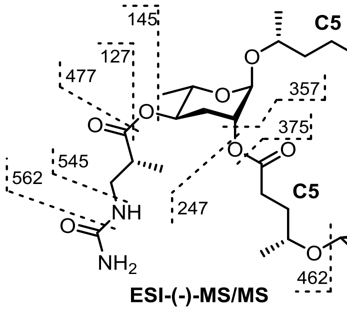

ubas#30 (9)
[4'-UB-2'-(asc-C5)-asc-C5]

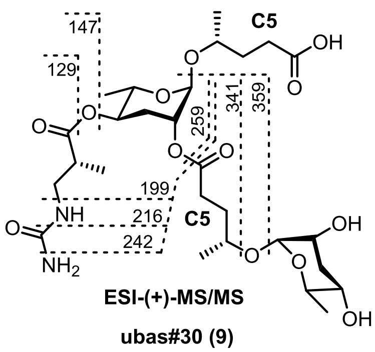

ubas#30 (9)
[4'-UB-2'-(asc-C5)-asc-C5]

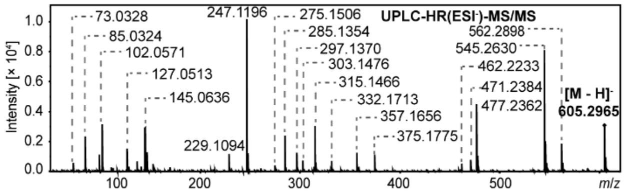

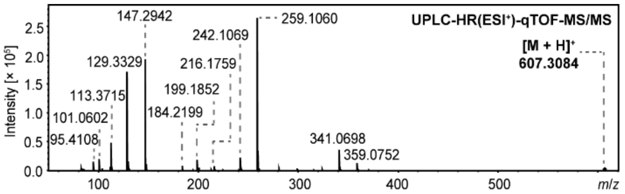

UPLC-HR(ESI⁻)-qTOF-MS/MS			
Obs. [*m/z*]	Calc. [*m/z*]	Error [ppm]	Ion Formula $[M-H]^-$
605.2954	605.2927	-4.5	$C_{27}H_{45}N_2O_{13}^-$
562.2898	562.2869	-5.1	$C_{26}H_{44}NO_{12}^-$
545.2630	545.2604	-4.9	$C_{26}H_{41}O_{12}^-$
477.2362	477.2341	-4.4	$C_{22}H_{37}O_{11}^-$
471.2384	471.2348	-7.6	$C_{22}H_{35}N_2O_9^-$
462.2233	462.2345	24.1	$C_{21}H_{36}NO_{10}^-$
375.1775	375.1773	-0.5	$C_{16}H_{27}N_2O_8^-$
357.1656	357.1667	3.0	$C_{16}H_{25}N_2O_7^-$
332.1713	332.1715	0.4	$C_{15}H_{26}NO_7^-$
315.1466	15.1449	-5.5	$C_{15}H_{23}O_7^-$
303.1476	303.1449	-8.8	$C_{14}H_{23}O_7^-$
297.1370	297.1370	-9.0	$C_{15}H_{21}O_6^-$
285.1354	285.1344	-3.8	$C_{14}H_{21}O_6^-$
275.1506	275.1500	-2.2	$C_{13}H_{23}O_6^-$
247.1196	247.1088	-3.4	$C_{11}H_{19}O_6^-$
229.1094	229.1081	-5.3	$C_{11}H_{17}O_5^-$
145.0636	145.0619	-	$C_5H_9N_2O_3^-$
127.0513	127.0513	0.0	$C_5H_7N_2O_2^-$
102.0571	102.0561	-	$C_4H_8NO_2^-$
85.0324	85.0295	-	$C_4H_5O_2^-$
73.0328	73.0295	-	$C_3H_5O_2^-$

UPLC-HR(ESI⁺)-qTOF-MS/MS			
Obs. [*m/z*]	Calc. [*m/z*]	Error [ppm]	Ion Formula $[M+H]^+$
607.3084	607.3073	-1.9	$C_{27}H_{47}N_2O_{13}^+$
359.1827	359.1813	-3.9	$C_{16}H_{27}N_2O_7^+$
341.1713	341.1707	-1.8	$C_{16}H_{25}N_2O_6^+$
259.1293	259.1288	-1.8	$C_{11}H_{19}N_2O_5^+$
242.1024	242.1023	-0.3	$C_{11}H_{16}NO_5^+$
216.1239	216.1230	-3.9	$C_{10}H_{18}NO_4^+$
199.0969	199.0965	-1.9	$C_{10}H_{15}O_4^+$
184.0968	184.0968	0.3	$C_9H_{14}NO_3^+$
147.0768	147.0764	-2.9	$C_5H_{11}N_2O_3^+$
129.0663	129.0659	-3.5	$C_5H_9N_2O_2^+$
113.0602	113.0597	-4.0	$C_6H_9O_2^+$
101.0605	101.0597	-	$C_5H_9O_2^+$
95.0497	95.0491	-	$C_6H_7O^+$

Figure 18. UPLC-HR(ESI$^{-/+}$)-qTOF-MS/MS mass spectral data of ubas#30 [4'-UB-2'-(asc-C5)-asc-C5, **9**] (from *P. fukushimae*) in both negative and positive ion modes. Peak at *m/z* 605.2954 was tentatively assigned to be ubas#30 [4'-UB-2'-(asc-C5)-asc-C5, **9**].

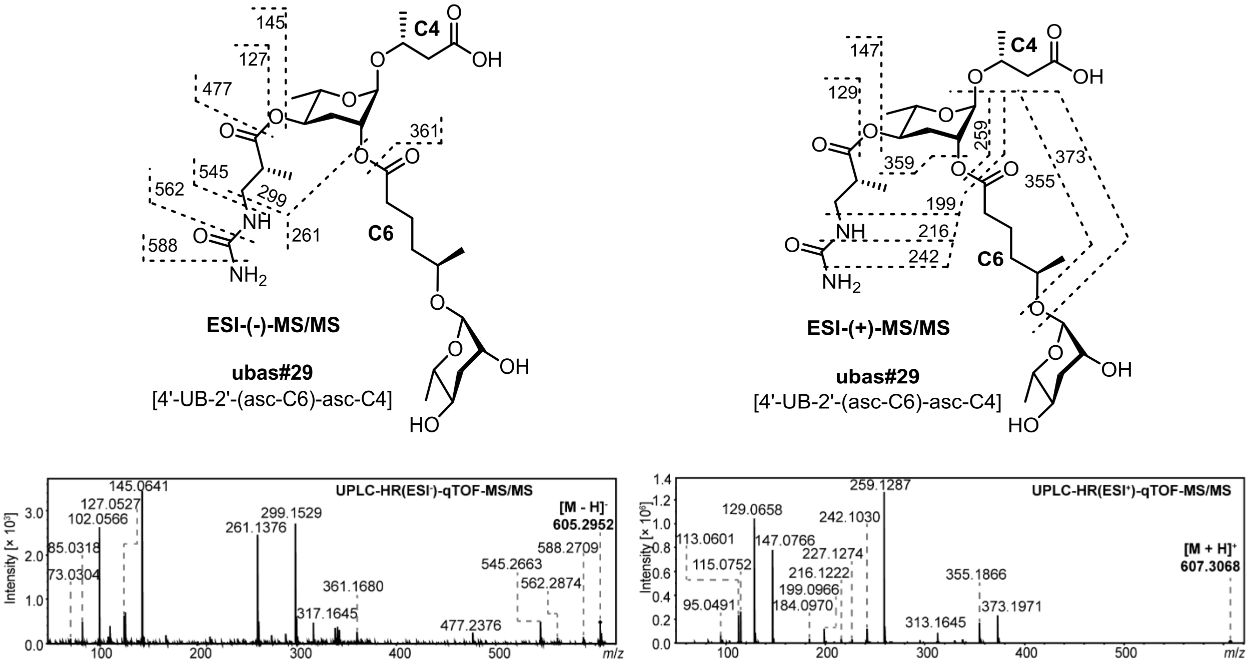

UPLC-HR(ESI⁻)-qTOF-MS/MS			
Obs. [*m*/*z*]	Calc. [*m*/*z*]	Error [ppm]	Ion Formula $[M-H]^-$
605.2952	605.2927	-4.1	$C_{27}H_{45}N_2O_{13}^-$
588.2709	588.2662	-8.1	$C_{27}H_{42}NO_{13}^-$
562.2874	562.2869	-0.9	$C_{26}H_{44}NO_{12}^-$
545.2663	545.2604	-10.9	$C_{26}H_{41}O_{12}^-$
477.2376	477.2341	-7.3	$C_{22}H_{37}O_{11}^-$
361.1680	361.1616	-17.6	$C_{15}H_{25}N_2O_8^-$
317.1645	317.1718	23.0	$C_{14}H_{25}N_2O_6^-$
299.1529	299.1500	-9.5	$C_{15}H_{23}O_6^-$
261.1376	261.1344	-	$C_{12}H_{21}O_6^-$
145.0641	145.0619	-	$C_5H_9N_2O_3^-$
127.0527	127.0513	-	$C_5H_7N_2O_2^-$
102.0566	102.0561	-	$C_4H_8NO_2^-$
85.0318	85.0295	-	$C_4H_5O_2^-$
73.0304	73.0295	-	$C_3H_5O_2^-$

UPLC-HR(ESI⁺)-qTOF-MS/MS			
Obs. [*m*/*z*]	Calc. [*m*/*z*]	Error [ppm]	Ion Formula $[M+H]^+$
607.3068	607.3073	0.8	$C_{27}H_{47}N_2O_{13}^+$
373.1971	373.1969	-0.5	$C_{17}H_{29}N_2O_7^+$
355.1866	355.1864	-0.8	$C_{17}H_{27}N_2O_6^+$
313.1645	313.1646	0.3	$C_{16}H_{25}O_6^+$
259.1287	259.1288	0.5	$C_{11}H_{19}N_2O_5^+$
242.1030	242.1023	-2.9	$C_{11}H_{16}NO_5^+$
227.1274	227.1278	1.8	$C_{12}H_{19}O_4^+$
216.1222	216.1230	3.7	$C_{10}H_{18}NO_4^+$
199.0966	199.0965	-0.4	$C_{10}H_{15}O_4^+$
184.0970	184.0968	-1.2	$C_9H_{14}NO_3^+$
147.0766	147.0764	-1.0	$C_5H_{11}N_2O_3^+$
129.0658	129.0659	0.2	$C_5H_9N_2O_2^+$
115.0752	115.0754	1.7	$C_6H_{11}O_2^+$
113.0601	113.0597	-3.4	$C_6H_9O_2^+$
95.0491	95.0491	0.4	$C_6H_7O^+$

Figure 19. UPLC-HR(ESI$^{-/+}$)-qTOF-MS/MS mass spectral data of ubas#29 [4'-UB-2'-(asc-C6)-asc-C4] in both negative and positive ion modes.

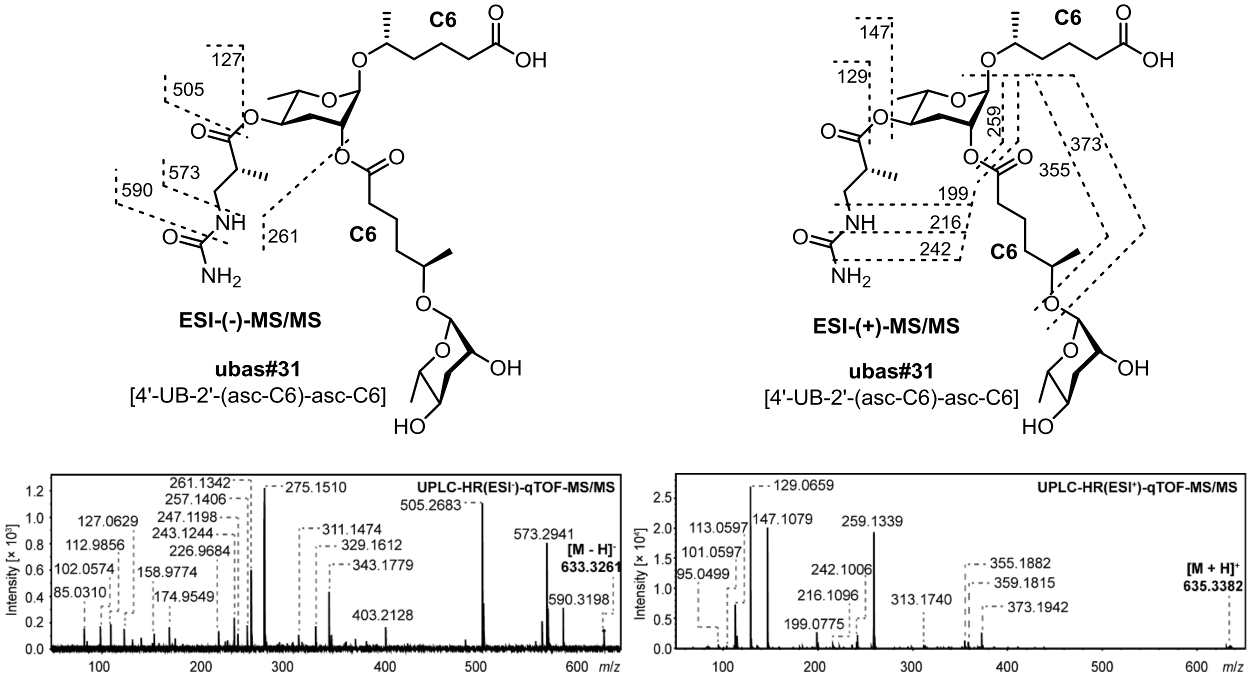

UPLC-HR(ESI$^-$)-qTOF-MS/MS			
Obs. [*m/z*]	Calc. [*m/z*]	Error [ppm]	Ion Formula [M − H]$^-$
633.3261	633.3240	-3.4	$C_{29}H_{49}N_2O_{13}^-$
590.3198	590.3182	-2.7	$C_{28}H_{48}NO_{12}^-$
573.2941	573.2917	-4.4	$C_{28}H_{45}O_{12}^-$
505.2683	505.2654	-5.7	$C_{24}H_{41}O_{11}^-$
403.2128	403.2126	-0.4	$C_{23}H_{31}O_6^-$
343.1779	343.1762	-4.8	$C_{27}H_{27}O_7^-$
329.1612	329.1606	-1.9	$C_{16}H_{25}O_7^-$
311.1474	311.1500	9.4	$C_{16}H_{23}O_6^-$
275.1510	275.1500	-3.5	$C_{13}H_{23}O_6^-$
261.1342	261.1344	0.6	$C_{12}H_{21}O_6^-$
257.1406	257.1394	-4.3	$C_{13}H_{21}O_5^-$
247.1198	247.1187	-4.4	$C_{11}H_{19}O_6^-$
243.1244	243.1238	-2.4	$C_{12}H_{19}O_5^-$
127.0629	127.0513	-	$C_5H_7N_2O_2^-$
102.0574	102.0561	-	$C_4H_8NO_2^-$
85.0310	85.0295	-	$C_4H_5O_2^-$

UPLC-HR(ESI$^+$)-qTOF-MS/MS			
Obs. [*m/z*]	Calc. [*m/z*]	Error [ppm]	Ion Formula [M + H]$^+$
635.3382	635.3386	0.4	$C_{29}H_{51}N_2O_{13}^+$
373.1942	373.1969	7.4	$C_{17}H_{29}N_2O_7^+$
359.1815	359.1813	-0.5	$C_{16}H_{27}N_2O_7^+$
355.1882	355.1864	-5.1	$C_{17}H_{27}N_2O_6^+$
313.1740	316.1758	-5.9	$C_{15}H_{25}NO_5^+$
259.1339	259.1288	-	$C_{11}H_{19}N_2O_5^+$
242.1006	242.1023	7.2	$C_{11}H_{16}NO_5^+$
199.0775	199.0965	-	$C_{10}H_{15}O_4^+$
147.1079	147.0764	-	$C_5H_{11}N_2O_3^+$
129.0659		-	$C_5H_9N_2O_2^+$
113.0597		-	$C_6H_9O_2^+$

Figure 20. UPLC-HR(ESI$^{-/+}$)-qTOF-MS/MS mass spectral data of ubas#31 [4'-UB-2'-(asc-C6)-asc-C6] in both negative and positive ion modes.

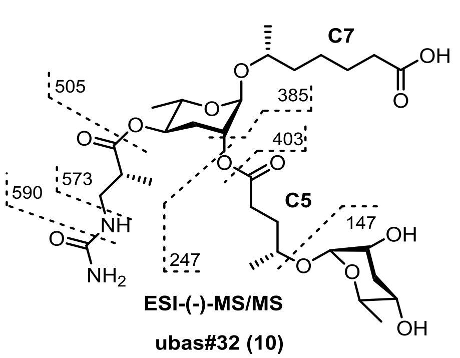

ubas#32 (10)
[4'-UB-2'-(asc-C5)-asc-C7]

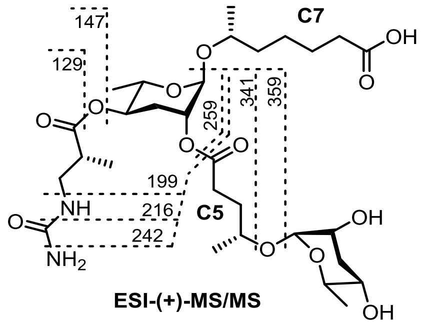

ubas#32 (10)
[4'-UB-2'-(asc-C5)-asc-C7]

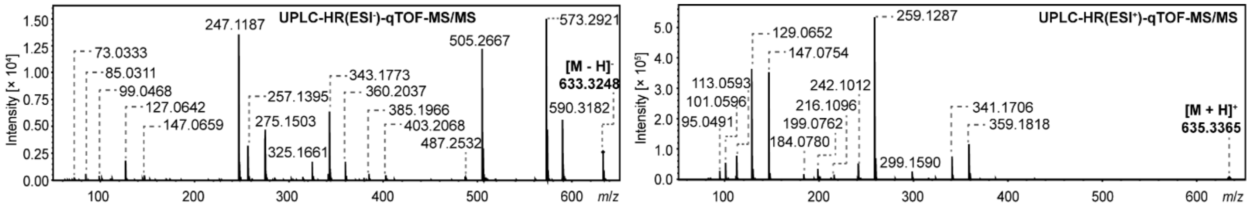

UPLC-HR(ESI$^-$)-qTOF-MS/MS			
Obs. [*m/z*]	Calc. [*m/z*]	Error [ppm]	Ion Formula [M − H]$^-$
633.3248	633.3240	-1.3	$C_{29}H_{49}N_2O_{13}^-$
590.3182	590.3182	0.0	$C_{28}H_{48}NO_{12}^-$
573.2921	573.2917	-0.8	$C_{28}H_{45}O_{12}^-$
505.2667	505.2654	-2.6	$C_{24}H_{41}O_{11}^-$
487.2532	487.2549	3.5	$C_{24}H_{39}O_{10}^-$
403.2068	403.2086	4.4	$C_{18}H_{31}N_2O_8^-$
385.1966	385.1980	3.8	$C_{18}H_{29}N_2O_7^-$
360.2037	360.2028	-2.6	$C_{17}H_{30}NO_7^-$
343.1773	343.1762	-3.2	$C_{27}H_{27}O_7^-$
325.1661	325.1657	-1.3	$C_{17}H_{25}O_6^-$
275.1503	275.1500	-1.2	$C_{13}H_{23}O_6^-$
257.1395	257.1394	-0.4	$C_{13}H_{21}O_5^-$
247.1187	247.1187	0.2	$C_{11}H_{19}O_6^-$
147.0659	147.0663	2.4	$C_6H_{11}O_4^-$
99.0468	99.0452	-	$C_5H_7O_2^-$
85.0311	85.0295	-	$C_4H_5O_2^-$
73.0333	73.0295	-	$C_3H_5O_2^-$

UPLC-HR(ESI$^+$)-qTOF-MS/MS			
Obs. [*m/z*]	Calc. [*m/z*]	Error [ppm]	Ion Formula [M + H]$^+$
635.3365	635.3386	2.1	$C_{29}H_{51}N_2O_{13}^+$
359.1818	359.1813	-1.6	$C_{16}H_{27}N_2O_7^+$
341.1706	341.1707	0.1	$C_{16}H_{27}N_2O_6^+$
299.1590	299.1601	3.9	$C_{14}H_{23}N_2O_5^+$
259.1287	259.1288	0.2	$C_{11}H_{19}N_2O_5^+$
242.1012	242.1023	4.7	$C_{11}H_{16}NO_5^+$
199.0962	199.0965	0.3	$C_{10}H_{15}O_4^+$
147.0754	147.0764	1.1	$C_5H_{11}N_2O_3^+$
129.0652	129.0659	0.5	$C_5H_9N_2O_2^+$
113.0593	113.0597	0.4	$C_6H_9O_2^+$
101.0596	101.0597	0.2	$C_5H_9O_2^+$
95.0491	95.0491	0.0	$C_6H_7O^+$

Figure 21. UPLC-HR(ESI$^{-/+}$)-qTOF-MS/MS mass spectral data of ubas#32 [4'-UB-2'-(asc-C5)-asc-C7, **10**] in both negative and positive ion modes. Analysis of the *dqf*-COSY spectrum of ubas#32 [4'-UB-2'-(asc-C5)-asc-C7, **10**] (800 MHz, CD_3OD) enriched from the *exo*-metabolome of *P. maxplancki* confirmed the presence of one 2'- and 4'-substituted ascarylose sugar, an unsubstituted ascarylose moiety, one (ω-1)-hydroxylated C5 side chain and one (ω-1)-hydroxylated C7 side chain in ubas#32 (***supplementary file 1d – Figure 20***).

supplementary file 2d: MS/MS spectral data of UPAS chemicals Pages

Figure 1. LC-HR(ESI$^{-/+}$)-MS/MS spectral data of upas#5 [4'-UP-asc-C4]. S48

Figure 2. LC-HR(ESI$^{-/+}$)-MS/MS spectral data of upas#3 [4'-UP-asc-C5]. S49

Figure 3. LC-HR(ESI$^{-/+}$)-MS/MS spectral data of upas#4 [4'-UP-asc-C7]. S50

Figure 4. LC-HR(ESI$^{-/+}$)-MS/MS spectral data of upas#34 [4'-UP-2'-(asc-C4)-asc-C4, **14**]. S51

Figure 5. LC-HR(ESI$^{-/+}$)-MS/MS spectral data of upas#28 [4'-UP-2'-(asc-C5)-asc-C4, **15**]. S52

Figure 6. LC-HR(ESI$^{-/+}$)-MS/MS spectral data of upas#30 [4'-UP-2'-(asc-C5)-asc-C5]. S53

Figure 7. LC-HR(ESI$^{-/+}$)-MS/MS spectral data of upas#32 [4'-UP-2'-(asc-C5)-asc-C7]. S54

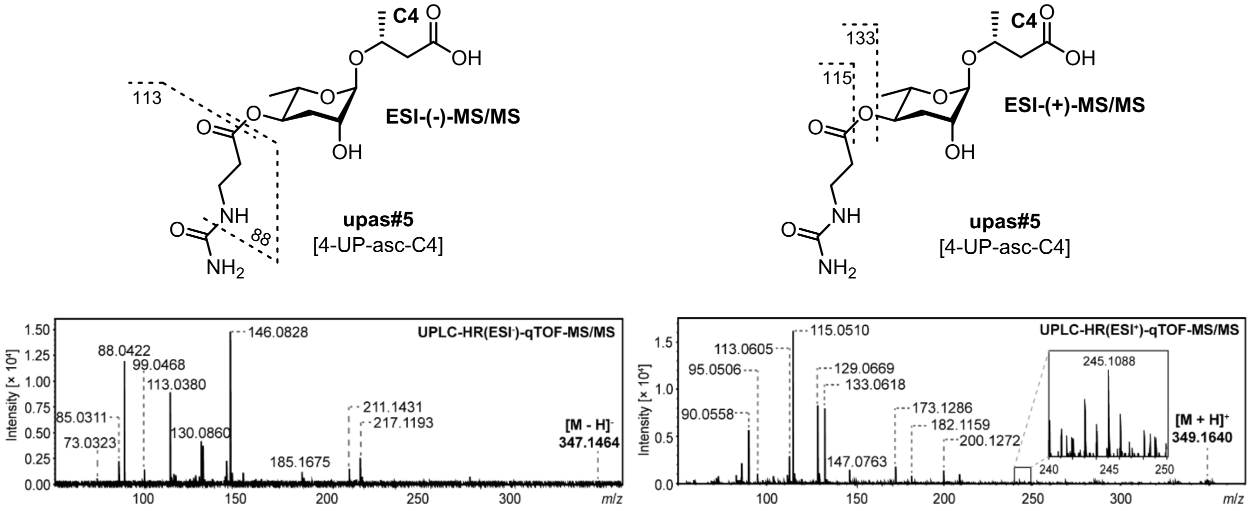

UPLC-HR(ESI⁻)-qTOF-MS/MS			
Obs. [*m/z*]	Calc. [*m/z*]	Error [ppm]	Ion Formula [M − H]⁻
347.1464	347.1460	-1.1	$C_{14}H_{23}N_2O_8^-$
217.1193	217.1194	0.5	$C_9H_{17}N_2O_4^-$
211.1431	211.1452	-	$C_{11}H_{19}N_2O_2^-$
185.1675	185.1659	-8.2	$C_{10}H_{21}N_2O^-$
146.0828	146.0823	-3.5	$C_6H_{12}NO_3^-$
130.0860	130.0874	-	$C_6H_{12}NO_2^-$
113.0380	113.0357	-	$C_4H_5N_2O_2^-$
99.0468	99.0452	-	$C_5H_7O_2^-$
88.0422	88.0404	-	$C_3H_6NO_2^-$
85.0311	85.0295	-	$C_4H_5O_2^-$

UPLC-HR(ESI⁺)-qTOF-MS/MS			
Obs. [*m/z*]	Calc. [*m/z*]	Error [ppm]	Ion Formula [M + H]⁺
349.1640	349.1605	-9.8	$C_{14}H_{25}N_2O_8^+$
245.1088	245.1132	-	$C_{10}H_{17}N_2O_5^+$
200.1272	200.1281	4.5	$C_{10}H_{18}NO_3^+$
182.1159	182.1176	8.9	$C_{10}H_{16}NO_2^+$
173.1286	173.1285	-0.9	$C_8H_{17}N_2O_2^+$
147.0763	147.0764	0.6	$C_5H_{11}N_2O_3^+$
133.0618	133.0608	-	$C_4H_9N_2O_3^+$
129.0669	129.0659	-	$C_5H_9N_2O_2^+$
115.0510	115.0502	-	$C_4H_7N_2O_2^+$
113.0605	113.0597	-	$C_6H_9O_2^+$
95.0506	95.0491	-	$C_6H_7O^+$
90.0558	90.0550	-	$C_3H_8NO_2^+$

Figure 1. UPLC-HR(ESI⁻/⁺)-qTOF-MS/MS mass spectral data of upas#5 [4'-UP-asc-C4] in both negative and positive ion modes.

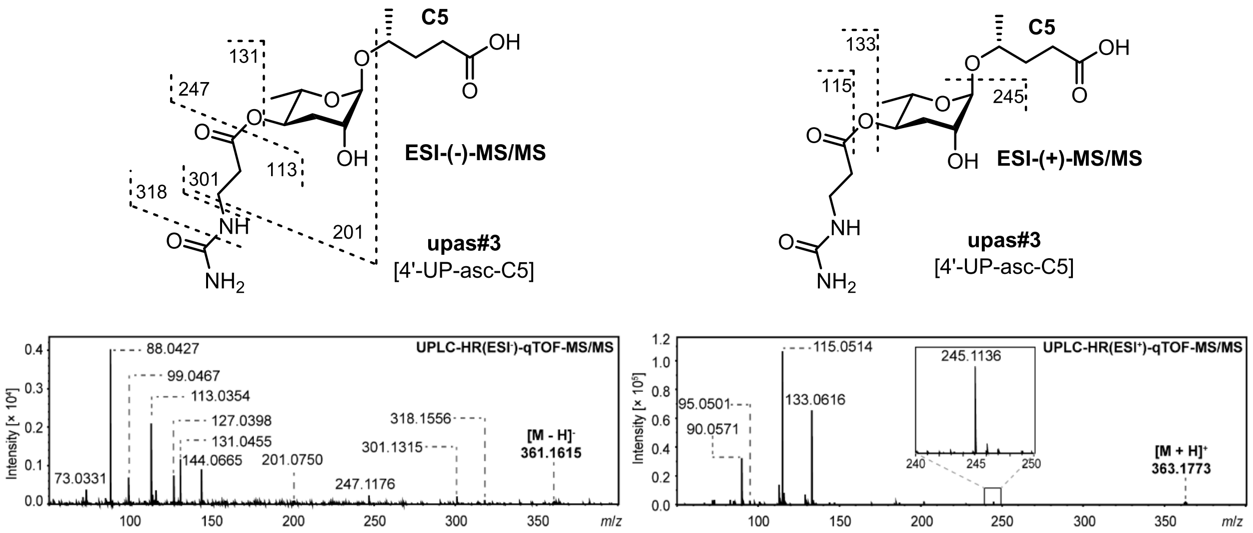

UPLC-HR(ESI⁻)-qTOF-MS/MS			
Obs. [*m*/*z*]	Calc. [*m*/*z*]	Error [ppm]	Ion Formula $[M - H]^-$
361.1615	361.1616	0.4	$C_{15}H_{25}N_2O_8^-$
318.1556	318.1558	0.8	$C_{14}H_{24}NO_7^-$
301.1315	301.1293	-7.5	$C_{14}H_{21}O_7^-$
247.1176	247.1187	4.6	$C_{11}H_{19}O_6^-$
201.0750	201.0768	9.0	$C_9H_{13}O_5^-$
144.0665	144.0666	0.8	$C_6H_{10}N_2O_3^-$
131.0455	131.0462	5.3	$C_4H_7N_2O_3^-$
127.0398	127.0401	1.9	$C_6H_7O_3^-$
113.0354	113.0357	2.0	$C_4H_5N_2O_2^-$
99.0467	99.0452	-	$C_5H_7O_2^-$
88.0427	88.0404	-	$C_3H_6NO_2^-$
73.0331	73.0295	-	$C_3H_5O_2^-$

UPLC-HR(ESI⁺)-qTOF-MS/MS			
Obs. [*m*/*z*]	Calc. [*m*/*z*]	Error [ppm]	Ion Formula $[M + H]^+$
363.1773	363.1762	-3.1	$C_{15}H_{27}N_2O_8^+$
245.1136	245.1132	-1.7	$C_{10}H_{17}N_2O_5^+$
133.0616	133.0608	-6.4	$C_4H_9N_2O_3^+$
115.0514	115.0502	-	$C_4H_7N_2O_2^+$
95.0501	95.0491	-	$C_6H_7O^+$

Figure 2. UPLC-HR(ESI$^{-/+}$)-qTOF-MS/MS mass spectral data of upas#3 [4'-UP-asc-C5] in both negative and positive ion modes.

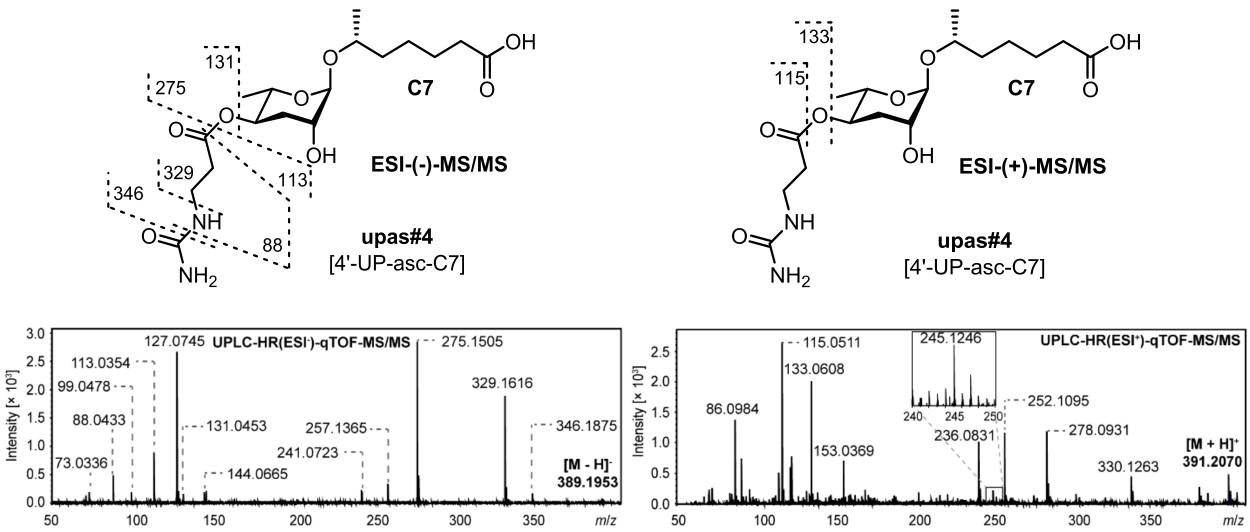

UPLC-HR(ESI⁻)-qTOF-MS/MS			
Obs. [*m/z*]	Calc. [*m/z*]	Error [ppm]	Ion Formula $[M-H]^-$
389.1935	389.1929	-1.4	$C_{17}H_{29}N_2O_8^-$
346.1875	346.1871	-1.0	$C_{16}H_{28}NO_7^-$
329.1616	329.1606	-3.2	$C_{16}H_{25}O_7^-$
275.1505	275.1500	-1.9	$C_{13}H_{23}O_6^-$
257.1365	257.1354	-4.3	$C_8H_{21}N_2O_7^-$
241.0723	241.0718	-2.1	$C_{11}H_{13}O_6^-$
144.0665	144.0666	0.9	$C_6H_{10}NO_3^-$
131.0453	131.0462	7.0	$C_4H_7N_2O_3^-$
127.0745	127.0765	-	$C_7H_{11}O_2^-$
113.0354	113.0357	2.0	$C_4H_5N_2O_2^-$
99.0478	99.0452	-	$C_5H_7O_2^-$
88.0433	88.0404	-	$C_3H_6NO_2^-$
73.0336	73.0295	-	$C_3H_5O_2^-$

UPLC-HR(ESI⁺)-qTOF-MS/MS			
Obs. [*m/z*]	Calc. [*m/z*]	Error [ppm]	Ion Formula $[M+H]^+$
391.2070	391.2075	1.2	$C_{17}H_{31}N_2O_8^+$
278.0931	278.0870	-	$C_{10}H_{16}NO_8^+$
252.1095	252.1078	-6.7	$C_9H_{18}NO_7^+$
245.1246	245.1132	-	$C_{10}H_{17}N_2O_5^+$
236.0831	236.0765	-	$C_8H_{14}NO_7^+$
133.0608	133.0608	0.0	$C_4H_9N_2O_3^+$
115.0511	115.0502	-	$C_4H_7N_2O_2^+$

Figure 3. UPLC-HR(ESI$^{-/+}$)-qTOF-MS/MS mass spectral data of upas#4 [4'-UP-asc-C7] in both negative and positive ion modes.

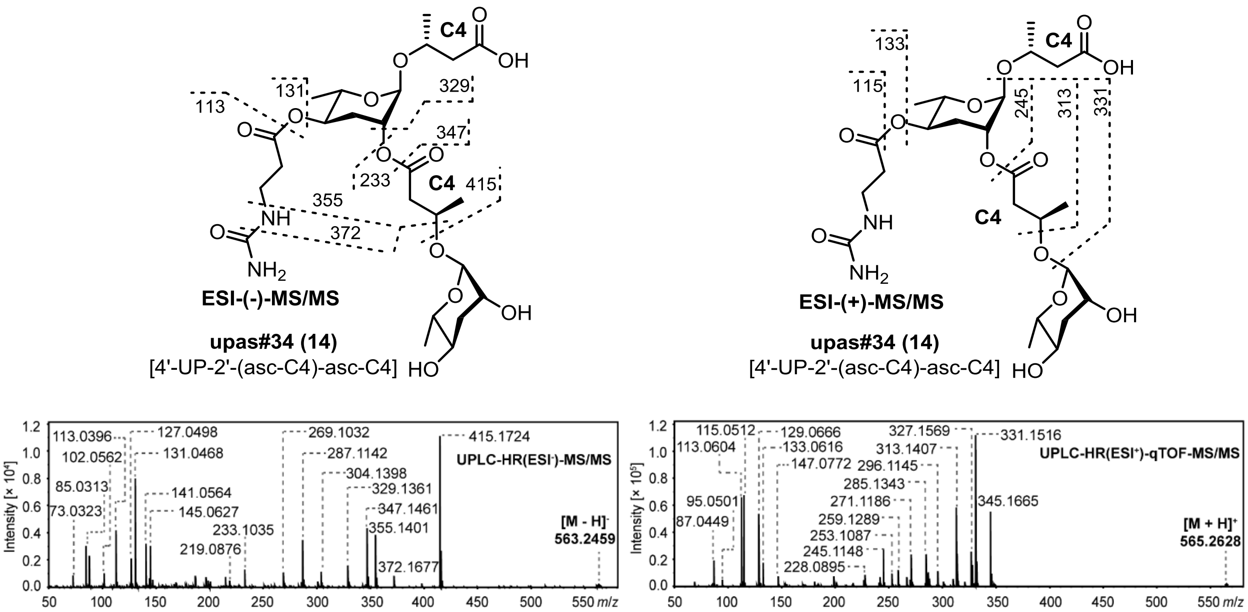

UPLC-HR(ESI$^-$)-qTOF-MS/MS			
Obs. [*m*/*z*]	Calc. [*m*/*z*]	Error [ppm]	Ion Formula [M − H]$^-$
563.2459	563.2458	-0.3	$C_{24}H_{39}N_2O_{13}^-$
415.1724	415.1722	-0.5	$C_{18}H_{27}N_2O_9^-$
372.1677	372.1664	-3.4	$C_{17}H_{26}NO_8^-$
355.1401	355.1398	-0.7	$C_{17}H_{23}O_8^-$
347.1461	347.1460	-0.4	$C_{14}H_{23}N_2O_8^-$
329.1361	329.1354	-1.9	$C_{14}H_{21}N_2O_7^-$
304.1398	304.1402	1.1	$C_{13}H_{22}NO_7^-$
287.1142	287.1136	-2.1	$C_{13}H_{19}O_7^-$
269.1032	269.1031	-0.5	$C_{13}H_{17}O_6^-$
233.1035	233.1031	-1.8	$C_{10}H_{17}O_6^-$
219.0876	219.0874	-0.7	$C_9H_{15}O_6^-$
145.0627	145.0619	-5.9	$C_5H_9N_2O_3^-$
141.0564	141.0557	-5.0	$C_5H_9O_3^-$
131.0468	131.0462	-4.7	$C_4H_7N_2O_3^-$
127.0498	127.0513	-	$C_5H_7N_2O_2^-$
113.0392	113.0357	-	$C_4H_5N_2O_2^-$
102.0562	102.0561	-1.7	$C_4H_8NO_2^-$
88.0413	88.0404	-	$C_3H_6NO_2^-$
85.0313	85.0295	-	$C_4H_5O_2^-$
73.0323	73.0295	-	$C_3H_5O_2^-$

UPLC-HR(ESI$^+$)-qTOF-MS/MS			
Obs. [*m*/*z*]	Calc. [*m*/*z*]	Error [ppm]	Ion Formula [M + H]$^+$
565.2628	565.2603	-4.3	$C_{24}H_{41}N_2O_{13}^+$
345.1665	345.1656	-2.4	$C_{15}H_{25}N_2O_7^+$
331.1516	331.1500	-4.8	$C_{14}H_{23}N_2O_7^+$
327.1569	327.1551	-5.5	$C_{15}H_{23}N_2O_6^+$
313.1407	313.1394	-4.1	$C_{14}H_{21}N_2O_6^+$
296.1145	296.1129	-5.6	$C_{14}H_{18}NO_6^+$
285.1343	285.1333	-3.5	$C_{14}H_{21}O_6^+$
271.1186	271.1176	-3.8	$C_{13}H_{19}O_6^+$
259.1289	259.1288	-0.3	$C_{11}H_{19}N_2O_5^+$
253.1087	253.1071	-6.6	$C_{13}H_{17}O_5^+$
245.1148	245.1132	-6.4	$C_{10}H_{17}N_2O_5^+$
228.0895	228.0866	-	$C_{10}H_{14}NO_5^+$
217.1083	217.1071	-5.9	$C_{10}H_{17}O_5^+$
199.0985	199.0965	-	$C_{10}H_{15}O_4^+$
181.0870	181.0859	-6.1	$C_{10}H_{13}O_3^+$
147.0772	147.0764	-5.1	$C_5H_{11}N_2O_3^+$
133.0616	133.0608	-6.0	$C_4H_9N_2O_3^+$
115.0512	115.0502	-	$C_4H_7N_2O_2^+$
113.0604	113.0597	-	$C_6H_9O_2^+$
95.0501	95.0491	-	$C_6H_7O^+$
87.0449	87.0441	-	$C_4H_7O_2^+$

Figure 4. UPLC-HR(ESI$^{-/+}$)-qTOF-MS/MS mass spectral data of upas#34 [4'-UP-2'-(asc-C4)-asc-C4, **14**] in both negative and positive ion modes. Observation of the fragment ion signal for $C_4H_7N_2O_3^-$ (*m/z* 131.0468 [M - H]$^-$) indicated the presence of a ureidopropionic acid group in upas#34 [4'-UP-2'-(asc-C4)-asc-C4, **14**].

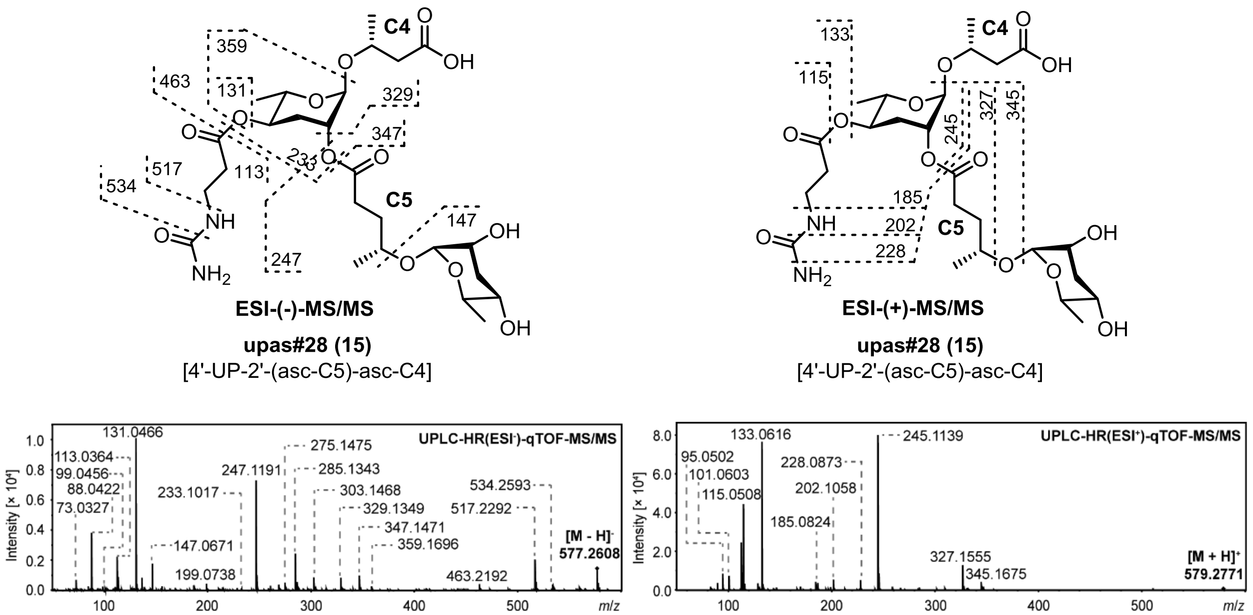

UPLC-HR(ESI⁻)-qTOF-MS/MS			
Obs. [*m/z*]	Calc. [*m/z*]	Error [ppm]	Ion Formula [M − H]⁻
577.2608	577.2596	-2.1	$C_{25}H_{41}N_2O_{13}^-$
534.2593	534.2556	-6.9	$C_{24}H_{40}NO_{12}^-$
517.2292	517.2291	-0.2	$C_{24}H_{37}O_{12}^-$
463.2192	463.2185	-1.4	$C_{21}H_{35}O_{11}^-$
359.1696	359.1711	4.2	$C_{17}H_{27}O_8^-$
347.1471	347.1460	-3.2	$C_{14}H_{23}N_2O_8^-$
329.1349	329.1354	-1.5	$C_{14}H_{21}N_2O_7^-$
303.1468	303.1449	-6.3	$C_{14}H_{23}O_7^-$
285.1343	285.1344	0.1	$C_{14}H_{21}O_6^-$
275.1475	275.1500	9.3	$C_{13}H_{23}O_6^-$
247.1191	247.1187	-1.5	$C_{11}H_{19}O_6^-$
233.1017	233.1031	5.9	$C_{10}H_{17}O_6^-$
199.0738	199.0724	-7.0	$C_8H_{11}N_2O_4^-$
147.0671	147.0663	-5.8	$C_6H_{11}O_4^-$
131.0466	131.0462	-3.1	$C_4H_7N_2O_3^-$
113.0364	113.0357	-6.4	$C_4H_5N_2O_2^-$
99.0456	99.0452	-4.8	$C_5H_7O_2^-$
88.0422	88.0404	-	$C_3H_6NO_2^-$
73.0327	73.0295	-	$C_3H_5O_2^-$

UPLC-HR(ESI⁺)-qTOF-MS/MS			
Obs. [*m/z*]	Calc. [*m/z*]	Error [ppm]	Ion Formula [M + H]⁺
579.2771	579.2760	-2.0	$C_{25}H_{43}N_2O_{13}^+$
345.1675	345.1656	-5.4	$C_{15}H_{25}N_2O_7^+$
327.1555	327.1551	-1.2	$C_{15}H_{23}N_2O_6^+$
245.1139	245.1132	-3.0	$C_{10}H_{17}N_2O_5^+$
228.0873	228.0866	-2.7	$C_{10}H_{14}NO_5^+$
202.1058	202.1074	-7.6	$C_9H_{16}NO_4^+$
185.0824	185.0808	-8.6	$C_9H_{13}O_4^+$
133.0616	133.0608	-5.9	$C_4H_9N_2O_3^+$
115.0508	115.0502	-5.5	$C_4H_7N_2O_2^+$
101.0603	101.0597	-5.9	$C_5H_9O_2^+$
95.0502	95.0491	-	$C_6H_7O^+$

Figure 5. UPLC-HR(ESI$^{-/+}$)-qTOF-MS/MS mass spectral data of upas#28 [4'-UP-2'-(asc-C5)-asc-C4, **15**] in both negative and positive ion modes. Observation of the fragment ion signal for $C_4H_7N_2O_3^-$ (*m/z* 131.0466 [M - H]⁻) indicated the presence of a ureidopropionic acid group in upas#28 [4'-UP-2'-(asc-C5)-asc-C4, **15**]. Observation of two other ion signals of $C_{15}H_{23}N_2O_6^-$ (*m/z* 327.1555 [M + H]⁺) and $C_{15}H_{25}N_2O_7^-$ (*m/z* 345.1675 [M + H]⁺) indicated that the first ascaroside in upas#28 [4'-UP-2'-(asc-C5)-asc-C4, **15**] should carry a C5 side chain.

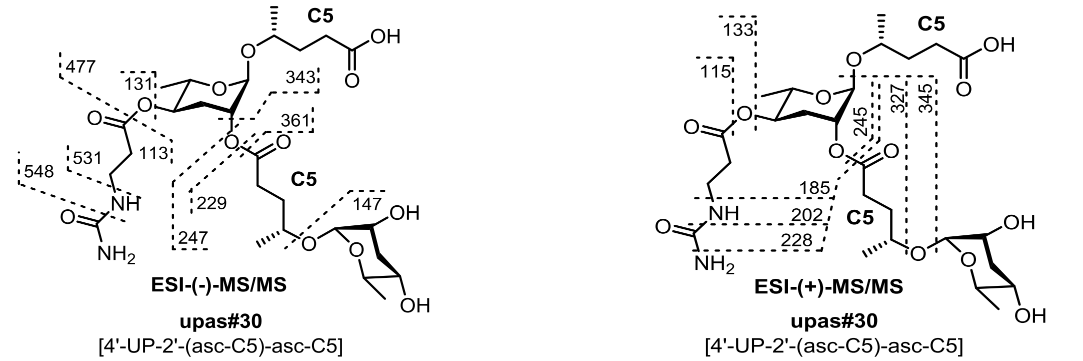

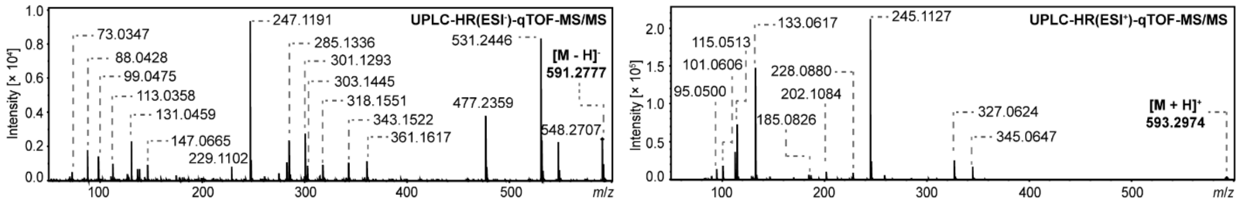

UPLC-HR(ESI$^-$)-qTOF-MS/MS			
Obs. [*m/z*]	Calc. [*m/z*]	Error [ppm]	Ion Formula [M − H]$^-$
591.2777	591.2771	-1.1	$C_{26}H_{43}N_2O_{13}^-$
548.2707	548.2712	1.0	$C_{25}H_{42}NO_{12}^-$
531.2446	531.2447	0.3	$C_{25}H_{39}O_{12}^-$
477.2359	477.2341	-3.7	$C_{22}H_{37}O_{11}^-$
361.1617	361.1616	-0.3	$C_{15}H_{25}N_2O_8^-$
343.1522	343.1511	-3.4	$C_{15}H_{23}N_2O_7^-$
318.1551	381.1558	2.3	$C_{14}H_{24}NO_7^-$
303.1445	303.1449	1.4	$C_{14}H_{23}O_7^-$
301.1293	301.1293	-0.2	$C_{14}H_{21}O_7^-$
285.1336	285.1344	0.1	$C_{14}H_{21}O_6^-$
247.1191	247.1187	-1.7	$C_{11}H_{19}O_6^-$
229.1102	229.1081	-	$C_{11}H_{17}O_5^-$
147.0665	147.0663	-1.4	$C_6H_{11}O_4^-$
131.0459	131.0462	2.1	$C_4H_7N_2O_3^-$
113.0358	113.0357	-1.7	$C_4H_5N_2O_2^-$
99.0475	99.0452	-	$C_5H_7O_2^-$
88.0428	88.0404	-	$C_3H_6NO_2^-$
73.0347	73.0295	-	$C_3H_5O_2^-$

UPLC-HR(ESI$^+$)-qTOF-MS/MS			
Obs. [*m/z*]	Calc. [*m/z*]	Error [ppm]	Ion Formula [M + H]$^+$
593.2974	593.2916	-5.2	$C_{26}H_{45}N_2O_{13}^+$
345.0647	345.1656	3.4	$C_{15}H_{25}N_2O_7^+$
327.0624	327.1551	-3.8	$C_{15}H_{23}N_2O_6^+$
245.1127	245.1132	2.0	$C_{10}H_{17}N_2O_5^+$
228.0880	228.0866	-5.9	$C_{10}H_{14}NO_5^+$
202.1084	202.1074	-5.3	$C_9H_{16}NO_4^+$
185.0826	185.0808	-	$C_9H_{13}O_4^+$
133.0617	133.0608	-7.2	$C_4H_9N_2O_3^+$
115.0513	115.0502	-	$C_4H_7N_2O_2^+$
101.0606	101.0597	-	$C_5H_9O_2^+$
95.0500	95.0491	-	$C_6H_7O^+$

Figure 6. UPLC-HR(ESI$^{-/+}$)-qTOF-MS/MS mass spectral data of upas#30 [4'-UP-2'-(asc-C5)-asc-C5] in both negative and positive ion modes**.**

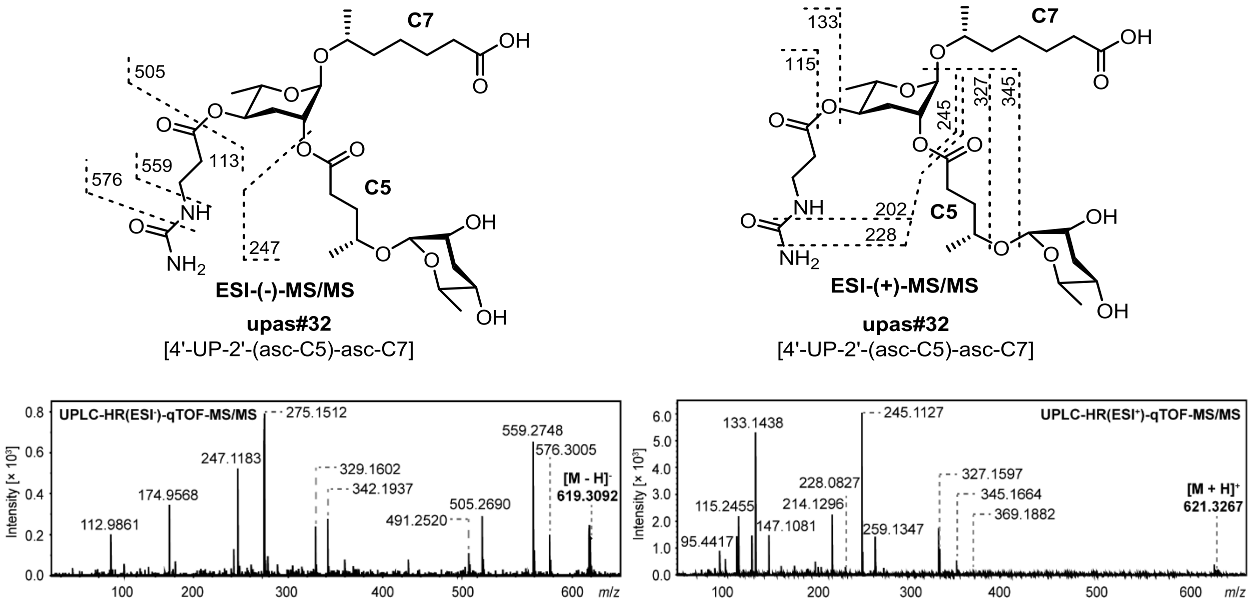

UPLC-HR(ESI⁻)-qTOF-MS/MS			
Obs. [*m*/*z*]	Calc. [*m*/*z*]	Error [ppm]	Ion Formula [M − H]$^-$
619.3092	619.3084	-1.4	$C_{28}H_{47}N_2O_{13}^-$
576.3005	576.3025	-3.6	$C_{27}H_{46}NO_{12}^-$
559.2748	559.2760	2.1	$C_{27}H_{43}O_{12}^-$
505.2690	505.2654	-7.1	$C_{24}H_{41}O_{11}^-$
491.2520	491.2498	-4.5	$C_{23}H_{39}O_{11}^-$
342.1937	342.1922	-4.5	$C_{27}H_{28}NO_6^-$
329.1602	329.1606	1.1	$C_{16}H_{25}O_7^-$
275.1512	275.1500	-4.3	$C_{13}H_{23}O_6^-$
247.1183	247.1187	1.8	$C_{11}H_{19}O_6^-$
112.9861	112.9880	-	$C_4HO_4^-$

UPLC-HR(ESI⁺)-qTOF-MS/MS			
Obs. [*m*/*z*]	Calc. [*m*/*z*]	Error [ppm]	Ion Formula [M + H]$^+$
621.3267	621.3229	-6.1	$C_{28}H_{49}N_2O_{13}^+$
369.1882	369.1908	-3.9	$C_{14}H_{29}N_2O_9^+$
345.1664	345.1656	-2.2	$C_{15}H_{25}N_2O_7^+$
259.1347	259.1288	-	$C_{11}H_{19}N_2O_5^+$
245.1127	245.1132	1.9	$C_{10}H_{17}N_2O_5^+$
228.0827	228.0866	-	$C_{10}H_{14}NO_5^+$
133.1438		-	$C_4H_9N_2O_3^+$
115.2455		-	$C_4H_7N_2O_2^+$

Figure 7. UPLC-HR(ESI$^{-/+}$)-qTOF-MS/MS mass spectral data of upas#32 [4'-UP-2'-(asc-C5)-asc-C7] in both negative and positive ion modes.

References

Bose N, Ogawa A, von Reuss SH, Yim JJ, Ragsdale EJ, Sommer RJ, Schroeder FC. 2012. Complex small-molecule architectures regulate phenotypic plasticity in a nematode. *Angewandte Chemie International Edition* **52**:12438-12443. DOI: https://doi.org/ 10.1002/anie.201206797

Falcke JM, Bose N, Artyukhin AB, Rödelsperger C, Markov GV, Yim JJ, Grimm D, Claassen MH, Panda O, Baccile JA, Zhang Y, Le HH, Jolic D, Schroeder FC, Sommer RJ. 2018. Linking genomic and metabolomic natural variation uncovers nematode pheromone biosynthesis. *Cell Chemical Biology* **25**:1-10. DOI: https://doi.org/10.1016/j.chembiol.2018.04.004

von Reuss SH, Bose N, Srinivasan J, Yim JJ, Judkins JC, Sternberg PW, Schroeder FC. 2012. Comparative metabolomics reveals biogenesis of ascarosides, a modular library of small-molecule signals in *C. elegans*. *Journal of the American Chemical Society* **134**:1817-1824. DOI: https://doi.org/10.1021/ja210202y
