# Supplementary material for: Convergent evolution of small molecule pheromones in Pristionchus nematodes
Source: eLife. 2020 Apr 27;9:e55687. doi: 10.7554/eLife.55687 (PMC7224695; doi:10.7554/eLife.55687)
Supplement: Supplementary file 3. [file elife-55687-supp3.pdf]

supplementary file 3: NDMMs detected from *Pristionchus* species and NMR data of isolated or enriched NDMMs Pages

Table 1. Known NDMMs detected from *Pristionchus* nematodes. S2
Table 2. Novel DASC chemicals detected from *Pristionchus* nematodes. S3
Table 3. Novel UBAS chemicals detected from *Pristionchus* nematodes. S4
Table 4. Novel UPAS chemicals detected from *Pristionchus* nematodes. S5
Table 5. Producers of unquantifiable novel chemicals. S6
Table 6. Procedures of isolating target ascarosides from *exo*-metabolomes. S8
Table 7. NMR spectroscopic data of isolated dasc#3 (**1**) from *P. fukushimae*. S9
Table 8. NMR spectroscopic data of isolated dasc#4 (**2**) from *P. taiwanensis*. S10
Table 9. NMR spectroscopic data of isolated dasc#5 (**3**) from *D. magnus*. S11
Table 10. NMR spectroscopic data of isolated dasc#6 (**4**) from *P. entomophagus*. S12
Table 11. NMR spectroscopic data of isolated dasc#10 (**5**) from *P. mayeri*. S13
Table 12. NMR spectroscopic data of isolated dasc#14 (**6**) from *P. entomophagus*. S14
Table 13. NMR spectroscopic data of isolated dasc#17 (**8**) from *P. uniformis*. S15
Table 14. NMR spectroscopic data of isolated paso#9 from *P. pacificus*. S16
Table 15. NMR spectroscopic data of isolated ubas#30 (**9**) from *P. maxplancki*. S17
Table 16. NMR spectroscopic data of isolated ubas#34 (**11**) from *P. quartusdecimus*. S18
Table 17. NMR spectroscopic data of isolated ubas#35 (**12**) from *P. quartusdecimus*. S19
Table 18. NMR spectroscopic data of isolated ubas#28 (**13**) from *P. fukushimae*. S20
Table 19. NMR spectroscopic data of isolated upas#34 (**14**) from *P. quartusdecimus*. S21
Table 20. NMR spectroscopic data of isolated upas#28 (**15**) from *P. fukushimae*. S22
References S23

Table 1. UPLC-HR(ESI⁻)-qTOF-MS detected known ascarosides and paratosides from the *exo*-metabolomes of *Pristionchus* nematodes.

Name [SMID]	Nomenclature	RT [min]	Obs. [*m/z*]	Calc. [*m/z*]	Error [ppm]	Ion Formula $[M - H]^-$	Families of Chemicals
ascr#5	asc-ωC3‡	14.08	219.0875	219.1874	-0.2	$C_9H_{15}O_6^-$	Simple NDMM
ascr#11	asc-C4	15.08	233.1029	233.1031	0.9	$C_{10}H_{17}O_6^-$	
ascr#9	asc-C5	16.02	247.1180	247.1187	3.1	$C_{11}H_{19}O_6^-$	
ascr#12	asc-C6	17.12	261.1332	261.1344	4.3	$C_{12}H_{21}O_6^-$	
ascr#7	asc-ΔC7	17.90	273.1346	273.1344	-0.3	$C_{13}H_{21}O_6^-$	
ascr#1	asc-C7	18.28	275.1489	275.1500	4.2	$C_{13}H_{23}O_6^-$	
ascr#14	asc-C8	19.51	289.1657	289.1657	0.0	$C_{14}H_{25}O_6^-$	
ascr#10	asc-C9	20.80	303.1805	303.1813	2.7	$C_{15}H_{27}O_6^-$	
ascr#18	asc-C11	23.69	331.2119	331.2126	2.1	$C_{17}H_{31}O_6^-$	
part#9	par-C5	16.21	247.1181	247.1187	2.4	$C_{11}H_{19}O_6^-$	
npar#1	-	17.25	641.2431	641.2424	-1.1	$C_{26}H_{37}N_6O_{13}^-$	NPAR
npar#2	-	17.96	509.2003	509.2002	-0.3	$C_{21}H_{29}N_6O_9^-$	
npar#3	-	17.87	525.1953	525.1951	-0.5	$C_{21}H_{29}N_6O_{10}^-$	
pasc#9	-	20.42	466.2071	466.2083	2.5	$C_{23}H_{32}NO_9^-$	PASC
pasc#12	-	21.00	480.2233	480.2239	1.2	$C_{24}H_{34}NO_9^-$	
pasc#1	-	21.68	494.2398	494.2396	-0.4	$C_{25}H_{36}NO_9^-$	
pasa#9	-	23.55	585.2473	585.2454	-3.4	$C_{30}H_{37}N_2O_{10}^-$	
paso#9	-	22.16	601.2405	601.2403	-0.4	$C_{30}H_{37}N_2O_{11}^-$	
dasc#1	4'-(asc-C7#)-asc-C7#	22.55	533.2961	533.2967	1.2	$C_{26}H_{45}O_{11}^-$	DASC
ubas#1	4'-UB-2'-(asc-ωC5#)-asc-C5#	20.56	605.2926	605.2927	0.1	$C_{27}H_{45}N_2O_{13}^-$	UBAS
ubas#2	4'-UB-2'-(asc-C6#)-asc-C5#	21.03	619.3092	619.3084	-1.4	$C_{27}H_{45}N_2O_{13}^-$	
ubas#5	4'-UB-asc-C4	17.63	361.1612	361.1616	1.1	$C_{15}H_{25}N_2O_8^-$	
ubas#3	4'-UB-asc-C5	18.32	375.1778	375.1773	-1.3	$C_{16}H_{27}N_2O_8^-$	
ubas#12	4'-UB-asc-C6	19.05	389.1928	389.1929	0.4	$C_{17}H_{29}N_2O_8^-$	
ubas#4	4'-UB-asc-C7	20.06	403.2054	403.2086	7.8	$C_{18}H_{31}N_2O_8^-$	

‡ indicates the fatty acid side chain (aglycone) length of an ascaroside.

# indicates the two fatty acid side chain lengths in DASC and UBAS chemicals. (Definition of the ascarosides units in DASC and UBAS chemicals is described in ***Figure 4 — figure supplement 1A-B*** and ***Figure 5 — figure supplement 3C***).

Table 2. UPLC-HR(ESI$^{-/+}$)-qTOF-MS detected the DASC family of novel ascarosides from the *exo*-metabolomes of *Pristionchus* nematodes.

**Name [SMID]**	**Nomenclature**	**RT [min]**	**Obs. [*m/z*]**	**Calc. [*m/z*]**	**Error [ppm]**	**Ion Formula [adducts assignments]**	
dasc#2	2'/4'-(asc-C4)-asc-C4#	18.30	449.2026	449.2028	0.6	$C_{20}H_{33}O_{11}^-$	[M - H]$^-$
			473.1976	473.1993	1.7	$C_{20}H_{34}NaO_{11}^+$	[M + Na]$^+$
dasc#3 (**1**)	2'-(asc-C5)-asc-C4	18.91	463.2178	463.2185	1.4	$C_{21}H_{35}O_{11}^-$	[M - H]$^-$
			487.2137	487.2150	2.6	$C_{21}H_{36}NaO_{11}^+$	[M + Na]$^+$
dasc#4 (**2**)	2'-(asc-C5)-asc-C5	19.48	477.2335	477.2341	1.3	$C_{22}H_{37}O_{11}^-$	[M - H]$^-$
			501.2307	501.2306	0.0	$C_{22}H_{38}NaO_{11}^+$	[M + Na]$^+$
dasc#5 (**3**)	4'-(asc-C5)-asc-C5	19.35	477.2343	477.2341	-0.3	$C_{22}H_{37}O_{11}^-$	[M - H]$^-$
			501.2327	501.2306	-4.1	$C_{22}H_{38}NaO_{11}^+$	[M + Na]$^+$
dasc#6 (**4**)	2'-(asc-C6)-asc-C5	20.16	491.2487	491.2498	2.3	$C_{23}H_{39}O_{11}^-$	[M - H]$^-$
			515.2474	515.2463	-2.2	$C_{23}H_{40}NaO_{11}^+$	[M + Na]$^+$
dasc#7	2'/4'-(asc-C7)-asc-C4#	20.34	491.2499	491.2498	-0.2	$C_{23}H_{39}O_{11}^-$	[M - H]$^-$
			515.2446	515.2463	3.3	$C_{23}H_{40}NaO_{11}^+$	[M + Na]$^+$
dasc#8	2'/4'-(asc-C6)-asc-C6#	20.75	505.2652	505.2654	0.4	$C_{24}H_{41}O_{11}^-$	[M - H]$^-$
			529.2639	529.2619	-3.8	$C_{24}H_{42}NaO_{11}^+$	[M + Na]$^+$
dasc#9	2'-(asc-C7)-asc-C5†	20.95	505.2662	505.2654	-1.5	$C_{24}H_{41}O_{11}^-$	[M - H]$^-$
			529.2625	529.2619	-1.0	$C_{24}H_{42}NaO_{11}^+$	[M + Na]$^+$
dasc#10 (**5**)	4'-(asc-C5)-asc-C7	21.05	505.2659	505.2654	-0.9	$C_{24}H_{41}O_{11}^-$	[M - H]$^-$
			529.2651	529.2619	6.0	$C_{24}H_{42}NaO_{11}^+$	[M + Na]$^+$
dasc#11	2'/4'-(asc-C7)-asc-C6#	21.60	519.2801	519.2811	1.9	$C_{25}H_{43}O_{11}^-$	[M - H]$^-$
			543.2766	543.2776	1.7	$C_{25}H_{44}NaO_{11}^+$	[M + Na]$^+$
dasc#12	4'-(asc-C6)-asc-C7†	21.54	519.2825	519.2811	-2.8	$C_{25}H_{43}O_{11}^-$	[M - H]$^-$
			543.2798	543.2776	-4.1	$C_{25}H_{44}NaO_{11}^+$	[M + Na]$^+$
dasc#13	2'/4'-(asc-C5)-asc-C9#	23.17	533.2961	533.2967	1.3	$C_{26}H_{45}O_{11}^-$	[M - H]$^-$
			557.2925	557.2932	1.4	$C_{26}H_{46}NaO_{11}^+$	[M + Na]$^+$
dasc#14 (**6**)	4'-(asc-ΔC7)-asc-C5	20.55	503.2492	503.2498	0.6	$C_{24}H_{39}O_{11}^-$	[M - H]$^-$
			522.2938	522.2909	-5.5	$C_{24}H_{44}NaO_{11}^+$	[M + Na]$^+$
dasc#15	4'-(asc-ΔC7)-asc-C6†	21.26	517.2662	517.2654	-0.8	$C_{25}H_{41}O_{11}^-$	[M - H]$^-$
			541.2632	541.2619	-2.3	$C_{25}H_{42}NaO_{11}^+$	[M + Na]$^+$
dasc#16 (**7**)	4'-(asc-ΔC7)-asc-C7	22.10	531.2815	531.2811	-0.4	$C_{26}H_{43}O_{11}^-$	[M - H]$^-$
			550.3216	550.3222	1.0	$C_{26}H_{48}NaO_{11}^+$	[M + Na]$^+$
dasc#17 (**8**)	4'-(asc-ΔC7)-asc-ΔC7	21.90	529.2652	529.2654	0.2	$C_{26}H_{41}O_{11}^-$	[M - H]$^-$
			553.2648	552.2619	-5.2	$C_{26}H_{42}NaO_{11}^+$	[M + Na]$^+$

† indicates DASCs where linkage position assignment is tentative: linkage is likely at the 2'-position for dasc#9, and at the 4'-position for dasc#12 and dasc#15.

# indicates DASCs where linkage position (2'- or 4'-position) has not been characterized.

Table 3. UPLC-HR(ESI$^{-/+}$)-qTOF-MS detected the UBAS family of novel ascarosides from the *exo*-metabolomes of *Pristionchus* nematodes.

**Name [SMID]**	**Nomenclature**	**RT [min]**	**Obs. [*m/z*]**	**Calc. [*m/z*]**	**Error [ppm]**	**Ion Formula [adducts assignments]**	
ubas#26	4'-UB-asc-ωC3	16.77	347.1458	347.1460	0.6	$C_{14}H_{23}N_2O_8^-$	[M - H]$^-$
			349.1626	349.1605	-5.8	$C_{14}H_{25}N_2O_8^+$	[M + H]$^+$
ubas#27	4'-UB-2'-(asc-C5)-asc-ωC3	19.27	577.2622	577.2614	-1.3	$C_{25}H_{41}N_2O_{13}^-$	[M - H]$^-$
			579.2778	579.2760	-3.1	$C_{25}H_{43}N_2O_{13}^+$	[M + H]$^+$
ubas#28 (**13**)	4'-UB-2'-(asc-C5)-asc-C4	20.18	591.2781	591.2771	-1.7	$C_{26}H_{43}N_2O_{13}^-$	[M - H]$^-$
			593.2916	593.1916	0.1	$C_{26}H_{45}N_2O_{13}^+$	[M + H]$^+$
ubas#29	4'-UB-2'-(asc-C6)-asc-C4	20.63	605.2952	605.2927	-4.1	$C_{27}H_{45}N_2O_{13}^-$	[M - H]$^-$
			607.3068	607.3073	0.8	$C_{27}H_{47}N_2O_{13}^+$	[M + H]$^+$
ubas#30 (**9**)	4'-UB-2'-(asc-C5)-asc-C5	20.52	605.2954	605.2927	-4.5	$C_{27}H_{45}N_2O_{13}^-$	[M - H]$^-$
			607.3084	607.3073	-1.9	$C_{27}H_{47}N_2O_{13}^+$	[M + H]$^+$
ubas#31	4'-UB-2'-(asc-C6)-asc-C6	21.66	633.3261	633.3240	-3.4	$C_{29}H_{49}N_2O_{13}^-$	[M - H]$^-$
			635.3382	635.3386	0.4	$C_{29}H_{51}N_2O_{13}^+$	[M + H]$^+$
ubas#32 (**10**)	4'-UB-2'-(asc-C5)-asc-C7	21.99	633.3248	633.3240	-1.3	$C_{29}H_{49}N_2O_{13}^-$	[M - H]$^-$
			635.3365	635.3386	2.1	$C_{29}H_{51}N_2O_{13}^+$	[M + H]$^+$
ubas#33	4'-UB-2'-(asc-ωC3)-asc-C4	19.05	563.2459	563.2458	-0.3	$C_{24}H_{39}N_2O_{13}^-$	[M - H]$^-$
			565.2674	565.2603	5.1	$C_{24}H_{41}N_2O_{13}^+$	[M + H]$^+$
ubas#34 (**11**)	4'-UB-2'-(asc-C4)-asc-C4	19.57	577.2607	577.2614	1.2	$C_{25}H_{41}N_2O_{13}^-$	[M - H]$^-$
			579.2767	579.2760	-1.2	$C_{25}H_{43}N_2O_{13}^+$	[M + H]$^+$
ubas#35 (**12**)	4'-UB-2'-(asc-C4)-asc-C5	19.96	591.2786	591.2771	-2.5	$C_{26}H_{43}N_2O_{13}^-$	[M - H]$^-$
			593.2925	593.2916	-1.4	$C_{26}H_{45}N_2O_{13}^+$	[M + H]$^+$
ubas#36	4'-UB-2'-(asc-C4)-asc-C6	20.48	605.2934	605.2927	-1.2	$C_{27}H_{45}N_2O_{13}^-$	[M - H]$^-$
			607.3091	607.3073	-3.0	$C_{27}H_{47}N_2O_{13}^+$	[M + H]$^+$
ubas#37	4'-UB-2'-(asc-C4)-asc-C7	21.35	619.3103	619.3084	-3.1	$C_{28}H_{47}N_2O_{13}^-$	[M - H]$^-$
			621.3243	621.3229	-2.2	$C_{28}H_{49}N_2O_{13}^+$	[M + H]$^+$

Table 4. UPLC-HR(ESI$^{-/+}$)-qTOF-MS detected the UPAS family of novel ascarosides from the *exo*-metabolomes of *Pristionchus* nematodes.

**Name [SMID]**	**Nomenclature**	**RT [min]**	**Obs. [*m*/*z*]**	**Calc. [*m*/*z*]**	**Error [ppm]**	**Ion Formula [adducts assignments]**	
upas#5	4'-UP-asc-C4	16.63	347.1464	347.1460	-1.1	$C_{14}H_{23}N_2O_8^-$	$[M - H]^-$
			349.1640	349.1605	-9.8	$C_{14}H_{25}N_2O_8^+$	$[M + H]^+$
upas#3	4'-UP-asc-C5	17.47	361.1615	361.1616	0.3	$C_{15}H_{25}N_2O_8^-$	$[M - H]^-$
			363.1773	363.1762	-3.1	$C_{15}H_{27}N_2O_8^+$	$[M + H]^+$
upas#4	4'-UP-asc-C7	19.34	389.1935	389.1929	-1.4	$C_{17}H_{29}N_2O_8^-$	$[M - H]^-$
			391.2070	391.2075	1.2	$C_{17}H_{31}N_2O_8^+$	$[M + H]^+$
upas#34 (**14**)	4'-UP-2'-(asc-C4)-asc-C4	18.83	563.2459	563.2458	-0.3	$C_{24}H_{39}N_2O_{13}^-$	$[M - H]^-$
			565.2628	565.2603	-4.3	$C_{24}H_{41}N_2O_{13}^+$	$[M + H]^+$
upas#28 (**15**)	4'-UP-2'-(asc-C5)-asc-C4	19.53	577.2608	577.2596	-2.1	$C_{25}H_{41}N_2O_{13}^-$	$[M - H]^-$
			579.2771	579.2760	-2.0	$C_{25}H_{43}N_2O_{13}^+$	$[M + H]^+$
upas#30	4'-UP-2'-(asc-C5)-asc-C5	19.92	591.2777	591.2771	-1.1	$C_{26}H_{43}N_2O_{13}^-$	$[M - H]^-$
			593.2974	593.2916	-5.2	$C_{26}H_{45}N_2O_{13}^+$	$[M + H]^+$
upas#32	4'-UP-2'-(asc-C5)-asc-C7	21.54	619.3092	619.3084	-1.4	$C_{28}H_{47}N_2O_{13}^-$	$[M - H]^-$
			621.3267	621.3229	-6.1	$C_{28}H_{49}N_2O_{13}^+$	$[M + H]^+$

Table 5. Several detected chemicals were not quantified and included in ***Figure 2*** in the main text due to their very low ion abundance. Thus, their relevant producers were described here. UPAS chemicals were also convergently distributed in both *pacificus*- and *triformis*-clades.

**Name [SMID]**	**Nomenclature**	**Nematode (Producer)**
ascr#18	asc-C11	*P. atlanticus*
dasc#2	2'/4'-(asc-C4)-asc-C4	*P. quartusdecimus* and *P. fukushimae*
dasc#7	2'/4'-(asc-C7)-asc-C4	*P. triformis*
dasc#8	2'/4'-(asc-C6)-asc-C6	*P. triformis*
dasc#11	2'/4'-(asc-C7)-asc-C6	*P. triformis*
dasc#13	2'/4'-(asc-C5)-asc-C9	*P. maxplancki*
ubas#31	4'-UB-2'-(asc-C6)-asc-C6	*P. maxplancki*
ubas#37	4'-UB-2'-(asc-C4)-asc-C7	*P. quartusdecimus*
upas#5	4'-UP-asc-C4	*P. triformis* (*triformis*-clade)
upas#3	4'-UP-asc-C5	*P. maxplancki* (*pacificus*-clade)
upas#4	4'-UP-asc-C7	*P. maxplancki* (*pacificus*-clade)
upas#34 (**14**)	4'-UP-2'-(asc-C4)-asc-C4	*P. quartusdecimus* (*pacificus*-clade)
upas#28 (**15**)	4'-UP-2'-(asc-C5)-asc-C4	*P. fukushimae* (*triformis*-clade)
upas#30	4'-UP-2'-(asc-C5)-asc-C5	*P. laevicollis* and *P. maxplancki* (*pacificus*-clade)
upas#32	4'-UP-2'-(asc-C5)-asc-C7	*P. maxplancki* (*pacificus*-clade)

Table 6a. Procedures for isolating target ascarosides from *exo*-metabolomes. Major components of simple and modular ascarosides were initially fractionated by reverse phase C18 solid phase extraction (SPE), and further purified or enriched using semi-preparative HPLC. Purified or partially isolated materials were subjected for NMR measurements. For example, SPE60/B12 indicates that the target compound was fractionated using a solvent mixture of 60% MeOH and 40% water, which was further purified by semi-preparative HPLC, and finally enriched in B12 fraction that was dried for NMR data acquirements.

ASCR<br>Nematodes	ascr#9	ascr#1	ascr#7	ascr#10	dasc#3<br>(**1**)	dasc#4<br>(**2**)	dasc#5<br>(**3**)	dasc#6<br>(**4**)	dasc#10<br>(**5**)	dasc#14<br>(**6**)	dasc#16<br>(**7**)	dasc#17<br>(**8**)
P. pacificus						SPE50<br>B8						
P. taiwanensis				SPE50<br>B17		SPE50<br>B8						
P. laevicollis												
P. maxplancki												
P. quartusdecimus												
P. mayeri							SPE50<br>B8		SPE60<br>B16			
P. bulgaricus											SPE70<br>B22	
P. uniformis			SPE30<br>A88									SPE70<br>B20
P. entomophagus	SPE30<br>A77					SPE50<br>B7		SPE50<br>B11		SPE60<br>B15		
P. fukushimae					SPE50<br>B4							
P. triformis		SPE40<br>B2										
D. magnus							SPE50<br>B7					

Table 6b. Procedures for isolating target ascarosides from *exo*-metabolomes. Major components of modular ascarosides were initially fractionated by reverse phase C18 solid phase extraction (SPE) and further purified or enriched using semi-preparative HPLC. Purified or partially isolated materials were subjected for NMR measurements as described above.

ASCR / Nematodes	pasc#9	paso#9	ubas#30 (**9**)	ubas#1	ubas#32 (**10**)	ubas#34 (**11**)	ubas#35 (**12**)	ubas#5	ubas#28 (**13**)	upas#34 (**14**)	upas#28 (**15**)
P. pacificus		SPE60 B22		SPE60 B12							
P. taiwanensis	SPE50 B12			SPE60 B13							
P. laevicollis				SPE60 B12							
P. maxplancki			SPE60 B12		SPE70 B20						
P. quartusdecimus						SPE50 B8	SPE50 B11			SPE50 B3	
P. mayeri											
P. bulgaricus											
P. uniformis											
P. entomophagus											
P. fukushimae									SPE60 B11		SPE60 B8
P. triformis								SPE40 A87			
D. magnus											

Table 7. ^{1}H (800 MHz) NMR spectroscopic data of natural **dasc#3** [2'-(asc-C5)-asc-C4, **1**] (CD_3OD) isolated from the *exo*-metabolome of *P. fukushimae*. Chemical shift was referenced to ($CD_2\underline{H}OD$) = 3.31 ppm. NMR data of dasc#3 [2'-(asc-C5)-asc-C4, **1**] are derived from NMR spectra in ***supplementary file 1b – Figure 1***.

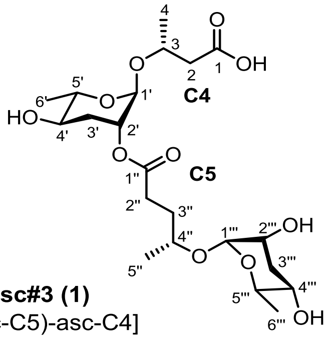

dasc#3 (1)
[2'-(asc-C5)-asc-C4]

Position	δ_H (mult., *J* in Hz, intensity)
1	-
2a	2.27 (*dd*, $J_{2a,\,2b}$ = 14.5, $J_{2a,\,3}$ = 6.8, 1H)
2b	2.49 (*dd*, $J_{2b,\,3}$ = 7.2, 1H)
3	4.25 (*m*, 1H)
4	1.16 (*d*, $J_{3,\,4}$ = 6.2, 3H)
1'	4.74 (*s*, 1H)
2'	4.78 (*s.br*, 1H)
3'ax	1.91 (*ddd*, $J_{2',\,3'ax}$ = 3.3, $J_{3'ax,\,3'eq}$ = 13.8, 1H)
3'eq	1.98 (*ddd*, $J_{2',\,3'eq}$ = 5.0, 1H)
4'	3.34 (*ddd*, $J_{3'ax,\,4'}$ = 11.0, $J_{3'eq,\,4'}$ = 4.0, 1H)
5'	3.79 (*dq*, $J_{4',\,5'}$ = 9.4, 1H)
6'	1.22 (*d*, $J_{5',\,6'}$ = 6.2, 3H)
1''	-
2''	2.50 (*m*, 2H)
3''	1.82 (*m*, 2H)
4''	3.85 (*m*, 1H)
5''	1.14 (*d*, $J_{4'',\,5''}$ = 6.1, 3H)
1'''	4.64 (*s*, 1H)
2'''	3.71 (*s.br*, $J_{1''',\,2'''}$ = 2.7, 1H)
3'''ax	1.77 (*ddd*, $J_{2''',\,3'''ax}$ = 3.2, $J_{3'''ax,\,3'''eq}$ = 13.5, 1H)
3'''eq	1.94 (*ddd*, $J_{2''',\,3'''eq}$ = 5.1, 1H)
4'''	3.50 (*ddd*, $J_{3'''ax,\,4'''}$ = 11.0, $J_{3'''eq,\,4'''}$ = 4.0, 1H)
5'''	3.63 (*dq*, $J_{4''',\,5'''}$ = 9.8, 1H)
6'''	1.22 (*d*, $J_{5''',\,6'''}$ = 6.2, 3H)

Table 8. ^{1}H (800 MHz), ^{13}C (200 MHz), HMBC and NOESY NMR spectroscopic data of natural **dasc#4** [2'-(asc-C5)-asc-C5, **2**] (CD_3OD) isolated from the *exo*-metabolome of *P. taiwanensis*. dasc#4 [2'-(asc-C5)-asc-C5, **2**] was also isolated from *P. entomophagus* and *P. pacificus*. Carbon shifts were determined from HSQC and HMBC spectra. Chemical shifts were referenced to ($CD_2\underline{H}OD$) = 3.31 ppm and ($\underline{C}D_3OD$) = 49.0 ppm. NMR data are derived from spectra in ***supplementary file 1b – Figures 2-15***.

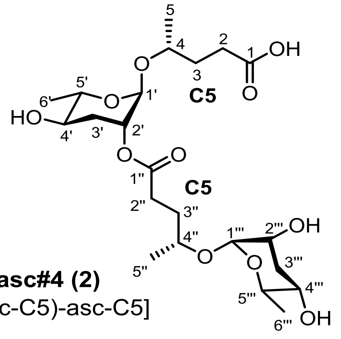

dasc#4 (2)
[2'-(asc-C5)-asc-C5]

Position	δ_C (ppm)	δ_H (mult., *J* in Hz, intensity)	HMBC	NOESY
1	180.1	-	-	-
2a	33.1	2.33 (*m*, 1H)	1, 3, 4	2b, 3, 4, 5
2b	33.1	2.38 (*m*, 1H)	1, 3, 4	2a, 3, 4, 5
3	34.1	1.83 (*m*, 2H)	1, 2, 4, 5	1', 2a, 2b, 4, 5
4	72.1	3.82 (*m*, 1H)	2, 1'	1', 3, 5
5	18.7	1.15 (*d*, $J_{4,5}$ = 6.3, 3H)	3, 4	1', 3, 4
1'	94.1	4.72 (*s*, 1H)	2', 3'	2', 4, 5
2'	72.6	4.79 (*s.br*, $J_{1',2'}$ = 2.1, 1H)	4', 1''	1', 3' ax, 3' eq
3'ax	33.1	1.88 (*ddd*, $J_{2',3'ax}$ = 3.1, $J_{3'ax,3'eq}$ = 13.6, 1H)	1', 2', 4'	2', 3' eq, 4', 5'
3'eq	33.1	2.02 (*ddd*, $J_{2',3'eq}$ = 4.8, 1H)	1', 2', 4'	2', 3' ax, 4'
4'	68.4	3.40 (*ddd*, $J_{3'ax,4'}$ = 11.1, $J_{3'eq,4'}$ = 4.0, 1H)		3'ax, 3' eq, 6'
5'	70.8	3.69 (*dq*, $J_{4',5'}$ = 9.4, 1H)	4'	3'ax, 6'
6'	17.9	1.22 (*d*, $J_{5',6'}$ = 6.2, 3H)	4', 5'	4', 5'
1''	172.8	-	-	-
2''	31.1	2.50 (*m*, 2H)	1'', 3'', 4''	3'', 4'', 5''
3''	33.0	1.84 (*m*, 2H)	4''	1''', 2'', 4'', 5''
4''	71.1	3.85 (*m*, 1H)	2'', 1'''	1''', 3'', 5''
5''	18.8	1.16 (*d*, $J_{4'',5''}$ = 6.1, 3H)	3'', 4''	1''', 3'', 4''
1'''	97.1	4.65 (*s*, 1H)	2''', 3'''	2''', 4'', 5''
2'''	69.6	3.72 (*s.br*, $J_{1''',2'''}$ = 2.5, 1H)	4'''	1''', 3'''ax, 3'''eq
3'''ax	35.7	1.76 (*ddd*, $J_{2''',3'''ax}$ = 3.1, $J_{3'''ax,3'''eq}$ = 13.1, 1H)	4'''	2''', 3'''eq, 4''', 5'''
3'''eq	35.7	1.96 (*ddd*, $J_{2''',3'''eq}$ = 4.7, 1H)	4'''	2''', 3'''ax, 4'''
4'''	68.0	3.51 (*ddd*, $J_{3'''ax,4''}$ = 11.0, $J_{3'''eq,4'''}$ = 4.0, 1H)	5'''	3'''ax, 3'''eq, 6'''
5'''	71.1	3.57 (*dq*, $J_{4''',5'''}$ = 9.3, 1H)	4'''	2'''', 3'''ax, 6'''
6'''	17.7	1.23 (*d*, $J_{5''',6'''}$ = 6.2, 3H)	4''', 5'''	4''', 5'''

Table 9. ^{1}H (800 MHz), ^{13}C (200 MHz), HMBC and NOESY NMR spectroscopic data of natural **dasc#5** [4'-(asc-C5)-asc-C5, **3**] (CD_3OD) isolated from the *exo*-metabolome of *D. magnus*. dasc#5 [4'-(asc-C5)-asc-C5, **3**] was also isolated from *P. mayeri*. Carbon shifts were determined from HSQC and HMBC spectra. Chemical shifts were referenced to ($CD_2\underline{H}OD$) = 3.31 ppm and ($\underline{C}D_3OD$) = 49.0 ppm. NMR data are derived from spectra in ***supplementary file 1b – Figures 16-26***.

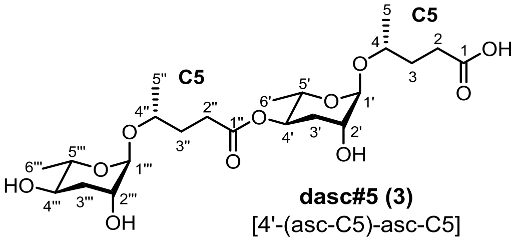

dasc#5 (3)
[4'-(asc-C5)-asc-C5]

Position	δ_C (ppm)	δ_H (mult., *J* in Hz, intensity)	HMBC	NOESY
1	181.5	-	-	-
2a	34.7	2.24 (*m*, 1H)	1, 3, 4	2b, 3
2b	34.7	2.35 (*m*, 1H)	1, 3, 4	2a, 3
3	34.1	1.82 (*m*, 2H)	1, 2, 4, 5	2a, 2b, 4, 5
4	71.7	3.82 (*m*, 1H)	2, 1'	1', 3, 5
5	18.1	1.15 (*d*, $J_{4,5}$ = 6.3, 3H)	3, 4	1', 3, 4
1'	97.0	4.69 (*s*, 1H)	4, 3', 5'	2', 4, 5
2'	69.2	3.72 (*s.br*, $J_{1',2'}$ = 2.6, 1H)	4'	1', 3' ax, 3' eq
3'ax	32.8	1.87 (*ddd*, $J_{2',3'ax}$ = 3.0, $J_{3'ax,3'eq}$ = 13.1, 1H)	2'	2', 3' eq, 4', 5'
3'eq	32.8	2.04 (*ddd*, $J_{2',3'eq}$ = 4.9, 1H)	2'	3' ax, 4'
4'	71.2	4.86 (*ddd*, $J_{3'ax,4'}$ = 11.2, $J_{3'eq,4'}$ = 4.1, 1H)	-	3'ax, 3' eq
5'	67.9	3.89 (*dq*, $J_{4',5'}$ = 10.0, 1H)	-	3'ax, 6'
6'	18.5	1.15 (*d*, $J_{5',6'}$ = 6.4, 3H)	4', 5'	4', 5'
1"	174.3	-	-	-
2"a	31.3	2.43 (*m*, 1H)	1", 3", 4"	2"b, 3"
2"b	31.3	2.47 (*m*, 1H)	1", 3", 4"	2"a, 3"
3"	35.0	1.80 (*m*, 2H)	1", 2", 4", 5"	2"a, 2"b, 4", 5"
4"	71.1	3.82 (*m*, 1H)	1'''	1''', 3", 5"
5"	18.1	1.15 (*d*, $J_{4",5"}$ = 6.3, 3H)	3", 4"	1''', 3", 4"
1'''	97.1	4.65 (*s*, 1H)	4", 3'''	2''', 4", 5"
2'''	69.5	3.72 (*s.br*, $J_{1''',2'''}$ = 2.6, 1H)	4'''	1''', 3'''ax, 3'''eq
3'''ax	35.7	1.77 (*ddd*, $J_{2''',3'''ax}$ = 3.2, $J_{3'''ax,3'''eq}$ = 13.3, 1H)	1''', 2'''	2''', 3'''eq, 4''', 5'''
3'''eq	35.7	1.95 (*ddd*, $J_{2''',3'''eq}$ = 4.8, 1H)	1''', 2'''	2''', 3'''ax, 4'''
4'''	68.2	3.51 (*ddd*, $J_{3'''ax,4'''}$ = 11.4, $J_{3'''eq,4'''}$ = 3.9, 1H)	5'''	3'''ax, 3'''eq, 6'''
5'''	71.1	3.58 (*dq*, $J_{4''',5'''}$ = 9.6, 1H)	-	3'''ax, 6'''
6'''	17.8	1.22 (*d*, $J_{5''',6'''}$ = 6.2, 3H)	4''', 5'''	4''', 5'''

Table 10. ^{1}H (800 MHz) NMR spectroscopic data of natural **dasc#6** [2'-(asc-C6)-asc-C5, **4**] (CD_3OD) isolated from the *exo*-metabolome of *P. entomophagus*. Chemical shift was referenced to ($CD_2\underline{H}OD$) = 3.31 ppm. NMR data are derived from spectra in ***supplementary file 1b – Figure 27***.

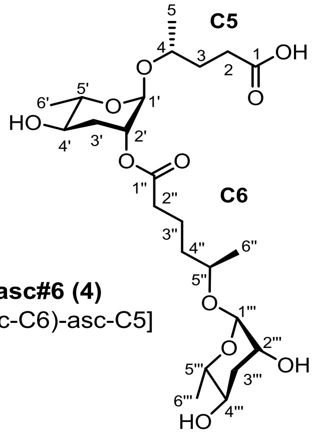

dasc#6 (4)
[2'-(asc-C6)-asc-C5]

Position	δ_H (mult., *J* in Hz, intensity)
1	-
2a	2.30 (*m*, 1H)
2b	2.38 (*m*, 1H)
3	1.81 (*m*, 2H)
4	3.83 (*m*, 1H)
5	1.14 (*d*, $J_{4, 5}$ = 6.3, 3H)
1'	4.72 (*s*, 1H)
2'	4.78 (*s.br*, $J_{1', 2'}$ = 2.1, 1H)
3'ax	1.90 (*ddd*, $J_{2', 3'ax}$ = 3.1, $J_{3'ax, 3'eq}$ = 13.3, 1H)
3'eq	2.00 (*ddd*, $J_{2', 3'eq}$ = 5.8, 1H)
4'	3.39 (*ddd*, $J_{3'ax, 4'}$ = 11.1, $J_{3'eq, 4'}$ = 4.0, 1H)
5'	3.70 (*dq*, $J_{4', 5'}$ = 9.3, 1H)
6'	1.21 (*d*, $J_{5', 6'}$ = 6.2, 3H)
1''	-
2''	2.21 (*t*, $J_{2'', 3''}$ = 6.1, 2H)
3''	1.59 (*m*, 2H)
4''	1.52 (*m*, 1H)
5''	3.81 (*m*, 1H)
6''	1.14 (*d*, $J_{5'', 6''}$ = 6.3, 3H)
1'''	4.65 (*s*, 1H)
2'''	3.71 (*s.br*, $J_{1''', 2'''}$ = 2.5, 1H)
3'''ax	1.77 (*ddd*, $J_{2''', 3'''ax}$ = 3.1, $J_{3'''ax, 3'''eq}$ = 13.3, 1H)
3'''eq	1.96 (*ddd*, $J_{2''', 3'''eq}$ = 6.3, 1H)
4'''	3.51 (*ddd*, $J_{3'''ax, 4''}$ = 11.0, $J_{3'''eq, 4'''}$ = 3.8, 1H)
5'''	3.61 (*dq*, $J_{4''', 5'''}$ = 9.3, 1H)
6'''	1.22 (*d*, $J_{5''', 6'''}$ = 6.2, 3H)

Table 11. ^{1}H (800 MHz) spectroscopic data of natural **dasc#10** [4'-(asc-C5)-asc-C7, **5**] (CD_3OD) isolated from the *exo*-metabolome of *P. mayeri*. Carbon shifts were determined from HSQC spectra. Chemical shifts was referenced to ($CD_2\underline{H}OD$) = 3.31 ppm and ($\underline{C}D_3OD$) = 49.0 ppm. n.d. denotes not determined. NMR data are derived from spectra in ***supplementary file 1b – Figures 28-31***.

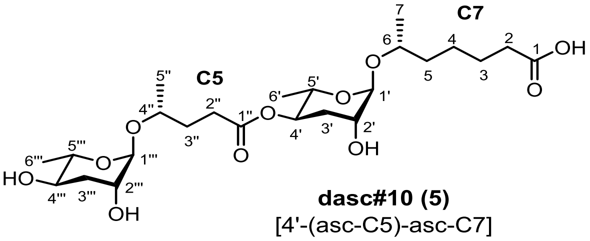

dasc#10 (5)
[4'-(asc-C5)-asc-C7]

Position	δ_C (ppm)	δ_H (mult., *J* in Hz, intensity)	NOESY
1	n.d.	-	-
2	39.1	2.18 (*m*, 2H)	3
3	27.2	1.61 (*m*, 2H)	2, 4
4	22.6	1.39 (*m*, 2H)	3, 5
5a	34.1	1.54 (*m*, 1H)	4, 6
5b	34.1	1.61 (*m*, 1H)	4, 6
6	72.3	3.79 (*m*, 1H)	5a, 5b, 7, 1'
7	18.8	1.15 (*d*, $J_{4'', 5''}$ = 6.1, 3H)	6, 1'
1'	97.4	4.68 (*s*, 1H)	6, 7, 2'
2'	69.2	3.71 (*s.br*, $J_{1', 2'}$ = 2.1, 1H)	1' , 3'ax, 3'eq
3'ax	33.4	1.84 (*ddd*, $J_{2', 3'ax}$ = 3.6, $J_{3'ax, 3'eq}$ = 12.8, 1H)	2', 3'eq, 4'
3'eq	33.4	2.04 (*ddd*, $J_{2', 3'eq}$ = 5.8, 1H)	2', 3'ax, 4'
4'	70.8	4.86 (*ddd*, $J_{3'ax, 4'}$ = 11.2, $J_{3'eq, 4'}$ = 4.0, 1H)	3'ax, 3'eq
5'	68.2	3.85 (*dq*, $J_{4', 5'}$ = 9.6, 1H)	3'ax, 6'
6'	18.5	1.15 (*d*, $J_{5', 6'}$ = 6.2, 3H)	5'
1''	n.d.	-	-
2''	39.2	2.47 (*m*, 2H)	3''
3''	35.2	1.80 (*m*, 2H)	2'', 4'', 5''
4''	71.8	3.83 (*m*, 1H)	1''', 3'', 5''
5''	18.5	1.15 (*d*, $J_{4'', 5''}$ = 6.1, 3H)	4'', 1'''
1'''	97.2	4.65 (*s*, 1H)	4'', 5'', 2'''
2'''	69.6	3.72 (*s.br*, $J_{1''', 2'''}$ = 2.5, 1H)	1''', 3'''ax, 3'''eq
3'''ax	35.8	1.76 (*ddd*, $J_{2''', 3'''ax}$ = 3.1, $J_{3'''ax, 3'''eq}$ = 13.6, 1H)	2''', 3'''eq, 4'''
3'''eq	35.8	1.95 (*ddd*, $J_{2''', 3'''eq}$ = 6.7, 1H)	2''', 3'''ax, 4'''
4'''	68.3	3.51 (*ddd*, $J_{3'''ax, 4''}$ = 11.0, $J_{3'''eq, 4'''}$ = 4.0, 1H)	3'''ax, 3'''eq
5'''	71.2	3.58 (*dq*, $J_{4''', 5'''}$ = 9.3, 1H)	3'''ax, 6'''
6'''	17.9	1.22 (*d*, $J_{5''', 6'''}$ = 6.2, 3H)	5'''

Table 12. ^{1}H (800 MHz), ^{13}C (200 MHz) and NOESY NMR spectroscopic data of natural **dasc#14** [4'-(asc-ΔC7)-asc-C5, **6**] (CD_3OD) isolated from the *exo*-metabolome of *P. entomophagus*. Carbon shifts were determined from HSQC and HMBC spectra. Chemical shifts were referenced to ($CD_2\underline{H}OD$) = 3.31 ppm and ($\underline{C}D_3OD$) = 49.0 ppm. n.d. denotes not determined. NMR data are derived from spectra in ***supplementary file 1b – Figures 32-39***.

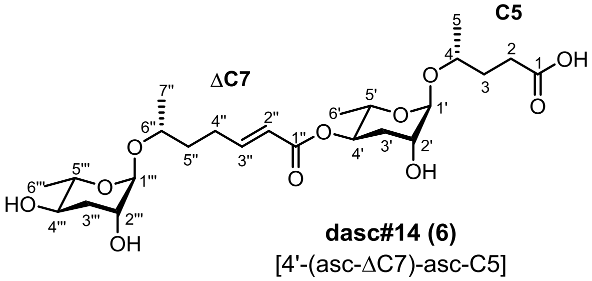

dasc#14 (6)
[4'-(asc-ΔC7)-asc-C5]

Position	δ_C (ppm)	δ_H (mult., *J* in Hz, intensity)	NOESY
1	n.d.	-	-
2a	35.3	2.28 (*dt*, $J_{2a, 2b}$ = 14.9, $J_{2a, 3}$ = 6.7, 1H)	2b, 3, 4, 5
2b	35.3	2.36 (*dt*, $J_{2b, 3}$ = 7.4, 1H)	2a, 3, 4, 5
3	34.9	1.77 (*m*, 2H)	2a, 2b, 4, 1', 5
4	71.9	3.83 (*m*, $J_{3, 4}$ = 5.8, 1H)	2a, 2b, 3, 5, 1'
5	18.5	1.16 (*d*, $J_{4, 5}$ = 6.1, 3H)	2a, 2b, 3, 4, 1'
1'	97.1	4.70 (*s*, 1H)	2', 4, 5
2'	69.5	3.73 (*s.br*, $J_{1', 2'}$ = 3.5, 1H)	1', 3'ax, 3'eq
3'ax	32.9	1.89 (*ddd*, $J_{2', 3'ax}$ = 3.0, $J_{3'ax, 3'eq}$ = 13.0, 1H)	2', 3'eq, 4', 5'
3'eq	32.9	2.05 (*ddd*, $J_{2', 3'eq}$ = 4.8, 1H)	3'ax, 4'
4'	71.0	4.91 (*ddd*, $J_{3'ax, 4'}$ = 11.7, $J_{3'eq, 4'}$ = 4.0, 1H)	5', 6', 3'ax, 3'eq
5'	68.0	3.91 (*dq*, $J_{4', 5'}$ = 9.5, 1H)	3'ax, 6'
6'	18.5	1.15 (*d*, $J_{5', 6'}$ = 6.2, 3H)	4', 5'
1''	n.d.	-	-
2''	122.0	5.87 (*d*, $J_{2'', 3''}$ = 15.7, 1H)	3'', 4'', 5''
3''	150.9	7.02 (*dt*, $J_{3'', 4''}$ = 6.8, 1H)	2'', 4'', 5''
4''	29.2	2.39 (*m*, 2H)	2'', 3'', 5'', 6'', 7''
5''	36.2	1.68 (*m*, 2H)	3'', 4'', 6'', 7''
6''	71.7	3.83 (*m*, 1H)	4'', 5'', 7'', 1'''
7''	17.6	1.16 (*d*, $J_{6'', 7''}$ = 6.1, 3H)	4'', 6'', 1'''
1'''	97.0	4.66 (*s*, 1H)	2''', 6'', 7''
2'''	69.4	3.72 (*s.br*, $J_{1''', 2'''}$ = 3.4, 1H)	1''', 3'''ax, 3'''eq
3'''ax	35.8	1.77 (*ddd*, $J_{2''', 3'''ax}$ = 3.1, $J_{3'''ax, 3'''eq}$ = 13.5, 1H)	2''', 3'''eq, 4''', 5'''
3'''eq	35.8	1.95 (*ddd*, $J_{2''', 3'''eq}$ = 4.7, 1H)	2''', 3'''ax, 4'''
4'''	68.1	3.52 (*ddd*, $J_{3'''ax, 4'''}$ = 12.0, $J_{3'''eq, 4'''}$ = 3.8, 1H)	3'''ax, 3'''eq, 6'''
5'''	71.1	3.60 (*dq*, $J_{4''', 5'''}$ = 9.4, 1H)	3'''ax, 6'''
6'''	18.0	1.22 (*d*, $J_{5''', 6'''}$ = 6.3, 3H)	4''', 5'''

Table 13. ^{1}H (800 MHz), ^{13}C (200 MHz) and NOESY NMR spectroscopic data of natural **dasc#17** [4'-(asc-ΔC7)-asc-ΔC7, **8**] (CD_3OD) isolated from the *exo*-metabolome of *P. uniformis*. Carbon shifts were determined from HSQC and HMBC spectra. Chemical shifts were referenced to ($CD_2\underline{H}OD$) = 3.31 ppm and ($\underline{C}D_3OD$) = 49.0 ppm. n.d. denotes not determined. NMR data are derived from spectra in ***supplementary file 1b – Figures 40-46***.

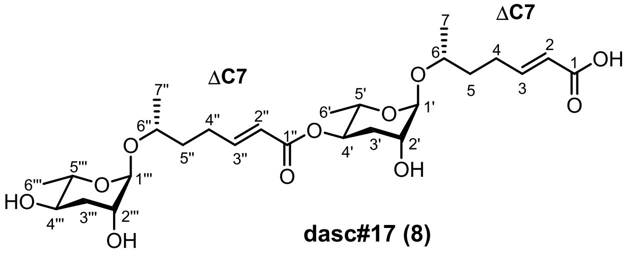

dasc#17 (8)
[4'-(asc-ΔC7)-asc-ΔC7]

Position	δ_C (ppm)	δ_H (mult., *J* in Hz, intensity)	NOESY
1	n.d.	-	-
2	128.9	5.86 (*d*, $J_{2,3}$ = 15.5, 1H)	3, 4, 5
3	143.2	6.68 (*dt*, $J_{3,4a}$ = 6.7, $J_{3,4b}$ = 7.1, 1H)	2, 4, 5
4a	29.0	2.23 (*m*, $J_{4a,4b}$ = 13.9, 1H)	2, 3, 5, 6, 7
4b	29.0	2.31 (*m*, 1H)	2, 3, 5, 6, 7
5	35.8	1.65 (*m*, 2H)	3, 4, 6, 7
6	71.6	3.84 (*m*, 1H)	4, 5, 7, 1'
7	18.0	1.15 (*d*, $J_{6,7}$ = 6.3, 3H)	5, 6, 1'
1'	97.3	4.71 (*s*, 1H)	6, 7, 2'
2'	68.8	3.74 (*s.br*, $J_{1',2'}$ = 3.5, 1H)	1', 3'ax, 3'eq
3'ax	32.6	1.88 (*ddd*, $J_{2',3'ax}$ = 3.0, $J_{3'ax,3'eq}$ = 13.0, 1H)	2', 3'eq, 5'
3'eq	32.6	2.06 (*ddd*, $J_{2',3'eq}$ = 4.9, 1H)	3'ax, 4'
4'	71.1	4.91 (*ddd*, $J_{3'ax,4'}$ = 11.2, $J_{3'eq,4'}$ = 4.0, 1H)	3'eq, 5', 6'
5'	68.1	3.88 (*dq*, $J_{4',5'}$ = 9.6, 1H)	4', 6', 3'ax
6'	18.6	1.14 (*d*, $J_{5',6'}$ = 6.3, 3H)	4', 5'
1"	n.d.	-	-
2"	121.9	5.89 (*d*, $J_{2'',3''}$ = 16.0, 1H)	3", 4", 5"
3"	150.7	7.02 (*dt*, $J_{3'',4''a}$ = 6.9, $J_{3'',4''b}$ = 7.2, 1H)	2", 4", 5"
4"a	29.3	2.36 (*m*, $J_{4''a,4''b}$ = 13.8, 1H)	3", 5", 6", 7"
4"b	29.3	2.40 (*m*, 1H)	3", 5", 6", 7"
5"	36.6	1.68 (*m*, 2H)	3", 4", 6", 7"
6"	71.2	3.82 (*m*, 1H)	4", 5", 7", 1"'
7"	18.7	1.16 (*d*, $J_{6'',7''}$ = 6.3, 3H)	5", 6", 1"'
1"'	97.0	4.66 (*s*, 1H)	6", 7", 2"'
2"'	69.9	3.72 (*s.br*, $J_{1''',2'''}$ = 3.6, 1H)	1"', 3"'ax, 3"'eq
3"'ax	35.6	1.77 (*ddd*, $J_{2''',3'''ax}$ = 3.1, $J_{3'''ax,3'''eq}$ = 13.2, 1H)	2"', 3"'eq, 5"'
3"'eq	35.6	1.96 (*ddd*, $J_{2''',3'''eq}$ = 4.9, 1H)	2"', 3"'ax, 4"'
4"'	68.0	3.52 (*ddd*, $J_{3'''ax,4'''}$ = 11.1, $J_{3'''eq,4'''}$ = 4.0, 1H)	3"'eq, 5"', 6"'
5"'	71.0	3.60 (*dq*, $J_{4''',5'''}$ = 9.3, 1H)	3"'ax, 4"', 6"'
6"'	17.9	1.22 (*d*, $J_{5''',6'''}$ = 6.2, 3H)	4"', 5"'

Table 14. ^{1}H (800 MHz), ^{13}C (200 MHz), HMBC and NOESY NMR spectroscopic data of natural **paso#9** (***Artyukhin et al., 2018***) (CD_3OD) isolated from the *exo*-metabolome of *P. pacificus*. Carbon shifts were determined from HSQC and HMBC spectra. Chemical shifts were referenced to ($CD_2\underline{H}OD$) = 3.31 ppm and ($\underline{C}D_3OD$) = 49.0 ppm. n.d. denotes not detected. NMR data are derived from spectra in ***supplementary file 1c – Figures 5-13***.

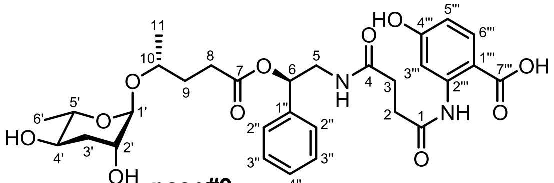

No.	δ_C [ppm]	δ_H [mult., *J* in Hz, intensity]	HMBC	NOESY
1	170.8	-	-	-
2	33.1	2.69 (*t*, $J_{2,3}$ = 7.1, 2H)	1, 4	3
3	30.8	2.56 (*t*, 2H)	1, 4	2
4	173.4	-	-	-
5a	44.2	3.51 (*dd*, $J_{5a,5b}$ = 14.3, $J_{5a,6}$ = 8.4, 1H)	4, 6, 1''	6, 2''
5b	44.2	3.59 (*dd*, $J_{5b,6}$ = 4.4, 1H)	4, 6, 1''	6, 2''
6	74.5	5.83 (*dd*, 1H)	2''	2''
7	173.0	-	-	-
8	30.7	2.52 (*m*, 2H)	7, 10	9, 10, 11
9	32.3	1.82 (*m*, 2H)	7, 8, 11	10, 11
10	70.3	3.77 (*m*, 1H)	n.d.	11, 1'
11	18.0	1.11 (*d*, $J_{10,11}$ = 6.1, 3H)	9, 10	1'
1'	96.1	4.63 (*s*, $J_{1',2'}$ = 2.3, 1H)	10, 5'	10, 11, 2'
2'	68.6	3.71 (*s.br*, $J_{2',3'ax}$ = 3.2, $J_{2',3'eq}$ = 4.8, 1H)	4'	3'ax, 3'eq
3'ax	34.8	1.76 (*ddd*, $J_{3'ax,3'eq}$ = 13.1, $J_{3'ax,4'}$ = 11.1, 1H)	1', 2', 4'	4', 5'
3'eq	34.8	1.95 (*ddd*, $J_{3'eq,4'}$ = 4.0, 1H)	1', 2', 4'	4', 3'ax
4'	67.1	3.51 (*ddd*, $J_{4',5'}$ = 9.2, 1H)	2' , 3'	6'
5'	70.1	3.60 (*dq*, $J_{5',6'}$ = 6.2, 1H)	n.d.	8, 9, 10, 3'ax, 6'
6'	17.0	1.19 (*d*, 3H)	4', 5'	5'
1''	138.3	-	-	-
2''	126.2	7.36 (*m*, 2H)	6, 4''	3''
3''	128.2	7.34 (*m*, 2H)	1''	2'', 4''
4''	127.9	7.28 (*m*, 1H)	2''	3''
1'''	114.2	-	-	-
2'''	141.8	-	-	-
3'''	105.8	8.04 (*d*, 1H)	1'''	
4'''	160.4	-	-	-
5'''	109.1	6.45 (*dd*, $J_{5''',3'''}$ = 2.4, 1H)	1''', 3'''	6'''
6'''	132.8	7.93 (*d*, $J_{6''',5'''}$ = 8.6, 1H)	7''', 4''', 2'''	5'''
7'''	173.4	-	-	-

Table 15. ^{1}H (800 MHz), ^{13}C (200 MHz), HMBC and NOESY NMR spectroscopic data of natural **ubas#30** [4'-UB-2'-(asc-C5)-asc-C5, **9**] (CD_3OD) isolated from the *exo*-metabolome of *P. maxplancki*. Carbon shifts were determined from HSQC and HMBC spectra. Chemical shifts were referenced to ($CD_2\underline{H}OD$) = 3.31 ppm and ($\underline{C}D_3OD$) = 49.0 ppm. n.d. denotes not detected. NMR data are derived from spectra in ***supplementary file 1d – Figures 1-8***.

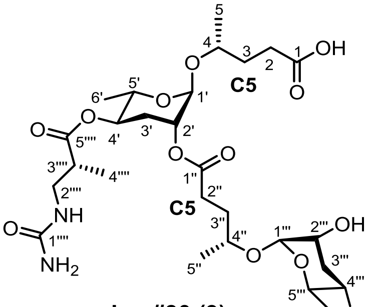

ubas#30 (9)
[4'-UB-2'-(asc-C5)-asc-C5]

Position	δ_C (ppm)	δ_H (mult., *J* in Hz, intensity)	HMBC	NOESY
1	181.3	-	-	-
2a	34.3	2.28 (*dt*, $J_{2a, 2b}$ = 14.8, $J_{2a, 3}$ = 8.1, 1H)	1, 3, 4	3, 4, 5, 5'
2b	34.3	2.34 (*dt*, $J_{2b, 3}$ = 8.4, 1H)	1, 3, 4	3, 4, 5, 5'
3	34.7	1.83 (*m*, 2H)	1, 2, 4, 5	2, 4, 5
4	72.9	3.85 (*m*, $J_{3, 4}$ = 6.6, 1H)	n.d.	2, 3, 5, 1', 5'
5	18.1	1.16 (*d*, $J_{4, 5}$ = 5.8, 3H)	3, 4	2, 3, 4, 1'
1'	94.4	4.78 (*s*, 1H)	4, 3', 5'	4, 5, 2'
2'	71.9	4.81 (*s.br*, $J_{1', 2'}$ = 2.5, 1H)	4', 1''	3'ax, 3'eq, 1'
3'ax	29.9	1.99 (*ddd*, $J_{2', 3'ax}$ = 3.1, $J_{3'ax, 3'eq}$ = 13.5, 1H)	n.d.	2', 3'eq, 4', 5'
3'eq	29.9	2.12 (*ddd*, $J_{2', 3'eq}$ = 4.7, 1H)	n.d.	2', 3'ax, 4'
4'	71.1	4.74 (*ddd*, $J_{3'ax, 4'}$ = 11.1, $J_{3'eq, 4'}$ = 4.1, 1H)	n.d.	3'ax, 3'eq, 5', 6'
5'	67.9	3.96 (*dq*, $J_{4', 5'}$ = 9.5, 1H)	n.d.	2, 3'ax, 4', 6'
6'	17.6	1.16 (*d*, $J_{5', 6'}$ = 6.0, 3H)	4', 5'	4', 5'
1''	173.9	-	-	-
2''	31.1	2.53 (*dd*, $J_{2'', 3''}$ = 6.2, 2H)	1'', 3'', 4''	3'', 4'', 5'', 5'''
3''a	32.9	1.82 (*m*, 1H)	1'', 4'', 5''	2'', 4'', 5''
3''b	32.9	1.88 (*m*, 1H)	1'', 4'', 5''	2'', 4'', 5''
4''	71.1	3.86 (*d*, $J_{3'', 4''}$ = 6.3, 1H)	n.d.	2'', 3'', 5'', 1''', 5'''
5''	18.7	1.17 (*d*, $J_{4'', 5''}$ = 6.0, 3H)	3'', 4''	2'', 3'', 4'', 1'''
1'''	97.1	4.66 (*s*, 1H)	4'', 3''', 5'''	4'', 5'', 2'''
2'''	69.7	3.73 (*s.br*, $J_{1''', 2'''}$ = 2.8, 1H)	4'''	1''', 3'''ax, 3'''eq
3'''ax	35.7	1.77 (*ddd*, $J_{2''', 3'''ax}$ = 3.3, $J_{3'''ax, 3'''eq}$ = 13.0, 1H)	n.d.	2''', 3'''eq, 4''', 5'''
3'''eq	35.7	1.95 (*ddd*, $J_{2''', 3'''eq}$ = 4.8, 1H)	n.d.	2''', 3'''ax, 4'''
4'''	68.0	3.51 (*ddd*, $J_{3'''ax, 4'''}$ = 11.1, $J_{3'''eq, 4'''}$ = 4.1, 1H)	n.d.	3'''ax, 3'''eq, 5''', 6'''
5'''	71.2	3.58 (*dq*, $J_{4''', 5'''}$ = 9.3, 1H)	n.d.	4''', 3'''ax, 6'''
6'''	17.9	1.22 (*d*, $J_{5''', 6'''}$ = 6.1, 3H)	4''', 5'''	4''', 5'''
1''''	161.8	-	-	-
2''''	43.4	3.26 (*m*, $J_{2'''', 3''''}$ = 6.7, 2H)	1'''', 3'''', 4'''', 5''''	3'''', 4''''
3''''	41.5	2.68 (*m*, 1H)	2'''', 4'''', 5''''	2'''', 4''''
4''''	14.7	1.14 (*d*, $J_{3'''', 4''''}$ = 7.1, 3H)	2'''', 3'''', 5''''	2'''', 3''''
5''''	175.6	-	-	-

Table 16. ^{1}H (800 MHz), ^{13}C (200 MHz), HMBC and NOESY NMR spectroscopic data of natural **ubas#34** [4'-UB-2'-(asc-C4)-asc-C4, **11**] (CD_3OD) isolated from the *exo*-metabolome of *P. quartusdecimus*. Carbon shifts were determined from HSQC and HMBC spectra. Chemical shifts are referenced to ($CD_2\underline{H}OD$) = 3.31 ppm and ($\underline{C}D_3OD$) = 49.0 ppm. n.d. denotes not detected. NMR data are derived from spectra in ***supplementary file 1d – Figures 21-28***.

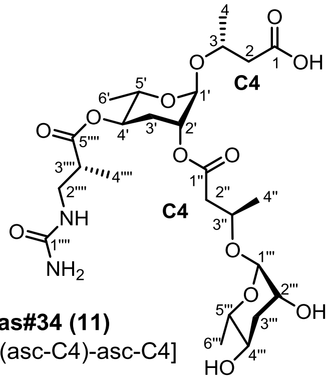

ubas#34 (11)
[4'-UB-2'-(asc-C4)-asc-C4]

Position	δ_C (ppm)	δ_H (mult., *J* in Hz, intensity)	HMBC	NOESY
1	176.8	-	-	-
2a	44.8	2.41 (*dd*, $J_{2a, 3}$ = 5.1, 1H)	1, 3, 4	2b, 3, 4
2b	44.8	2.50 (*dd*, $J_{2a, 2b}$ = 14.8, $J_{2b, 3}$ = 8.4, 1H)	1, 3, 4	2a, 3, 4
3	70.6	4.27 (*m*, 1H)	1''‡, 1‡	2a, 2b, 4, 1', 5'
4	18.8	1.21 (*d*, $J_{3, 4}$ = 6.1, 3H)	2, 3	2a, 2b, 3, 1'
1'	94.1	4.83 (*s*, 1H)	3', 5', 3‡	3, 4
2'	72.1	4.81 (*s.br*, $J_{1', 2'}$ = 2.6, 1H)	1'', 4'	4, 3'ax, 3'eq
3'ax	29.9	1.98 (*ddd*, $J_{2', 3'ax}$ = 3.1, $J_{3'ax, 3'eq}$ = 13.5, 1H)	1', 2', 4', 5'	2', 3'eq, 4', 5'
3'eq	29.9	2.11 (*ddd*, $J_{2', 3'eq}$ = 4.7, 1H)	1', 2', 4', 5'	2', 3'ax, 4'
4'	71.1	4.75 (*ddd*, $J_{3'ax, 4'}$ = 11.1, $J_{3'eq, 4'}$ = 4.0, 1H)	5', 6', 5''''	5', 6', 3'ax, 3'eq
5'	67.8	4.03 (*dq*, $J_{4', 5'}$ = 9.6, 1H)	n.d.	3, 3'ax, 4', 6'
6'	17.7	1.15 (*d*, $J_{5', 6'}$ = 6.2, 3H)	4', 5'	4', 5'
1''	171.9	-	-	-
2''a	43.0	2.56 (*dd*, $J_{2''a, 3}$ = 5.0, 1H)	1'', 3'', 4''	2''b, 3'', 4''
2''b	43.0	2.62 (*dd*, $J_{2''a, 2''b}$ = 14.8, $J_{2''b, 3''}$ = 8.4, 1H)	1'', 3'', 4''	2''a, 3'', 4''
3''	69.7	4.27 (*m*, 1H)	1'''‡, 1''‡, 2''‡	2''a, 2''b, 4'', 1''', 5'''
4''	19.0	1.24 (*d*, $J_{3'', 4''}$ = 6.1, 3H)	2'', 3''	2''a, 2''b, 3'', 1'''
1'''	97.4	4.68 (*s*, 1H)	3'', 3''', 5'''	3'', 4'', 2''', 3'''ax, 5'''
2'''	69.5	3.72 (*s.br*, $J_{1''', 2'''}$ = 3.2, 1H)	4'''	4'', 1''', 3'''ax, 3'''eq
3'''ax	35.6	1.74 (*ddd*, $J_{2''', 3'''ax}$ = 3.0, $J_{3'''ax, 3'''eq}$ = 13.1, 1H)	1''', 2''', 4''', 5'''	2''', 3'''eq, 4''', 5'''
3'''eq	35.6	1.93 (*ddd*, $J_{2''', 3'''eq}$ = 4.9, 1H)	1''', 2''', 4''', 5'''	2''', 3'''ax, 4'''
4'''	68.0	3.49 (*ddd*, $J_{3'''ax, 4'''}$ = 11.2, $J_{3'''eq, 4'''}$ = 3.9, 1H)	5''', 6'''	3'''ax, 3'''eq, 6'''
5'''	71.1	3.56 (*dq*, $J_{4''', 5'''}$ = 9.3, 1H)	n.d.	3'', 3'''ax, 6'''
6'''	17.9	1.21 (*d*, $J_{5''', 6'''}$ = 6.1, 3H)	4''', 5'''	4''', 5'''
1''''	161.8	-	-	-
2''''	43.4	3.25 (*d*, $J_{2'''', 3''''}$ = 6.6, 2H)	1'''', 3'''', 4'''', 5''''	3'''', 4''''
3''''	41.5	2.67 (*m*, 1H)	2'''', 5''''	2'''', 4''''
4''''	14.7	1.13 (*d*, $J_{3'''', 4''''}$ = 7.2, 3H)	2'''', 3'''', 5''''	2'''', 3''''
5''''	175.7	-	-	-

‡ indicates HMBC correlations summarized from the HMBC spectrum of an enriched SPE fraction containing ubas#34 [4'-UB-2'-(asc-C4)-asc-C4, **11**] (***supplementary file 1d – Figure 27***).

Table 17. ^{1}H (800 MHz) spectroscopic data of natural **ubas#35** [4'-UB-2'-(asc-C4)-asc-C5, **12**] (CD_3OD) isolated from the *exo*-metabolome of *P. quartusdecimus*. Chemical shift was referenced to ($CD_2\underline{H}OD$) = 3.31 ppm. NMR data are derived from spectra in ***supplementary file 1d – Figure 29***.

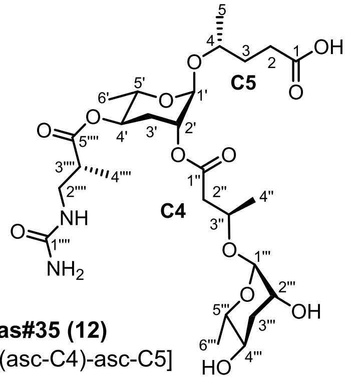

ubas#35 (12)
[4'-UB-2'-(asc-C4)-asc-C5]

Position	δ_H (mult., J in Hz, intensity)
1	-
2a	2.32 (*dd*, $J_{2a,\,2b}$ = 14.7, $J_{2a,\,3}$ = 4.2, 1H)
2b	2.36 (*dd*, $J_{2b,\,3}$ = 7.8, 1H)
3	1.84 (*m*, 1H)
4	3.85 (*m*, 1H)
5	1.18 (*d*, $J_{4,\,5}$ = 6.1, 3H)
1'	4.81 (*s*, 1H)
2'	4.83 (*s.br*, $J_{1',\,2'}$ = 2.4, 1H)
3'ax	2.01 (*ddd*, $J_{2',\,3'ax}$ = 3.1, $J_{3'ax,\,3'eq}$ = 13.7, 1H)
3'eq	2.13 (*ddd*, $J_{2',\,3'eq}$ = 4.7, 1H)
4'	4.78 (*ddd*, $J_{3'ax,\,4'}$ = 12.4, $J_{3'eq,\,4'}$ = 4.3, 1H)
5'	3.98 (*dq*, $J_{4',\,5'}$ = 9.2, 1H)
6'	1.17 (*d*, $J_{5',\,6'}$ = 6.2, 3H)
1''	-
2''a	2.56 (*dd*, $J_{2''a,\,2''b}$ = 15.0, $J_{2''a,\,3''}$ = 5.4, 1H)
2''b	2.62 (*dd*, $J_{2''b,\,3''}$ = 7.7, 1H)
3''	4.27 (*m*, 1H)
4''	1.24 (*d*, $J_{4'',\,5''}$ = 6.2, 3H)
1'''	4.68 (*s*, 1H)
2'''	3.73 (*s.br*, $J_{1''',\,2'''}$ = 2.9, 1H)
3'''ax	1.77 (*ddd*, $J_{2''',\,3'''ax}$ = 3.0, $J_{3'''ax,\,3'''eq}$ = 13.0, 1H)
3'''eq	1.95 (*ddd*, $J_{2''',\,3'''eq}$ = 6.4, 1H)
4'''	3.50 (*ddd*, $J_{3'''ax,\,4'''}$ = 11.6, $J_{3'''eq,\,4'''}$ = 4.1, 1H)
5'''	3.57 (*dq*, $J_{4''',\,5'''}$ = 9.4, 1H)
6'''	1.22 (*d*, $J_{5''',\,6'''}$ = 6.2, 3H)
1''''	-
2''''	3.27 (*d*, $J_{2'''',\,3''''}$ = 7.0, 2H)
3''''	2.67 (*m*, 1H)
4''''	1.16 (*d*, $J_{3'''',\,4''''}$ = 6.9, 3H)
5''''	-

Table 18. ^{1}H (800 MHz), ^{13}C (200 MHz), HMBC and NOESY NMR spectroscopic data of natural **ubas#28** [4'-UB-2'-(asc-C5)-asc-C4, **13**] (CD_3OD) isolated from the *exo*-metabolome of *P. fukushimae*. Carbon shifts were determined from HSQC and HMBC spectra. Chemical shifts were referenced to ($CD_2\underline{H}OD$) = 3.31 ppm and ($\underline{C}D_3OD$) = 49.0 ppm. n.d. denotes not detected. NMR data are derived from spectra in ***supplementary file 1d – Figures 36-42***.

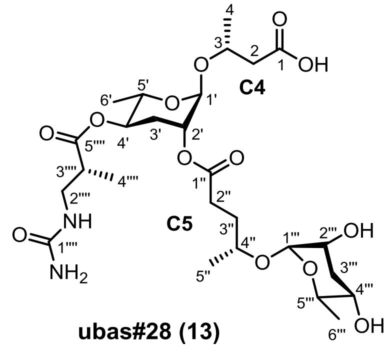

ubas#28 (13)
[4'-UB-2'-(asc-C5)-asc-C4]

Position	δC (ppm)	δH (mult., *J* in Hz, intensity)	HMBC	NOESY
1	176.2	-	-	-
2a	44.4	2.43 (*dd*, $J_{2a,3}$ = 4.9, 1H)	1, 3, 4	2b, 3, 4
2b	44.4	2.50 (*dd*, $J_{2a,2b}$ = 14.5, $J_{2b,3}$ = 8.3, 1H)	1, 3, 4	2a, 3, 4
3	70.5	4.28 (*m*, 1H)	n.d.	2a, 2b, 4, 1', 5'
4	18.8	1.21 (*d*, $J_{3,4}$ = 6.1, 3H)	2, 3	2a, 2b, 3, 1'
1'	94.3	4.80 (*s*, 1H)	3', 5', 3	3, 4, 2'
2'	71.9	4.81 (*s.br*, $J_{1',2'}$ = 2.6, 1H)	1''	1', 3'ax, 3'eq
3'ax	30.0	1.96 (*ddd*, $J_{2',3'ax}$ = 3.1, $J_{3'ax,3'eq}$ = 13.4, 1H)	1', 2', 4', 5'	2', 3'eq, 5'
3'eq	30.0	2.09 (*ddd*, $J_{2',3'eq}$ = 4.7, 1H)	1', 2', 4', 5'	2', 3'ax, 4', 6'
4'	71.1	4.72 (*ddd*, $J_{3'ax,4'}$ = 11.3, $J_{3'eq,4'}$ = 4.3, 1H)	n.d.	3'eq, 5', 6'
5'	67.7	4.01 (*dq*, $J_{4',5'}$ = 9.6, 1H)	n.d.	3'ax, 4', 6'
6'	17.8	1.15 (*d*, $J_{5',6'}$ = 6.2, 3H)	4', 5'	4', 5'
1''	173.7	-	-	-
2''	31.1	2.53 (*t*, $J_{2'',3''a}$ = 7.4, $J_{2'',3''b}$ = 6.6, 2H)	1'', 3'', 4''	3''a, 3''b, 4'', 5'', 5'''
3''a	33.0	1.82 (*m*, $J_{3''a,4''}$ = 6.6, 1H)	1'', 2'', 4'', 5''	2'', 3''b, 4'', 5''
3''b	33.0	1.87 (*m*, $J_{3''a,3''b}$ = 14.4, $J_{3''b,4''}$ = 7.1, 1H)	1'', 2'', 4'', 5''	2'', 3''a, 4'', 5''
4''	71.2	3.86 (*m*, 1H)	n.d.	2'', 3''a, 3''b, 5'', 1''', 5'''
5''	18.8	1.17 (*d*, $J_{4'',5''}$ = 6.1, 3H)	3'', 4''	2'', 3''a, 3''b, 4'', 1'''
1'''	97.2	4.66 (*s*, 1H)	4'', 3''', 5'''	4'', 5'', 2'''
2'''	69.7	3.73 (*s.br*, $J_{1''',2'''}$ = 2.9, 1H)	4'''	1''', 3'''ax, 3'''eq
3'''ax	35.7	1.77 (*ddd*, $J_{2''',3'''ax}$ = 3.1, $J_{3'''ax,3'''eq}$ = 13.1, 1H)	1''', 2''', 4''', 5'''	2''', 3'''eq, 4''', 5'''
3'''eq	35.7	1.95 (*ddd*, $J_{2''',3'''eq}$ = 4.9, 1H)	1''', 2''', 4''', 5'''	2''', 3'''ax, 4'''
4'''	68.1	3.51 (*ddd*, $J_{3'''ax,4'''}$ = 11.3, $J_{3'''eq,4'''}$ = 4.1, 1H)	5'''	3'''ax, 3'''eq, 5''', 6'''
5'''	71.2	3.59 (*dq*, $J_{4''',5'''}$ = 9.4, 1H)	4'''	2'', 4'', 3'''ax, 6'''
6'''	18.0	1.23 (*d*, $J_{5''',6'''}$ = 6.2, 3H)	4''', 5'''	4''', 5'''
1''''	161.8	-	-	-
2''''	43.4	3.25 (*d*, $J_{2'''',3''''}$ = 7.0, 2H)	1'''', 3'''', 4'''', 5''''	3'''', 4''''
3''''	41.5	2.66 (*m*, 1H)	2'''', 4'''', 5''''	2'''', 4''''
4''''	14.7	1.13 (*d*, $J_{3'''',4''''}$ = 6.9, 3H)	2'''', 3'''', 5''''	2'''', 3''''
5''''	175.8	-	-	-

Table 19. ^{1}H (800 MHz) NMR spectroscopic data of natural **upas#34** [4'-UP-2'-(asc-C4)-asc-C4, **14**] (CD_3OD) isolated from the *exo*-metabolome of *P. quartusdecimus*. Chemical shift was referenced to (CD_2<u>H</u>OD) = 3.31. NMR data are derived from spectra in ***supplementary file 1e – Figure 1***.

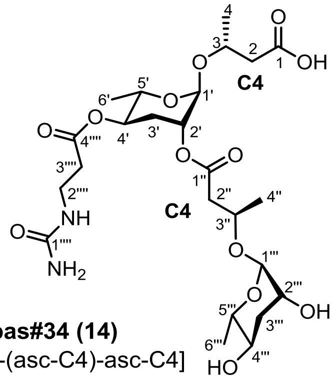

upas#34 (14)
[4'-UP-2'-(asc-C4)-asc-C4]

Position	δ_H (mult., *J* in Hz, intensity)
1	-
2a	2.38 (*dd*, $J_{2a, 3}$ = 6.8, 1H)
2b	2.49 (*dd*, $J_{2a, 2b}$ = 13.7, $J_{2b, 3}$ = 8.3, 1H)
3	4.28 (*m*, 1H)
4	1.20 (*d*, $J_{3, 4}$ = 6.4, 3H)
1'	4.77 (*s*, 1H)
2'	4.85 (1H)
3'ax	1.97 ($J_{3'ax, 3'eq}$ = 13.7, 1H)
3'eq	2.11 (1H)
4'	4.75 (1H)
5'	4.04 (*dq*, $J_{4', 5'}$ = 9.4, 1H)
6'	1.14 (*d*, $J_{5', 6'}$ = 6.1, 3H)
1''	-
2''a	2.57 (*dd*, $J_{2''a, 3''}$ = 7.6, 1H)
2''b	2.62 (*dd*, $J_{2''a, 2''b}$ = 13.4, $J_{2''b, 3''}$ = 8.2, 1H)
3''	4.27 (*m*, 1H)
4''	1.24 (*d*, $J_{3'', 4''}$ = 6.5, 3H)
1'''	4.68 (*s*, 1H)
2'''	3.72 (1H)
3'''ax	1.74 ($J_{3'''ax, 3'''eq}$ = 13.4, 1H)
3'''eq	1.93 (1H)
4'''	3.49 (1H)
5'''	3.57 (*dq*, $J_{4''', 5'''}$ = 9.6, 1H)
6'''	1.21 (*d*, $J_{5''', 6'''}$ = 6.2, 3H)
1''''	-
2''''	3.36 (*t*, $J_{2'''', 3''''}$ = 6.4, 2H)
3''''	2.51 (*t*, 2H)
4''''	-

Table 20. ^{1}H (800 MHz) NMR spectroscopic data of natural **upas#28** [4'-UP-2'-(asc-C5)-asc-C4, **15**] (CD_3OD) isolated from the *exo*-metabolome of *P. fukushimae*. Chemical shift was referenced to ($CD_2\underline{H}OD$) = 3.31 ppm. NMR data are derived from spectra in ***supplementary file 1e – Figure 2***.

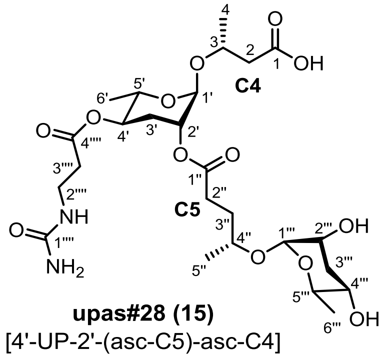

upas#28 (15)
[4'-UP-2'-(asc-C5)-asc-C4]

Position	δ_H (mult., *J* in Hz, intensity)
1	-
2a	2.29 (*dd*, $J_{2a,\,3}$ = 6.0, 1H)
2b	2.48 (*dd*, $J_{2a,\,2b}$ = 13.6, $J_{2b,\,3}$ = 8.0, 1H)
3	4.27 (*m*, 1H)
4	1.19 (*d*, $J_{3,\,4}$ = 6.1, 3H)
1'	4.79 (*s*, 1H)
2'	4.80 (*s.br*, $J_{1',\,2'}$ = 2.6, 1H)
3'ax	2.02 (*ddd*, $J_{2',\,3'ax}$ = 3.6, $J_{3'ax,\,3'eq}$ = 13.5, 1H)
3'eq	2.09 (*ddd*, $J_{2',\,3'eq}$ = 4.9, 1H)
4'	4.73 (*ddd*, $J_{3'ax,\,4'}$ = 11.3, $J_{3'eq,\,4'}$ = 4.5, 1H)
5'	4.07 (*dq*, $J_{4',\,5'}$ = 9.6, 1H)
6'	1.15 (*d*, $J_{5',\,6'}$ = 6.2, 3H)
1''	-
2''	2.51 (*t*, 2H)
3''a	1.82 (*m*, 1H)
3''b	1.86 (*m*, 1H)
4''	3.86 (*m*, 1H)
5''	1.18 (*d*, $J_{4'',\,5''}$ = 6.2, 3H)
1'''	4.67 (*s*, 1H)
2'''	3.73 (*s.br*, $J_{1''',\,2'''}$ = 3.0, 1H)
3'''ax	1.77 (*ddd*, $J_{2''',\,3'''ax}$ = 3.1, $J_{3'''ax,\,3'''eq}$ = 13.1, 1H)
3'''eq	1.95 (*ddd*, $J_{2''',\,3'''eq}$ = 4.9, 1H)
4'''	3.51 (*ddd*, $J_{3'''ax,\,4'''}$ = 11.3, $J_{3'''eq,\,4'''}$ = 4.2, 1H)
5'''	3.58 (*dq*, $J_{4''',\,5'''}$ = 9.6, 1H)
6'''	1.22 (*d*, $J_{5''',\,6'''}$ = 6.1, 3H)
1''''	-
2''''	3.36 (*t*, $J_{2'''',\,3''''}$ = 6.5, 2H)
3''''	2.51 (*t*, 2H)
4''''	-

References

Artyukhin AB, Zhang Y, Akagi AE, Panda O, Sternberg PW, Schroeder FC. 2018. Metabolomic "dark matter" dependent on peroxisomal β-oxidation in *Caenorhabditis elegans*. *Journal of the American Chemical Society* **140**:2841-2852. DOI: https://doi.org/10.1021/jacs.7b11811
