# Supplementary material for: Convergent evolution of small molecule pheromones in Pristionchus nematodes
Source: eLife. 2020 Apr 27;9:e55687. doi: 10.7554/eLife.55687 (PMC7224695; doi:10.7554/eLife.55687)
Supplement: Supplementary file 4. [file elife-55687-supp4.pdf]

supplementary file 4: Ancestral reconstruction results for individual compounds.

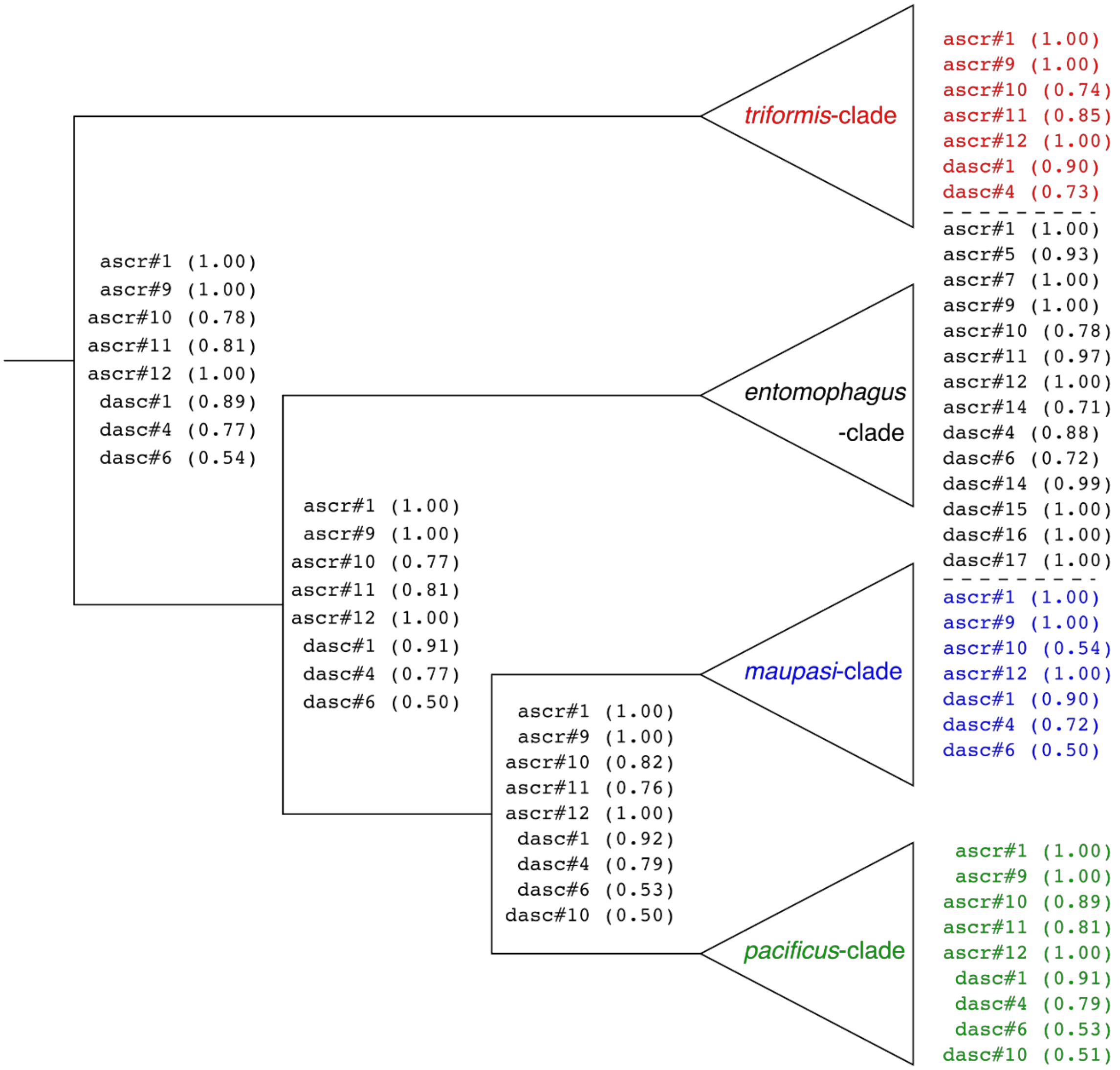

Figure 1. Summary of ancestral reconstruction results at key nodes in the *Pristionchus* phylogeny. The results of ancestral reconstruction results for individual NDMMs (provided in ***supplementary file 4 — Figure 2***) were extracted for the key nodes that define the major *Pristionchus* clades. Results show NDMMs predicted to be present at the given node (with probability provided in parentheses). Three NDMMs—ascr#1, ascr#9, and ascr#12—were not explicitly reconstructed: these compounds are present in all studied species and were therefore assumed to be present at all internal nodes with Prob = 1.0. Entries to the right correspond to predictions for the ancestral nodes for the respective *Pristionchus* clade (red = triformis; black = entomophagus; blue = maupasi; green = pacificus).

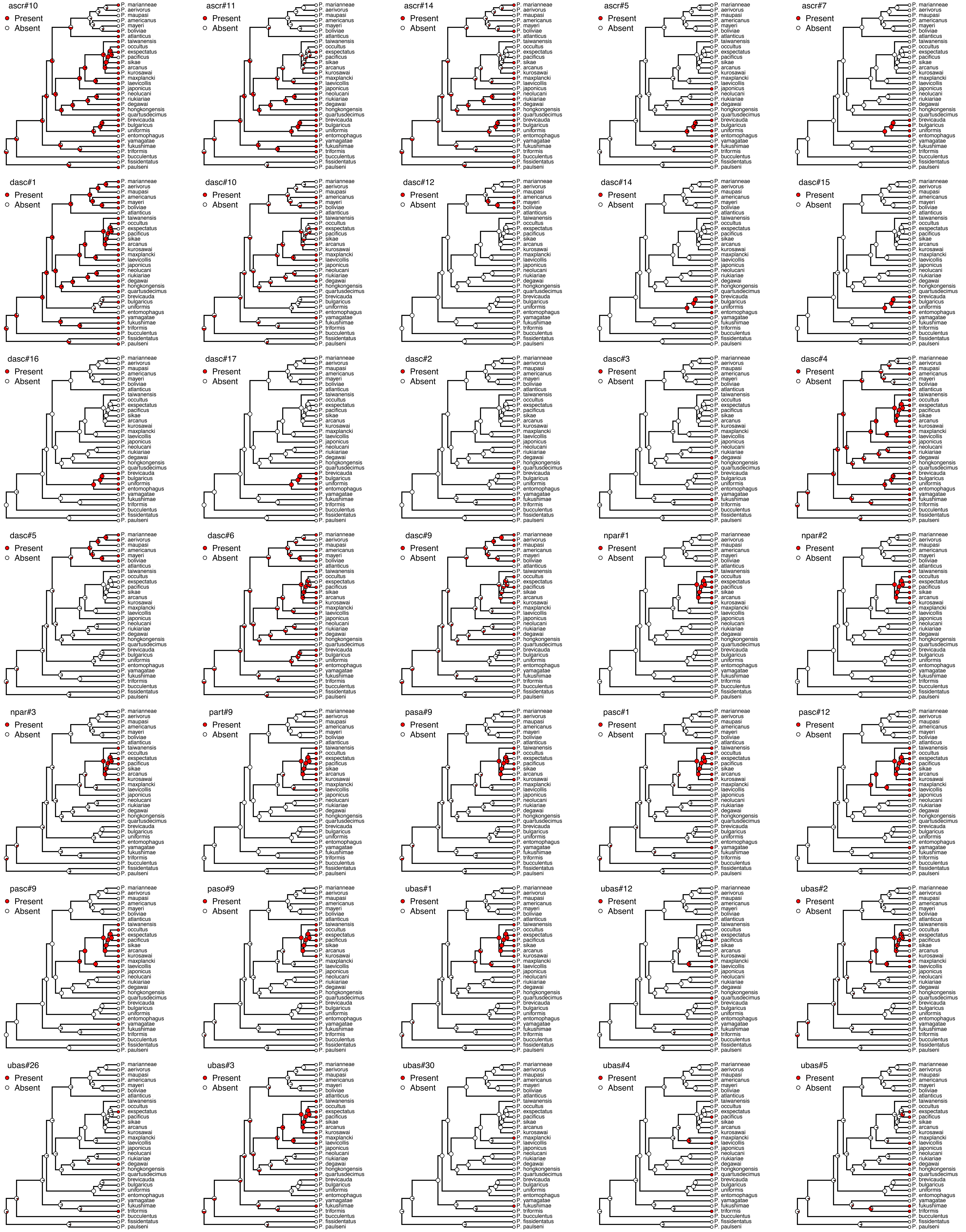
ascr#10
ascr#11
ascr#14
ascr#5
ascr#7
dasc#1
dasc#10
dasc#12
dasc#14
dasc#15
dasc#16
dasc#17
dasc#2
dasc#3
dasc#4
dasc#5
dasc#6
dasc#9
npar#1
npar#2
npar#3
part#9
pasa#9
pasc#1
pasc#12
pasc#9
paso#9
ubas#1
ubas#12
ubas#2
ubas#26
ubas#3
ubas#30
ubas#4
ubas#5
Present
Absent
P. marianneae
P. aerivorus
P. maupasi
P. americanus
P. mayeri
P. boliviae
P. atlanticus
P. taiwanensis
P. occultus
P. exspectatus
P. pacificus
P. sikae
P. arcanus
P. kurosawai
P. maxplancki
P. laevicollis
P. japonicus
P. neolucani
P. riukiariae
P. degawai
P. hongkongensis
P. quartusdecimus
P. brevicauda
P. bulgaricus
P. uniformis
P. entomophagus
P. yamagatae
P. fukushimae
P. triformis
P. bucculentus
P. fissidentatus
P. paulseni

Figure 2. Ancestral reconstruction results for individual NDMMs. Ancestral reconstruction was performed for all non-constant, non-singleton NDMMs listed in ***Figure 2*** of the main text.
